# Supplementary material for: How do the prevalence and relative risk of non-suicidal self-injury and suicidal thoughts vary across the population distribution of common mental distress (the p factor)? Observational analyses replicated in two independent UK cohorts of young people
Source: BMJ Open. 2020 May 11;10(5):e032494. doi: 10.1136/bmjopen-2019-032494 (PMC7223145; doi:10.1136/bmjopen-2019-032494)
Supplement: Supplementary data [file bmjopen-2019-032494supp001.pdf]

Bifactor modelling:

Bifactor psychometric modelling is designed to extract variance common for all items in the model to generate one "general" factor. In addition to this general factor, specific factor/s may emerge, which are uncorrelated with each other or with the general factor. Specific factor/s contain the remaining variance after the extraction of the general factor[1]. St Clair et al. (2017) found in her psychometric study a bifactor model with one general factor and 5 specific factors, which fitted the data better than the correlated-factors model or second-order model. In our study, we first replicated St Clair et al. (2017) psychometric model in Cohort 1 (T1, T2, T3) and Cohort 2. In accordance with the original study, in our psychometric modelling the same measures of common mental illness frequently emerging during adolescence (depression, anxiety, psychotic experiences, obsessions and compulsions, conduct problems) as well as traits and characteristics commonly considered to contribute to mental wellness (well-being, self-esteem) were used as constructs contributing the general factor (see items below). Having replicated St Clair et al (2017) bifactor model, we then computed factor scores for the general factor – here termed *Common Mental Distress (CMD).*

The confirmatory bifactor analysis in Cohort 1 was computed with the multiple group method (MGM) in Mplus 8 with the three data point used as a grouping variable; the same model was fitted to the data in each group. MGM in Mplus by default holds thresholds and loadings invariant across groups[2], thus allowing the comparison if the model fits data well in all groups under study (here data from the three measurement points). The effective sample for the 3 data waves was, respectively, n=2403, n=1815, n=1245 (Total N=5463). The overall chi-square test for the model was $\chi 2$=33648.24 (*df*=14983, *p*=0.000), for Time 1 it was $\chi 2$=14791.20, for Time 2 it was $\chi 2$=10400.56 and for Time 3 it was $\chi 2$=8456.47. The overall Root Mean Square Error of Approximation (RMSEA) for the model was 0.026 (0.026-0.027), Comparative Fit Index (CFI) was 0.969, Tucker-Lewis Index (TLI) was 0.969, and weighted root mean square residual (WRMR) was 2.91. The confirmatory bifactor analysis was used in Cohort 2 as well. The following fit indexes were obtained in Cohort 2: $\chi 2$=7602.17 (df=4462, *p*=0.000), RMSEA=0.026 (0.025-0.027), CFI=0.96, TLI=0.96, WRMR= 1.34. The above-cited fit indexes suggest that the bifactor model fitted the data well in both cohorts.

In both analyses – for Cohort 1 and 2 – we used WLSMV estimator and THETA parametrisation with PROBIT link, and all items were treated as ordered-categorical variables.

Much debate in the literature has focused on the issue of interpretability of specific factors, i.e., whether they should be considered as measures of meaningful concepts or should be treated as comprising the residual, uninterpretable variance[3]. The general factor in St Clair et al (2017) study demonstrated high reliability and validity, as well as low measurement error compared to validity and error of the specific factors. As follows, we focused in our study only on this general (CMD) factor; we did not attempt to interpret or use in our analyses the specific factors, even though they emerged in our bifactor modelling, due to their relatively high measurement error and ambiguity of their theoretical interpretation. The list of items contributing to CMD factor with factor loadings on this factor in Cohort 1 (T1, T2, T3) and Cohort 2 are listed below in Supplementary Table 1.

Polek E, *et al. BMJ Open* 2020; 10:e032494. doi: 10.1136/bmjopen-2019-032494

Multiple imputation procedure in Cohort 1:

Missingness in Cohort 1 predominantly arose from longitudinal attrition – 24% at T2 and 48% at T3; a small fraction of data was also missing due to omissions of items (between 0 to 6%). Before performing imputations, we examined if longitudinal attrition was related to demographic variables and other variables under study. Indeed, we found small, yet statistically significant correlations between attrition at T2 and T3 and demographic and exposure variables at T1 (see Supplementary Table 8), thus indicating that the assumption of "missing completely at random (MCAR) is not met. Moreover, we performed Little's MCAR test and found that it was significant (p<.001). Therefore, we assumed that MAR condition was met. As follows, we imputed missing data under MAR condition in Cohort 1 at T2 and T3 with the following variables in one imputation model: CMD factor scores, NSSI and ST variables. We used the following auxiliary variables: research points, sex, age, ethnicity, and Index of Multiple Deprivation (IMD) (as an indicator of a socioeconomic status[4]) as predictors of the missingness, in addition to main predictors – CMD factor scores, NSSI, and ST at T1.

Multiple imputations were computed in R program with MICE package[5]; convergence was examined by visual inspection of MCMC chains (with a maximum number of 20 iterations per chain and Gibbs sampling). Fifty-four (N=2403) datasets were generated to equal the percentage of missing data in CMD, NSSI, and ST at T3[6]. In terms of the imputation model, we used mean matching for continuous variables (CMD factor scores) and logistic regression for binary variables (NSSI and ST). The imputed 54 datasets were then used in pathway analysis (see the main manuscript and Supplementary Figure 3 for details) with MLM estimator in Mplus 7.4, which automates the process of analysing and combining parameter estimates from each imputed dataset using Rubin's rules[7].

Polek E, *et al. BMJ Open* 2020; 10:e032494. doi: 10.1136/bmjopen-2019-032494

Supplementary Table 1: List of all items used in the study

Outcome measures:

Suicidal Thought (ST)

I thought about killing myself (MFQ19, response options: *Always, Mostly, Sometimes, Never*)**Cohort 1 & 2**

This is one of the 4 items assessing suicidal thoughts in the 33-item Mood and Feelings Questionnaire (MFQ)[8] : MFQ16 - I thoughts that life was not worth living; MFQ17 - I thought about dying; MFQ18 – I thought my family would be better off without me; MFQ19 - I thought about killing myself. We used item 19, as it had the highest (.70) loading on this sub-subscale. Responses to this item were recoded into a binary format: no ST (original response option *Never*) and ST (original response options *Sometimes* or *Mostly* or *Always*). We did not include MFQ items 16-18 in CMD factor to avoid content overlap between the outcome measure (ST) and the predictor – the CMD factor.

Non-Suicidal Self-Injury (NSSI)

NSSI in Cohort 1 was assessed with one question from the Drug, Alcohol and Self-Injury (DASI) questionnaire asking about engaging in self-injury without suicidal intent during the last month:

In the last month, have you tried to hurt yourself on purpose without trying to kill yourself? (Response options: *Yes, No)*

NSSI in Cohort 2 was assessed with one question from the DASI questionnaire asking about life-time occurrence of NSSI:

Supplementary Table 9: Items comprising the Common Mental Distress (CMD) factor

**Items and associated measures**	**Standardised Factor Loadings**			
**The Moods and Feelings Questionnaire (MFQ)[11] Cohort 1 & 2** **(response options: *Always, Mostly, Sometimes, Never*)** *Note:* 4 items measuring suicidality were excluded to avoid content overlap between the measures of variables treated here as predictors (CMD, Depression) and the outcome variable (ST). We excluded 4 other items which caused model convergence problems: *I was less hungry than usual (MFQ3), I ate more than usual (MFQ4), It was hard for me to make up my mind (MFQ10), I slept a lot more than usual (MFQ33)*	**Cohort 1**			**Cohort 2**
	**Time 1**	**Time 2**	**Time 3**	
1. I felt miserable or unhappy. (MFQ1)	.69	.73	.71	.73
2. I didn't enjoy anything. (MFQ2)	.62	.70	.72	.67
3. I felt so tired I just sat around and did nothing. (MFQ5)	.53	.56	.57	.54

4. I was moving and walking more slowly than usual. (MFQ6)	.54	.59	.54	.52
5. I was very restless. (MFQ7)	.48	.54	.56	.49
6. I felt I was no good any more. (MFQ8)	.78	.82	.84	.77
7. I sometimes blamed myself for things that weren't my fault. (MFQ9)	.70	.74	.75	.73
8. I got grumpy and cross easily. (MFQ11)	.60	.65	.68	.65
9. I felt like talking a lot less than usual. (MFQ12)	.64	.66	.69	.65
10. I was talking more slowly than usual. (MFQ13)	.56	.64	.55	.59
11. I cried a lot. (MFQ14)	.64	.64	.68	.69
12. I thought there was nothing good for me in the future. (MFQ15)	.72	.77	.78	.72
13. I didn't want to see my friends. (MFQ20)	.69	.73	.70	.66
14. I found it hard to think properly or concentrate. (MFQ21)	.73	.77	.77	.72
15. I thought bad things would happen to me. (MFQ22)	.76	.77	.80	.81
16. I hated myself. (MFQ23)	.81	.82	.85	.80
17. I was a bad person. (MFQ24)	.73	.76	.78	.72
18. I thought I looked ugly. (MFQ25)	.65	.70	.70	.69
19. I worried about aches and pains. (MFQ26)	.46	.50	.50	.56
20. I felt lonely. (MFQ27)	.70	.74	.73	.74
21. I thought nobody really loved me. (MFQ28)	.75	.79	.83	.76
22. I didn't have any fun at school / college / work. (MFQ29)	.62	.67	.66	.58
23. I thought I could never be as good as other people my age. (MFQ30)	.76	.79	.78	.76
24. I did everything wrong. (MFQ31)	.83	.85	.87	.82
25. I didn't sleep as well as usual. (MFQ32)	.53	.57	.61	.60
**The Revised Children's Manifest Anxiety Scale (RCMAS)[12] Cohort 1 & 2**<br>**(response options: *Always, Mostly, Sometimes, Never*)**				
1. I had trouble making up my mind. (RCMAS1)	.60	.68	.71	.59
2. I worried when things did not go the right way for me. (RCMAS2)	.71	.77	.79	.78
3. Others seemed to do things more easily than I could. (RCMAS3)	.76	.80	.83	.76
4. Often I had trouble getting a breath. (RCMAS4)	.56	.60	.59	.55
5. I worried a lot of the time. (RCMAS5)	.78	.80	.82	.78
6. I was afraid of a lot of things. (RCMAS6)	.78	.80	.82	.77
7. I got angry easily. (RCMAS7)	.63	.68	.74	.68

Polek E, *et al. BMJ Open* 2020; 10:e032494. doi: 10.1136/bmjopen-2019-032494

8. I worried about what my parents would say to me. (RCMAS8)	.62	.67	.71	.65
9. I felt that others did not like the way I did things. (RCMAS9)	.73	.79	.78	.74
10. It was hard for me to get to sleep at night. (RCMAS10)	.55	.63	.58	.57
11. I worried about what other people thought about me. (RCMAS11)	.74	.79	.80	.71
12. I felt alone even when there were people with me. (RCMAS12)	.80	.84	.86	.85
13. Often I felt sick to my stomach. (RCMAS13)	.69	.74	.74	.76
16. I was tired a lot. (RCMAS16)	.62	.67	.69	.65
17. I worried about what was going to happen. (RCMAS17)	.77	.80	.81	.79
18. Other people my age were happier than me. (RCMAS18)	.79	.83	.83	.79
19. I had bad dreams. (RCMAS19)	.54	.59	.57	.62
20. My feelings got hurt easily when I was fussed at. (RCMAS20)	.75	.76	.78	.77
21. I felt someone would tell me I did things the wrong way. (RCMAS21)	.70	.77	.77	.71
22. I wake up scared some of the time. (RCMAS22)	.64	.74	.72	.67
23. I worried when I went to bed at night. (RCMAS23)	.67	.74	.73	.75
24. It was hard for me to keep my mind on my work. (RCMAS24)	.48	.58	.56	.55
25. I wiggled in my seat a lot. (RCMAS25)	.77	.79	.80	.76
27. A lot of people were against me. (RCMAS27)	.75	.80	.83	.80
28**.** I often worried about something bad happening to me. (RCMAS28)	.74	.79	.79	.80
**The Revised Leyton Obsessional Inventory (R-LOI)[13 Cohort 1 & 2]** **(response options: *Always, Mostly, Sometimes, Never*)**				
1. I felt I had to do things in a certain way, like counting or saying special words, to stop something bad from happening. (R-LOI1)	.53	.58	.50	.47
2. I had trouble finishing my homework or other jobs because I had to do things over and over again. (R-LOI2)	.58	.63	.64	.53
3. I hated dirt and dirty things. (R-LOI3)	.35	.44	.43	.39
4. I had a special number that I counted up to, or I felt I had to do things just that number of times. (R-LOI4)	.40	.46	.42	.41
5. I often felt guilty or bad about things I had done even though no one else thought I had done anything wrong. (R-LOI5)	.71	.77	.79	.73
6. I worried about being clean enough. (R-LOI6)	.48	.51	.55	.45

7. I moved or talked in a special way to avoid bad luck. (R-LOI7)	.38	.46	.38	.33
8. I worried a lot if I did something, not exactly the way I liked. (R-LOI8)	.60	.67	.66	.53
9. I was fussy about keeping my hands clean. (R-LOI9)	.35	.40	.41	.35
10. I had special numbers or words that I said because I hoped they kept bad luck or bad things away. (R-LOI10)	.43	.47	.47	.42
11. I kept thinking about the things that I had done because I wasn't sure that they were the right things to do. (R-LOI11)	.71	.73	.71	.67

**Antisocial Behaviour Questionnaire (ABQ)[14 Cohort 1 & 2]** **(response options: *Always, Mostly, Sometimes, Never*)**				
1. I deliberately broke the rules or disobeyed people (e.g. parents, teachers or supervisors). (ABQ1)	.45	.48	.47	.38
2. I stole things (e.g. from home or a shop or school). (ABQ2)	.37	.40	.36	.26
3. I deliberately damaged property (e.g. broke windows or chairs or wrote graffiti or started fires). (ABQ3)	.35	.39	.39	.38
4. I skipped lessons/work, skived, or played truant from school. (ABQ5)	.36	.39	.40	.35
5. I deliberately lied or cheated to get what I wanted. (ABQ6)	.43	.39	.41	.40
6. I ran away from home (e.g. for half a day or overnight). (ABQ7)	.51	.56	.58	.56
**Rosenberg Self-Esteem Questionnaire (RSEQ)[15 Cohort 1 & 2]** **(response options: *Always, Mostly, Sometimes, Never*)**				
1. At times, I thought I was no good at all. (RSEQ1)	.82	.84	.85	.83
2. I was satisfied with myself. (RSEQ2)	-.58	-.61	-.60	-.53
3. I felt I had a number of good qualities. (RSEQ3)	-.53	-.55	-.56	-.52
4. I was able to do things as well as most people. (RSEQ4)	-.56	-.60	-.62	-.56
5. I felt I did not have much to be proud of. (RSEQ5)	.70	.73	.72	.70
6. I certainly felt useless at times. (RSEQ6)	.79	.81	.79	.77
7. I felt that I was as good as anyone else. (RSEQ7)	-.53	-.56	-.54	-.44
8. I wished I could have more respect for myself. (RSEQ8)	.62	.66	.68	.69
9. I felt that I was a failure. (RSEQ9)	.80	.82	.83	.75
10. I took a positive attitude toward myself. (RSEQ10)	-.60	-.63	-.63	-.56
**Warwick-Edinburgh Mental Well-Being Scale (WEMWBS)[16 Cohort 1 & 2]** **(response options: *None of the time, Rarely, Some of the time, Often, All of the time*)**				
1. I've been feeling optimistic about the future. (WEMWBS1)	-.46	-.51	-.54	-.25
2. I've been feeling useful. (WEMWBS2)	-.52	-.58	-.60	-.33
3. I've been feeling relaxed. (WEMWBS3)	-.57	-.62	-.63	-.49
4. I've had the energy to spare. (WEMWBS5)	-.40	-.46	-.49	-.36
5. I've been dealing with problems well. (WEMWBS6)	-.57	-.63	-.64	-.46
6. I've been thinking clearly. (WEMWBS7)	-.62	-.67	-.68	-.48
7. I've been feeling good about myself. (WEMWBS8)	-.65	-.71	-.70	-.55
8. I've been feeling close to other people. (WEMWBS9)	-.44	-.50	-.52	-.28

Polek E, *et al. BMJ Open* 2020; 10:e032494. doi: 10.1136/bmjopen-2019-032494

9. I've been feeling confident. (WEMWBS10)	-.58	-.63	-.66	-.46
10. I've been able to make up my own mind about things. (WEMWBS11)	-.52	-.59	-.60	-.39
11. I've been feeling loved. (WEMWBS12)	-.49	-.54	-.60	-.29
12. I've been interested in new things. (WEMWBS13)	-.36	-.45	-.46	-.20
13. I've been feeling cheerful. (WEMWBS14)	-.61	-.67	-.67	-.49
**Psychotic-Like Experiences:**<br>**Cohort 1 – selected 10 items from the Schizotypal Personality Questionnaire (SPQ)**[17]<br>**Cohort 2 – selected 7 items from the Diagnostic Interview Schedule for Children (DISC)**[18]<br>**(response options: *Yes, No*)**				
1. Have you often mistaken objects or shadows for people or noises for voices? (SPQ4) **Cohort 1**	.38	.43	.41	*Not used*
2. I am sure I am being talked about behind my back. (SPQ9, DISC3) **Cohort 1 & 2**	.59	.67	.66	.60
3. Have you ever had the sense that some person or force is around you, even though you cannot see anyone? (SPQ13, DISC5) **Cohort 1 & 2**	.33	.38	.34	.41
4. Have you ever noticed a common event or object that seemed to be a special sign for you? (SPQ28, DISC8) **Cohort 1 & 2**	.33	.33	.35	.38
5. I often hear a voice speaking my thoughts aloud. (SPQ31, DISC10) **Cohort 1 & 2**	.33	.39	.34	.40
6. Have you ever seen things invisible to other people? (SPQ40, DISC13) **Cohort 1 & 2**	.36	.50	.37	.48
7. Do you sometimes feel that other people are watching you? (SPQ60, DISC19) **Cohort 1 & 2**	.53	.55	.59	.54
8. Do you ever suddenly feel distracted by distant sounds that you are not normally aware of? (SPQ61) **Cohort 1**	.40	.49	.45	*Not used*
9. Do you sometimes feel that people are talking about you? (SPQ63, DISC15) **Cohort 1 & 2**	.52	.56	.59	.60
10. Are your thoughts sometimes so strong that you can almost hear them? (SPQ64) **Cohort 1**	.44	.52	.50	*Not used*

Polek E, *et al. BMJ Open* 2020; 10:e032494. doi: 10.1136/bmjopen-2019-032494

Supplementary Table 2: Predictive power of Common Mental Distress versus the conventional psychopathology dimensions in Cohort 1_{T1} and Cohort 2: AUC (for ST and NSSI as criteria) and ORs for continuous and binary predictors (with cut-off point of 1SD)

		AUC		Suicidal thought (ST)				Non-suicidal self-injury (NSSI)			
				Continuous predictor		Binary (1SD cut-off)		Continuous predictor		Binary (1SD cut-off)	
		ST	NSSI	OR	95% C.I.	OR	95% C.I.	OR	95% C.I.	OR	95% C.I.
Common Mental Distress	Cohort 1_{T1}	.87	.83	7.07	[5.66 - 8.84]	15.60	[11.56 - 21.06]	4.15	[3.44 - 5.01]	8.93	[6.63 - 12.03]
	Cohort 2	.88	.72	6.79	[4.51 - 10.21]	20.97	[6.47 - 67.92]	2.38	[1.90 - 2.98]	4.00	[2.55 - 6.28]
Depression	Cohort 1_{T1}	.88	.83	5.10	[4.28 - 6.07]	15.60	[11.56 - 21.06]	3.21	[2.77 - 3.72]	8.28	[6.15 - 11.14]
	Cohort 2	.88	.70	7.18	[4.77 - 10.80]	15.32	[8.52 - 27.57]	2.14	[1.73 - 2.64]	3.56	[2.32 - 5.46]
Anxiety	Cohort 1_{T1}	.85	.81	4.82	[4.04 - 5.75]	13.62	[10.11 - 18.34]	3.75	[3.16 - 4.45]	7.61	[5.67 - 10.22]
	Cohort 2	.86	.71	5.69	[3.90 - 8.29]	10.51	[5.89 - 18.73]	2.24	[1.81 - 2.77]	3.68	[2.39 - 5.67]
Self-esteem (reversed)	Cohort 1_{T1}	.85	.83	4.81	[4.00 - 5.79]	15.62	[11.49 - 21.23]	3.75	[3.16 - 4.45]	9.86	[7.28 - 13.35]
	Cohort 2	.87	.65	6.42	[4.24 - 9.74]	15.16	[8.32 - 27.62]	1.79	[1.45 - 2.21]	3.34	[2.20 - 5.07]
Well-being (reversed)	Cohort 1_{T1}	.82	.80	4.29	[3.59 - 5.13]	10.31	[8.06 - 13.19]	3.45	[2.90 - 4.09]	6.66	[4.93 - 8.99]
	Cohort 2	.78	.61	2.88	[2.11 - 3.93]	5.27	[3.01 - 9.24]	1.44	[1.18 - 1.76]	2.19	[1.40 - 3.42]
Psychotic-like experiences	Cohort 1_{T1}	.74	.73	2.70	[2.32 - 3.13]	4.94	[3.70 - 6.60]	2.36	[2.03 - 2.74]	4.03	[2.98 - 5.45]
	Cohort 2	.74	.71	2.65	[2.00 - 3.50]	6.78	[3.89 - 11.83]	2.11	[1.72 - 2.58]	4.11	[2.69 - 6.27]
Antisocial trait*	Cohort 1_{T1}	.64	.63	1.65	[1.45 - 1.88]	2.67	[1.96 - 3.63]	1.79	[1.56 - 2.05]	2.48	[1.78 - 3.47]
Schizotypal trait	Cohort 1_{T1}	.79	.78	3.14	[2.71 - 3.64]	6.26	[4.70 - 8.32]	2.77	[2.39 - 3.21]	6.08	[4.52 - 8.19]
	Cohort 2	.76	.72	1.98	[1.66 - 2.36]	5.66	[3.23 - 9.91]	2.41	[1.93 - 3.01]	4.45	[2.90 - 6.83]
Conduct problems	Cohort 1_{T1}	.69	.67	1.87	[1.66 - 2.10]	3.38	[2.52 - 4.52]	1.67	[1.49 - 1.87]	3.46	[2.54 - 4.71]
	Cohort 2	.68	.61	2.00	[1.58 - 2.53]	3.78	[2.16 - 6.63]	1.54	[1.29 - 1.84]	2.13	[1.36 - 3.34]
Obsessions & compulsions	Cohort 1_{T1}	.76	.72	2.18	[1.94 - 2.45]	5.74	[4.25 - 7.75]	1.76	[1.57 - 1.98]	3.55	[2.58 - 4.89]
	Cohort 2	.71	.63	1.57	[1.31 - 1.88]	4.16	[2.37 - 7.28]	2.11	[1.64 - 2.71]	2.75	[1.79 - 4.22]

** measures were available only for Cohort 1_{T1}*

Polek E, *et al. BMJ Open* 2020; 10:e032494. doi: 10.1136/bmjopen-2019-032494

Supplementary Table 3: Association between ST and demographic variables in Cohort 1 (T1, T2, T3) and Cohort 2 (polychoric correlations)

	ST Cohort 1			ST Cohort 2
	T1	T2	T3	
Socioeconomic status (IMD)	-.05	-.01	-.01	.02
Research centre (0-Cambridge, 1-London)	-.12	-.04	-.03	not applicable
Ethnicity (1-white; 0-other)	-.08	-.02	-.04	-0.01
Age	-.05	-.02	-.05	0.03
Gender (0-Female, 1-Male)	-.10	-.08	-.01	0.03

All p-values non-significant

Supplementary Table 4: Association between NSSI and demographic variables in Cohort 1 (T1, T2, T3) and Cohort 2 (polychoric correlations)

	Cohort 1			Cohort 2
	T1	T2	T3	
Socioeconomic status (IMD)	.00	.00	.02	-.01
Research centre (0-Cambridge, 1-London)	-.01	-.01	.00	not applicable
Ethnicity (1-white; 0-other)	.00	.00	.00	.00
Age	-.02	-.04	-.01	-.02
Gender (0-Female, 1-Male)	.05	-.23	.02	.08

All p-values non-significant

Supplementary Table 5: Association between CMD and demographic variables in Cohort 1 (T1, T2, T3) and Cohort 2 (polychoric correlations)

	Cohort 1			Cohort 2
	T1	T2	T3	
Socioeconomic status (IMD)	-.02	-.02	-.01	.02
Research centre (0-Cambridge, 1-London)	.07*	.01	.01	not applicable
Ethnicity (1-white; 0-other)	-.08**	-.04	-.04	.04
Age	.01	.01	.01	.01
Gender (0-Female, 1-Male)	-.15**	-.15*	-.11**	.20**

*p<.01, **p<.001

Supplementary Table 6: Test of change in the prevalence of NSSI in Cohort 1: frequency over three time points (chi-square test)

	T1	T2	T3
NSSI	223	199	197
No-NSSI	2180	2204	2206

Chi-square=2.22, *df*=2, *p*=0.32, Yates' chi-square =2.04, *p*=0.35

Supplementary Table 7: Test of change in the prevalence of ST in Cohort 1: frequency over three time points (chi-square test)

	T1	T2	T3
NSSI	243	274	281
No-NSSI	2160	2129	2122

Chi-square=3.45, *df*=2, *p*=0.17, Yates' chi-square =3.26, *p*=0.19

Supplementary Table 8: Association between attrition in Cohort 1 at T2 and T3 and other variables in the study (Spearman rho)

	**Attrition Cohort 1**	
T1 variables:	**T2**	**T3**
Socioeconomic status (IMD index)[#]	-.07**	-.05*
Research centre (0-Cambridge, 1-London)	.05*	.05*
Ethnicity (1-white; 0-other)	-.05*	-.05*
Age	.07**	.05*
Gender (0-Female, 1-Male)	.09**	.12**
NSSI	-.01	.00
ST	-.01	-.03
Common Mental Distress	.06*	.05*
Depression	.06**	.05*
Impulsivity	.10**	.14**
Anxiety	.04*	.04*
Self - esteem (reversed)	.07**	.06*
Well - being (reversed)	.06*	.05*
Psychotic - like experiences even coerced	.00	.01
Antisocial trait	.08**	.12**
Schizotypal trait	.04*	.03
Conduct problems	.10**	.13**
Obsessions & compulsions	.03	.03

**$p<.001$, *$p<.01$
[#]higher number indicated *lower* socioeconomic deprivation

Polek E, *et al. BMJ Open* 2020; 10:e032494. doi: 10.1136/bmjopen-2019-032494

Supplementary Table 9: Direct and indirect effects in mediation (pathway) models in a female (F), male (M) and total (T) sample

		Standardised				Non- standardised			
		Coeff.	S.E.	Lower 95% C.I.	Upper 95% C.I.	Coeff.	S.E.	Lower 95% C.I.	Upper 95% C.I.
$NSSI_{T1}$->CMD_{T2}	F	.14***	.03	.09	.19	.46***	.09	.30	.61
	M	13***	.03	.07	.18	.56***	.14	.32	.80
	T	.15***	.02	.11	.18	.53***	.07	.40	.66
$NSSI_{T1}$->$NSSI_{T3}$	F	.16**	.05	.07	.25	.54**	.17	.25	.83
	M	.14**	.05	.05	.23	.65**	.24	.25	1.05
	T	.15**	.03	.09	.22	.58**	.14	.34	.82
$NSSI_{T1}$->ST_{T3}	F	.07	.05	.00	.16	.27	.17	-.01	.56
	M	.04	.05	-.03	.13	.22	.24	-.18	.62
	T	.05	.03	.00	.12	.22	.15	-.02	.47
ST_{T1}->CMD_{T2}	F	.25***	.03	.19	.30	.83***	.10	.66	1.00
	M	.24***	.03	.18	.30	.85***	.11	.65	1.05
	T	.24***	.02	.20	.28	.83***	.07	.70	.96
ST_{T1}->$NSSI_{T3}$	F	.10*	.05	.01	.20	.38	.19	.05	.70
	M	.07	.06	-.03	.17	.25	.23	-.13	.64
	T	.19*	.04	.13	.25	.33	.16	.06	.60
ST_{T1}->ST_{T3}	F	.20***	.04	.13	.27	.76***	.16	.49	1.03
	M	.17***	.05	.08	.25	.66***	.19	.33	.98
	T	.19**	.03	.13	.25	.72***	.13	.50	.95
CMD_{T2}-> $NSSI_{T3}$	F	.22***	.07	.11	.34	.24**	.07	.11	.37
	M	.21*	.08	.07	.34	.21*	.09	.06	.36
	T	.22***	.06	.11	.32	.22***	.06	.11	.34
CMD_{T2}-> ST_{T3}	F	.32***	.05	.22	.41	.35***	.07	.23	.47
	M	.35***	.06	.25	.46	.39***	.07	.26	.51
	T	.33***	.04	.25	.40	.35***	.05	.26	.45
NSS_{T1}<->ST_{T1}	F	.40***	.02	.36	.45	.04***	.00	.03	.04
	M	.32***	.03	.26	.37	.02***	.00	.01	.03
	T	.37***	.02	.33	.40	.03***	.00	.02	.03
$NSSI_{T3}$<->ST_{T3}	F	.67***	.07	.55	.79	.67***	.07	.55	.79
	M	.57***	.10	.39	.75	.57***	.10	.39	.75
	T	.63***	.07	.51	.75	.63***	.07	.51	.75
$NSSI_{T1}$->CMD_{T2} ->$NSSI_{T3}$	F	.03**	.01	.01	.05	.11**	.04	.04	.18
	M	.02*	.01	.00	.05	.12*	.06	.02	.22

	T	**.03***	.01	.01	.05	***.12****	.04	.05	.19
**ST_{T1}->CMD_{T2}->$NSSI_{T3}$**	F	**.05****	.02	.02	.09	***.20****	.07	.08	.32
	M	**.05***	.02	.01	.08	***.18****	.08	.05	.31
	T	**.05****	.01	.02	.07	***.19*****	.05	.09	.28
**$NSSI_{T1}$->CMD_{T2}->ST_{T3}**	F	**.04*****	.01	.02	.06	***16*****	.04	.08	.24
	M	**.04*****	.01	.02	.07	***.22****	.07	.10	.33
	T	**.05****	.01	.03	.06	***.19*****	.04	.12	.26
**ST_{T1}->CMD_{T2}->ST_{T3}**	F	**.08*****	.01	.05	.11	***.29*****	.07	.17	.41
	M	**.08*****	.02	.05	.12	***.33*****	.08	.20	.47
	T	**.08****	.01	.05	.10	***.30*****	.05	.20	.39

*Significance levels:**$p<.05$, **$p<.01$, ***$p<.001$

Item Response Theory (IRT) analysis

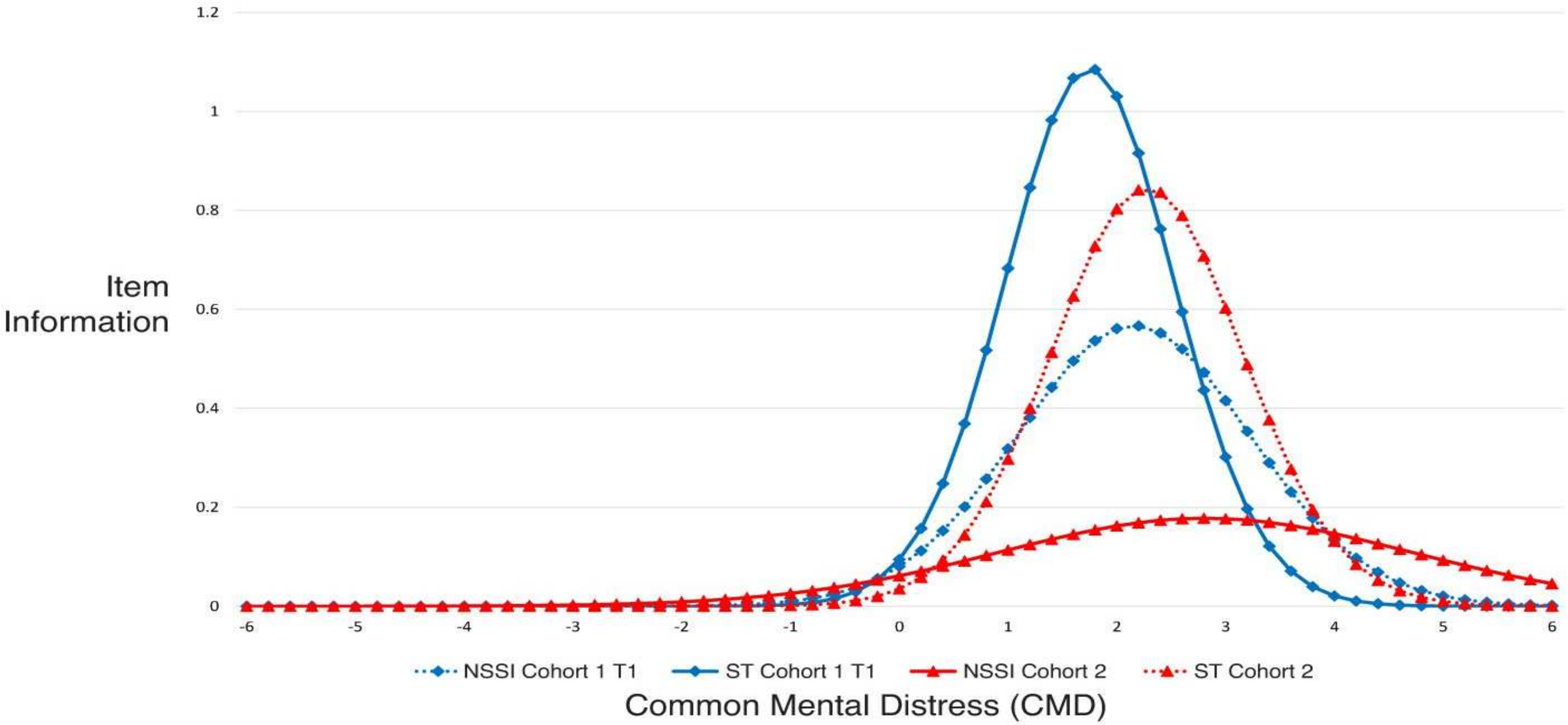

Supplementary Figure 1: Hierarchy of symptoms: the place of non-suicidal self-harm (NSSI) and suicidal though (ST) on the latent continuum of Common Mental Distress (in standard deviations) in Cohort 1_{T1} and Cohort 2.

Item Response Theory (IRT) analysis is concerned, broadly speaking, with investigating the relationship between items and the latent construct. Here we computed item response function showing how much information NSSI and ST (here treated as indicators of CMD) contribute to the latent variable – CMD. The above graph shows that NSSI and ST provided information in above-average to high ranges of CMD, with the peak of the information curves for NSSI occurring around +2 SD in both cohorts. The information curve for ST in Cohort 2 was flatter, suggesting less contribution to the latent CMD dimension than ST had in Cohort 1_{T1} dataset. This may be due to the differences in age structure and psychopathology status in both cohorts. Nonetheless, in both cohorts the peak in the ST curves occurred between +2 and +3 SD (high end of the CMD dimension), showing that ST lies on the more severe spectrum of CMD dimension than NSSI does.

Polek E, *et al. BMJ Open* 2020; 10:e032494. doi: 10.1136/bmjopen-2019-032494

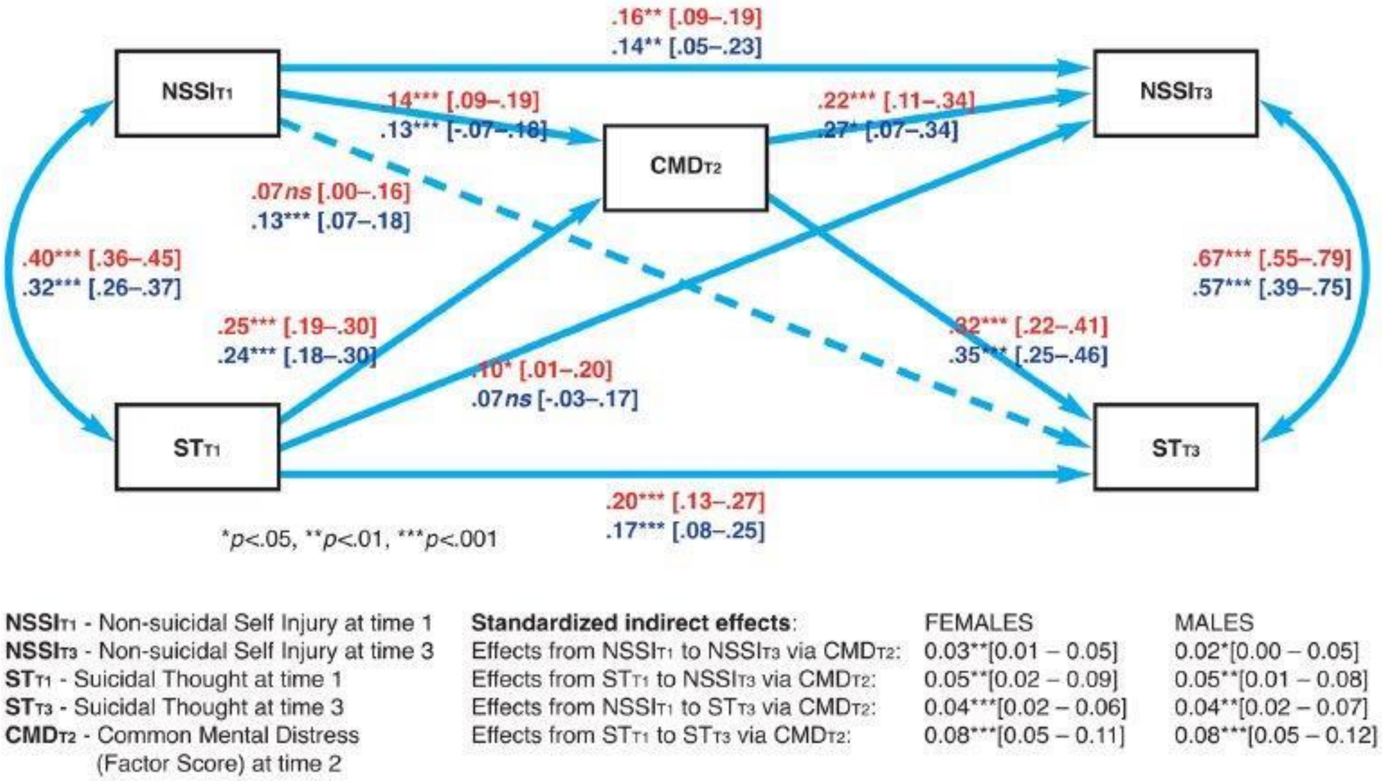

Supplementary Figure 2: Mediation effect of Common Mental Distress at time 2 (CMD_{T2}) moderated by sex (female n=1286 (red colour); male n=1115 (blue colour)) in the Cohort 1

Standardised pathway coefficients (with confidence intervals reported in squarer brackets) were obtained in multiple group pathway analysis in which sex was treated as a grouping variable. We tested the equivalence in pathway coefficients by means of comparing chi-square tests when the coefficient was "fixed" to be equal across sexes versus when it was free to vary across sexes[2]. We also tested the equivalence of fit indices of the model in both sexes. We found no evidence for differences in individual pathway coefficients or fit indices between sexes. This suggests that CMD at T2 mediated the longitudinal persistence of NSSI and ST in the same manner in females and males – no evidence of sex differences in the longitudinal mediation process was found. Additional details are reported in Supplementary Table 10.

Polek E, *et al. BMJ Open* 2020; 10:e032494. doi: 10.1136/bmjopen-2019-032494

Age and gender: Descriptive analysis

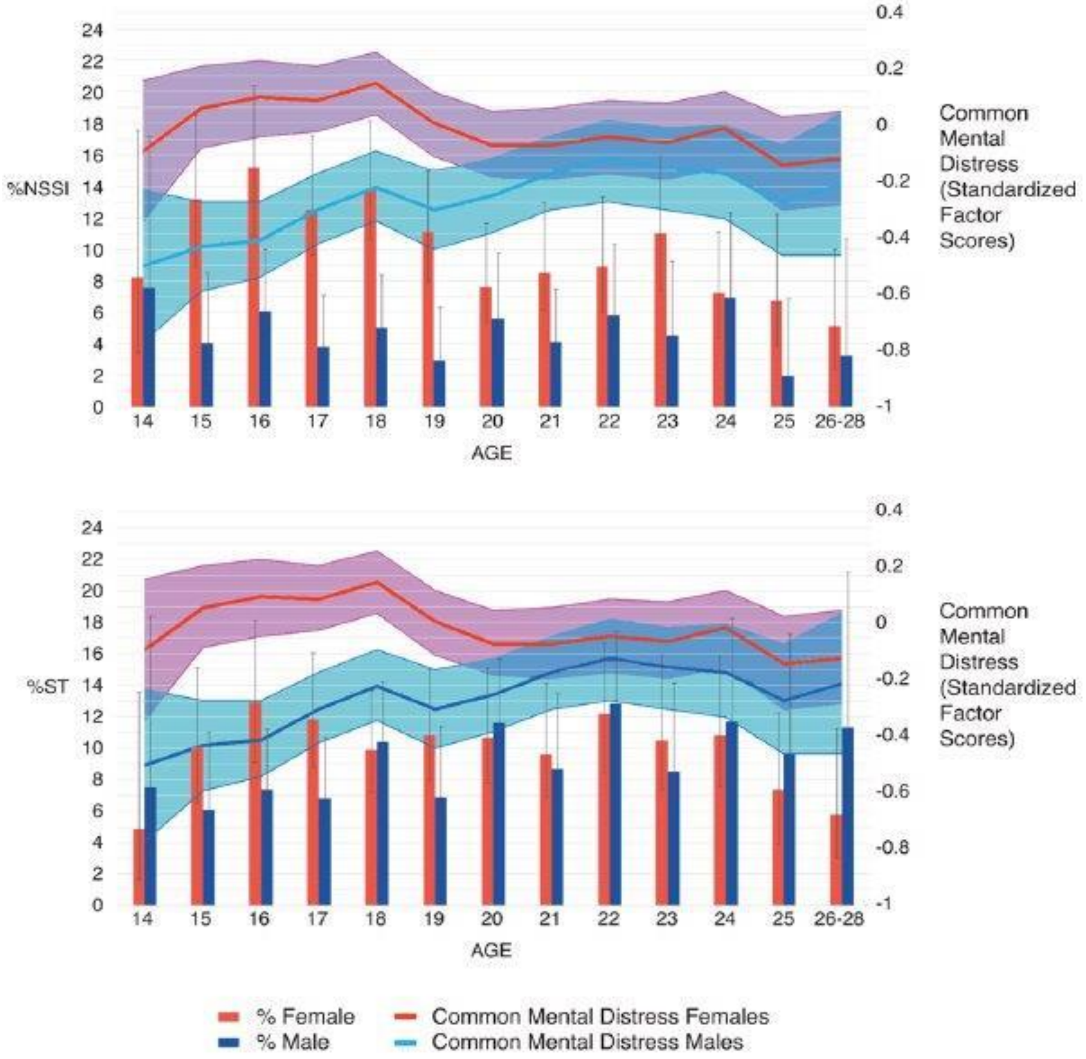

Supplementary Figure 3. Percentages of non-suicidal self-injury (NSSI), suicidal thoughts (ST) and levels of Common Mental Distress in age groups for both sexes in Cohort 1

To analyse the relationship between age, sex, NSSI, ST, and CMD descriptively, we grouped observations from all 3 time points in Cohort 1_{T1-T3} by age, rather than by data time point. This grouping allowed us to investigate levels of CMD, NSSI and ST in a broad age range of 14-28 years (note that this also entailed the inclusion of the same individuals from consecutive data sweeps (e.g., when an individual was 14, 15 and 16 years old) in the adjacent age groups). The histograms showing percentages of NSSI and ST with Wilson confidence intervals were plotted against the lines representing the means of CMD with confidence intervals for every age group for both sexes separately (Figure 3 above).

Polek E, *et al. BMJ Open* 2020; 10:e032494. doi: 10.1136/bmjopen-2019-032494

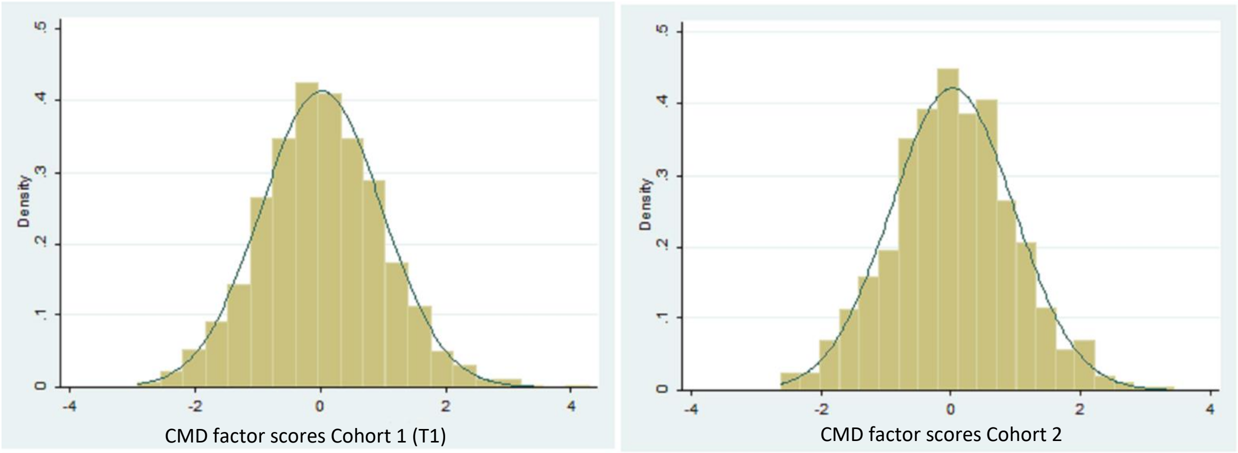

Supplementary Figure 4: Histograms of CMD factor scores in Cohort 1 (T1) and Cohort 2 with a schematic normal distribution line

Data collection tools:

Study data were collected and managed using REDCap electronic data capture tools[19] hosted at the University of Cambridge. REDCap (Research Electronic Data Capture) is a secure, web-based application designed to support data capture for research studies, providing 1) an intuitive interface for validated data entry; 2) audit trails for tracking data manipulation and export procedures; 3) automated export procedures for seamless data downloads to common statistical packages; and 4) procedures for importing data from external sources.

Group Information

NSPN (NeuroScience in Psychiatry Network: http://www.nspn.org.uk/) is a research consortium formed by the University of Cambridge and University College London, launched in November 2012 and supported by Wellcome Trust Award (095844/Z/11/Z). The group included the following members:

Principal investigators:

Edward Bullmore (CI from 01/01/2017)[1,2]

Ian Goodyer (CI until 01/01/2017)[1]

Peter Jones[1,2,3]

Raymond Dolan[4,5]

Peter Fonagy[6]

NSPN (funded) staff:

Michael Moutoussis[4,5]

Tobias Hauser[4,5]

Sharon Neufeld[1]

Petra Vértes[1,2]

Kirstie Whitaker[1,2]

Gita Prabhu[4,5]

Laura Villis[1]

Junaid Bhatti[1]

Becky Inkster[1]

Cinly Ooi[1]

Barry Widmer[1]

Ayesha Alrumaithi[1]

Sarah Birt[1]

Kalia Cleridou[5]

Hina Dadabhoy[5]

Sian Granville[5]

Elizabeth Harding[5]

Alexandra Hopkins[4,5]

Daniel Isaacs[5]

Janchai King[5]

Danae Kokorikou[5,6]

Harriet Mills[5]

Ciara O'Donnell[1]

Sara Pantaleone[5]

Aislinn Bowler[5]

Affiliated scientists:

Pasco Fearon[6]

Anne-Laura van Harmelen[1]

Rogier Kievit[4,7]

1 Department of Psychiatry, University of Cambridge, United Kingdom

2 NIHR Applied Research Collaboration East of England, UK

3 NIHR Cambridge Biomedical Research Centre, UK

4 Max Planck University College London Centre for Computational Psychiatry and Ageing Research, University College London, UK

5 Wellcome Centre for Human Neuroimaging, University College London, United Kingdom

6 Research Department of Clinical, Educational and Health Psychology, University College London,

United Kingdom

7 Medical Research Council Cognition and Brain Sciences Unit, University of Cambridge, United Kingdom

References:

1 Reise, S. P. The rediscovery of bifactor measurement models. *Multivariate Behavioural Research*. 2012;47:667-696, doi:10.1080/00273171.2012.715555.

2 Muthen, L. & Muthen, B. *Mplus Users's Guide*. (Muthen & Muthen, 1998-2002).

3 Reise, S. P., Moore, T. M. & Haviland, M. G. Bifactor models and rotations: Exploring the extent to which multidimensional data yield univocal scale scores. *Journal of Personality Assessment*. 2010;92:544-559, doi:10.1080/00223891.2010.496477.

4 Noble M, McLennan, D, Wilkinson K, Whitworth A, & Barne H. The English Indices of Deprivation 2007. London: Department for Communities and Local Government. (2008).

5 van Buuren, S. & Groothuis-Oudshoorn, K. MICE: Multivariate Imputation by Chained Equations in R. *Journal of Statistical Software.*2011;45,1-67.

6 Sterne, J. A. C. *et al.* Multiple imputation for missing data in epidemiological and clinical research: Potential and pitfalls. *British Medical Journal.* 2009;339:157-160.

7 Rubin, D. B. *Multiple imputation for nonresponse in surveys.* (Wiley, 1987).

8 Hammerton, G., Zammit, S., Potter, R., Thapar, A. & Collishaw, S. Validation of a composite of suicide items from the Mood and Feelings Questionnaire (MFQ) in offspring of recurrently depressed parents. . *Psychiatry Research.* 2014;216:82-88.

9 Wilkinson P.O., Qiu T., Neufeld S., Jones P.B. & Goodyer I.M. Sporadic and recurrent non-suicidal self-injury before age 14 and incident onset of psychiatric disorders by 17 years: prospective cohort study. *British Journal of Psychiatry.*2018;212:222-226, doi:10.1192/bjp.2017.45.

10 Cassels M. *et al.* Poor family functioning mediates the link between childhood adversity and adolescent non-suicidal self-injury. *Journal of Child Psychology and Psychiatry*. 2018;59(8):881-887. doi: 10.1111/jcpp.12866

11 Angold, A. *et al.* The development of a short questionnaire for use in epidemiological studies of depression in children and adolescents. *International Journal of Methods in Psychiatric Research.*1995;5:237-249.

12 Reynolds, C. R. Concurrent validity of what I think and feel: The revised children's manifest anxiety scale. *Journal of Consulting and Clinical Psychology.* 1980;48:774-775. doi:10.1037/0022-006x.48.6.774 (1980).

13 Bamber, D., Tamplin, A., Park, R. J., Kyte, Z. A. & Goodyer, I. M. Development of a short Leyton Obsessional Inventory For Children and Adolescents. *Journal of the American Academy of Child and Adolescent Psychiatry.*2002;41:1246-1252.

14 St Clair C. M. *et al.* Characterising the latent structure and organisation of self-reported thoughts, feelings and behaviours in adolescents and young adults. *PLOS One.* 2017;12:1-27, doi:https://doi.org/10.1371/journal.pone.0175381.

15 Rosenberg, M. *Society and the adolescent self-image*. (Princeton University Press, 1965).

16 Tennant, R. *et al.* The Warwick-Edinburgh mental well-being scale (WEMWBS): development and UK validation. *Health and Quality of Life Outcomes* **5**, doi:10.1186/1477-7525-5-63 (2007).

17 Raine, A. The SPQ: A scale for the assessment of schizotypal personality based on DSM-III-R criteria. *Schizophrenia Bulletin.*1991;17:555-564.

18 Shaffer, D., Fisher, P., Lucas, C. P., Dulcan, M. K. & Schwab-Stone, M. E. NIMH Diagnostic Interview Schedule for Children Version IV (NIMH DISC-IV): description, differences from previous versions, and reliability of some common diagnoses. *Journal of the American Academy of Child and Adolescent Psychiatry*. 2000;39:28-38.

19 Harris PA, Taylor R, Thielke R, Payne J, Gonzalez N, Conde JG. Research electronic data capture (REDCap) – A metadata-driven methodology and workflow process for providing translational research informatics support. *Journal of Biomedical Informatics.* 2009;42(2):377-81.

Polek E, *et al. BMJ Open* 2020; 10:e032494. doi: 10.1136/bmjopen-2019-032494
