# Supplementary material for: How do the prevalence and relative risk of non-suicidal self-injury and suicidal thoughts vary across the population distribution of common mental distress (the p factor)? Observational analyses replicated in two independent UK cohorts of young people
Source: BMJ Open. 2020 May 11;10(5):e032494. doi: 10.1136/bmjopen-2019-032494 (PMC7223145; doi:10.1136/bmjopen-2019-032494)
Supplement: Author's manuscript [file bmjopen-2019-032494.draft_revisions.pdf]

BMJ Open

BMJ Open is committed to open peer review. As part of this commitment we make the peer review history of every article we publish publicly available.

When an article is published we post the peer reviewers' comments and the authors' responses online. We also post the versions of the paper that were used during peer review. These are the versions that the peer review comments apply to.

The versions of the paper that follow are the versions that were submitted during the peer review process. They are not the versions of record or the final published versions. They should not be cited or distributed as the published version of this manuscript.

BMJ Open is an open access journal and the full, final, typeset and author-corrected version of record of the manuscript is available on our site with no access controls, subscription charges or pay-per-view fees (http://bmjopen.bmj.com).

If you have any questions on BMJ Open's open peer review process please email info.bmjopen@bmj.com

BMJ Open

Replicable associations between common mental distress and suicide risk in young people: implications for clinical practice and population suicide prevention

Journal:	*BMJ Open*
Manuscript ID	bmjopen-2019-032494
Article Type:	Research
Date Submitted by the Author:	27-Jun-2019
Complete List of Authors:	polek, ela; University College Dublin<br>Neufeld, Sharon A. S.; Univ Cambridge<br>Wilkinson, Paul; University of Cambridge, Cambridge Neuroscience<br>Goodyer, Ian; Cambridge University, Psychiatry<br>St Clair, Michelle<br>Prabhu, Gita<br>Dolan, Ray<br>Bullmore, Edward<br>Fonagy, Peter<br>Stochl, Jan; University of Cambridge, Department of Psychiatry; NIHR Collaboration for Leadership in Applied Health Research & Care (CLAHRC) East of England,<br>Jones, Peter; University of Cambridge, Department of Psychiatry
Keywords:	EPIDEMIOLOGY, Child & adolescent psychiatry < PSYCHIATRY, PUBLIC HEALTH

SCHOLARONE™
Manuscripts

I, the Submitting Author has the right to grant and does grant on behalf of all authors of the Work (as defined in the below author licence), an exclusive licence and/or a non-exclusive licence for contributions from authors who are: i) UK Crown employees; ii) where BMJ has agreed a CC-BY licence shall apply, and/or iii) in accordance with the terms applicable for US Federal Government officers or employees acting as part of their official duties; on a worldwide, perpetual, irrevocable, royalty-free basis to BMJ Publishing Group Ltd ("BMJ") its licensees and where the relevant Journal is co-owned by BMJ to the co-owners of the Journal, to publish the Work in this journal and any other BMJ products and to exploit all rights, as set out in our licence.

The Submitting Author accepts and understands that any supply made under these terms is made by BMJ to the Submitting Author unless you are acting as an employee on behalf of your employer or a postgraduate student of an affiliated institution which is paying any applicable article publishing charge ("APC") for Open Access articles. Where the Submitting Author wishes to make the Work available on an Open Access basis (and intends to pay the relevant APC), the terms of reuse of such Open Access shall be governed by a Creative Commons licence – details of these licences and which Creative Commons licence will apply to this Work are set out in our licence referred to above.

Other than as permitted in any relevant BMJ Author's Self Archiving Policies, I confirm this Work has not been accepted for publication elsewhere, is not being considered for publication elsewhere and does not duplicate material already published. I confirm all authors consent to publication of this Work and authorise the granting of this licence.

For peer review only

Replicable associations between common mental distress and suicide risk in young people: implications for clinical practice and population suicide prevention

Ela Polek[1,9], Sharon Neufeld[1], Paul Wikinson[1], Ian M. Goodyer[1], NSPN Consortium[1,2], Michelle C. St Clair[3], Gita Prabhu[2], Ray Dolan[2,7], Ed Bullmore[1,5], Peter Fonagy[4], Jan Stochl[1,5,8] & Peter B. Jones[1,5,6]

[1] Department of Psychiatry, University of Cambridge, UK

[2] Wellcome Centre for Human Neuroimaging, University College London, UK

[3] Department of Psychology, University of Bath, UK

[4] Division of Psychology and Language Sciences, University College London, UK

[5] NIHR Collaboration for Leadership in Applied Health Research & Care East of England, UK

[6] NIHR Cambridge Biomedical Research Centre, UK

[7] Max Planck UCL Centre for Computational Psychiatry and Ageing Research, UK

[8] Department of Kinanthropology, Charles University, Czech Republic

[9] School of Psychology, University College Dublin

Correspondence to:

Professor Peter B. Jones

Herchel Smith Building

Cambridge Biomedical Campus

CB2 0SZ

UK

Fax:01223 336581

e-mail: pbj21@cam.ac.uk

the total word count: 3594

For peer review only

For peer review only - http://bmjopen.bmj.com/site/about/guidelines.xhtml

Abstract:

Objectives: To inform suicide prevention policies and responses to youths at risk by investigating whether suicide risk is predicted by a summary measure of common mental distress (CMD) as well as by conventional psychopathological domains; to define the distribution of suicide risks over the population range of CMD; to test whether such distress mediates the medium-term persistence of suicide risks.

Design: Two independent samples of young people studied during three sweeps: the Neuroscience in Psychiatry (NSPN) 2400 cohort (n=2403) and the ROOTS cohort (n=1074); Cohorts 1 and 2, respectively.

Setting: Population-based in two UK centres.

Participants: Volunteers age 14-24 years recruited from primary health care registers, schools and colleges; advertisements to complete quotas in age-sex-strata.

Method: We analysed questionnaire data from Cohort 1 (sweeps 1-3) and Cohort 2 (sweep 3), collected between November 2012 – December 2016 and February 2008 – December 2009, respectively. We calculated a CMD score using confirmatory bifactor analysis; used logistic regressions to determine adjusted associations between risks and psychopathology (in continuous and above-the-norm categorical format); curve-fitting to examine the relative prevalence of ST and NSSI over the population distribution of CMD; and pathway mediation models to examine longitudinal associations.

Results: We found a dose-response relationship between levels of CMD and risk of suicide. The majority of all subjects experiencing ST and NSSI (Cohort 1 78% and 76%; Cohort 2 66% and 71%, respectively) had CMD scores no more than two standard deviations above the population mean; higher scores indicated the highest risk but were, by definition, infrequent. CMD mediated the longitudinal course of both suicide risks.

For peer review only - http://bmjopen.bmj.com/site/about/guidelines.xhtml

Conclusions. NSSI and ST in youths reflect common mental distress that also mediates their persistence. Universal prevention strategies reducing levels of CMD in the whole population without recourse to screening or measurement may prevent more suicides than approaches targeting youths with the most severe distress or with psychiatric disorders.

Article summary

Article focus

- Is *Common Mental Distress (CMD),* a latent dimension summarising various mental symptoms, useful in prevention policies focusing on the heightened risk of suicide?

Key messages

- The results argue for interventions and public health approaches to reduce suicide risk by lowering the population mean of common mental distress; focus on the few individuals with the highest levels of CMD misses the majority of individuals at risk.

Strengths and limitations of this study

- Replication of the findings in two independent cohorts strengthens confidence in the findings.
- The main limitation is related to sample attrition, which was the main bias in both cohorts.
- Multiple imputations mitigated possible biases related to attrition.

For peer review only - http://bmjopen.bmj.com/site/about/guidelines.xhtml

Introduction

Adolescence sees the onset of a range of psychopathology including suicidal thoughts (ST) and non-suicidal self-injury (NSSI)[1-3] that individually or together convey heightened risk of suicide attempts[4-6]. Non-suicidal and suicidal self-harm predict completed suicide[7], the second most common cause of deaths among 10 to 24 year-olds, worldwide[8]. Prediction and prevention in young people are priorities but NSSI (5-42% in community samples[9,10]) and ST (15-25% in community samples[11,12]) is common so it is difficult to predict who will ultimately make a serious attempt[13] or die by suicide. Indeed, the usefulness of clinical risk protocols relying on the identification of a psychiatric diagnosis is questionable[14,15]. The same problems affect public health suicide prevention programmes. A seminal study revealed a high prevalence of false-negatives in prospective identification of suicide[16]. Prevention policies that embrace the whole population might overcome these difficulties but lack theoretical or empirical foundations[1].

Suicidal thoughts and behaviours are routinely considered as markers of depression (e.g., in DSM-5) but by no means all young people dying by suicide have had a mood disorder[17]. NSSI increases the risk of suicide when occurring in combination with any internalising or externalising symptoms[18,19], or with any psychiatric diagnosis[20], particularly multiple diagnoses[21]. Thus, this risk might be better predicted by multiple symptoms rather than by the presence of a single disorder, such as depression.

Recent studies suggest that a broad range of symptoms conventionally seen as components of distinct disorders are better construed as manifestations of a single, latent dimension distributed within the general population. This dimension has been variously referred to as the p-factor[22], general psychopathology[23] or, as we prefer here, common mental distress (CMD)[24,25]. Parsimonious statistical models with dimensions that encompass low-prevalence phenomena such as psychotic experiences, fit empirical data better than models

For peer review only

with distinct disorders[22,26]. High co-morbidity of psychiatric diagnoses, shared causal factors and treatments, and trans-diagnostic psychological and neural correlates support the validity of a CMD concept[22-24,26-29]. Suicide risk is related to multiple symptoms or disorders (and thus to higher CMD scores), not the presence of one specific symptom or disorder, so it is important to understand the nature of dose-response relationships between CMD and suicide risks. This could guide a clinical response in the face of suicide risk[30] and also shape population-based suicide prevention.

In this study, we describe the presence of a CMD dimension in young people aged 14-26 years and the occurrence of ST and NSSI referred to collectively, hereafter, as a suicide risk. Drawing on a psychometric study[25], which demonstrated high theoretical validity and high measurement qualities of the CMD factor comprising measures of common mental illness (depression, anxiety, psychotic experiences, obsessions and compulsions) as well as traits and characteristics commonly considered to contribute to the general level of mental health (antisocial trait, well-being, self-esteem) we aimed to test here associations between CMD and suicide risk, and contrast CMD with specific psychopathological domains, exploring the utility of this summary measure.

Next, we aimed to answer the following questions:

1. What are the prevalence and relative risk of NSSI and ST across the distribution of CMD?
2. Does the CMD dimension mediate the medium-term persistence of NSSI and ST?

We used data from two population-based cohorts with complementary designs and very similar measures. To address the first question, we used cross-sectional data from Cohort 1, Time 1 (used as a discovery sample) and Cohort 2 (used as a stepwise replication sample); to

For peer review only - http://bmjopen.bmj.com/site/about/guidelines.xhtml

address the second question we used three longitudinal waves of Cohort 1 (see details in Method).

Method

Study Design and Participants

Cohort 1

Participants in the NSPN 2400 Cohort[31] were recruited largely via postal invitations sent through general practitioners and schools in Cambridgeshire and Greater London, UK. Data collection was carried out in two research centres: University College London and the University of Cambridge between November 2012 and December 2016. Purposive sampling obtained at least 200 males and 200 females from the community in 5 age groups: 14-15, 16-17, 18-19, 20-21, 22-24 years. Three data collections took place a year apart (T1-T3). At T1, 2403 individuals returned questionnaires (average age 18.9 years, SD=3.0; 54% females); at T2, 1815 returned questionnaires (76% response, average age 20.0 years, SD=3.1; 56% female), and 1245 at T3 (52% of baseline; average age 21.0 years, SD=3.1; 59% female).

Cohort 2

The ROOTS study[32] was used for replication of findings from Cohort 1. Two-stage sampling involved random selection of 27 schools in Cambridgeshire, UK. Eighteen schools agreed to participate; invitations were sent to 14-year-olds randomly selected from class registers and to their parents; 1238 students participated in the initial data collection (55% female) (and further 4 data collection waves took place). Note that in the current analysis we used only the data from the third data sweep collected between February 2008 and December 2009, when participants were of average age 17.5 years, *SD*=0.3 (N=1074, 56% female; 87% of baseline sample), the closest age to T1 of Cohort 1.

For peer review only - http://bmjopen.bmj.com/site/about/guidelines.xhtml

Both cohorts comprised predominantly white European (77% in Cohort 1 and 87% in Cohort 2) young people, consistent with the self-ascribed demographics of the two study populations. Written consent from participants age 14 or 15 years was supplemented by written consent from their parent or legal guardian; older participants gave their own written consent. Ethical approval was obtained for Cohort 1 from the National Health Service Research Ethics Service (# 97546) and for Cohort 2 from the Cambridgeshire 2 REC (# 03/302).

For peer review only

Measures

Sociodemographic information was collected using routine methods[31,33]. The Index of Multiple Deprivation (IMD), a summary measure of the socioeconomic status of participants' residential neighbourhood, is calculated from census information[34]. Questionnaires of mental illness and wellness are set out in Table 1 and items are listed in the Supplementary table 1. Scores in questionnaires were computed according to published manuals or validation studies (cited in Table 1), standardized to unify their measurement scales.

Table 1

Statistical analysis

Confirmatory bifactor analysis with a WLMSV estimator in Mplus 7.4 was used to compute factor scores for CMD in the three data sweeps of Cohort 1 and Cohort 2 based on the model validated elsewhere[25] (see CMD measures in Table 1 beneath; the list of used items and details of bifactor modelling can be found in the Supplementary table 1). Next, we addressed attrition in Cohort 1 by means of multiple imputations (see details in the Supplement).

To prove that NSSI and ST were predicted by multiple psychopathological domains and also by CMD (which represents a summary of those domains), we used Stata 12 to compute for Cohort 1_{T1} and Cohort 2 data sensitivity / specificity indicator – the area under the curve (AUC – reported in the Supplementary table 2) for NSSI and ST as criteria. We

For peer review only - http://bmjopen.bmj.com/site/about/guidelines.xhtml

computed a series of logistic regressions, estimating odds ratios (OR) with confidence intervals for each predictor (treated as categorical with the cut-off point above 1SD and then continuous), while we controlled for effects of age and sex (Figure 1).

To answer Question 1, distributions of CMD scores in both cohorts were plotted against lines representing percentages of subjects reporting NSSI and ST within bands of CMD expressed as standard deviations (upper panel of Figure 2) and against bar histograms representing NSSI and ST frequencies in both cohorts (lower panel of Figure 2). In addition, NSSI and ST information curves were computed to determine in what range of the CMD dimension these items are located (see Supplementary figure 1).

Using Cohort $1_{T1\text{-}T3}$ data to answer Question 2, we examined the longitudinal relationship between CMD, NSSI and ST (in particular the predictive role of CMD in persistence of NSSI and ST): we computed direct and mediation (via CMD_{T2}) effects of ST_{T1} and $NSSI_{T1}$ on $NSSI_{T3}$ and ST_{T3} in a pathway mediation model with confidence intervals in Mplus 7.4 (computing bias-corrected bootstrapping was not possible due to the use of multiply imputed datasets). We computed this model for the total sample (Figure 3) and then for both sexes separately (Supplementary figure 2) using the Multiple Group Method, so as to test a moderated-mediation model (with CMD_{T2} as a mediator, and sex as a moderator). Age was a control variable.

For peer review only

Results

Associations of NSSI and ST with demographic and psychopathological variables

In both cohorts NSSI and ST were unrelated to demographic variables, including sex and age (See Supplementary tables 3 and 4); CMD was negatively related to male gender (Supplementary table 5). When examined descriptively over the pooled age groups, the prevalence of NSSI and ST mirrored the CMD levels (see Supplementary figure 3). CMD

For peer review only - http://bmjopen.bmj.com/site/about/guidelines.xhtml

and all conventional psychopathological predictors of NSSI and ST had statistically significant and similar size ORs in logistic regression models (see Figure 1 and Supplementary table 2).

Figure 1

Prevalence of NSSI and ST in the two cohorts

In Cohort 1 (N=2403) there was no statistically significant change in the prevalence of NSSI (within the last month) over the three time points: in the imputed data 9.3% (n=223) reported $NSSI_{T1}$, 8.3% (n=199) $NSSI_{T2}$ and 8.2% (n=197) $NSSI_{T3}$. Similarly, there was no statistically significant change in prevalence of ST (within the last two weeks) over the three time points: 10.1% (n=243) ST_{T1}, 11.4% (n=274) ST_{T2} and 11.7% (n=281) ST_{T3} (see Supplementary tables 6 and 7).

In Cohort 2 (N=1074), 11.7% (n=126) reported lifetime NSSI and 5.4% (n=58) reported ST within the two last weeks. Accuracy and precision of these prevalence estimates were affected by attrition (see *Discussion: limitations*). Attrition in Cohort 1 at T2 and T3 was only marginally related to demographic and exposure variables at T1 (Spearman's rho 0.05-0.12), but unrelated to the outcome – NSSI and ST (see Supplementary table 8).

Question 1: Associations of NSSI and ST with CMD

Next, we focused on absolute risk and the numbers of NSSI and ST events generated by these risk functions. The dose-response curves in the upper panel of Figure 3 show that relative risks of NSSI and ST increased markedly with increasing severity of CMD, the highest risks being in those with very high scores beyond two standard deviations above the mean. On the other hand, most participants from both cohorts who reported NSSI or ST had mild (one SD above the mean) to moderate (two SD above the mean) CMD scores (lower panel of Figure 3). CMD was normally distributed so these scores were much more common; only a minority

For peer review only - http://bmjopen.bmj.com/site/about/guidelines.xhtml

of the total reports came from the few participants with very high CMD (>2 standard deviations above mean CMD). Thus, the majority of subjects experiencing ST or NSSI (Cohort 1: 78% and 76%; Cohort 2: 66% and 71%, respectively) had CMD scores within two standard deviations above the population mean. Very high CMD scores indicated the highest suicide risk but were rare, so generated the minority of events.

Figure 2

Question 2: Mediating effect of CMD on suicide risks in Cohort 1 over time

Cohort 1 CMD_{T2} contributed to the longitudinal persistence of NSSI and ST (i.e. $NSSI_{T1}$ predicted $NSSI_{T3}$ directly, and via mediation through CMD_{T2}; it also completely mediated the longitudinal effect of $NSSI_{T1}$ on ST_{T3}). Moreover, CMD_{T2} contributed to the longitudinal persistence of ST (i.e. ST_{T1} predicted ST_{T3} directly, as well as via mediating variable - CMD_{T2}). Overall, CMD_{T2} was a stronger predictor of $NSSI_{T3}$ and ST_{T3} than the antecedent variables measured at T1 (see Figure 3). There were no significant sex differences in direct and mediation pathways, showing that the mediation effects of CMD_{T2} were not moderated by sex (Supplementary figure 2). Age_{T1} was not a significant predictor of any variable in the model; the results when age was controlled for were very similar to those without controlling for age (differences in coefficients were in the second decimal place digits).

Figure 3

Discussion

In the present study, depressive phenomena were by no means the only psychopathological domain associated with increased risk of non-suicidal self-injury (NSSI) and suicidal thoughts (ST). Thus, the common mental distress factor with a normal population distribution appeared as a parsimonious and efficient summary that was, itself, a key predictor of suicide risk in both cohorts. NSSI and ST were not confined to participants scoring in the very high, quasi-clinical range for CMD. Around half of all participants expressing NSSI or ST came

For peer review only - http://bmjopen.bmj.com/site/about/guidelines.xhtml

from those scoring up to one standard deviation above mean CMD in a dose-response manner. The majority expressing these phenomena (two thirds to three quarters) scored within 2SD above the mean (Figure 2).

Regarding medium-term determinants of persistent NSSI and ST we showed (Figure 3) that CMD_{T2} mediated the persistence of NSSI and ST over two years, independent of gender and age. This mediation operates in two stages: first, ST and NSSI persist because these behaviours are markers for worsening CMD in the general population. This extends findings in adolescents with depressive disorder, where suicidal thoughts are a predictor of poor outcome[35]. Second, this greater CMD, itself, increases the risk for further suicidal thoughts and behaviours.

Strengths

Both cohorts were designed on epidemiological principles to capture behavioural and psychological variation in the population during the post-pubertal epoch during which risk for psychopathology accelerates. Replication of the findings in these independent cohorts strengthens confidence in the findings, as does internal consistency between cross-sectional associations found in both cohorts, and longitudinal associations found in Cohort 1.

Limitations

Sample attrition was the main bias in both cohorts. Each retained more young women than men; we found marginally higher attrition among lower socio-economic class, participants of non-white ethnicity and those with higher CMD (Supplementary table 8). Cohort 1 is robustly representative of the England and Wales population[31], whereas Cohort 2 under-represents participants with lowest socioeconomic status[32]. However, we have no reason to suppose that attrition biased our results, as it was unrelated to NSSI and ST (Supplementary table 8). If

For peer review only - http://bmjopen.bmj.com/site/about/guidelines.xhtml

there was a bias, it probably limits power rather than skewing an effect and is mitigated by replication between the cohorts. We used multiple imputation to minimise this bias.

There was only modest reliability of our obsessionality measure and a skewed measure of conduct problems in Cohort 1. A completely comprehensive range of psychopathological (and behavioural) items was unavailable; we did not have measures of unstable or abnormally elevated mood, addictions, eating disorders or hyperactivity. Thus, our measurement of CMD focused primarily on internalising rather than externalising symptoms. We broadened our scope far beyond depression, usually the focus of psychological disturbance in suicidality research, but future studies could include a broader range of measures and extend the investigation into clinical populations to improve measurement precision at the highest levels of CMD. Finally, we could not account for the effects of clustered design in the modelling, due to unavailability of the information about clustering of participants in both cohorts.

Implications & Conclusions

Our findings provide yet more evidence that a latent mental distress factor, conceptually akin to the p-factor, is a useful summary measure of psychopathology in the general population[24], diagnostic[22], and clinical[23] samples. We speculate that psychopathology is generated in a probabilistic manner rather than in diagnostic clusters, with common phenomena concerning depression and anxiety much more likely to occur prior to rarer phenomena such as NSSI, ST or psychotic experiences. Less frequent phenomena begin to co-occur as the severity of psychological disorder (or CMD) increases, in terms of more mental and behavioural phenomena or symptoms. This begins to yield clusters linked by common items that current diagnostic systems tend to ignore. This is consistent with the co-occurrence of suicidal risk and psychotic experiences seen in other[36-38] studies of young people, and with the present

For peer review only - http://bmjopen.bmj.com/site/about/guidelines.xhtml

IRT analysis showing that NSSI and ST are measuring the higher end of CMD (Supplementary figure 1). The approach we have followed illustrates the value of moving away from categorical classification and embracing an empirically-rooted, dimensional, hierarchical taxonomy in psychopathology research[39]. Such hierarchical approaches to phenomenological classification had been put forward before[40] or shortly after[41] the publication of DSM-3 and its successor classifications. Hierarchical models merit renewed interest[42], as they may resolve problems of comorbidity[26] as well as overlapping causes and biological mechanisms for suicide risk and other phenomena[43,44].

Our findings also have major implications for intervention and prevention of suicidal thoughts and behaviours. Clinically, the results suggest that NSSI and ST should never be dismissed or downplayed when they occur in young people without clear evidence of psychiatric disorder, a logical fallacy because NSSI and ST are *themselves* indicators of higher distress on a CMD factor. NSSI and ST will usually, but not always occur with other, more common psychopathology and their co-occurrence is a strong risk factor for suicide attempts[6]. Thus, NSSI and ST merit a swift professional response regardless of whether or not they occur with other symptoms that take individuals beyond conventional clinical thresholds and trigger traditional clinical risk protocols. Our findings help explain why research focused on high-risk subjects has yet to translate into useful clinical prediction tools[14,15,45].

From a public health and prevention perspective, the fact that rates of NSSI and ST begin to accelerate at levels of CMD well within a normal or non-clinical range argues strongly for universal interventions overtly aimed at lowering the population mean CMD and shifting the curve to the left. This should be alongside targeted approaches and effective clinical services[46]. Strategies concentrated on clinical populations, those with evidence of a psychiatric disorder or other individual markers will miss the majority of individuals

For peer review only - http://bmjopen.bmj.com/site/about/guidelines.xhtml

experiencing ST or engaging in NSSI because there are so few compared with those at lower risk: the *prevention paradox*[30].

Defining putative universal interventions to shift the population distribution of CMD will require careful research that can draw from other areas of medicine such as cardiovascular disease and stroke[30]. Elements have been widely scoped in the USA[15] and elsewhere, but not for constructs of population health and wellbeing such as CMD. Many involve decreasing common triggers[15] or improving young people's abilities to cope with stressors[47]. Delivery systems might include digital platforms that are virtually ubiquitous amongst young people, while schools and colleges are increasingly recognised as contexts for the delivery of such universal interventions[48]. However, the burgeoning importance of social media providing a broad-based and uniquely tailored environment for youth must be considered in suicide prevention strategies as both a toxic and a potential therapeutic milieu.

For peer review only - http://bmjopen.bmj.com/site/about/guidelines.xhtml

Conflict of Interest Disclosures

E.P., S.N., I.M.G., and J.S. have no competing interests. E.B., P.F., and P.B.J. are in receipt of National Institute for Health Research (NIHR) Senior Investigator Awards (NF-SI-0514-10157, and NF-SI-0514-10117. P.F. was in part supported by the NIHR Collaboration for Leadership in Applied Health Research and Care (CLAHRC) North Thames at Barts Health NHS Trust. P.W. has recent/current grant support from NIHR, Cambridgeshire County Council and CLAHRC East of England. P.W. discloses consulting for Lundbeck and Takeda; P.B.J. discloses consulting for Janssen and Ricordati. E.B. is employed half-time by the University of Cambridge and half-time by GlaxoSmithKline in which he holds stock.

Access to the data

E.P. had full access to all the data in the study and takes responsibility for the integrity of the data and the accuracy of the data analysis. The data are deposited in the University of Cambridge Data Repository, with the placeholder DOI https://doi.org/10.17863/CAM.25331 available to researchers via openNSPN@medschl.cam.ac.uk.

Funding/Support and Acknowledgments

The ROOTS study was supported by a Wellcome Trust Grant (Grant number 074296) to I.M.G. and P.B.J., the NIHR Collaborations for Leadership in Applied Research and Care (CLAHRC) East of England, and the NIHR Cambridge Biomedical Research Centre. The NSPN study was supported by the Wellcome Trust Strategic Award (095844/Z/11/Z) to I.M.G., E.B., P.B.J., R.D., P.F. The work has been carried out in the Department of Psychiatry, University of Cambridge. We wish to thank the NSPN and ROOTS participants and Dr Golam Khandaker for his comments.

Role of the Funder/Sponsor

The funding sources had no role in the design and conduct of the study; collection, management, analysis, and interpretation of the data; preparation, review, or approval of the manuscript; and decision to submit the manuscript for publication.

NSPN Consortium Information –see the Supplement

Patient and public involvement: No patient involved

For peer review only - http://bmjopen.bmj.com/site/about/guidelines.xhtml

Table 1. Measures used in both cohorts

Variables	*Measures*	Cohorts	
**Outcome variables:**		NSPN$_{T1-T3}$ (1)	ROOTS$_{age\ 17}$ (2)
Suicidal thoughts (ST)	One item from the MFQ[49]: I thought about killing myself. Responses were recoded into a binary format: no ST (original response option *Never)* and ST (original response options *Sometimes* or *Mostly* or *Always*).	×	×
Non-suicidal self-injury (NSSI)	One question from the Drug, Alcohol and Self-Injury (DASI)[25] questionnaire asking about engaging in self-injury without suicidal intent during the last month. Responses were recoded into a binary format indicating the occurrence of NSSI or lack of thereof.	×	
	One question asking about the occurrence of lifetime NSSI		×
**Predictors:**			
Conduct problems	11-item Antisocial Behaviour Questionnaire[25]	×	×
Anxiety	28-item Revised Children's Manifest Anxiety Scale[50]	×	×
Depression	29 items from the 33-item MFQ[49] (all items except for 4 items measuring suicidality)		
Obsessions and compulsions	11-item Revised Leyton Obsessional Inventory[51]	×	×
Psychotic-like experiences	11 items selected from the 74-item Schizotypal Personality Questionnaire (SPQ)[52]	×	
	11 items from the 20-item semi-structured interview from the Diagnostic Interview Schedule for Children-IV[53]		×
Self-esteem	10-item Rosenberg Self-Esteem Questionnaire (*)[54]	×	×
Well-being	14-item Warwick-Edinburgh Mental Well-Being Scale(*)[55]	×	×
Impulsivity	15 items from the 30-item Barratt Impulsiveness Scale[56] selected based on exploratory factor analysis - loadings above .25	×	
Antisocial traits	Total score from the 17-item Antisocial Process Screening Device (APSD)[57]	×	
Schizotypal traits	Total score from the 74-item Schizotypal Personality Questionnaire (SPQ) [52]	×	×

*scales were reversely scored, thus higher scores indicated lower self-esteem and well-being; for all other measures higher score indicates more psychopathology

For peer review only

For peer review only - http://bmjopen.bmj.com/site/about/guidelines.xhtml

Figures' legends:

Figure 1: Odds ratio in logistic regressions for suicidal thoughts (ST) and non-suicidal self-harm (NSSI) as outcomes predicted by psychopathological predictors (listed on the left) here treated as continuous variables; regressions were computed separately for each predictor and effects of age and sex were controlled in each regression for in both cohorts (see Supplementary Table 2).

Figure 2: Upper panel shows the dose-response effect of Common Mental Distress on non-suicidal self-harm (NSSI) and suicidal thought (ST) in Cohort 1 and Cohort 2. The lower panel shows the proportion of total reports in non-suicidal self-injury (NSSI) and suicidal thought (ST) broken down by standard deviations of Common Mental Distress; these add up to 100% from left to right. The normal population distribution of CMD, which was strikingly similar, but not identical, in Cohort 1 and 2, is shown by the purple line (see density plots in Supplement, Figure 1).

Figure 3: Mediation effect of Common Mental Distress at time 2 in Cohort 2: Standardised pathway coefficients with confidence intervals in square brackets.

For peer review only

For peer review only - http://bmjopen.bmj.com/site/about/guidelines.xhtml

References:

1. Hawton K, Saunders EA, O'Connor R. Self-harm and suicide in adolescents. *Lancet.* 2012;379:2373-2382.
2. Kidger J, Heron J, Lewis G, Evans J, Gunnell D. Adolescent self-harm and suicidal thoughts in the ALSPAC cohort: A self-report survey in England. *BMC Psychiatry.* 2012;12:1-12.
3. Nock MK. Future directions for the study of suicide and self-injury. J Clin Child Adolesc Psychol. 2012;41:255-259.
4. Scott LN, Pilkonis PA, Hipwell AE, Keenan K, Stepp SD. Non-suicidal self-injury and suicidal ideation as predictors of suicide attempts in adolescent girls: A multi-wave prospective study. *Compr Psychiatry.* 2015;58:1-10.
5. Ribeiro JD, Franklin JC, Fox KR, et al. Self-injurious thoughts and behaviors as risk factors for future suicide ideation, attempts, and death: A meta-analysis of longitudinal studies. *Psychol Med.* 2016;46:225-236.
6. Victor SE, Klonsky ED. Correlates of suicide attempts among self-injurers: A meta-analysis. *Clin Psychol Rev.* 2014;34(4):282-297.
7. Cooper J, Kapur N, Webb R, et al. Suicide after deliberate self-harm: a 4-year cohort study. *Am J Psychiatry.* 2005;162:297-303.
8. Patton GC, Coffey C, Sawyer SM, et al. Global patterns of mortality in young people: a systematic analysis of population health data. *Lancet*. 2009;374:881-892.
9. Muehlenkamp JJ, Claes L, Havertape L, Plener PL. International prevalence of adolescent non-suicidal self-injury and deliberate self-harm. *Child Adolesc Psychiatry Ment Health.* 2012;6(10).
10. Brunner R, Parzer P, Haffner J, et al. Prevalence and psychological correlates of occasional and repetitive deliberate self-harm in adolescents. *Arch Pediatr Adolesc Med.* 2007;161(7):641-649.
11. Bridge JA, Goldstein TR, Brent DA. Adolescent suicide and suicidal behavior. *J Child Psychol Psychiatry*. 2006;47:372-394.
12. Evans E, Hawton K, Rodham K, Deeks J. The prevalence of suicidal phenomena in adolescents: A systematic review of population-based study. *Suicide Life Threat Behav.* 2005;35(3):239-50.
13. Nielssen O, Wallace D, Large M. Pokorny's complaint: the insoluble problem of the overwhelming number of false positives generated by suicide risk assessment. *BJPsych Bulletin.* 2017;41:18-20.
14. Quinlivan L, Cooper J, Davies L, et al. Which are the most useful scales for predicting repeat self-harm? A systematic review evaluating risk scales using measures of diagnostic accuracy. *BMJ Open.* 2016;6(2): https://bmjopen.bmj.com/content/6/2/e009297.
15. Quinlivan L, Jayne Cooper J, Meehan D, et al. Predictive accuracy of risk scales following self-harm: multicentre, prospective cohort study. *Br J Psychiatry.* 2017;210:429-436.
16. Pokorny AD. Prediction of suicide in psychiatric patients. Report of a prospective study. *Arch Gen Psychiatry.* 1983;40:249-257.
17. Shaffer D, Gould MS, Fisher P, et al. Psychiatric diagnosis in child and adolescent suicide. *Arch Gen Psychiatry.* 1996;53:339-348.
18. Berona J, Horwitz AG, Czyz EK, King CA. Psychopathology profiles of acutely suicidal adolescents: Associations with post-discharge suicide attempts and rehospitalisation. *J Affect Disord*. 2017;209:97-104.
19. Nock MK, Joiner TE, Gordon KH, Lloyd-Richardson E, Prinstein MJ. Non-suicidal self-injury among adolescents: diagnostic correlates and relation to suicide attempts. *Psychiatry Res*. 2006;144(1):65-72.
20. Beckman K, Mittendorfer-Rutz E, Lichtenstein P, et al. Mental illness and suicide after self-harm among young adults: long-term follow-up of self-harm patients, admitted to hospital care, in a national cohort. *Psychol Med.* 2016;46:3397-3405.

For peer review only - http://bmjopen.bmj.com/site/about/guidelines.xhtml

21. Windfuhr K, While D, Kapur N, et al. Suicide risk linked with clinical consultation frequency, psychiatric diagnoses and psychotropic medication prescribing in a national study of primary-care patients. *Psychol Med.* 2016;46:3407-3417.
22. Caspi A, Houts RM, Belsky DW, et al. The p factor: One General Psychopathology Factor in the structure of psychiatric disorders? *Clin Psychol Sci.* 2014;2:119-137.
23. Patalay P, Fonagy P, Deighton J, et al. A general psychopathology factor in early adolescence. *The Br J Psychiatry.* 2015;207:15-22.
24. Stochl J, Khandaker GM, Lewis G, et al. Mood, anxiety and psychotic phenomena measure a common psychopathological factor. *Psychol Med.* 2015;45:1483-1493.
25. St Clair CM, Neufeld S, Jones BP, et al. Characterising the latent structure and organisation of self-reported thoughts, feelings and behaviours in adolescents and young adults. *PLOS* One. 2017; 12(4), 1-27. doi: https://doi.org/10.1371/journal.pone.0175381
26. Krueger RF. The structure of common mental disorders. *Arch Gen Psychiatry.* 1999;56:921-926.
27. Barch DM. The Neural Correlates of Transdiagnostic Dimensions of Psychopathology. *Am J Psychiatry*. 2017;174:613-615.
28. McTeague LM, Huemer J, Carreon DM, et al. Identification of Common Neural Circuit Disruptions in Cognitive Control Across Psychiatric Disorders. *Am J Psychiatry*. 2017;174:676-685.
29. Sharma A, Wolf DH, Ciric R, et al. Common Dimensional Reward Deficits Across Mood and Psychotic Disorders: A Connectome-Wide Association Study. *Am J Psychiatry.* 2017;174:657-666.
30. Rose G, Khaw M, Marmot G, Kay-Tee K, Marmot M. Chapter 3: The relation of risk to exposure. In: *Rose's strategy of preventive medicine (New ed.)* Oxford, Oxford University Press: 2008.
31. Kiddle B, Inkster B, Prabhu G, et al. The NSPN 2400 Cohort: a developmental sample supporting the Wellcome Trust NeuroScience in Psychiatry Network. *Int J Epidemiol.* 2018;47(1):18-19g.
32. Goodyer IM, Croudace T, Dunn V, Herbert J, Jones BP. Cohort Profile: Risk patterns and processes for psychopathology emerging during adolescence: the ROOTS project. *Int J Epidemiol.* 2010;39:361-369.
33. Office for National Statistics. Ethnicity and National Identity in England and Wales. 2012. Retrieved on 6th Feb 2018 from: https://www.ons.gov.uk/peoplepopulationandcommunity/culturalidentity/ethnicity/articles/ethnicityandnationalidentityinenglandandwales/2012-12-11#ethnicity-in-england-and-wales.
34. Noble M, McLennan, D, Wilkinson K, Whitworth A, & Barne H. The English Indices of Deprivation 2007. London: Department for Communities and Local Government. (2008).
35. King RA. Adolescent suicidal thoughts/behaviors are a marker of long-term vulnerability to poor adult outcomes. *J Am Acad Child Adolesc Psychiatry.* 2017;56:920-921.
36. Kelleher I, Keeley H, Corcoran P, et al. Clinicopathological significance of psychotic experiences in non-psychotic young people: evidence from four population-based studies. *Br J Psychiatry*. 2012;201:26-32.
37. Honings S, Drukker M, Groen R, van Os J. Psychotic experiences and risk of self-injurious behaviour in the general population: a systematic review and meta-analysis. *Psychol Med.* 2016;46:237-251.
38. Kelleher I, Corcoran P, Keeley H, et al. Symptoms and population risk for suicide attempt: A prospective cohort study. *JAMA Psychiatry.* 2013;70:940-948.
39. Lahey BB, Krueger RF, Rathouz PJ, Waldman ID, Zald DH. A hierarchical causal taxonomy of psychopathology across the life span. *Psychol Bull.* 2017;143:142-186.
40. Foulds GA, Bedford A. Hierarchy of classes of personal illness. *Psychol Med.* 1975;5:181-192.
41. Sturt E. Hierarchical patterns in the distribution of psychiatric symptoms. *Psychol Med.* 1981;11:783-792.

For peer review only - http://bmjopen.bmj.com/site/about/guidelines.xhtml

42. Kotov R, Krueger RF, Watson D. A paradigm shift in psychiatric classification: the Hierarchical Taxonomy Of Psychopathology (HiTOP). *World Psychiatry.* 2018;17:24-25.
43. Zald DH, Lahey BB. Implications of the Hierarchical Structure of Psychopathology for Psychiatric Neuroimaging. *Biol Psychiatry Cogn Neurosci Neuroimaging.* 2017;2:310-317.
44. Carver CS, Johnson SL, Timpano KR. Toward a functional view of the p factor in psychopathology. *Clinical Psychological Scienc.* 2017;5(5):880-889.
45. Franklin JC, Ribeiro JD, Fox KR, et al. Risk factors for suicidal thoughts and behaviors: A meta-analysis of 50 years of research. *Psychol Bull.* 2017;143(2):187-232.
46. Lewis G, Hawton K, Jones PB. Strategies for preventing suicide. *British Joournal of Psychiatry.* 1997;171:351-354.
47. Galante J, Dufour G, Benton A. et al. Protocol for the Mindful Student Study: A randomised controlled trial of the provision of a mindfulness intervention to support university students' wellbeing and resilience to stress. *BMJ Open*. 2016;6: https://bmjopen.bmj.com/content/6/11/e012300
48. Wasserman D, Hoven CW, Wasserman C, et al. School-based suicide prevention programmes: the SEYLE cluster-randomised, controlled trial. *Lancet*. 2015;385:1536-1544.
49. Hammerton G, Zammit S, Potter R, Thapar A, Collishaw S. Validation of a composite of suicide items from the Mood and Feelings Questionnaire (MFQ) in offspring of recurrently depressed parents. *Psychiatry Res.* 2014;216:82-88.
50. Reynolds CR. Concurrent validity of what I think and feel: The revised children's manifest anxiety scale. J Consult Clin Psychol. 1980;48:774-775.
51. Bamber, D., Tamplin, A., Park, R.J., Kyte, Z.A. & Goodyer, I.M. Development of a short Leyton Obsessional Inventory For Children and Adolescents. *Journal of the American Academy of Child and Adolescent Psychiatry* **41**, 1246-1252 (2002).
52. Raine A. The SPQ: A scale for the assessment of schizotypal personality based on DSM-III-R criteria. *Schizophr Bull.* 1991;17:555-564.
53. Shaffer D, Fisher P, Lucas CP, Dulcan MK, Schwab-Stone ME. NIMH Diagnostic Interview Schedule for Children Version IV (NIMH DISC-IV): description, differences from previous versions, and reliability of some common diagnoses. *J Am Acad Child Adolesc Psychiatry.* 2000;39:28-38.
54. Rosenberg M. *Society and the adolescent self-image*. Princeton, NJ: Princeton University Press; 1965.
55. Tennant R, Hiller L, Fishwick R, et al. The Warwick-Edinburgh mental well-being scale (WEMWBS): development and UK validation. Health Qual Life Outcomes. 2007;5:63.
56. Patton JH, Stanford MS, Barratt ES. Factor structure of the Barratt Impulsiveness Scale. *J Soc Clin Psychol.* 1995;51:768-774.
57. Poythress NG, Poythress NG, Douglas KS, Falkenbach D, et al. Internal consistency reliability of the self-report antisocial process screening device. *Asmnt.* 2006;13:107-113.

For peer review only - http://bmjopen.bmj.com/site/about/guidelines.xhtml

Authors contributions:

E.P. – conceptualised the study, computed statistical analyses and drafted the manuscript

P.B.J. – provided senior supervision, conceptualised the study, advised on statistical analyses, read and critically apprised the manuscript, re-drafted and edited the manuscript

J.S. – provided statistical advice, replicated multiple imputations, provided data from multiple imputations, read and critically apprised the manuscript

S.N. – advised on handling missing data, replicated multiple imputations, read and critically apprised the manuscript

P.W. – read and critically apprised the manuscript, provided key referred articles

R.D. – read and critically apprised the manuscript, provided key referred articles

I.M.G. – read and critically apprised the manuscript, provided key referred articles

E.B. – read and critically apprised the manuscript, provided key referred articles

P.F. – read and critically apprised the manuscript, provided key referred articles

R.D. – read and critically apprised the manuscript

M.C.S.C. – contributed to data collection and project management, provided advise on bifactor modelling

G.P. – contributed to data collection and project management

For peer review only

For peer review only - http://bmjopen.bmj.com/site/about/guidelines.xhtml

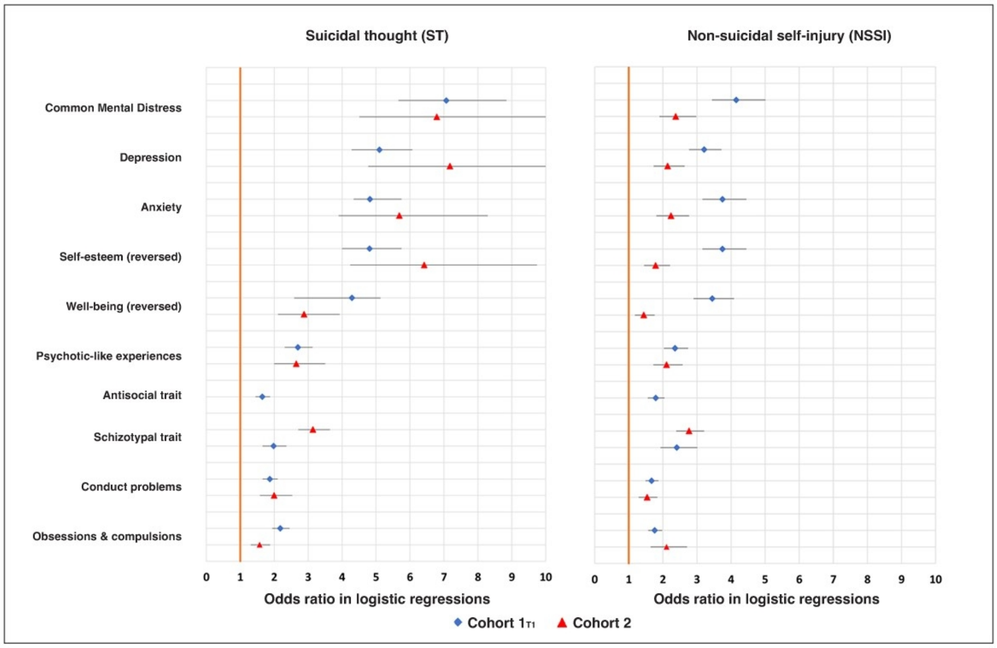

123x80mm (240 x 240 DPI)

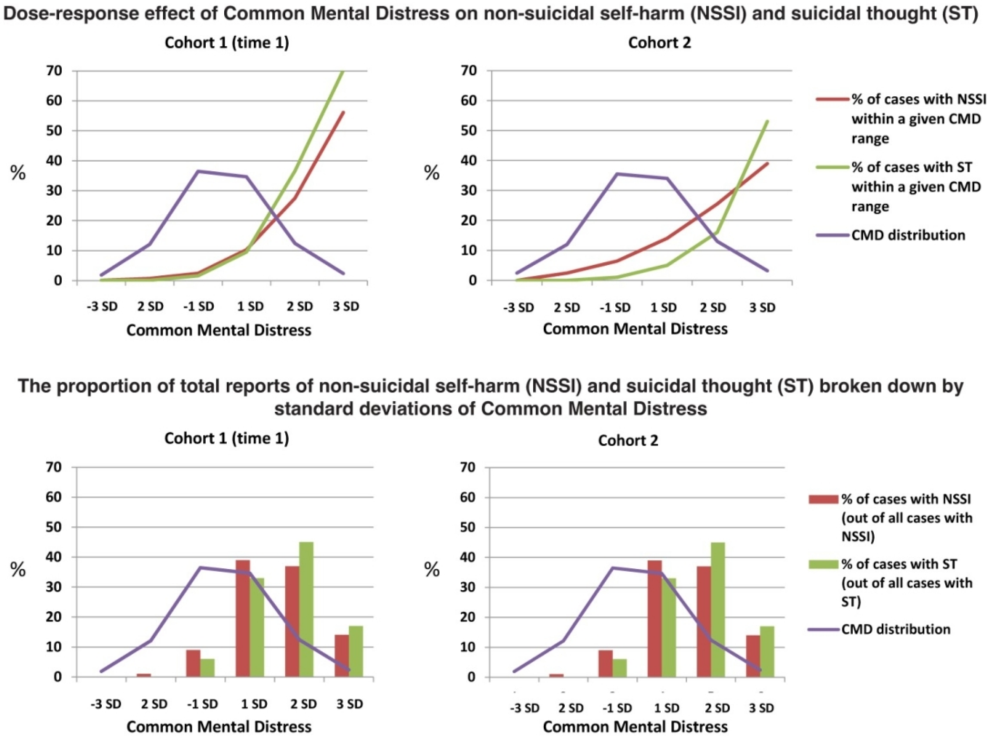

175x132mm (220 x 220 DPI)

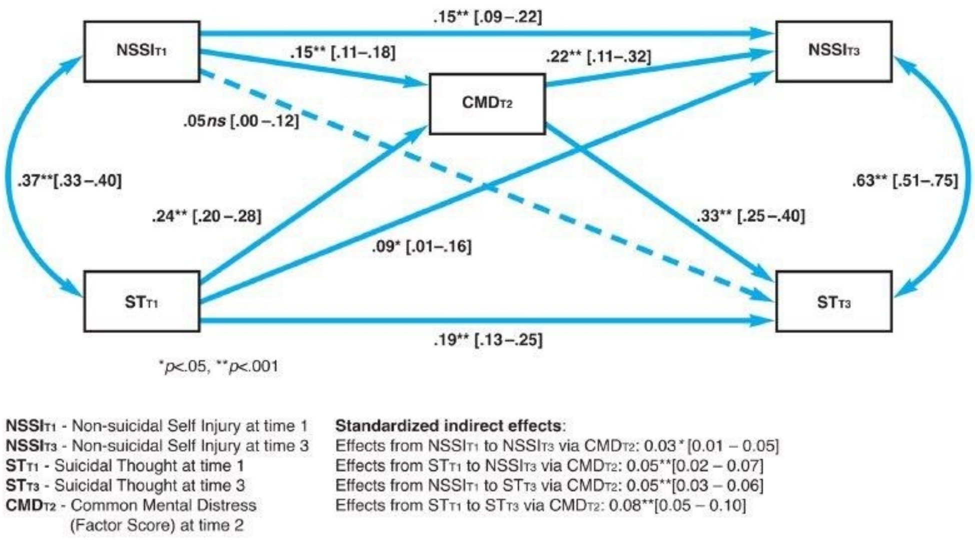

211x115mm (96 x 96 DPI)

Bifactor modelling:

Bifactor psychometric modelling is designed to extract variance common for all items in the model to generate one "general" factor. In addition to this general factor, specific factor/s may emerge, which are uncorrelated with each other or with the general factor. Specific factor/s contain the remaining variance after the extraction of the general factor[1]. St Clair et al. (2017) found in her psychometric study a bifactor model with one general factor and 5 specific factors, which fitted the data better than the correlated-factors model or second-order model. In our study, we first replicated St Clair et al. (2017) psychometric model in Cohort 1 (T1, T2, T3) and Cohort 2. In accordance with the original study, in our psychometric modelling the same measures of common mental illness frequently emerging during adolescence (depression, anxiety, psychotic experiences, obsessions and compulsions, conduct problems) as well as traits and characteristics commonly considered to contribute to mental wellness (well-being, self-esteem) were used as constructs contributing the general factor (see items below). Having replicated St Clair et al (2017) bifactor model, we then computed factor scores for the general factor – here termed *Common Mental Distress (CMD)*.

The confirmatory bifactor analysis in Cohort 1 was computed with the multiple group method (MGM) in Mplus 8 with the three data point used as a grouping variable; the same model was fitted to the data in each group. MGM in Mplus by default holds thresholds and loadings invariant across groups[2], thus allowing the comparison if the model fits data well in all groups under study (here data from the three measurement points). The effective sample for the 3 data waves was, respectively, n=2403, n=1815, n=1245 (Total N=5463). The overall chi-square test for the model was $\chi2$=33648.24 (*df*=14983, *p*=0.000), for Time 1 it was $\chi2$=14791.20, for Time 2 it was $\chi2$=10400.56 and for Time 3 it was $\chi2$=8456.47. The overall Root Mean Square Error of Approximation (RMSEA) for the model was 0.026 (0.026-0.027), Comparative Fit Index (CFI) was 0.969, Tucker-Lewis Index (TLI) was 0.969, and weighted root mean square residual (WRMR) was 2.91. The confirmatory bifactor analysis was used in Cohort 2 as well. The following fit indexes were obtained in Cohort 2: $\chi2$=7602.17 (df=4462, *p*=0.000), RMSEA=0.026 (0.025-0.027), CFI=0.96, TLI=0.96, WRMR= 1.34. The above-cited fit indexes suggest that the bifactor model fitted the data well in both cohorts.

In both analyses – for Cohort 1 and 2 – we used WLSMV estimator and THETA parametrisation with PROBIT link, and all items were treated as ordered-categorical variables.

Much debate in the literature has focused on the issue of interpretability of specific factors, i.e., whether they should be considered as measures of meaningful concepts or should be treated as comprising the residual, uninterpretable variance[3]. The general factor in St Clair et al (2017) study demonstrated high reliability and validity, as well as low measurement error compared to validity and error of the specific factors. As follows, we focused in our study only on this general (CMD) factor; we did not attempt to interpret or use in our analyses the specific factors, even though they emerged in our bifactor modelling, due to their relatively high measurement error and ambiguity of their theoretical interpretation. The list of items contributing to CMD factor with factor loadings on this factor in Cohort 1 (T1, T2, T3) and Cohort 2 are listed below in Supplementary Table 8.

For peer review only - http://bmjopen.bmj.com/site/about/guidelines.xhtml

Multiple imputation procedure in Cohort 1:

Missingness in Cohort 1 predominantly arose from longitudinal attrition – 24% at T2 and 48% at T3; a small fraction of data was also missing due to omissions of items (between 0 to 6%). Before performing imputations, we examined if longitudinal attrition was related to demographic variables and other variables under study. Indeed, we found small, yet statistically significant correlations between attrition at T2 and T3 and demographic and exposure variables at T1 (see Supplementary Table 7), thus indicating that the assumption of "missing completely at random (MCAR) is not met. Moreover, we performed Little's MCAR test and found that it was significant (p<.001). Therefore, we assumed that MAR condition was met. As follows, we imputed missing data under MAR condition in Cohort 1 at T2 and T3 with the following variables in one imputation model: CMD factor scores, NSSI and ST variables. We used the following auxiliary variables: research centre, sex, age, ethnicity, and Index of Multiple Deprivation (IMD) (as an indicator of a socioeconomic status[4]) as predictors of the missingness, in addition to main predictors – CMD factor scores, NSSI, and ST at T1.

Multiple imputations were computed in R program with MICE package[5]; convergence was examined by visual inspection of MCMC chains (with a maximum number of 20 iterations per chain and Gibbs sampling). Fifty-four (N=2403) datasets were generated to equal the percentage of missing data in CMD, NSSI, and ST at T3[6]. In terms of the imputation model, we used mean matching for continuous variables (CMD factor scores) and logistic regression for binary variables (NSSI and ST). The imputed 54 datasets were then used in pathway analysis (see the main manuscript and Supplementary Figure 3 for details) with MLM estimator in Mplus 7.4, which automates the process of analysing and combining parameter estimates from each imputed dataset using Rubin's rules[7].

For peer review only - http://bmjopen.bmj.com/site/about/guidelines.xhtml

Supplementary Table 1: List of all items used in the study

**Outcome measures:**
**Suicidal Thought (ST)**<br>*I thought about killing myself* (MFQ19, response options: *Always, Mostly, Sometimes, Never*)[Cohort 1 & 2]<br>This is one of the 4 items assessing suicidal thoughts in the 33-item Mood and Feelings Questionnaire (MFQ)[8] : MFQ16 - I thoughts that life was not worth living; MFQ17 - I thought about dying; MFQ18 – I thought my family would be better off without me; MFQ19 - I thought about killing myself. We used item 19, as it had the highest (.70) loading on this sub-subscale. Responses to this item were recoded into a binary format: no ST (original response option *Never*) and ST (original response options *Sometimes* or *Mostly* or *Always*). We did not include MFQ items 16-18 in CMD factor to avoid content overlap between the outcome measure (ST) and the predictor – the CMD factor.<br><br>**Non-Suicidal Self-Injury (NSSI)**<br>NSSI in Cohort 1 was assessed with one question from the Drug, Alcohol and Self-Injury (DASI) questionnaire asking about engaging in self-injury without suicidal intent during the last month:<br>*In the last month, have you tried to hurt yourself on purpose without trying to kill yourself? (*Response options: *Yes, No)*<br><br>NSSI in Cohort 2 was assessed with one question from the DASI questionnaire asking about life-time occurrence of NSSI:<br>[illegible]

Supplementary Table 9: Items comprising the Common Mental Distress (CMD) factor

**Items and associated measures**	**Standardised Factor Loadings**			
**The Moods and Feelings Questionnaire (MFQ)[11] [Cohort 1 & 2]**<br>**(response options: *Always, Mostly, Sometimes, Never*)**<br>*Note:* 4 items measuring suicidality were excluded to avoid content overlap between the measures of variables treated here as predictors (CMD, Depression) and the outcome variable (ST). We excluded 4 other items which caused model convergence problems: *I was less hungry than usual (MFQ3), I ate more than usual (MFQ4), It was hard for me to make up my mind (MFQ10), I slept a lot more than usual (MFQ33)*	**Cohort 1**			**Cohort 2**
	**Time 1**	**Time 2**	**Time 3**	
1. I felt miserable or unhappy. (MFQ1)	.69	.73	.71	.73
2. I didn't enjoy anything. (MFQ2)	.62	.70	.72	.67
3. I felt so tired I just sat around and did nothing. (MFQ5)	.53	.56	.57	.54

For peer review only

4. I was moving and walking more slowly than usual. (MFQ6)	.54	.59	.54	.52
5. I was very restless. (MFQ7)	.48	.54	.56	.49
6. I felt I was no good any more. (MFQ8)	.78	.82	.84	.77
7. I sometimes blamed myself for things that weren't my fault. (MFQ9)	.70	.74	.75	.73
8. I got grumpy and cross easily. (MFQ11)	.60	.65	.68	.65
9. I felt like talking a lot less than usual. (MFQ12)	.64	.66	.69	.65
10. I was talking more slowly than usual. (MFQ13)	.56	.64	.55	.59
11. I cried a lot. (MFQ14)	.64	.64	.68	.69
12. I thought there was nothing good for me in the future. (MFQ15)	.72	.77	.78	.72
13. I didn't want to see my friends. (MFQ20)	.69	.73	.70	.66
14. I found it hard to think properly or concentrate. (MFQ21)	.73	.77	.77	.72
15. I thought bad things would happen to me. (MFQ22)	.76	.77	.80	.81
16. I hated myself. (MFQ23)	.81	.82	.85	.80
17. I was a bad person. (MFQ24)	.73	.76	.78	.72
18. I thought I looked ugly. (MFQ25)	.65	.70	.70	.69
19. I worried about aches and pains. (MFQ26)	.46	.50	.50	.56
20. I felt lonely. (MFQ27)	.70	.74	.73	.74
21. I thought nobody really loved me. (MFQ28)	.75	.79	.83	.76
22. I didn't have any fun at school / college / work. (MFQ29)	.62	.67	.66	.58
23. I thought I could never be as good as other people my age. (MFQ30)	.76	.79	.78	.76
24. I did everything wrong. (MFQ31)	.83	.85	.87	.82
25. I didn't sleep as well as usual. (MFQ32)	.53	.57	.61	.60
**The Revised Children's Manifest Anxiety Scale (RCMAS)**[12] **Cohort 1 & 2**<br>**(response options: *Always, Mostly, Sometimes, Never*)**				
1. I had trouble making up my mind. (RCMAS1)	.60	.68	.71	.59
2. I worried when things did not go the right way for me. (RCMAS2)	.71	.77	.79	.78
3. Others seemed to do things more easily than I could. (RCMAS3)	.76	.80	.83	.76
4. Often I had trouble getting a breath. (RCMAS4)	.56	.60	.59	.55
5. I worried a lot of the time. (RCMAS5)	.78	.80	.82	.78
6. I was afraid of a lot of things. (RCMAS6)	.78	.80	.82	.77
7. I got angry easily. (RCMAS7)	.63	.68	.74	.68

For peer review only

For peer review only - http://bmjopen.bmj.com/site/about/guidelines.xhtml

8. I worried about what my parents would say to me. (RCMAS8)	.62	.67	.71	.65
9. I felt that others did not like the way I did things. (RCMAS9)	.73	.79	.78	.74
10. It was hard for me to get to sleep at night. (RCMAS10)	.55	.63	.58	.57
11. I worried about what other people thought about me. (RCMAS11)	.74	.79	.80	.71
12. I felt alone even when there were people with me. (RCMAS12)	.80	.84	.86	.85
13. Often I felt sick to my stomach. (RCMAS13)	.69	.74	.74	.76
16. I was tired a lot. (RCMAS16)	.62	.67	.69	.65
17. I worried about what was going to happen. (RCMAS17)	.77	.80	.81	.79
18. Other people my age were happier than me. (RCMAS18)	.79	.83	.83	.79
19. I had bad dreams. (RCMAS19)	.54	.59	.57	.62
20. My feelings got hurt easily when I was fussed at. (RCMAS20)	.75	.76	.78	.77
21. I felt someone would tell me I did things the wrong way. (RCMAS21)	.70	.77	.77	.71
22. I wake up scared some of the time. (RCMAS22)	.64	.74	.72	.67
23. I worried when I went to bed at night. (RCMAS23)	.67	.74	.73	.75
24. It was hard for me to keep my mind on my work. (RCMAS24)	.48	.58	.56	.55
25. I wiggled in my seat a lot. (RCMAS25)	.77	.79	.80	.76
27. A lot of people were against me. (RCMAS27)	.75	.80	.83	.80
28. I often worried about something bad happening to me. (RCMAS28)	.74	.79	.79	.80
**The Revised Leyton Obsessional Inventory (R-LOI)[13] Cohort 1 & 2**<br>**(response options: *Always, Mostly, Sometimes, Never*)**				
1. I felt I had to do things in a certain way, like counting or saying special words, to stop something bad from happening. (R-LOI1)	.53	.58	.50	.47
2. I had trouble finishing my homework or other jobs because I had to do things over and over again. (R-LOI2)	.58	.63	.64	.53
3. I hated dirt and dirty things. (R-LOI3)	.35	.44	.43	.39
4. I had a special number that I counted up to, or I felt I had to do things just that number of times. (R-LOI4)	.40	.46	.42	.41
5. I often felt guilty or bad about things I had done even though no one else thought I had done anything wrong. (R-LOI5)	.71	.77	.79	.73
6. I worried about being clean enough. (R-LOI6)	.48	.51	.55	.45

For peer review only - http://bmjopen.bmj.com/site/about/guidelines.xhtml

7. I moved or talked in a special way to avoid bad luck. (R-LOI7)	.38	.46	.38	.33
8. I worried a lot if I did something, not exactly the way I liked. (R-LOI8)	.60	.67	.66	.53
9. I was fussy about keeping my hands clean. (R-LOI9)	.35	.40	.41	.35
10. I had special numbers or words that I said because I hoped they kept bad luck or bad things away. (R-LOI10)	.43	.47	.47	.42

**Antisocial Behaviour Questionnaire (ABQ)[14] Cohort 1 & 2 (response options: *Always, Mostly, Sometimes, Never*)**				
1. I deliberately broke the rules or disobeyed people (e.g. parents, teachers or supervisors). (ABQ1)	.45	.48	.47	.38
2. I stole things (e.g. from home or a shop or school). (ABQ2)	.37	.40	.36	.26
3. I deliberately damaged property (e.g. broke windows or chairs or wrote graffiti or started fires). (ABQ3)	.35	.39	.39	.38
4. I skipped lessons/work, skived, or played truant from school. (ABQ5)	.36	.39	.40	.35
5. I deliberately lied or cheated to get what I wanted. (ABQ6)	.43	.39	.41	.40
6. I ran away from home (e.g. for half a day or overnight). (ABQ7)	.51	.56	.58	.56
**Rosenberg Self-Esteem Questionnaire (RSEQ)[15] Cohort 1 & 2 (response options: *Always, Mostly, Sometimes, Never*)**				
1. At times, I thought I was no good at all. (RSEQ1)	.82	.84	.85	.83
2. I was satisfied with myself. (RSEQ2)	-.58	-.61	-.60	-.53
3. I felt I had a number of good qualities. (RSEQ3)	-.53	-.55	-.56	-.52
4. I was able to do things as well as most people. (RSEQ4)	-.56	-.60	-.62	-.56
5. I felt I did not have much to be proud of. (RSEQ5)	.70	.73	.72	.70
6. I certainly felt useless at times. (RSEQ6)	.79	.81	.79	.77
7. I felt that I was as good as anyone else. (RSEQ7)	-.53	-.56	-.54	-.44
8. I wished I could have more respect for myself. (RSEQ8)	.62	.66	.68	.69
9. I felt that I was a failure. (RSEQ9)	.80	.82	.83	.75
10. I took a positive attitude toward myself. (RSEQ10)	-.60	-.63	-.63	-.56
11. I kept thinking about the things that I had done because I wasn't sure that they were the right things to do. (R-LOI11)	.71	.73	.71	.67

For peer review only - http://bmjopen.bmj.com/site/about/guidelines.xhtml

**Warwick-Edinburgh Mental Well-Being Scale (WEMWBS)[16] Cohort 1 & 2**<br>**(response options: *None of the time, Rarely, Some of the time, Often, All of the time*)**				
1. I've been feeling optimistic about the future. (WEMWBS1)	-.46	-.51	-.54	-.25
2. I've been feeling useful. (WEMWBS2)	-.52	-.58	-.60	-.33
3. I've been feeling relaxed. (WEMWBS3)	-.57	-.62	-.63	-.49
4. I've had the energy to spare. (WEMWBS5)	-.40	-.46	-.49	-.36
5. I've been dealing with problems well. (WEMWBS6)	-.57	-.63	-.64	-.46
6. I've been thinking clearly. (WEMWBS7)	-.62	-.67	-.68	-.48
7. I've been feeling good about myself. (WEMWBS8)	-.65	-.71	-.70	-.55
8. I've been feeling close to other people. (WEMWBS9)	-.44	-.50	-.52	-.28
9. I've been feeling confident. (WEMWBS10)	-.58	-.63	-.66	-.46
10. I've been able to make up my own mind about things. (WEMWBS11)	-.52	-.59	-.60	-.39
11. I've been feeling loved. (WEMWBS12)	-.49	-.54	-.60	-.29
12. I've been interested in new things. (WEMWBS13)	-.36	-.45	-.46	-.20
13. I've been feeling cheerful. (WEMWBS14)	-.61	-.67	-.67	-.49
**Psychotic-Like Experiences:**<br>**Cohort 1 – selected 10 items from the Schizotypal Personality Questionnaire (SPQ)[17]**<br>**Cohort 2 – selected 7 items from the Diagnostic Interview Schedule for Children (DISC)[18]**<br>**(response options: *Yes, No*)**				
1. Have you often mistaken objects or shadows for people or noises for voices? (SPQ4) **Cohort 1**	.38	.43	.41	*Not used*
2. I am sure I am being talked about behind my back. (SPQ9, DISC3) **Cohort 1 & 2**	.59	.67	.66	.60
3. Have you ever had the sense that some person or force is around you, even though you cannot see anyone? (SPQ13, DISC5) **Cohort 1 & 2**	.33	.38	.34	.41
4. Have you ever noticed a common event or object that seemed to be a special sign for you? (SPQ28, DISC8) **Cohort 1 & 2**	.33	.33	.35	.38
5. I often hear a voice speaking my thoughts aloud. (SPQ31, DISC10) **Cohort 1 & 2**	.33	.39	.34	.40
6. Have you ever seen things invisible to other people? (SPQ40, DISC13) **Cohort 1 & 2**	.36	.50	.37	.48
7. Do you sometimes feel that other people are watching you? (SPQ60, DISC19) **Cohort 1 & 2**	.53	.55	.59	.54
8. Do you ever suddenly feel distracted by distant sounds that you are not normally aware of? (SPQ61) **Cohort 1**	.40	.49	.45	*Not used*

For peer review only - http://bmjopen.bmj.com/site/about/guidelines.xhtml

9. Do you sometimes feel that people are talking about you? (SPQ63, DISC15) **Cohort 1 & 2**	.52	.56	.59	.60
10. Are your thoughts sometimes so strong that you can almost hear them? (SPQ64) **Cohort 1**	.44	.52	.50	*Not used*

For peer review only

Supplementary Table 2: Predictive power of Common Mental Distress versus the conventional psychopathology dimensions in Cohort 1_{T1} and Cohort 2: AUC (for ST and NSSI as criteria) and ORs for continuous and binary predictors (with cut-off point of 1SD)

		AUC		Suicidal thought (ST)				Non-suicidal self-injury (NSSI)			
				Continuous predictor		Binary (1SD cut-off)		Continuous predictor		Binary (1SD cut-off)	
		ST	NSSI	OR	95% C.I.	OR	95% C.I.	OR	95% C.I.	OR	95% C.I.
Common Mental Distress	Cohort 1_{T1}	.87	.83	7.07	[5.66 - 8.84]	15.60	[11.56 - 21.06]	4.15	[3.44 - 5.01]	8.93	[6.63 - 12.03]
	Cohort 2	.88	.72	6.79	[4.51 - 10.21]	20.97	[6.47 - 67.92]	2.38	[1.90 - 2.98]	4.00	[2.55 - 6.28]
Depression	Cohort 1_{T1}	.88	.83	5.10	[4.28 - 6.07]	15.60	[11.56 - 21.06]	3.21	[2.77 - 3.72]	8.28	[6.15 - 11.14]
	Cohort 2	.88	.70	7.18	[4.77 - 10.80]	15.32	[8.52 - 27.57]	2.14	[1.73 - 2.64]	3.56	[2.32 - 5.46]
Anxiety	Cohort 1_{T1}	.85	.81	4.82	[4.04 - 5.75]	13.62	[10.11 - 18.34]	3.75	[3.16 - 4.45]	7.61	[5.67 - 10.22]
	Cohort 2	.86	.71	5.69	[3.90 - 8.29]	10.51	[5.89 - 18.73]	2.24	[1.81 - 2.77]	3.68	[2.39 - 5.67]
Self-esteem (reversed)	Cohort 1_{T1}	.85	.83	4.81	[4.00 - 5.79]	15.62	[11.49 - 21.23]	3.75	[3.16 - 4.45]	9.86	[7.28 - 13.35]
	Cohort 2	.87	.65	6.42	[4.24 - 9.74]	15.16	[8.32 - 27.62]	1.79	[1.45 - 2.21]	3.34	[2.20 - 5.07]
Well-being (reversed)	Cohort 1_{T1}	.82	.80	4.29	[3.59 - 5.13]	10.31	[8.06 - 13.19]	3.45	[2.90 - 4.09]	6.66	[4.93 - 8.99]
	Cohort 2	.78	.61	2.88	[2.11 - 3.93]	5.27	[3.01 - 9.24]	1.44	[1.18 - 1.76]	2.19	[1.40 - 3.42]
Psychotic-like experiences	Cohort 1_{T1}	.74	.73	2.70	[2.32 - 3.13]	4.94	[3.70 - 6.60]	2.36	[2.03 - 2.74]	4.03	[2.98 - 5.45]
	Cohort 2	.74	.71	2.65	[2.00 - 3.50]	6.78	[3.89 - 11.83]	2.11	[1.72 - 2.58]	4.11	[2.69 - 6.27]
Antisocial trait*	Cohort 1_{T1}	.64	.63	1.65	[1.45 - 1.88]	2.67	[1.96 - 3.63]	1.79	[1.56 - 2.05]	2.48	[1.78 - 3.47]
Schizotypal trait	Cohort 1_{T1}	.79	.78	3.14	[2.71 - 3.64]	6.26	[4.70 - 8.32]	2.77	[2.39 - 3.21]	6.08	[4.52 - 8.19]
	Cohort 2	.76	.72	1.98	[1.66 - 2.36]	5.66	[3.23 - 9.91]	2.41	[1.93 - 3.01]	4.45	[2.90 - 6.83]
Conduct problems	Cohort 1_{T1}	.69	.67	1.87	[1.66 - 2.10]	3.38	[2.52 - 4.52]	1.67	[1.49 - 1.87]	3.46	[2.54 - 4.71]
	Cohort 2	.68	.61	2.00	[1.58 - 2.53]	3.78	[2.16 - 6.63]	1.54	[1.29 - 1.84]	2.13	[1.36 - 3.34]
Obsessions & compulsions	Cohort 1_{T1}	.76	.72	2.18	[1.94 - 2.45]	5.74	[4.25 - 7.75]	1.76	[1.57 - 1.98]	3.55	[2.58 - 4.89]
	Cohort 2	.71	.63	1.57	[1.31 - 1.88]	4.16	[2.37 - 7.28]	2.11	[1.64 - 2.71]	2.75	[1.79 - 4.22]

** measures were available only for Cohort 1_{T1}*

For peer review only - http://bmjopen.bmj.com/site/about/guidelines.xhtml

Supplementary Table 3: Association between ST and demographic variables in Cohort 1 (T1, T2, T3) and Cohort 2 (polychoric correlations)

	ST Cohort 1			ST Cohort 2
	T1	T2	T3	
Socioeconomic status (IMD)	-.05	-.01	-.01	.02
Research centre (0-Cambridge, 1-London)	-.12	-.04	-.03	not applicable
Ethnicity (1-white; 0-other)	-.08	-.02	-.04	-0.01
Age	-.05	-.02	-.05	0.03
Gender (0-Female, 1-Male)	-.10	-.08	-.01	0.03

All p-values non-significant

Supplementary Table 4: Association between NSSI and demographic variables in Cohort 1 (T1, T2, T3) and Cohort 2 (polychoric correlations)

	Cohort 1			Cohort 2
	T1	T2	T3	
Socioeconomic status (IMD)	.00	.00	.02	-.01
Research centre (0-Cambridge, 1-London)	-.01	-.01	.00	not applicable
Ethnicity (1-white; 0-other)	.00	.00	.00	.00
Age	-.02	-.04	-.01	-.02
Gender (0-Female, 1-Male)	.05	-.23	.02	.08

All p-values non-significant

Supplementary Table 5: Association between CMD and demographic variables in Cohort 1 (T1, T2, T3) and Cohort 2 (polychoric correlations)

	Cohort 1			Cohort 2
	T1	T2	T3	
Socioeconomic status (IMD)	-.02	-.02	-.01	.02
Research centre (0-Cambridge, 1-London)	.07*	.01	.01	not applicable
Ethnicity (1-white; 0-other)	-.08**	-.04	-.04	.04
Age	.01	.01	.01	.01
Gender (0-Female, 1-Male)	-.15**	-.15*	-.11**	.20**

*$p<.01$, **$p<.001$

For peer review only - http://bmjopen.bmj.com/site/about/guidelines.xhtml

For peer review only

Supplementary Table 6: Test of change in the prevalence of NSSI in Cohort 1: frequency over three time points (chi-square test)

	T1	T2	T3
NSSI	223	199	197
No-NSSI	2180	2204	2206

Chi-square=2.22, *df*=2, *p*=0.32, Yates' chi-square =2.04, *p*=0.35

Supplementary Table 7: Test of change in the prevalence of ST in Cohort 1: frequency over three time points (chi-square test)

	T1	T2	T3
NSSI	243	274	281
No-NSSI	2160	2129	2122

Chi-square=3.45, *df*=2, *p*=0.17, Yates' chi-square =3.26, *p*=0.19

For peer review only

Supplementary Table 8: Association between attrition in Cohort 1 at T2 and T3 and other variables in the study (Spearman rho)

	Attrition Cohort 1	
T1 variables:	**T2**	**T3**
Socioeconomic status (IMD index)[#]	-.07**	-.05*
Research centre (0-Cambridge, 1-London)	.05*	.05*
Ethnicity (1-white; 0-other)	-.05*	-.05*
Age	.07**	.05*
Gender (0-Female, 1-Male)	.09**	.12**
NSSI	-.01	.00
ST	-.01	-.03
Common Mental Distress	.06*	.05*
Depression	.06**	.05*
Impulsivity	.10**	.14**
Anxiety	.04*	.04*
Self - esteem (reversed)	.07**	.06*
Well - being (reversed)	.06*	.05*
Psychotic - like experiences even coerced	.00	.01
Antisocial trait	.08**	.12**
Schizotypal trait	.04*	.03
Conduct problems	.10**	.13**
Obsessions & compulsions	.03	.03

**p<.001, *p<.01
[#]higher number indicated *lower* socioeconomic deprivation

For peer review only

Item Response Theory (IRT) analysis

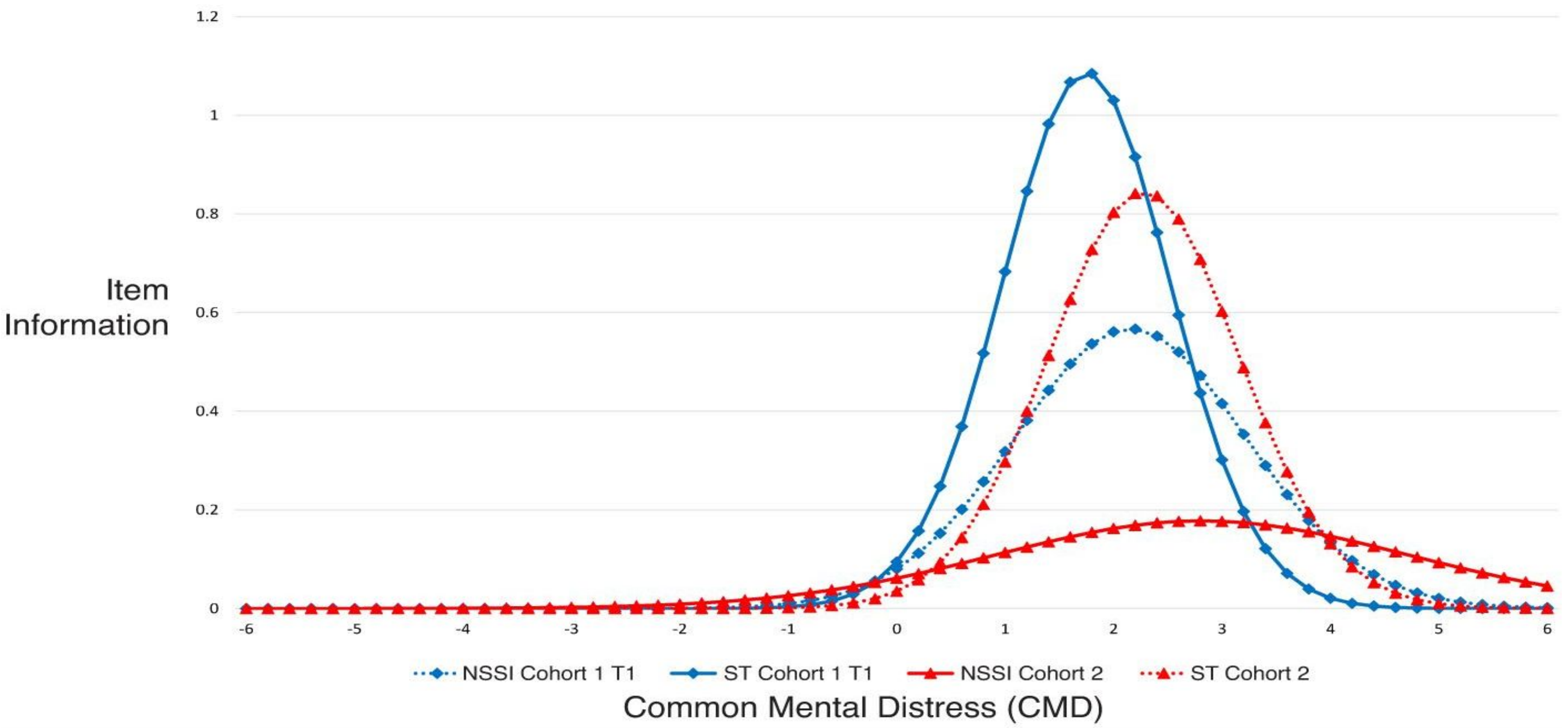

Supplementary Figure 1: Hierarchy of symptoms: the place of non-suicidal self-harm (NSSI) and suicidal though (ST) on the latent continuum of Common Mental Distress (in standard deviations) in Cohort 1_{T1} and Cohort 2.

IRT analysis on GP with NSSI and ST (here treated as indicators of CMD) showed that NSSI and ST provided information in above-average to high ranges of CMD, with the peak of the information curves for NSSI occurring around +2 SD in both cohorts. Information curve for ST in Cohort 2 was flatter, suggesting less contribution to the latent CMD dimension than ST had in Cohort 1_{T1} dataset. This may be due to the differences in age structure and psychopathology status in both cohorts. Nonetheless, in both cohorts the peak in the ST curves occurred between +2 and +3 SD (high end of the CMD dimension), showing that ST lies on the more severe spectrum of CMD dimension than NSSI does.

For peer review only - http://bmjopen.bmj.com/site/about/guidelines.xhtml

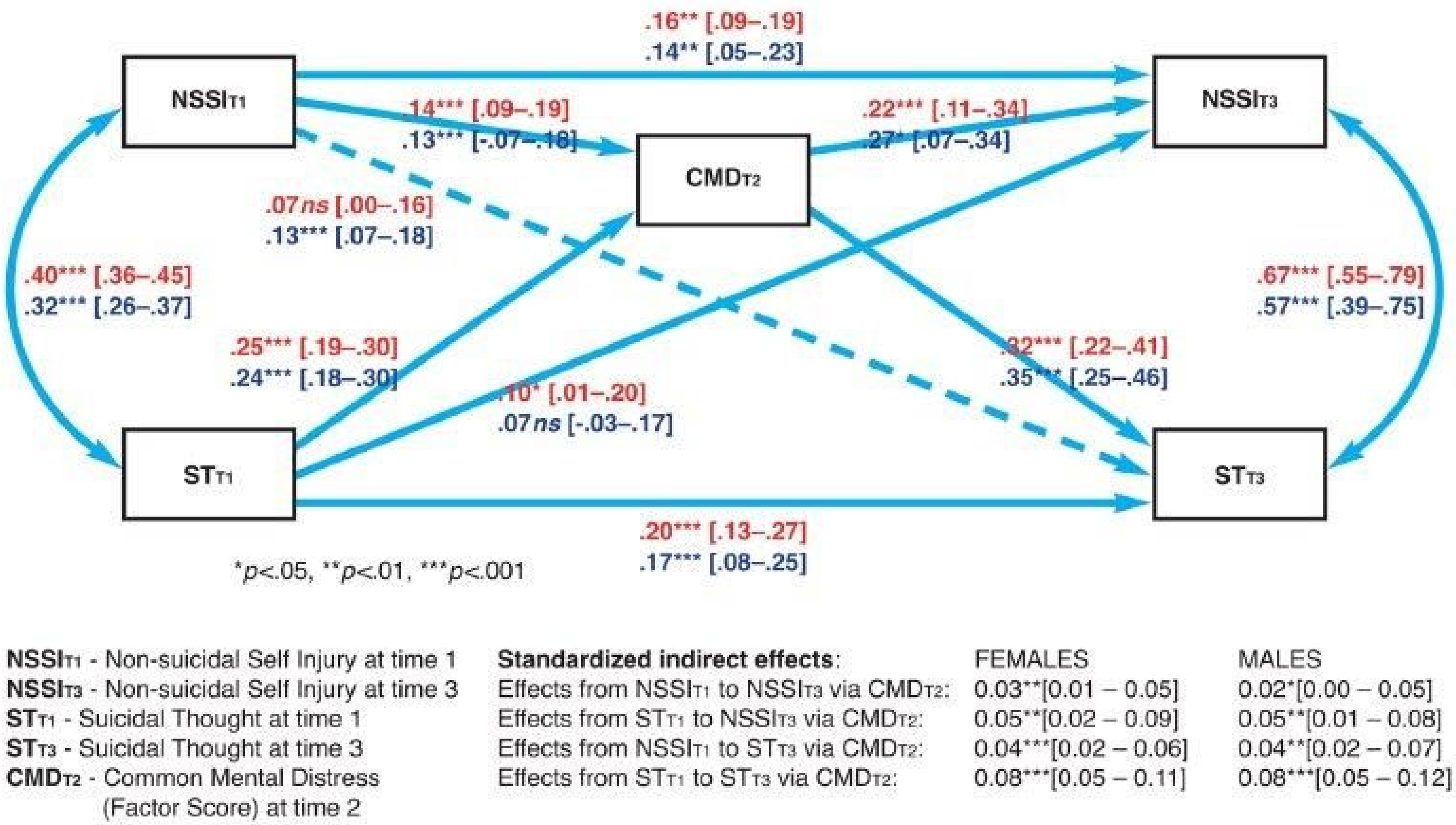

Supplementary Figure 2: Mediation effect of Common Mental Distress at time 2 (CMD_{T2}) moderated by sex (female n=1286; male n=1115) in the Cohort 1

Standardised pathway coefficients (with confidence intervals reported in squarer brackets) were obtained in multiple group pathway analysis in which sex was treated as a grouping variable. We tested the equivalence in pathway coefficients by means of comparing chi-square tests when the coefficient was "fixed" to be equal across sexes versus when it was free to vary across sexes[2]. We also tested the equivalence of fit indices of the model in both sexes. We found no evidence for differences in individual pathway coefficients or fit indices between sexes. This suggests that CMD at T2 mediated the longitudinal persistence of NSSI and ST in the same manner in females and males – no evidence of sex differences in the longitudinal mediation process was found.

Age and gender: Descriptive analysis

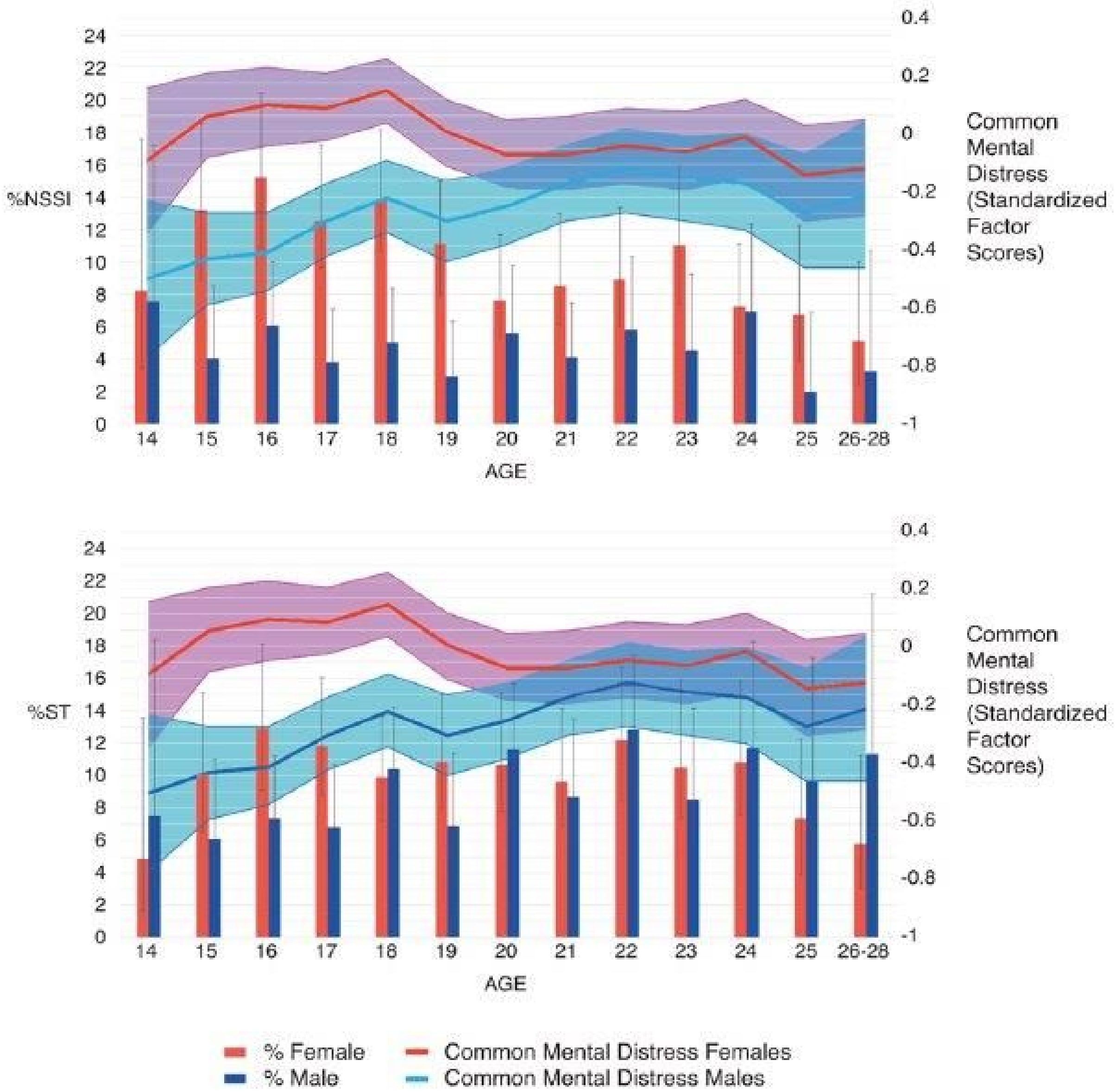

Supplementary Figure 3. Percentages of non-suicidal self-injury (NSSI), suicidal thoughts (ST) and levels of Common Mental Distress in age groups for both sexes in Cohort 1

To analyse the relationship between age, sex, NSSI, ST, and CMD descriptively, we grouped observations from all 3 time points in Cohort $1_{T1\text{-}T3}$ by age, rather than by data time point. This grouping allowed us to investigate levels of CMD, NSSI and ST in a broad age range of 14-28 years (note that this also entailed the inclusion of the same individuals from consecutive data sweeps (e.g., when an individual was 14, 15 and 16 years old) in the adjacent age groups). The histograms showing percentages of NSSI and ST with Wilson confidence intervals were plotted against the lines representing the means of CMD with confidence intervals for every age group for both sexes separately (Figure 3 above).

For peer review only - http://bmjopen.bmj.com/site/about/guidelines.xhtml

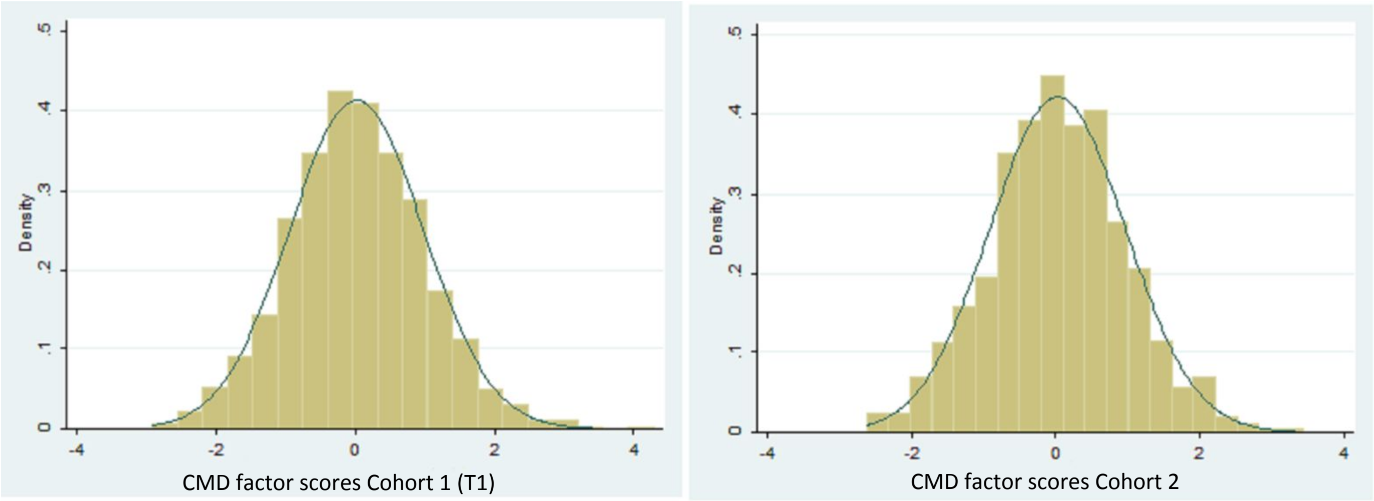

Supplementary Figure 4: Histograms of CMD factor scores in Cohort 1 (T1) and Cohort 2 with a schematic normal distribution line

For peer review only - http://bmjopen.bmj.com/site/about/guidelines.xhtml

Data collection tools:

Study data were collected and managed using REDCap electronic data capture tools[19] hosted at the University of Cambridge. REDCap (Research Electronic Data Capture) is a secure, web-based application designed to support data capture for research studies, providing 1) an intuitive interface for validated data entry; 2) audit trails for tracking data manipulation and export procedures; 3) automated export procedures for seamless data downloads to common statistical packages; and 4) procedures for importing data from external sources.

Group Information

NSPN (NeuroScience in Psychiatry Network: http://www.nspn.org.uk/) is a research consortium formed by the University of Cambridge and University College London, launched in November 2012 and supported by Wellcome Trust Award (095844/Z/11/Z). The group included the following members:

Principal investigators:

Edward Bullmore (CI from 01/01/2017) [1,2,3]

Raymond Dolan[4,5]

Ian Goodyer (CI until 01/01/2017) [1]

Peter Fonagy[6]

Peter Jones[1]

NSPN (funded) staff:

Michael Moutoussis[4,5]

Tobias Hauser[4,5]

Sharon Neufeld[1]

Petra Vértes[1,2]

Kirstie Whitaker[1,2]

Gita Prabhu[4,5]

Laura Villis[1]

Junaid Bhatti[1]

Becky Inkster[1]

Cinly Ooi[1]

Barry Widmer[1]

Ayesha Alrumaithi[1]

Sarah Birt[1]

For peer review only - http://bmjopen.bmj.com/site/about/guidelines.xhtml

For peer review only

Kalia Cleridou[5]

Hina Dadabhoy[5]

Sian Granville[5]

Elizabeth Harding[5]

Alexandra Hopkins[4,5]

Daniel Isaacs[5]

Janchai King[5]

Danae Kokorikou[5,6]

Harriet Mills[5]

Ciara O'Donnell[1]

Sara Pantaleone[5]

Aislinn Bowler[5]

Affiliated scientists:

Pasco Fearon[6]

Anne-Laura van Harmelen[1]

Rogier Kievit [4,7]

1 Department of Psychiatry, University of Cambridge, United Kingdom

2 Behavioural and Clinical Neuroscience Institute, University of Cambridge, United Kingdom

3 ImmunoPsychiatry, GlaxoSmithKline Research and Development, United Kingdom

4 Max Planck University College London Centre for Computational Psychiatry and Ageing Research,

University College London, UK

5 Wellcome Centre for Human Neuroimaging, University College London, United Kingdom

6 Research Department of Clinical, Educational and Health Psychology, University College London,

United Kingdom

7 Medical Research Council Cognition and Brain Sciences Unit, University of Cambridge, United Kingdom

For peer review only - http://bmjopen.bmj.com/site/about/guidelines.xhtml

References:

1 Reise, S. P. The rediscovery of bifactor measurement models. *Multivariate Behavioural Research*. 2012;47:667-696, doi:10.1080/00273171.2012.715555.

2 Muthen, L. & Muthen, B. *Mplus Users's Guide*. (Muthen & Muthen, 1998-2002).

3 Reise, S. P., Moore, T. M. & Haviland, M. G. Bifactor models and rotations: Exploring the extent to which multidimensional data yield univocal scale scores. *Journal of Personality Assessment*. 2010;92:544-559, doi:10.1080/00223891.2010.496477.

4 Noble M, McLennan, D, Wilkinson K, Whitworth A, & Barne H. The English Indices of Deprivation 2007. London: Department for Communities and Local Government. (2008).

5 van Buuren, S. & Groothuis-Oudshoorn, K. MICE: Multivariate Imputation by Chained Equations in R. *Journal of Statistical Software.*2011;45,1-67.

6 Sterne, J. A. C. *et al.* Multiple imputation for missing data in epidemiological and clinical research: Potential and pitfalls. *British Medical Journal.* 2009;339:157-160.

7 Rubin, D. B. *Multiple imputation for nonresponse in surveys.* (Wiley, 1987).

8 Hammerton, G., Zammit, S., Potter, R., Thapar, A. & Collishaw, S. Validation of a composite of suicide items from the Mood and Feelings Questionnaire (MFQ) in offspring of recurrently depressed parents. . *Psychiatry Research.* 2014;216:82-88.

9 Wilkinson P.O., Qiu T., Neufeld S., Jones P.B. & Goodyer I.M. Sporadic and recurrent non-suicidal self-injury before age 14 and incident onset of psychiatric disorders by 17 years: prospective cohort study. *British Journal of Psychiatry.*2018;212:222-226, doi:10.1192/bjp.2017.45.

10 Cassels M. *et al.* Poor family functioning mediates the link between childhood adversity and adolescent non-suicidal self-injury. *Journal of Child Psychology and Psychiatry*. 2018;59(8):881-887. doi: 10.1111/jcpp.12866

11 Angold, A. *et al.* The development of a short questionnaire for use in epidemiological studies of depression in children and adolescents. *International Journal of Methods in Psychiatric Research.*1995;5:237-249.

12 Reynolds, C. R. Concurrent validity of what I think and feel: The revised children's manifest anxiety scale. *Journal of Consulting and Clinical Psychology.* 1980;48:774-775. doi:10.1037/0022-006x.48.6.774 (1980).

13 Bamber, D., Tamplin, A., Park, R. J., Kyte, Z. A. & Goodyer, I. M. Development of a short Leyton Obsessional Inventory For Children and Adolescents. *Journal of the American Academy of Child and Adolescent Psychiatry.*2002;41:1246-1252.

14 St Clair C. M. *et al.* Characterising the latent structure and organisation of self-reported thoughts, feelings and behaviours in adolescents and young adults. *PLOS One.* 2017;12:1-27, doi:https://doi.org/10.1371/journal.pone.0175381.

15 Rosenberg, M. *Society and the adolescent self-image*. (Princeton University Press, 1965).

16 Tennant, R. *et al.* The Warwick-Edinburgh mental well-being scale (WEMWBS): development and UK validation. *Health and Quality of Life Outcomes* **5**, doi:10.1186/1477-7525-5-63 (2007).

17 Raine, A. The SPQ: A scale for the assessment of schizotypal personality based on DSM-III-R criteria. *Schizophrenia Bulletin.*1991;17:555-564.

18 Shaffer, D., Fisher, P., Lucas, C. P., Dulcan, M. K. & Schwab-Stone, M. E. NIMH Diagnostic Interview Schedule for Children Version IV (NIMH DISC-IV): description, differences from previous versions, and reliability of some common diagnoses. *Journal of the American Academy of Child and Adolescent Psychiatry*. 2000;39:28-38.

19 Harris PA, Taylor R, Thielke R, Payne J, Gonzalez N, Conde JG. Research electronic data capture (REDCap) – A metadata-driven methodology and workflow process for providing translational research informatics support. *Journal of Biomedical Informatics.* 2009;42(2):377-81.

For peer review only - http://bmjopen.bmj.com/site/about/guidelines.xhtml

STROBE Statement—Checklist of items that should be included in reports of ***cohort studies***

	Item No	Recommendation
**Title and abstract**	1	(*a*) Indicate the study's design with a commonly used term in the title or the abstract
		(*b*) Provide in the abstract an informative and balanced summary of what was done and what was found
**Introduction**		
Background/rationale	2	Explain the scientific background and rationale for the investigation being reported
Objectives	3	State specific objectives, including any prespecified hypotheses
**Methods**		
Study design	4	Present key elements of study design early in the paper
Setting	5	Describe the setting, locations, and relevant dates, including periods of recruitment, exposure, follow-up, and data collection
Participants	6	(*a*) Give the eligibility criteria, and the sources and methods of selection of participants. Describe methods of follow-up
		(*b*) For matched studies, give matching criteria and number of exposed and unexposed
Variables	7	Clearly define all outcomes, exposures, predictors, potential confounders, and effect modifiers. Give diagnostic criteria, if applicable
Data sources/ measurement	8*	For each variable of interest, give sources of data and details of methods of assessment (measurement). Describe comparability of assessment methods if there is more than one group
Bias	9	Describe any efforts to address potential sources of bias
Study size	10	Explain how the study size was arrived at
Quantitative variables	11	Explain how quantitative variables were handled in the analyses. If applicable, describe which groupings were chosen and why
Statistical methods	12	(*a*) Describe all statistical methods, including those used to control for confounding
		(*b*) Describe any methods used to examine subgroups and interactions
		(*c*) Explain how missing data were addressed
		(*d*) If applicable, explain how loss to follow-up was addressed
		(*e*) Describe any sensitivity analyses
**Results**		
Participants	13*	(a) Report numbers of individuals at each stage of study—eg numbers potentially eligible, examined for eligibility, confirmed eligible, included in the study, completing follow-up, and analysed
		(b) Give reasons for non-participation at each stage
		(c) Consider use of a flow diagram
Descriptive data	14*	(a) Give characteristics of study participants (eg demographic, clinical, social) and information on exposures and potential confounders
		(b) Indicate number of participants with missing data for each variable of interest
		(c) Summarise follow-up time (eg, average and total amount)
Outcome data	15*	Report numbers of outcome events or summary measures over time
Main results	16	(*a*) Give unadjusted estimates and, if applicable, confounder-adjusted estimates and their precision (eg, 95% confidence interval). Make clear which confounders were adjusted for and why they were included
		(*b*) Report category boundaries when continuous variables were categorized
		(*c*) If relevant, consider translating estimates of relative risk into absolute risk for a meaningful time period

For peer review only - http://bmjopen.bmj.com/site/about/guidelines.xhtml

Other analyses	17	Report other analyses done—eg analyses of subgroups and interactions, and sensitivity analyses
**Discussion**		
Key results	18	Summarise key results with reference to study objectives
Limitations	19	Discuss limitations of the study, taking into account sources of potential bias or imprecision. Discuss both direction and magnitude of any potential bias
Interpretation	20	Give a cautious overall interpretation of results considering objectives, limitations, multiplicity of analyses, results from similar studies, and other relevant evidence
Generalisability	21	Discuss the generalisability (external validity) of the study results
**Other information**		
Funding	22	Give the source of funding and the role of the funders for the present study and, if applicable, for the original study on which the present article is based

*Give information separately for exposed and unexposed groups.

Note: An Explanation and Elaboration article discusses each checklist item and gives methodological background and published examples of transparent reporting. The STROBE checklist is best used in conjunction with this article (freely available on the Web sites of PLoS Medicine at http://www.plosmedicine.org/, Annals of Internal Medicine at http://www.annals.org/, and Epidemiology at http://www.epidem.com/). Information on the STROBE Initiative is available at http://www.strobe-statement.org.

For peer review only

BMJ Open

How do the prevalence and relative risk of non-suicidal self-injury and suicidal thoughts vary across the population distribution of common mental distress (the p-factor) in two independent UK cohorts of young people?

Journal:	*BMJ Open*
Manuscript ID	bmjopen-2019-032494.R1
Article Type:	Original research
Date Submitted by the Author:	31-Oct-2019
Complete List of Authors:	Polek, Ela; University of Cambridge, Psychiatry; University College Dublin, Psychology<br>Neufeld, Sharon A. S.; Univ Cambridge<br>Wilkinson, Paul; University of Cambridge, Cambridge Neuroscience<br>Goodyer, Ian; Cambridge University, Psychiatry<br>St Clair, Michelle<br>Prabhu, Gita<br>Dolan, Ray<br>Bullmore, Edward<br>Fonagy, Peter<br>Stochl, Jan; University of Cambridge, Department of Psychiatry; NIHR Collaboration for Leadership in Applied Health Research & Care (CLAHRC) East of England,<br>Jones, Peter; University of Cambridge, Department of Psychiatry
**Primary Subject Heading**:	Public health
Secondary Subject Heading:	Mental health
Keywords:	EPIDEMIOLOGY, Child & adolescent psychiatry < PSYCHIATRY, PUBLIC HEALTH, Suicide & self-harm < PSYCHIATRY

SCHOLARONE™
Manuscripts

I, the Submitting Author has the right to grant and does grant on behalf of all authors of the Work (as defined in the below author licence), an exclusive licence and/or a non-exclusive licence for contributions from authors who are: i) UK Crown employees; ii) where BMJ has agreed a CC-BY licence shall apply, and/or iii) in accordance with the terms applicable for US Federal Government officers or employees acting as part of their official duties; on a worldwide, perpetual, irrevocable, royalty-free basis to BMJ Publishing Group Ltd ("BMJ") its licensees and where the relevant Journal is co-owned by BMJ to the co-owners of the Journal, to publish the Work in this journal and any other BMJ products and to exploit all rights, as set out in our licence.

The Submitting Author accepts and understands that any supply made under these terms is made by BMJ to the Submitting Author unless you are acting as an employee on behalf of your employer or a postgraduate student of an affiliated institution which is paying any applicable article publishing charge ("APC") for Open Access articles. Where the Submitting Author wishes to make the Work available on an Open Access basis (and intends to pay the relevant APC), the terms of reuse of such Open Access shall be governed by a Creative Commons licence – details of these licences and which Creative Commons licence will apply to this Work are set out in our licence referred to above.

Other than as permitted in any relevant BMJ Author's Self Archiving Policies, I confirm this Work has not been accepted for publication elsewhere, is not being considered for publication elsewhere and does not duplicate material already published. I confirm all authors consent to publication of this Work and authorise the granting of this licence.

For peer review only

How do the prevalence and relative risk of non-suicidal self-injury and suicidal thoughts vary across the population distribution of common mental distress (the p-factor) in two independent UK cohorts of young people?

Ela Polek[1,9], Sharon Neufeld[1], Paul Wikinson[1], Ian M. Goodyer[1], NSPN Consortium[1,2], Michelle C. St Clair[3], Gita Prabhu[2], Ray Dolan[2,7], Ed Bullmore[1,5], Peter Fonagy[4], Jan Stochl[1,5,8] & Peter B. Jones[1,5,6]

[1] Department of Psychiatry, University of Cambridge, UK

[2] Wellcome Centre for Human Neuroimaging, University College London, UK

[3] Department of Psychology, University of Bath, UK

[4] Division of Psychology and Language Sciences, University College London, UK

[5] NIHR Applied Research Collaboration East of England, UK

[6] NIHR Cambridge Biomedical Research Centre, UK

[7] Max Planck UCL Centre for Computational Psychiatry and Ageing Research, UK

[8] Department of Kinanthropology, Charles University, Czech Republic

[9] School of Psychology, University College Dublin

Correspondence to:

Professor Peter B. Jones

Herchel Smith Building

Cambridge Biomedical Campus

CB2 0SZ

UK

Fax:01223 336581

e-mail: pbj21@cam.ac.uk

the total word count: 5977

For peer review only - http://bmjopen.bmj.com/site/about/guidelines.xhtml

For peer review only

Abstract:

Objectives: To inform suicide prevention policies and responses to youths at risk by investigating whether suicide risk is predicted by a summary measure of common mental distress (CMD, (the p-factor)) as well as by conventional psychopathological domains; to define the distribution of suicide risks over the population range of CMD; to test whether such distress mediates the medium-term persistence of suicide risks.

Design: Two independent samples of young people studied during three sweeps: the Neuroscience in Psychiatry (NSPN) 2400 cohort (n=2403) and the ROOTS cohort (n=1074); Cohorts 1 and 2, respectively.

Setting: Population-based in two UK centres.

Participants: Volunteers age 14-24 years recruited from primary health care registers, schools and colleges; advertisements to complete quotas in age-sex-strata.

Method: We analysed questionnaire data from Cohort 1 (sweeps 1-3) and Cohort 2 (sweep 3), collected between November 2012 – December 2016 and February 2008 – December 2009, respectively. We calculated a CMD score using confirmatory bifactor analysis; used logistic regressions to determine adjusted associations between risks and psychopathology (in continuous and above-the-norm categorical format); curve-fitting to examine the relative prevalence of suicidal thoughts (ST) and non-suicidal self-injury (NSSI) over the population distribution of CMD; and pathway mediation models to examine longitudinal associations.

Results: We found a dose-response relationship between levels of CMD and risk of suicide. The majority of all subjects experiencing ST and NSSI (78% and 76% in Cohort 1, and 66% and 71% in Cohort 2) had CMD scores no more than two standard deviations above the population mean; higher scores indicated the highest risk but were, by definition, infrequent. CMD mediated the longitudinal course of both ST and NSSI.

For peer review only - http://bmjopen.bmj.com/site/about/guidelines.xhtml

Conclusions. NSSI and ST in youths reflect common mental distress that also mediates their persistence. Universal prevention strategies reducing levels of CMD in the whole population without recourse to screening or measurement may prevent more suicides than approaches targeting youths with the most severe distress or with psychiatric disorders.

Article summary

Strengths and limitations of this study

- Replication of the findings in two independent cohorts strengthens confidence in the findings.
- Sample attrition was a limitation in both cohorts.
- Multiple imputations mitigated biases arising from attrition.

For peer review only - http://bmjopen.bmj.com/site/about/guidelines.xhtml

Introduction

Adolescence sees the onset of a range of psychopathology including suicidal thoughts (ST) and non-suicidal self-injury (NSSI)[1-3] that individually or together convey heightened risk of suicide attempts[4-6]. Non-suicidal and suicidal self-harm predict completed suicide[7], the second most common cause of deaths among 10 to 24-year-olds, worldwide[8]. Moreover, ST and NSSI are significant problems in their own right, representing a considerable burden to individuals, their families and health services. Prediction and prevention of self-harm and suicide in young people are priorities but NSSI (5-42% in community samples[9,10]) and ST (15-25% in community samples[11,12]) are common so it is difficult to predict who will ultimately make a serious attempt[13] or die by suicide. Indeed, the usefulness of clinical risk protocols relying on the identification of a psychiatric diagnosis is questionable[14,15]. The same problems affect public health suicide prevention programmes. A seminal study revealed a high prevalence of false-negatives in prospective identification of suicide[16]. Prevention policies that embrace the whole population might overcome these difficulties but lack theoretical or empirical foundations[1].

Suicidal thoughts and behaviours are routinely considered as markers of depression (e.g., in DSM-5) but by no means all young people dying by suicide have had a mood disorder[17]. NSSI is strongly associated with the risk of suicide when occurring in combination with any internalising or externalising symptoms[18,19], or with any psychiatric diagnosis[20], particularly multiple diagnoses[21]. Thus, this risk might be better predicted by multiple symptoms rather than by the presence of a single disorder, such as depression.

Recent studies suggest that a broad range of symptoms conventionally seen as components of distinct disorders are better construed as manifestations of a single, latent dimension distributed within the general population. This dimension has been variously referred to as the p-factor[22], general psychopathology[23] or, as we prefer here, common mental

For peer review only - http://bmjopen.bmj.com/site/about/guidelines.xhtml

distress (CMD)[24,25]. Parsimonious statistical models with dimensions that encompass low-prevalence phenomena such as psychotic experiences, fit empirical data better than models with distinct disorders[22,26]. High co-morbidity of psychiatric diagnoses, shared causal factors and treatments, and trans-diagnostic psychological and neural correlates support the validity of a CMD concept[22-24,26-29]. Suicide risk is related to multiple symptoms or disorders (and thus to higher CMD scores), not the presence of one specific symptom or disorder, so it is important to understand the nature of dose-response relationships between CMD and suicide risks. This could guide a clinical response in the face of suicide risk[30] and also shape population-based suicide prevention.

In this study, we describe the presence of a CMD dimension in young people aged 14-26 years and the occurrence of ST and NSSI referred to collectively, hereafter, as a suicide risk. We draw on a psychometric study[25] that demonstrated high theoretical validity and high measurement qualities of the CMD factor comprising measures of common mental illness (depression, anxiety, psychotic experiences, obsessions and compulsions) as well as traits and characteristics commonly considered to contribute to the general level of mental health (antisocial trait, well-being, self-esteem). Our approach had three steps whereby we:

1. Tested associations between CMD and suicide risk, and contrasted CMD with specific psychopathological domains, exploring the utility of this summary measure;
2. Defined the prevalence and relative risk of NSSI and ST across the distribution of CMD;
3. Established whether the CMD_{T2} dimension measured at time 2 mediate the relationship between ST_{T1} and $NSSI_{T1}$ at time 1 and $NSSI_{T3}$ and ST_{T3} at time 3.

We used data from two population-based cohorts with complementary designs and very similar measures. In step two we used cross-sectional data from Cohort 1, time 1 (used as a

For peer review only - http://bmjopen.bmj.com/site/about/guidelines.xhtml

discovery sample) and Cohort 2 (used as a stepwise replication sample); in the third step we used three longitudinal waves of Cohort 1 (see details in Method).

Method

Study Design and Participants

Cohort 1

Participants in the NSPN 2400 Cohort[31] were recruited largely via postal invitations sent through general practitioners and schools in Cambridgeshire and Greater London, UK. Data collection was carried out in two research centres: University College London and the University of Cambridge between November 2012 and December 2016. Purposive sampling obtained at least 200 males and 200 females from the community in 5 age groups: 14-15, 16-17, 18-19, 20-21, 22-24 years. Three data collections took place a year apart (T1-T3). At T1, 2403 individuals returned questionnaires (average age 18.9 years, SD=3.0; 54% females); at T2, 1815 returned questionnaires (76% response, average age 20.0 years, SD=3.1; 56% female), and 1245 at T3 (52% of baseline; average age 21.0 years, SD=3.1; 59% female).

Cohort 2

The ROOTS study[32] was used for replication of findings from Cohort 1. Two-stage sampling involved random selection of 27 schools in Cambridgeshire, UK. Eighteen schools agreed to participate; invitations were sent to 14-year-olds randomly selected from class registers and to their parents; 1238 students participated in the initial data collection (55% female) (and further 4 data collection waves took place). Note that in the current analysis we used only the data from the third data sweep collected between February 2008 and December 2009, when participants were of average age 17.5 years, *SD*=0.3 (N=1074, 56% female; 87% of baseline sample), the closest age to T1 of Cohort 1.

For peer review only - http://bmjopen.bmj.com/site/about/guidelines.xhtml

Both cohorts comprised predominantly white European (77% in Cohort 1 and 87% in Cohort 2) young people, consistent with the self-ascribed demographics of the two study populations. Written consent from participants age 14 or 15 years was supplemented by written consent from their parent or legal guardian; older participants gave their own written consent. Ethical approval was obtained for Cohort 1 from the National Health Service Research Ethics Service (# 97546) and for Cohort 2 from the Cambridgeshire 2 REC (# 03/302).

Measures

Sociodemographic information was collected using routine methods[31,33]. The Index of Multiple Deprivation (IMD), a summary measure of the socioeconomic status of participants' residential neighbourhood, is calculated from census information[34]. Questionnaires of mental illness and wellness are set out in Table 1 and items are listed in the Supplementary table 1. Scores in questionnaires were computed according to published manuals or validation studies (cited in Table 1), standardized to unify their measurement scales.

Table 1

Statistical analysis

Confirmatory bifactor analysis with a weighted least square mean and variance adjusted (WLMSV) estimator in Mplus 7.4 was used to compute factor scores for CMD in the three data sweeps of Cohort 1 and Cohort 2 based on the model validated elsewhere[25] (see CMD measures in Table 1 beneath; the list of used items and details of bifactor modelling can be found in the Supplementary table 1). CMD factor scores were then used in all subsequent computations. Next, we addressed attrition in Cohort 1 by means of multiple imputations (see details in the Supplement).

To prove that NSSI and ST were predicted by multiple psychopathological domains and also by CMD (which represents a summary of those domains), we used Stata 12 to

For peer review only - http://bmjopen.bmj.com/site/about/guidelines.xhtml

compute for Cohort 1_{T1} and Cohort 2 data sensitivity / specificity indicator – the area under the curve (AUC – reported in the Supplementary table 2) for NSSI and ST as criteria. We computed a series of logistic regressions, estimating odds ratios (OR) with confidence intervals for each predictor (treated as categorical with the cut-off point above 1SD and then continuous), while we controlled for effects of age and sex (Figure 1).

For step two, distributions of CMD scores in both cohorts were plotted against lines representing percentages of subjects reporting NSSI and ST within bands of CMD expressed as standard deviations (upper panel of Figure 2) and against bar histograms representing NSSI and ST frequencies in both cohorts (lower panel of Figure 2). In addition, NSSI and ST information curves were computed to determine in what range of the CMD dimension these items are located (see Supplementary figure 1).

Using Cohort $1_{T1\text{-}T3}$ data for step three, we examined the longitudinal relationship between CMD, NSSI and ST (in particular the predictive role of CMD in persistence of NSSI and ST): we computed direct and mediation (via CMD_{T2}) effects of ST_{T1} and $NSSI_{T1}$ on $NSSI_{T3}$ and ST_{T3} in a pathway mediation model with confidence intervals in Mplus 7.4 (computing bias-corrected bootstrapping was not possible due to the use of multiply imputed datasets). We computed this model for the total sample (Figure 3) and then for both sexes separately (Supplementary figure 2) using the Multiple Group Method, so as to test a moderated-mediation model (with CMD_{T2} as a mediator, and sex as a moderator). Age was a control variable. In both pathway analyses CMD_{T2} factor scores (computed on imputed data, as described above) were modelled as observed variables.

Results

Step one: Associations of NSSI and ST with demographic and psychopathological variables

For peer review only - http://bmjopen.bmj.com/site/about/guidelines.xhtml

In both cohorts NSSI and ST were unrelated to demographic variables, including sex and age (See Supplementary tables 3 and 4); CMD was negatively related to male gender (Supplementary table 5). When examined descriptively over the pooled age groups, the prevalence of NSSI and ST mirrored the CMD levels (see Supplementary figure 3). CMD and all conventional psychopathological predictors of NSSI and ST had statistically significant ORs in logistic regression models (see Figure 1 and Supplementary table 2).

Figure 1

Prevalence of NSSI and ST in the two cohorts

In Cohort 1 (N=2403) there was no statistically significant change in the prevalence of NSSI (within the last month) over the three time points: in the imputed data 9.3% (n=223) reported $NSSI_{T1}$, 8.3% (n=199) $NSSI_{T2}$ and 8.2% (n=197) $NSSI_{T3}$. Similarly, there was no statistically significant change in prevalence of ST (within the last two weeks) over the three time points: 10.1% (n=243) ST_{T1}, 11.4% (n=274) ST_{T2} and 11.7% (n=281) ST_{T3} (see Supplementary tables 6 and 7).

In Cohort 2 (N=1074), 11.7% (n=126) reported lifetime NSSI and 5.4% (n=58) reported ST within the two last weeks. Accuracy and precision of these prevalence estimates were affected by attrition (see *Discussion: limitations*). Attrition in Cohort 1 at T2 and T3 was only marginally related to demographic and exposure variables at T1 (Spearman's rho 0.05-0.12), but unrelated to the outcome – NSSI and ST (see Supplementary table 8).

Step two: Associations of NSSI and ST with CMD

Next, we focused on absolute risk and the numbers of NSSI and ST events generated by these risk functions. The dose-response curves in the upper panel of Figure 3 show that relative risks of NSSI and ST increased markedly with increasing severity of CMD, the highest risks being in those with very high scores beyond two standard deviations above the mean. On the

For peer review only - http://bmjopen.bmj.com/site/about/guidelines.xhtml

other hand, most participants from both cohorts who reported NSSI or ST had mild (one SD above the mean) to moderate (two SD above the mean) CMD scores (lower panel of Figure 3). CMD was normally distributed (see Supplementary figure 4) so these scores were much more common; only a minority of the total reports came from the few participants with very high CMD (>2 standard deviations above mean CMD). Thus, the majority of subjects experiencing ST or NSSI (Cohort 1: 78% and 76%; Cohort 2: 66% and 71%, respectively) had CMD scores within two standard deviations above the population mean. Very high CMD scores indicated the highest suicide risk but were rare, so generated the minority of events.

Figure 2

Step three: Mediating effect of CMD on suicide risks in Cohort 1 over time

Cohort 1 CMD_{T2} contributed to the longitudinal persistence of NSSI and ST (i.e. $NSSI_{T1}$ predicted $NSSI_{T3}$ directly, and via mediation through CMD_{T2}; it also completely mediated the longitudinal effect of $NSSI_{T1}$ on ST_{T3}). Moreover, CMD_{T2} contributed to the longitudinal persistence of ST (i.e. ST_{T1} predicted ST_{T3} directly, as well as via mediating variable - CMD_{T2}). Overall, CMD_{T2} was a stronger predictor of $NSSI_{T3}$ and ST_{T3} than the antecedent variables measured at T1 (see Figure 3). There were no significant sex differences in direct and mediation pathways, showing that the mediation effects of CMD_{T2} were not moderated by sex (Supplementary figure 2 and Supplementary table 9). Age_{T1} was not a significant predictor of any variable in the model; the results when age was controlled for were very similar to those without controlling for age (differences in coefficients were in the second decimal place digits).

Figure 3

Discussion

In the present study, all the domains of psychopathology and mental wellness available (depression, anxiety, self-esteem, well-being, psychotic-like experiences, antisocial trait,

For peer review only - http://bmjopen.bmj.com/site/about/guidelines.xhtml

schizotypal trait, conduct problems, obsessions and compulsions) predicted risk of non-suicidal self-injury (NSSI) and suicidal thoughts (ST). Thus, the common mental distress factor with a normal population distribution appeared a parsimonious and efficient summary of these domains and was, itself, a key predictor of suicide risk in both cohorts. NSSI and ST were not confined to participants scoring in the very high, quasi-clinical range for CMD. Around half of all participants expressing NSSI or ST came from those scoring up to one standard deviation above mean CMD in a dose-response manner. The majority expressing these phenomena (two thirds to three quarters) scored within 2SD above the mean (Figure 2). Regarding medium-term determinants of persistent NSSI and ST we showed (Figure 3) that CMD_{T2} mediated the persistence of NSSI and ST over two years, independent of gender and age. This mediation operates in two stages: first, ST and NSSI persist because these behaviours are markers for worsening CMD in the general population. This extends findings in adolescents with depressive disorder, where suicidal thoughts are a predictor of poor outcome[35]. Second, this greater CMD, itself, predicts the risk for further suicidal thoughts and behaviours.

Strengths

Both cohorts were designed on epidemiological principles to capture behavioural and psychological variation in the population during the post-pubertal epoch during which risk for psychopathology accelerates. Replication of the findings in these independent cohorts strengthens confidence in the findings, as does internal consistency between cross-sectional associations found in both cohorts, and longitudinal associations found in Cohort 1.

Limitations

For peer review only - http://bmjopen.bmj.com/site/about/guidelines.xhtml

Sample attrition was the main bias in both cohorts. Each retained more young women than men; we found marginally higher attrition among lower socio-economic class, participants of non-white ethnicity and those with higher CMD (Supplementary table 8). Cohort 1 is robustly representative of the England and Wales population[31], whereas Cohort 2 under-represents participants with lowest socioeconomic status[32]. However, we have no reason to suppose that attrition biased our results, as it was unrelated to NSSI and ST (Supplementary table 8). If there was a bias, it probably limits power rather than skewing an effect and is mitigated by replication between the cohorts. We used multiple imputation to minimise this bias.

There was only modest reliability of our obsessionality measure and a skewed measure of conduct problems in Cohort 1. A completely comprehensive range of psychopathological (and behavioural) items was unavailable; we did not have measures of unstable or abnormally elevated mood, addictions, eating disorders or hyperactivity. Thus, our measurement of CMD focused primarily on internalising rather than externalising symptoms. Future studies could include a broader range of measures and extend the investigation into clinical populations to improve measurement precision at the highest levels of CMD. Although ethnicity and socioeconomic status (indicated by IMD) were unrelated to ST and NSSI (Supplementary tables 3 and 4), and thus were not included in our analyses, we did not control for the effect of other possible confounders such as adverse life experiences, early trauma, family structure or more detailed information about family socio-economic situation (unemployment, poverty etc.). Finally, we could not account for the effects of clustered design in the modelling, due to unavailability of the information about clustering of participants in both cohorts.

Implications & Conclusions

For peer review only - http://bmjopen.bmj.com/site/about/guidelines.xhtml

Our findings provide yet more evidence that a latent mental distress factor, conceptually akin to the p-factor, is a useful summary measure of psychopathology in the general population[24], diagnostic[22], and clinical[23] samples. We speculate that psychopathological items accumulate in a probabilistic manner rather than in diagnostic clusters, with common phenomena concerning depression and anxiety much more likely to occur before rarer phenomena such as NSSI, ST or psychotic experiences. Less frequent phenomena begin to co-occur as the severity of psychological disorder (or CMD) increases, in terms of more mental and behavioural phenomena or symptoms. This begins to yield clusters linked by common items that current diagnostic systems tend to ignore. This is consistent with the co-occurrence of suicidal risk and psychotic experiences seen in other[36-38] studies of young people, and with the present IRT analysis showing that NSSI and ST are measuring the higher end of CMD (Supplementary figure 1). The approach we have followed illustrates the value of moving away from categorical classification and embracing an empirically-rooted, dimensional, hierarchical taxonomy in psychopathology research[39]. Such hierarchical approaches to phenomenological classification had been put forward before[40] or shortly after[41] the publication of DSM-3 and its successor classifications. Hierarchical models merit renewed interest[42], as they may resolve problems of comorbidity[26] as well as overlapping causes and biological mechanisms for suicide risk and other phenomena[43,44]. In contrast to the CMD idea, there is also increasing interest in approaches focusing on individual symptoms and experiences, particularly to guide individual clinical interventions, rather than grouping the symptoms into diagnostic categories or higher-order constructs[45]. Future studies may investigate and compare the utility of such novel approaches (CMD and item-focused approach) for clinical practice and public health policies.

Our findings also have major implications for intervention and prevention of suicidal thoughts and behaviours. Clinically, the results suggest that NSSI and ST should never be

For peer review only - http://bmjopen.bmj.com/site/about/guidelines.xhtml

dismissed or downplayed when they occur in young people without clear evidence of psychiatric disorder, a logical fallacy because NSSI and ST are *themselves* indicators of higher distress on a CMD factor. NSSI and ST will usually, but not always occur with other, more common psychopathology and their co-occurrence is a strong risk factor for suicide attempts[6]. Thus, NSSI and ST merit a swift professional response regardless of whether or not they occur with other symptoms that take individuals beyond conventional clinical thresholds and trigger traditional clinical risk protocols. Our findings help explain why research focused on high-risk subjects has yet to translate into useful clinical prediction tools[14,15,45].

From a public health and prevention perspective, the fact that rates of NSSI and ST begin to accelerate at levels of CMD well within a normal or non-clinical range argues strongly for universal interventions overtly aimed at lowering the population mean CMD and shifting the curve to the left. This should be alongside targeted approaches and effective clinical services[46]. Strategies concentrated on clinical populations, those with evidence of a psychiatric disorder or other individual markers will miss the majority of individuals experiencing ST or engaging in NSSI because there are so few compared with those at lower risk: the *prevention paradox*[30].

Defining putative universal interventions to shift the population distribution of CMD will require careful research that can draw from other areas of medicine such as cardiovascular disease and stroke[30]. Elements have been widely scoped in the USA[15] and elsewhere, but not for constructs of population health and wellbeing such as CMD. Interventions may involve decreasing common triggers[15] or improving young people's abilities to cope with stressors[47, 48, 49].

For peer review only

Conflict of Interest Disclosures

E.P., S.N., I.M.G., and J.S. have no competing interests. E.B., P.F., and P.B.J. are in receipt of National Institute for Health Research (NIHR) Senior Investigator Awards (NF-SI-0514-10157, and NF-SI-0514-10117. P.F. was in part supported by the NIHR Collaboration for Leadership in Applied Health Research and Care (CLAHRC) North Thames at Barts Health NHS Trust. P.W. has recent/current grant support from NIHR, Cambridgeshire County Council and CLAHRC East of England. P.W. discloses consulting for Lundbeck and Takeda; P.B.J. discloses consulting for Janssen and Ricordati. E.B. is employed half-time by the University of Cambridge and half-time by GlaxoSmithKline in which he holds stock.

Data sharing/ Data availability

E.P. had full access to all the data in the study and takes responsibility for the integrity of the data and the accuracy of the data analysis. The data are deposited in the University of Cambridge Data Repository, with the placeholder DOI https://doi.org/10.17863/CAM.25331 available to researchers via openNSPN@medschl.cam.ac.uk.

Funding/Support and Acknowledgments

The ROOTS study was supported by a Wellcome Trust Grant (Grant number 074296) to I.M.G. and P.B.J., the NIHR Collaborations for Leadership in Applied Research and Care (CLAHRC) East of England, and the NIHR Cambridge Biomedical Research Centre. The NSPN study was supported by the Wellcome Trust Strategic Award (095844/Z/11/Z) to I.M.G., E.B., P.B.J., R.D., P.F. The work has been carried out in the Department of Psychiatry, University of Cambridge. We wish to thank the NSPN and ROOTS participants and Dr Golam Khandaker for his comments.

Role of the Funder/Sponsor

The funding sources had no role in the design and conduct of the study; collection, management, analysis, and interpretation of the data; preparation, review, or approval of the manuscript; and decision to submit the manuscript for publication.

NSPN Consortium Information –see the Supplement

For peer review only - http://bmjopen.bmj.com/site/about/guidelines.xhtml

Patient and public involvement: Patients and the public were not involved in the design or planning of the study.

For peer review only

For peer review only - http://bmjopen.bmj.com/site/about/guidelines.xhtml

Table 1. Measures used in both cohorts

Variables	*Measures*	Cohorts	
**Outcome variables:**		$NSPN_{T1-T3}$ (1)	$ROOTS_{age\ 17}$ (2)
Suicidal thoughts (ST)	One item from the MFQ[50]: I thought about killing myself. Responses were recoded into a binary format: no ST (original response option *Never)* and ST (original response options *Sometimes* or *Mostly* or *Always*).	×	×
Non-suicidal self-injury (NSSI)	One question from the Drug, Alcohol and Self-Injury (DASI)[25] questionnaire asking about engaging in self-injury without suicidal intent during the last month. Responses were recoded into a binary format indicating the occurrence of NSSI or lack of thereof.	×	
	One question asking about the occurrence of lifetime NSSI (DASI)[25]		×
**Predictors:**			
Conduct problems	11-item Antisocial Behaviour Questionnaire[25]	×	×
Anxiety	28-item Revised Children's Manifest Anxiety Scale[51]	×	×
Depression	29 items from the 33-item MFQ[50] (all items except for 4 items measuring suicidality)		
Obsessions and compulsions	11-item Revised Leyton Obsessional Inventory[52]	×	×
Psychotic-like experiences	11 items selected from the 74-item Schizotypal Personality Questionnaire (SPQ)[53]	×	
	11 items from the 20-item semi-structured interview from the Diagnostic Interview Schedule for Children-IV[54]		×
Self-esteem	10-item Rosenberg Self-Esteem Questionnaire (*)[55]	×	×
Well-being	14-item Warwick-Edinburgh Mental Well-Being Scale(*)[56]	×	×
Impulsivity	15 items from the 30-item Barratt Impulsiveness Scale[57] selected based on exploratory factor analysis - loadings above .25	×	
Antisocial traits	Total score from the 17-item Antisocial Process Screening Device (APSD)[58]	×	
Schizotypal traits	Total score from the 74-item Schizotypal Personality Questionnaire (SPQ) [53]	×	×

*scales were reversely scored, thus higher scores indicated lower self-esteem and well-being; for all other measures higher score indicates more psychopathology

For peer review only - http://bmjopen.bmj.com/site/about/guidelines.xhtml

Figures' legends:

Figure 1: Odds ratio in logistic regressions for suicidal thoughts (ST) and non-suicidal self-harm (NSSI) as outcomes predicted by psychopathological predictors (listed on the left) here treated as continuous variables; regressions were computed separately for each predictor and effects of age and sex were controlled in each regression for in both cohorts (see Supplementary Table 2).

Figure 2: Upper panel shows the dose-response effect of Common Mental Distress on non-suicidal self-harm (NSSI) and suicidal thought (ST) in Cohort 1 and Cohort 2. The lower panel shows the proportion of total reports in non-suicidal self-injury (NSSI) and suicidal thought (ST) broken down by standard deviations of Common Mental Distress; these add up to 100% from left to right. The normal population distribution of CMD, which was strikingly similar, but not identical, in Cohort 1 and 2, is shown by the purple line (see density plots in Supplement, Figure 1).

Figure 3: Mediation effect of Common Mental Distress at time 2 in Cohort 2: Standardised pathway coefficients with confidence intervals in square brackets.

For peer review only

For peer review only - http://bmjopen.bmj.com/site/about/guidelines.xhtml

References:

1. Hawton K, Saunders EA, O'Connor R. Self-harm and suicide in adolescents. *Lancet.* 2012;379:2373-2382.
2. Kidger J, Heron J, Lewis G, Evans J, Gunnell D. Adolescent self-harm and suicidal thoughts in the ALSPAC cohort: A self-report survey in England. *BMC Psychiatry.* 2012;12:1-12.
3. Nock MK. Future directions for the study of suicide and self-injury. J Clin Child Adolesc Psychol. 2012;41:255-259.
4. Scott LN, Pilkonis PA, Hipwell AE, Keenan K, Stepp SD. Non-suicidal self-injury and suicidal ideation as predictors of suicide attempts in adolescent girls: A multi-wave prospective study. *Compr Psychiatry.* 2015;58:1-10.
5. Ribeiro JD, Franklin JC, Fox KR, et al. Self-injurious thoughts and behaviors as risk factors for future suicide ideation, attempts, and death: A meta-analysis of longitudinal studies. *Psychol Med.* 2016;46:225-236.
6. Victor SE, Klonsky ED. Correlates of suicide attempts among self-injurers: A meta-analysis. *Clin Psychol Rev.* 2014;34(4):282-297.
7. Cooper J, Kapur N, Webb R, et al. Suicide after deliberate self-harm: a 4-year cohort study. *Am J Psychiatry.* 2005;162:297-303.
8. Patton GC, Coffey C, Sawyer SM, et al. Global patterns of mortality in young people: a systematic analysis of population health data. *Lancet*. 2009;374:881-892.
9. Muehlenkamp JJ, Claes L, Havertape L, Plener PL. International prevalence of adolescent non-suicidal self-injury and deliberate self-harm. *Child Adolesc Psychiatry Ment Health.* 2012;6(10).
10. Brunner R, Parzer P, Haffner J, et al. Prevalence and psychological correlates of occasional and repetitive deliberate self-harm in adolescents. *Arch Pediatr Adolesc Med.* 2007;161(7):641-649.
11. Bridge JA, Goldstein TR, Brent DA. Adolescent suicide and suicidal behavior. *J Child Psychol Psychiatry*. 2006;47:372-394.
12. Evans E, Hawton K, Rodham K, Deeks J. The prevalence of suicidal phenomena in adolescents: A systematic review of population-based study. *Suicide Life Threat Behav.* 2005;35(3):239-50.
13. Nielssen O, Wallace D, Large M. Pokorny's complaint: the insoluble problem of the overwhelming number of false positives generated by suicide risk assessment. *BJPsych Bulletin.* 2017;41:18-20.
14. Quinlivan L, Cooper J, Davies L, et al. Which are the most useful scales for predicting repeat self-harm? A systematic review evaluating risk scales using measures of diagnostic accuracy. *BMJ Open.* 2016;6(2): https://bmjopen.bmj.com/content/6/2/e009297.
15. Quinlivan L, Jayne Cooper J, Meehan D, et al. Predictive accuracy of risk scales following self-harm: multicentre, prospective cohort study. *Br J Psychiatry.* 2017;210:429-436.
16. Pokorny AD. Prediction of suicide in psychiatric patients. Report of a prospective study. *Arch Gen Psychiatry.* 1983;40:249-257.
17. Shaffer D, Gould MS, Fisher P, et al. Psychiatric diagnosis in child and adolescent suicide. *Arch Gen Psychiatry.* 1996;53:339-348.
18. Berona J, Horwitz AG, Czyz EK, King CA. Psychopathology profiles of acutely suicidal adolescents: Associations with post-discharge suicide attempts and rehospitalisation. *J Affect Disord*. 2017;209:97-104.
19. Nock MK, Joiner TE, Gordon KH, Lloyd-Richardson E, Prinstein MJ. Non-suicidal self-injury among adolescents: diagnostic correlates and relation to suicide attempts. *Psychiatry Res*. 2006;144(1):65-72.
20. Beckman K, Mittendorfer-Rutz E, Lichtenstein P, et al. Mental illness and suicide after self-harm among young adults: long-term follow-up of self-harm patients, admitted to hospital care, in a national cohort. *Psychol Med.* 2016;46:3397-3405.

For peer review only - http://bmjopen.bmj.com/site/about/guidelines.xhtml

21. Windfuhr K, While D, Kapur N, et al. Suicide risk linked with clinical consultation frequency, psychiatric diagnoses and psychotropic medication prescribing in a national study of primary-care patients. *Psychol Med.* 2016;46:3407-3417.
22. Caspi A, Houts RM, Belsky DW, et al. The p factor: One General Psychopathology Factor in the structure of psychiatric disorders? *Clin Psychol Sci.* 2014;2:119-137.
23. Patalay P, Fonagy P, Deighton J, et al. A general psychopathology factor in early adolescence. *The Br J Psychiatry.* 2015;207:15-22.
24. Stochl J, Khandaker GM, Lewis G, et al. Mood, anxiety and psychotic phenomena measure a common psychopathological factor. *Psychol Med.* 2015;45:1483-1493.
25. St Clair CM, Neufeld S, Jones BP, et al. Characterising the latent structure and organisation of self-reported thoughts, feelings and behaviours in adolescents and young adults. *PLOS* One. 2017; 12(4), 1-27. doi: https://doi.org/10.1371/journal.pone.0175381
26. Krueger RF. The structure of common mental disorders. *Arch Gen Psychiatry.* 1999;56:921-926.
27. Barch DM. The Neural Correlates of Transdiagnostic Dimensions of Psychopathology. *Am J Psychiatry*. 2017;174:613-615.
28. McTeague LM, Huemer J, Carreon DM, et al. Identification of Common Neural Circuit Disruptions in Cognitive Control Across Psychiatric Disorders. *Am J Psychiatry*. 2017;174:676-685.
29. Sharma A, Wolf DH, Ciric R, et al. Common Dimensional Reward Deficits Across Mood and Psychotic Disorders: A Connectome-Wide Association Study. *Am J Psychiatry.* 2017;174:657-666.
30. Rose G, Khaw M, Marmot G, Kay-Tee K, Marmot M. Chapter 3: The relation of risk to exposure. In: *Rose's strategy of preventive medicine (New ed.)* Oxford, Oxford University Press: 2008.
31. Kiddle B, Inkster B, Prabhu G, et al. The NSPN 2400 Cohort: a developmental sample supporting the Wellcome Trust NeuroScience in Psychiatry Network. *Int J Epidemiol.* 2018;47(1):18-19g.
32. Goodyer IM, Croudace T, Dunn V, Herbert J, Jones BP. Cohort Profile: Risk patterns and processes for psychopathology emerging during adolescence: the ROOTS project. *Int J Epidemiol.* 2010;39:361-369.
33. Office for National Statistics. Ethnicity and National Identity in England and Wales. 2012. Retrieved on 6th Feb 2018 from: https://www.ons.gov.uk/peoplepopulationandcommunity/culturalidentity/ethnicity/articles/ethnicityandnationalidentityinenglandandwales/2012-12-11#ethnicity-in-england-and-wales.
34. Noble M, McLennan, D, Wilkinson K, Whitworth A, & Barne H. The English Indices of Deprivation 2007. London: Department for Communities and Local Government. (2008).
35. King RA. Adolescent suicidal thoughts/behaviors are a marker of long-term vulnerability to poor adult outcomes. *J Am Acad Child Adolesc Psychiatry.* 2017;56:920-921.
36. Kelleher I, Keeley H, Corcoran P, et al. Clinicopathological significance of psychotic experiences in non-psychotic young people: evidence from four population-based studies. *Br J Psychiatry*. 2012;201:26-32.
37. Honings S, Drukker M, Groen R, van Os J. Psychotic experiences and risk of self-injurious behaviour in the general population: a systematic review and meta-analysis. *Psychol Med.* 2016;46:237-251.
38. Kelleher I, Corcoran P, Keeley H, et al. Symptoms and population risk for suicide attempt: A prospective cohort study. *JAMA Psychiatry.* 2013;70:940-948.
39. Lahey BB, Krueger RF, Rathouz PJ, Waldman ID, Zald DH. A hierarchical causal taxonomy of psychopathology across the life span. *Psychol Bull.* 2017;143:142-186.
40. Foulds GA, Bedford A. Hierarchy of classes of personal illness. *Psychol Med.* 1975;5:181-192.
41. Sturt E. Hierarchical patterns in the distribution of psychiatric symptoms. *Psychol Med.* 1981;11:783-792.

For peer review only - http://bmjopen.bmj.com/site/about/guidelines.xhtml

42. Kotov R, Krueger RF, Watson D. A paradigm shift in psychiatric classification: the Hierarchical Taxonomy Of Psychopathology (HiTOP). *World Psychiatry.* 2018;17:24-25.
43. Zald DH, Lahey BB. Implications of the Hierarchical Structure of Psychopathology for Psychiatric Neuroimaging. *Biol Psychiatry Cogn Neurosci Neuroimaging.* 2017;2:310-317.
44. Carver CS, Johnson SL, Timpano KR. Toward a functional view of the p factor in psychopathology. *Clinical Psychological Scienc.* 2017;5(5):880-889.
45. Fried EI, & Nesse RM. Depression sum-scores don't add up: why analyzing specific depression symptoms is essential. BMC Medicine. 2015; 13:72; https://bmcmedicine.biomedcentral.com/track/pdf/10.1186/s12916-015-0325-4
46. Franklin JC, Ribeiro JD, Fox KR, et al. Risk factors for suicidal thoughts and behaviors: A meta-analysis of 50 years of research. *Psychol Bull.* 2017;143(2):187-232.
47. Lewis G, Hawton K, Jones PB. Strategies for preventing suicide. *British Joournal of Psychiatry.* 1997;171:351-354.
48. Galante J, Dufour G, Vainre M, et al. A mindfulness-based intervention to increase resilience to stress in university students (the Mindful Student Study): a pragmatic randomised controlled trial. Lancet Public Health. 2018 Feb;3(2):e72-e81.
49. Wasserman D, Hoven CW, Wasserman C, et al. School-based suicide prevention programmes: the SEYLE cluster-randomised, controlled trial. *Lancet*. 2015;385:1536-1544.
50. Hammerton G, Zammit S, Potter R, Thapar A, Collishaw S. Validation of a composite of suicide items from the Mood and Feelings Questionnaire (MFQ) in offspring of recurrently depressed parents. *Psychiatry Res.* 2014;216:82-88.
51. Reynolds CR. Concurrent validity of what I think and feel: The revised children's manifest anxiety scale. J Consult Clin Psychol. 1980;48:774-775.
52. Bamber, D., Tamplin, A., Park, R.J., Kyte, Z.A. & Goodyer, I.M. Development of a short Leyton Obsessional Inventory For Children and Adolescents. *Journal of the American Academy of Child and Adolescent Psychiatry* **41**, 1246-1252 (2002).
53. Raine A. The SPQ: A scale for the assessment of schizotypal personality based on DSM-III-R criteria. *Schizophr Bull.* 1991;17:555-564.
54. Shaffer D, Fisher P, Lucas CP, Dulcan MK, Schwab-Stone ME. NIMH Diagnostic Interview Schedule for Children Version IV (NIMH DISC-IV): description, differences from previous versions, and reliability of some common diagnoses. *J Am Acad Child Adolesc Psychiatry.* 2000;39:28-38.
55. Rosenberg M. *Society and the adolescent self-image*. Princeton, NJ: Princeton University Press; 1965.
56. Tennant R, Hiller L, Fishwick R, et al. The Warwick-Edinburgh mental well-being scale (WEMWBS): development and UK validation. Health Qual Life Outcomes. 2007;5:63.
57. Patton JH, Stanford MS, Barratt ES. Factor structure of the Barratt Impulsiveness Scale. *J Soc Clin Psychol.* 1995;51:768-774.
58. Poythress NG, Poythress NG, Douglas KS, Falkenbach D, et al. Internal consistency reliability of the self-report antisocial process screening device. *Asmnt.* 2006;13:107-113.

For peer review only - http://bmjopen.bmj.com/site/about/guidelines.xhtml

Authors contributions:

E.P. – conceptualised the study, computed statistical analyses and drafted the manuscript

P.B.J. – provided senior supervision, conceptualised the study, advised on statistical analyses, read and critically apprised the manuscript, re-drafted and edited the manuscript

J.S. – provided statistical advice, replicated multiple imputations, provided data from multiple imputations, read and critically apprised the manuscript

S.N. – advised on handling missing data, replicated multiple imputations, read and critically apprised the manuscript

P.W. – read and critically apprised the manuscript, provided key referred articles

R.D. – read and critically apprised the manuscript, provided key referred articles

I.M.G. – read and critically apprised the manuscript, provided key referred articles

E.B. – read and critically apprised the manuscript, provided key referred articles

P.F. – read and critically apprised the manuscript, provided key referred articles

R.D. – read and critically apprised the manuscript

M.C.S.C. – contributed to data collection and project management, provided advise on bifactor modelling

G.P. – contributed to data collection and project management

For peer review only

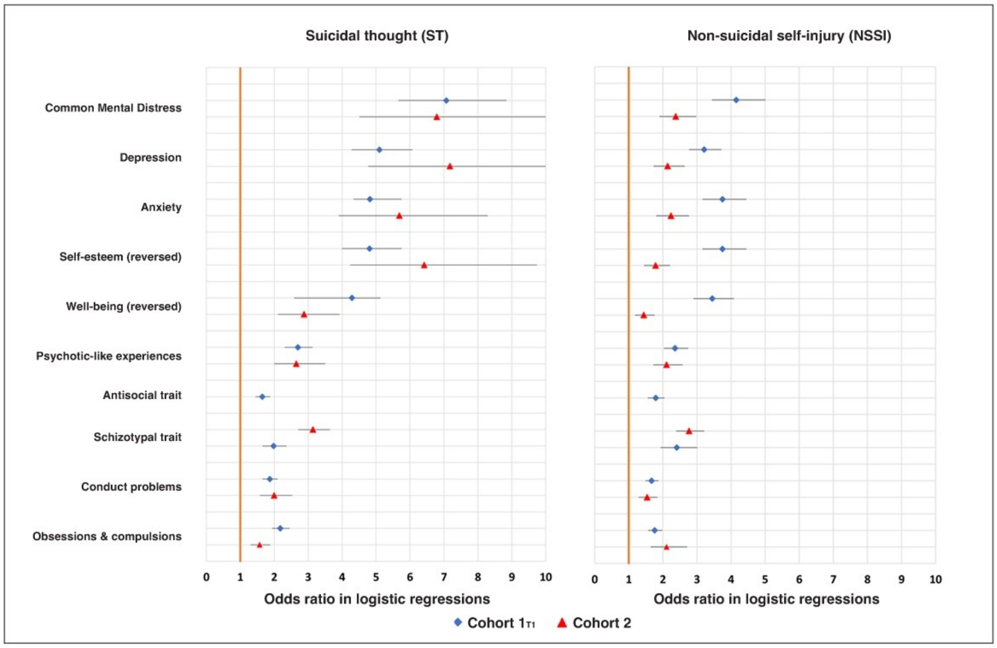

123x80mm (240 x 240 DPI)

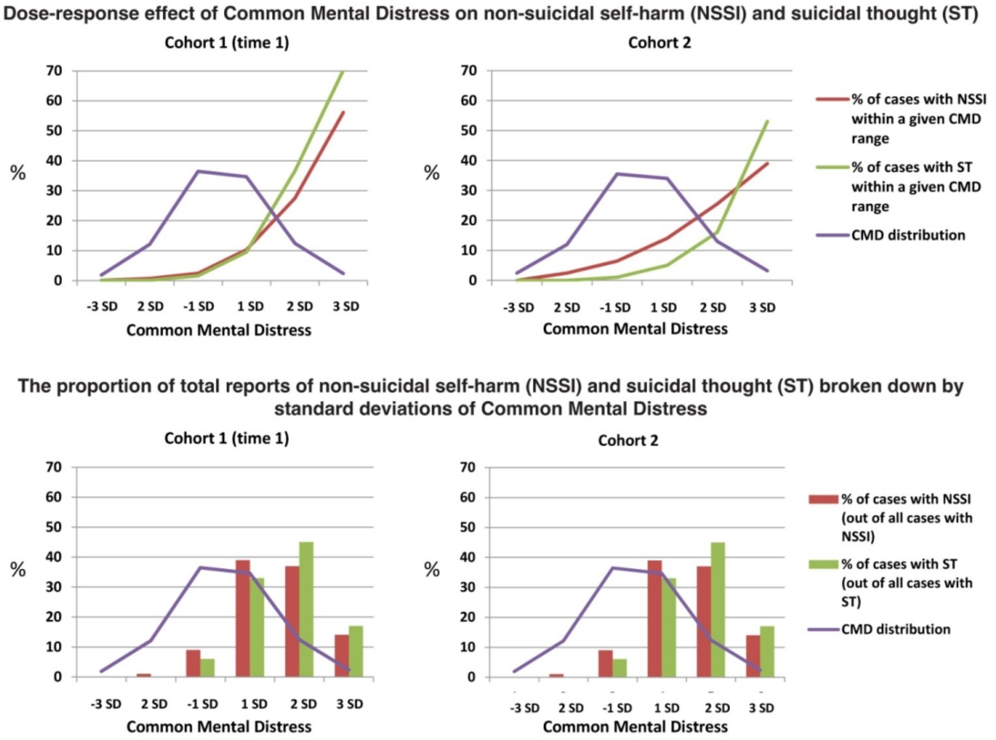

175x132mm (220 x 220 DPI)

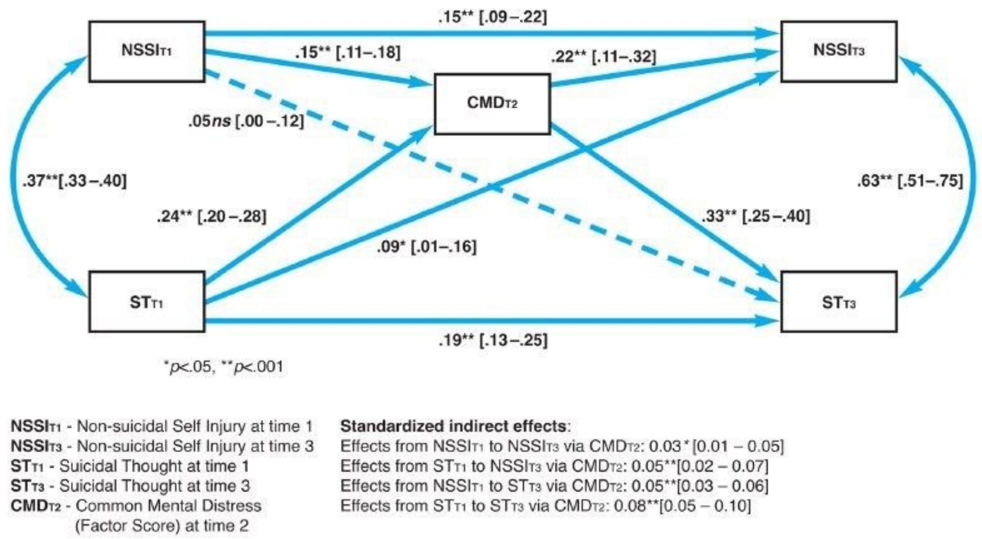

211x115mm (96 x 96 DPI)

Bifactor modelling:

Bifactor psychometric modelling is designed to extract variance common for all items in the model to generate one “general” factor. In addition to this general factor, specific factor/s may emerge, which are uncorrelated with each other or with the general factor. Specific factor/s contain the remaining variance after the extraction of the general factor[1]. St Clair et al. (2017) found in her psychometric study a bifactor model with one general factor and 5 specific factors, which fitted the data better than the correlated-factors model or second-order model. In our study, we first replicated St Clair et al. (2017) psychometric model in Cohort 1 (T1, T2, T3) and Cohort 2. In accordance with the original study, in our psychometric modelling the same measures of common mental illness frequently emerging during adolescence (depression, anxiety, psychotic experiences, obsessions and compulsions, conduct problems) as well as traits and characteristics commonly considered to contribute to mental wellness (well-being, self-esteem) were used as constructs contributing the general factor (see items below). Having replicated St Clair et al (2017) bifactor model, we then computed factor scores for the general factor – here termed *Common Mental Distress (CMD)*.

The confirmatory bifactor analysis in Cohort 1 was computed with the multiple group method (MGM) in Mplus 8 with the three data point used as a grouping variable; the same model was fitted to the data in each group. MGM in Mplus by default holds thresholds and loadings invariant across groups[2], thus allowing the comparison if the model fits data well in all groups under study (here data from the three measurement points). The effective sample for the 3 data waves was, respectively, n=2403, n=1815, n=1245 (Total N=5463). The overall chi-square test for the model was $\chi2$=33648.24 (df=14983, p=0.000), for Time 1 it was $\chi2$=14791.20, for Time 2 it was $\chi2$=10400.56 and for Time 3 it was $\chi2$=8456.47. The overall Root Mean Square Error of Approximation (RMSEA) for the model was 0.026 (0.026-0.027), Comparative Fit Index (CFI) was 0.969, Tucker-Lewis Index (TLI) was 0.969, and weighted root mean square residual (WRMR) was 2.91. The confirmatory bifactor analysis was used in Cohort 2 as well. The following fit indexes were obtained in Cohort 2: $\chi2$=7602.17 (df=4462, p=0.000), RMSEA=0.026 (0.025-0.027), CFI=0.96, TLI=0.96, WRMR= 1.34. The above-cited fit indexes suggest that the bifactor model fitted the data well in both cohorts.

In both analyses – for Cohort 1 and 2 – we used WLSMV estimator and THETA parametrisation with PROBIT link, and all items were treated as ordered-categorical variables.

Much debate in the literature has focused on the issue of interpretability of specific factors, i.e., whether they should be considered as measures of meaningful concepts or should be treated as comprising the residual, uninterpretable variance[3]. The general factor in St Clair et al (2017) study demonstrated high reliability and validity, as well as low measurement error compared to validity and error of the specific factors. As follows, we focused in our study only on this general (CMD) factor; we did not attempt to interpret or use in our analyses the specific factors, even though they emerged in our bifactor modelling, due to their relatively high measurement error and ambiguity of their theoretical interpretation. The list of items contributing to CMD factor with factor loadings on this factor in Cohort 1 (T1, T2, T3) and Cohort 2 are listed below in Supplementary Table 1.

For peer review only - http://bmjopen.bmj.com/site/about/guidelines.xhtml

Multiple imputation procedure in Cohort 1:

Missingness in Cohort 1 predominantly arose from longitudinal attrition – 24% at T2 and 48% at T3; a small fraction of data was also missing due to omissions of items (between 0 to 6%). Before performing imputations, we examined if longitudinal attrition was related to demographic variables and other variables under study. Indeed, we found small, yet statistically significant correlations between attrition at T2 and T3 and demographic and exposure variables at T1 (see Supplementary Table 8), thus indicating that the assumption of "missing completely at random (MCAR) is not met. Moreover, we performed Little's MCAR test and found that it was significant ($p<.001$). Therefore, we assumed that MAR condition was met. As follows, we imputed missing data under MAR condition in Cohort 1 at T2 and T3 with the following variables in one imputation model: CMD factor scores, NSSI and ST variables. We used the following auxiliary variables: research points, sex, age, ethnicity, and Index of Multiple Deprivation (IMD) (as an indicator of a socioeconomic status[4]) as predictors of the missingness, in addition to main predictors – CMD factor scores, NSSI, and ST at T1.

Multiple imputations were computed in R program with MICE package[5]; convergence was examined by visual inspection of MCMC chains (with a maximum number of 20 iterations per chain and Gibbs sampling). Fifty-four (N=2403) datasets were generated to equal the percentage of missing data in CMD, NSSI, and ST at T3[6]. In terms of the imputation model, we used mean matching for continuous variables (CMD factor scores) and logistic regression for binary variables (NSSI and ST). The imputed 54 datasets were then used in pathway analysis (see the main manuscript and Supplementary Figure 3 for details) with MLM estimator in Mplus 7.4, which automates the process of analysing and combining parameter estimates from each imputed dataset using Rubin's rules[7].

For peer review only - http://bmjopen.bmj.com/site/about/guidelines.xhtml

Supplementary Table 1: List of all items used in the study

**Outcome measures:**
**Suicidal Thought (ST)**<br>*I thought about killing myself* (MFQ19, response options: *Always, Mostly, Sometimes, Never*)[Cohort 1 & 2]<br>This is one of the 4 items assessing suicidal thoughts in the 33-item Mood and Feelings Questionnaire (MFQ)[8] : MFQ16 - I thoughts that life was not worth living; MFQ17 - I thought about dying; MFQ18 – I thought my family would be better off without me; MFQ19 - I thought about killing myself. We used item 19, as it had the highest (.70) loading on this sub-subscale. Responses to this item were recoded into a binary format: no ST (original response option *Never*) and ST (original response options *Sometimes* or *Mostly* or *Always*). We did not include MFQ items 16-18 in CMD factor to avoid content overlap between the outcome measure (ST) and the predictor – the CMD factor.<br><br>**Non-Suicidal Self-Injury (NSSI)**<br>NSSI in Cohort 1 was assessed with one question from the Drug, Alcohol and Self-Injury (DASI) questionnaire asking about engaging in self-injury without suicidal intent during the last month:<br>*In the last month, have you tried to hurt yourself on purpose without trying to kill yourself*? (Response options: *Yes, No)*<br><br>NSSI in Cohort 2 was assessed with one question from the DASI questionnaire asking about life-time occurrence of NSSI:<br>[illegible]

Supplementary Table 9: Items comprising the Common Mental Distress (CMD) factor

**Items and associated measures**	**Standardised Factor Loadings**			
**The Moods and Feelings Questionnaire (MFQ)**[11 Cohort 1 & 2]<br>**(response options: *Always, Mostly, Sometimes, Never*)**<br>*Note:* 4 items measuring suicidality were excluded to avoid content overlap between the measures of variables treated here as predictors (CMD, Depression) and the outcome variable (ST). We excluded 4 other items which caused model convergence problems: *I was less hungry than usual (MFQ3), I ate more than usual (MFQ4), It was hard for me to make up my mind (MFQ10), I slept a lot more than usual (MFQ33)*	**Cohort 1**			**Cohort 2**
	**Time 1**	**Time 2**	**Time 3**	
1. I felt miserable or unhappy. (MFQ1)	.69	.73	.71	.73
2. I didn't enjoy anything. (MFQ2)	.62	.70	.72	.67
3. I felt so tired I just sat around and did nothing. (MFQ5)	.53	.56	.57	.54

For peer review only - http://bmjopen.bmj.com/site/about/guidelines.xhtml

For peer review only

4. I was moving and walking more slowly than usual. (MFQ6)	.54	.59	.54	.52
5. I was very restless. (MFQ7)	.48	.54	.56	.49
6. I felt I was no good any more. (MFQ8)	.78	.82	.84	.77
7. I sometimes blamed myself for things that weren't my fault. (MFQ9)	.70	.74	.75	.73
8. I got grumpy and cross easily. (MFQ11)	.60	.65	.68	.65
9. I felt like talking a lot less than usual. (MFQ12)	.64	.66	.69	.65
10. I was talking more slowly than usual. (MFQ13)	.56	.64	.55	.59
11. I cried a lot. (MFQ14)	.64	.64	.68	.69
12. I thought there was nothing good for me in the future. (MFQ15)	.72	.77	.78	.72
13. I didn't want to see my friends. (MFQ20)	.69	.73	.70	.66
14. I found it hard to think properly or concentrate. (MFQ21)	.73	.77	.77	.72
15. I thought bad things would happen to me. (MFQ22)	.76	.77	.80	.81
16. I hated myself. (MFQ23)	.81	.82	.85	.80
17. I was a bad person. (MFQ24)	.73	.76	.78	.72
18. I thought I looked ugly. (MFQ25)	.65	.70	.70	.69
19. I worried about aches and pains. (MFQ26)	.46	.50	.50	.56
20. I felt lonely. (MFQ27)	.70	.74	.73	.74
21. I thought nobody really loved me. (MFQ28)	.75	.79	.83	.76
22. I didn't have any fun at school / college / work. (MFQ29)	.62	.67	.66	.58
23. I thought I could never be as good as other people my age. (MFQ30)	.76	.79	.78	.76
24. I did everything wrong. (MFQ31)	.83	.85	.87	.82
25. I didn't sleep as well as usual. (MFQ32)	.53	.57	.61	.60
**The Revised Children's Manifest Anxiety Scale (RCMAS)**[12] **Cohort 1 & 2** **(response options:** ***Always, Mostly, Sometimes, Never*****)**				
1. I had trouble making up my mind. (RCMAS1)	.60	.68	.71	.59
2. I worried when things did not go the right way for me. (RCMAS2)	.71	.77	.79	.78
3. Others seemed to do things more easily than I could. (RCMAS3)	.76	.80	.83	.76
4. Often I had trouble getting a breath. (RCMAS4)	.56	.60	.59	.55
5. I worried a lot of the time. (RCMAS5)	.78	.80	.82	.78
6. I was afraid of a lot of things. (RCMAS6)	.78	.80	.82	.77
7. I got angry easily. (RCMAS7)	.63	.68	.74	.68

For peer review only

8. I worried about what my parents would say to me. (RCMAS8)	.62	.67	.71	.65
9. I felt that others did not like the way I did things. (RCMAS9)	.73	.79	.78	.74
10. It was hard for me to get to sleep at night. (RCMAS10)	.55	.63	.58	.57
11. I worried about what other people thought about me. (RCMAS11)	.74	.79	.80	.71
12. I felt alone even when there were people with me. (RCMAS12)	.80	.84	.86	.85
13. Often I felt sick to my stomach. (RCMAS13)	.69	.74	.74	.76
16. I was tired a lot. (RCMAS16)	.62	.67	.69	.65
17. I worried about what was going to happen. (RCMAS17)	.77	.80	.81	.79
18. Other people my age were happier than me. (RCMAS18)	.79	.83	.83	.79
19. I had bad dreams. (RCMAS19)	.54	.59	.57	.62
20. My feelings got hurt easily when I was fussed at. (RCMAS20)	.75	.76	.78	.77
21. I felt someone would tell me I did things the wrong way. (RCMAS21)	.70	.77	.77	.71
22. I wake up scared some of the time. (RCMAS22)	.64	.74	.72	.67
23. I worried when I went to bed at night. (RCMAS23)	.67	.74	.73	.75
24. It was hard for me to keep my mind on my work. (RCMAS24)	.48	.58	.56	.55
25. I wiggled in my seat a lot. (RCMAS25)	.77	.79	.80	.76
27. A lot of people were against me. (RCMAS27)	.75	.80	.83	.80
28. I often worried about something bad happening to me. (RCMAS28)	.74	.79	.79	.80
**The Revised Leyton Obsessional Inventory (R-LOI)[13] Cohort 1 & 2**<br>**(response options: *Always, Mostly, Sometimes, Never*)**				
1. I felt I had to do things in a certain way, like counting or saying special words, to stop something bad from happening. (R-LOI1)	.53	.58	.50	.47
2. I had trouble finishing my homework or other jobs because I had to do things over and over again. (R-LOI2)	.58	.63	.64	.53
3. I hated dirt and dirty things. (R-LOI3)	.35	.44	.43	.39
4. I had a special number that I counted up to, or I felt I had to do things just that number of times. (R-LOI4)	.40	.46	.42	.41
5. I often felt guilty or bad about things I had done even though no one else thought I had done anything wrong. (R-LOI5)	.71	.77	.79	.73
6. I worried about being clean enough. (R-LOI6)	.48	.51	.55	.45

For peer review only - http://bmjopen.bmj.com/site/about/guidelines.xhtml

7. I moved or talked in a special way to avoid bad luck. (R-LOI7)	.38	.46	.38	.33
8. I worried a lot if I did something, not exactly the way I liked. (R-LOI8)	.60	.67	.66	.53
9. I was fussy about keeping my hands clean. (R-LOI9)	.35	.40	.41	.35
10. I had special numbers or words that I said because I hoped they kept bad luck or bad things away. (R-LOI10)	.43	.47	.47	.42

**Antisocial Behaviour Questionnaire (ABQ)**[14] **Cohort 1 & 2** **(response options: *Always, Mostly, Sometimes, Never*)**				
1. I deliberately broke the rules or disobeyed people (e.g. parents, teachers or supervisors). (ABQ1)	.45	.48	.47	.38
2. I stole things (e.g. from home or a shop or school). (ABQ2)	.37	.40	.36	.26
3. I deliberately damaged property (e.g. broke windows or chairs or wrote graffiti or started fires). (ABQ3)	.35	.39	.39	.38
4. I skipped lessons/work, skived, or played truant from school. (ABQ5)	.36	.39	.40	.35
5. I deliberately lied or cheated to get what I wanted. (ABQ6)	.43	.39	.41	.40
6. I ran away from home (e.g. for half a day or overnight). (ABQ7)	.51	.56	.58	.56
**Rosenberg Self-Esteem Questionnaire (RSEQ)**[15] **Cohort 1 & 2** **(response options: *Always, Mostly, Sometimes, Never*)**				
1. At times, I thought I was no good at all. (RSEQ1)	.82	.84	.85	.83
2. I was satisfied with myself. (RSEQ2)	-.58	-.61	-.60	-.53
3. I felt I had a number of good qualities. (RSEQ3)	-.53	-.55	-.56	-.52
4. I was able to do things as well as most people. (RSEQ4)	-.56	-.60	-.62	-.56
5. I felt I did not have much to be proud of. (RSEQ5)	.70	.73	.72	.70
6. I certainly felt useless at times. (RSEQ6)	.79	.81	.79	.77
7. I felt that I was as good as anyone else. (RSEQ7)	-.53	-.56	-.54	-.44
8. I wished I could have more respect for myself. (RSEQ8)	.62	.66	.68	.69
9. I felt that I was a failure. (RSEQ9)	.80	.82	.83	.75
10. I took a positive attitude toward myself. (RSEQ10)	-.60	-.63	-.63	-.56
11. I kept thinking about the things that I had done because I wasn't sure that they were the right things to do. (R-LOI11)	.71	.73	.71	.67

For peer review only - http://bmjopen.bmj.com/site/about/guidelines.xhtml

**Warwick-Edinburgh Mental Well-Being Scale (WEMWBS)**[16] **Cohort 1 & 2**<br>**(response options: *None of the time, Rarely, Some of the time, Often, All of the time*)**				
1. I've been feeling optimistic about the future. (WEMWBS1)	-.46	-.51	-.54	-.25
2. I've been feeling useful. (WEMWBS2)	-.52	-.58	-.60	-.33
3. I've been feeling relaxed. (WEMWBS3)	-.57	-.62	-.63	-.49
4. I've had the energy to spare. (WEMWBS5)	-.40	-.46	-.49	-.36
5. I've been dealing with problems well. (WEMWBS6)	-.57	-.63	-.64	-.46
6. I've been thinking clearly. (WEMWBS7)	-.62	-.67	-.68	-.48
7. I've been feeling good about myself. (WEMWBS8)	-.65	-.71	-.70	-.55
8. I've been feeling close to other people. (WEMWBS9)	-.44	-.50	-.52	-.28
9. I've been feeling confident. (WEMWBS10)	-.58	-.63	-.66	-.46
10. I've been able to make up my own mind about things. (WEMWBS11)	-.52	-.59	-.60	-.39
11. I've been feeling loved. (WEMWBS12)	-.49	-.54	-.60	-.29
12. I've been interested in new things. (WEMWBS13)	-.36	-.45	-.46	-.20
13. I've been feeling cheerful. (WEMWBS14)	-.61	-.67	-.67	-.49
**Psychotic-Like Experiences:**<br>**Cohort 1 – selected 10 items from the Schizotypal Personality Questionnaire (SPQ)**[17]<br>**Cohort 2 – selected 7 items from the Diagnostic Interview Schedule for Children (DISC)**[18]<br>**(response options: *Yes, No*)**				
1. Have you often mistaken objects or shadows for people or noises for voices? (SPQ4) **Cohort 1**	.38	.43	.41	*Not used*
2. I am sure I am being talked about behind my back. (SPQ9, DISC3) **Cohort 1 & 2**	.59	.67	.66	.60
3. Have you ever had the sense that some person or force is around you, even though you cannot see anyone? (SPQ13, DISC5) **Cohort 1 & 2**	.33	.38	.34	.41
4. Have you ever noticed a common event or object that seemed to be a special sign for you? (SPQ28, DISC8) **Cohort 1 & 2**	.33	.33	.35	.38
5. I often hear a voice speaking my thoughts aloud. (SPQ31, DISC10) **Cohort 1 & 2**	.33	.39	.34	.40
6. Have you ever seen things invisible to other people? (SPQ40, DISC13) **Cohort 1 & 2**	.36	.50	.37	.48
7. Do you sometimes feel that other people are watching you? (SPQ60, DISC19) **Cohort 1 & 2**	.53	.55	.59	.54
8. Do you ever suddenly feel distracted by distant sounds that you are not normally aware of? (SPQ61) **Cohort 1**	.40	.49	.45	*Not used*

For peer review only - http://bmjopen.bmj.com/site/about/guidelines.xhtml

9. Do you sometimes feel that people are talking about you? (SPQ63, DISC15) [Cohort 1 & 2]	.52	.56	.59	.60
10. Are your thoughts sometimes so strong that you can almost hear them? (SPQ64) [Cohort 1]	.44	.52	.50	*Not used*

For peer review only

Supplementary Table 2: Predictive power of Common Mental Distress versus the conventional psychopathology dimensions in Cohort 1_{T1} and Cohort 2: AUC (for ST and NSSI as criteria) and ORs for continuous and binary predictors (with cut-off point of 1SD)

		AUC		Suicidal thought (ST)				Non-suicidal self-injury (NSSI)			
				Continuous predictor		Binary (1SD cut-off)		Continuous predictor		Binary (1SD cut-off)	
		ST	NSSI	OR	95% C.I.	OR	95% C.I.	OR	95% C.I.	OR	95% C.I.
Common Mental Distress	Cohort 1_{T1}	.87	.83	7.07	[5.66 - 8.84]	15.60	[11.56 - 21.06]	4.15	[3.44 - 5.01]	8.93	[6.63 - 12.03]
	Cohort 2	.88	.72	6.79	[4.51 - 10.21]	20.97	[6.47 - 67.92]	2.38	[1.90 - 2.98]	4.00	[2.55 - 6.28]
Depression	Cohort 1_{T1}	.88	.83	5.10	[4.28 - 6.07]	15.60	[11.56 - 21.06]	3.21	[2.77 - 3.72]	8.28	[6.15 - 11.14]
	Cohort 2	.88	.70	7.18	[4.77 - 10.80]	15.32	[8.52 - 27.57]	2.14	[1.73 - 2.64]	3.56	[2.32 - 5.46]
Anxiety	Cohort 1_{T1}	.85	.81	4.82	[4.04 - 5.75]	13.62	[10.11 - 18.34]	3.75	[3.16 - 4.45]	7.61	[5.67 - 10.22]
	Cohort 2	.86	.71	5.69	[3.90 - 8.29]	10.51	[5.89 - 18.73]	2.24	[1.81 - 2.77]	3.68	[2.39 - 5.67]
Self-esteem (reversed)	Cohort 1_{T1}	.85	.83	4.81	[4.00 - 5.79]	15.62	[11.49 - 21.23]	3.75	[3.16 - 4.45]	9.86	[7.28 - 13.35]
	Cohort 2	.87	.65	6.42	[4.24 - 9.74]	15.16	[8.32 - 27.62]	1.79	[1.45 - 2.21]	3.34	[2.20 - 5.07]
Well-being (reversed)	Cohort 1_{T1}	.82	.80	4.29	[3.59 - 5.13]	10.31	[8.06 - 13.19]	3.45	[2.90 - 4.09]	6.66	[4.93 - 8.99]
	Cohort 2	.78	.61	2.88	[2.11 - 3.93]	5.27	[3.01 - 9.24]	1.44	[1.18 - 1.76]	2.19	[1.40 - 3.42]
Psychotic-like experiences	Cohort 1_{T1}	.74	.73	2.70	[2.32 - 3.13]	4.94	[3.70 - 6.60]	2.36	[2.03 - 2.74]	4.03	[2.98 - 5.45]
	Cohort 2	.74	.71	2.65	[2.00 - 3.50]	6.78	[3.89 - 11.83]	2.11	[1.72 - 2.58]	4.11	[2.69 - 6.27]
Antisocial trait*	Cohort 1_{T1}	.64	.63	1.65	[1.45 - 1.88]	2.67	[1.96 - 3.63]	1.79	[1.56 - 2.05]	2.48	[1.78 - 3.47]
Schizotypal trait	Cohort 1_{T1}	.79	.78	3.14	[2.71 - 3.64]	6.26	[4.70 - 8.32]	2.77	[2.39 - 3.21]	6.08	[4.52 - 8.19]
	Cohort 2	.76	.72	1.98	[1.66 - 2.36]	5.66	[3.23 - 9.91]	2.41	[1.93 - 3.01]	4.45	[2.90 - 6.83]
Conduct problems	Cohort 1_{T1}	.69	.67	1.87	[1.66 - 2.10]	3.38	[2.52 - 4.52]	1.67	[1.49 - 1.87]	3.46	[2.54 - 4.71]
	Cohort 2	.68	.61	2.00	[1.58 - 2.53]	3.78	[2.16 - 6.63]	1.54	[1.29 - 1.84]	2.13	[1.36 - 3.34]
Obsessions & compulsions	Cohort 1_{T1}	.76	.72	2.18	[1.94 - 2.45]	5.74	[4.25 - 7.75]	1.76	[1.57 - 1.98]	3.55	[2.58 - 4.89]
	Cohort 2	.71	.63	1.57	[1.31 - 1.88]	4.16	[2.37 - 7.28]	2.11	[1.64 - 2.71]	2.75	[1.79 - 4.22]

** measures were available only for Cohort 1_{T1}*

For peer review only

For peer review only - http://bmjopen.bmj.com/site/about/guidelines.xhtml

Supplementary Table 3: Association between ST and demographic variables in Cohort 1 (T1, T2, T3) and Cohort 2 (polychoric correlations)

	ST Cohort 1			ST Cohort 2
	T1	T2	T3	
Socioeconomic status (IMD)	-.05	-.01	-.01	.02
Research centre (0-Cambridge, 1-London)	-.12	-.04	-.03	not applicable
Ethnicity (1-white; 0-other)	-.08	-.02	-.04	-0.01
Age	-.05	-.02	-.05	0.03
Gender (0-Female, 1-Male)	-.10	-.08	-.01	0.03

All p-values non-significant

Supplementary Table 4: Association between NSSI and demographic variables in Cohort 1 (T1, T2, T3) and Cohort 2 (polychoric correlations)

	Cohort 1			Cohort 2
	T1	T2	T3	
Socioeconomic status (IMD)	.00	.00	.02	-.01
Research centre (0-Cambridge, 1-London)	-.01	-.01	.00	not applicable
Ethnicity (1-white; 0-other)	.00	.00	.00	.00
Age	-.02	-.04	-.01	-.02
Gender (0-Female, 1-Male)	.05	-.23	.02	.08

All p-values non-significant

Supplementary Table 5: Association between CMD and demographic variables in Cohort 1 (T1, T2, T3) and Cohort 2 (polychoric correlations)

	Cohort 1			Cohort 2
	T1	T2	T3	
Socioeconomic status (IMD)	-.02	-.02	-.01	.02
Research centre (0-Cambridge, 1-London)	.07*	.01	.01	not applicable
Ethnicity (1-white; 0-other)	-.08**	-.04	-.04	.04
Age	.01	.01	.01	.01
Gender (0-Female, 1-Male)	-.15**	-.15*	-.11**	.20**

*$p<.01$, **$p<.001$

For peer review only - http://bmjopen.bmj.com/site/about/guidelines.xhtml

For peer review only

Supplementary Table 6: Test of change in the prevalence of NSSI in Cohort 1: frequency over three time points (chi-square test)

	T1	T2	T3
NSSI	223	199	197
No-NSSI	2180	2204	2206

Chi-square=2.22, *df*=2, *p*=0.32, Yates' chi-square =2.04, *p*=0.35

Supplementary Table 7: Test of change in the prevalence of ST in Cohort 1: frequency over three time points (chi-square test)

	T1	T2	T3
NSSI	243	274	281
No-NSSI	2160	2129	2122

Chi-square=3.45, *df*=2, *p*=0.17, Yates' chi-square =3.26, *p*=0.19

For peer review only

Supplementary Table 8: Association between attrition in Cohort 1 at T2 and T3 and other variables in the study (Spearman rho)

	**Attrition Cohort 1**	
T1 variables:	**T2**	**T3**
Socioeconomic status (IMD index)[#]	-.07**	-.05*
Research centre (0-Cambridge, 1-London)	.05*	.05*
Ethnicity (1-white; 0-other)	-.05*	-.05*
Age	.07**	.05*
Gender (0-Female, 1-Male)	.09**	.12**
NSSI	-.01	.00
ST	-.01	-.03
Common Mental Distress	.06*	.05*
Depression	.06**	.05*
Impulsivity	.10**	.14**
Anxiety	.04*	.04*
Self - esteem (reversed)	.07**	.06*
Well - being (reversed)	.06*	.05*
Psychotic - like experiences even coerced	.00	.01
Antisocial trait	.08**	.12**
Schizotypal trait	.04*	.03
Conduct problems	.10**	.13**
Obsessions & compulsions	.03	.03

***p*<.001, **p*<.01
[#]higher number indicated *lower* socioeconomic deprivation

For peer review only

Supplementary Table 9: Direct and indirect effects in mediation (pathway) models in a female (F), male (M) and total (T) sample

		Standardised				Non- standardised			
		Coeff.	S.E.	Lower 95% C.I.	Upper 95% C.I.	Coeff.	S.E.	Lower 95% C.I.	Upper 95% C.I.
NSSI$_{T1}$->CMD$_{T2}$	F	.14***	.03	.09	.19	.46***	.09	.30	.61
	M	13***	.03	.07	.18	.56***	.14	.32	.80
	T	.15***	.02	.11	.18	.53***	.07	.40	.66
NSSI$_{T1}$->NSSI$_{T3}$	F	.16**	.05	.07	.25	.54**	.17	.25	.83
	M	.14**	.05	.05	.23	.65**	.24	.25	1.05
	T	.15**	.03	.09	.22	.58**	.14	.34	.82
NSSI$_{T1}$->ST$_{T3}$	F	.07	.05	.00	.16	.27	.17	-.01	.56
	M	.04	.05	-.03	.13	.22	.24	-.18	.62
	T	.05	.03	.00	.12	.22	.15	-.02	.47
ST$_{T1}$->CMD$_{T2}$	F	.25***	.03	.19	.30	.83***	.10	.66	1.00
	M	.24***	.03	.18	.30	.85***	.11	.65	1.05
	T	.24***	.02	.20	.28	.83***	.07	.70	.96
ST$_{T1}$->NSSI$_{T3}$	F	.10*	.05	.01	.20	.38	.19	.05	.70
	M	.07	.06	-.03	.17	.25	.23	-.13	.64
	T	.19*	.04	.13	.25	.33	.16	.06	.60
ST$_{T1}$->ST$_{T3}$	F	.20***	.04	.13	.27	.76***	.16	.49	1.03
	M	.17***	.05	.08	.25	.66***	.19	.33	.98
	T	.19**	.03	.13	.25	.72***	.13	.50	.95
CMD$_{T2}$-> NSSI$_{T3}$	F	.22***	.07	.11	.34	.24**	.07	.11	.37
	M	.21*	.08	.07	.34	.21*	.09	.06	.36
	T	.22***	.06	.11	.32	.22***	.06	.11	.34
CMD$_{T2}$-> ST$_{T3}$	F	.32***	.05	.22	.41	.35***	.07	.23	.47
	M	.35***	.06	.25	.46	.39***	.07	.26	.51
	T	.33***	.04	.25	.40	.35***	.05	.26	.45
NSS$_{T1}$<->ST$_{T1}$	F	.40***	.02	.36	.45	.04***	.00	.03	.04
	M	.32***	.03	.26	.37	.02***	.00	.01	.03
	T	.37***	.02	.33	.40	.03***	.00	.02	.03
NSSI$_{T3}$<->ST$_{T3}$	F	.67***	.07	.55	.79	.67***	.07	.55	.79
	M	.57***	.10	.39	.75	.57***	.10	.39	.75
	T	.63***	.07	.51	.75	.63***	.07	.51	.75
NSSI$_{T1}$->CMD$_{T2}$ ->NSSI$_{T3}$	F	.03**	.01	.01	.05	.11**	.04	.04	.18
	M	.02*	.01	.00	.05	.12*	.06	.02	.22

For peer review only

For peer review only - http://bmjopen.bmj.com/site/about/guidelines.xhtml

	T	.03*	.01	.01	.05	.12**	.04	.05	.19
ST_{T1}->CMD_{T2}->$NSSI_{T3}$	F	.05**	.02	.02	.09	.20**	.07	.08	.32
	M	.05*	.02	.01	.08	.18*	.08	.05	.31
	T	.05**	.01	.02	.07	.19***	.05	.09	.28
$NSSI_{T1}$->CMD_{T2}->ST_{T3}	F	.04***	.01	.02	.06	16***	.04	.08	.24
	M	.04***	.01	.02	.07	.22**	.07	.10	.33
	T	.05**	.01	.03	.06	.19***	.04	.12	.26
ST_{T1}->CMD_{T2}->ST_{T3}	F	.08***	.01	.05	.11	.29***	.07	.17	.41
	M	.08***	.02	.05	.12	.33***	.08	.20	.47
	T	.08**	.01	.05	.10	.30***	.05	.20	.39

Significance levels: *$p<.05$, **$p<.01$, ***$p<.001$

For peer review only

For peer review only - http://bmjopen.bmj.com/site/about/guidelines.xhtml

Item Response Theory (IRT) analysis

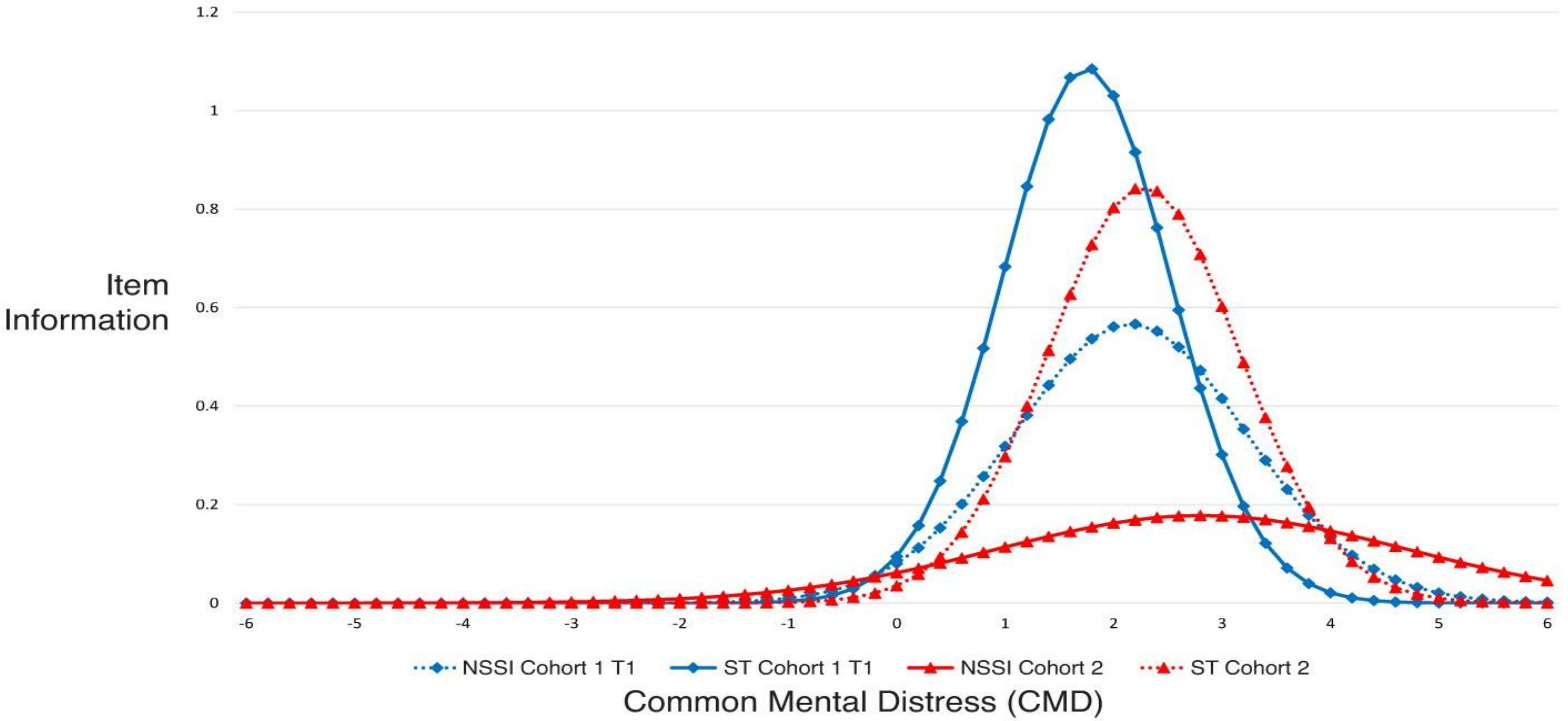

Supplementary Figure 1: Hierarchy of symptoms: the place of non-suicidal self-harm (NSSI) and suicidal though (ST) on the latent continuum of Common Mental Distress (in standard deviations) in Cohort 1_{T1} and Cohort 2.

Item Response Theory (IRT) analysis is concerned, broadly speaking, with investigating the relationship between items and the latent construct. Here we computed item response function showing how much information NSSI and ST (here treated as indicators of CMD) contribute to the latent variable – CMD. The above graph shows that NSSI and ST provided information in above-average to high ranges of CMD, with the peak of the information curves for NSSI occurring around +2 SD in both cohorts. The information curve for ST in Cohort 2 was flatter, suggesting less contribution to the latent CMD dimension than ST had in Cohort 1_{T1} dataset. This may be due to the differences in age structure and psychopathology status in both cohorts. Nonetheless, in both cohorts the peak in the ST curves occurred between +2 and +3 SD (high end of the CMD dimension), showing that ST lies on the more severe spectrum of CMD dimension than NSSI does.

For peer review only - http://bmjopen.bmj.com/site/about/guidelines.xhtml

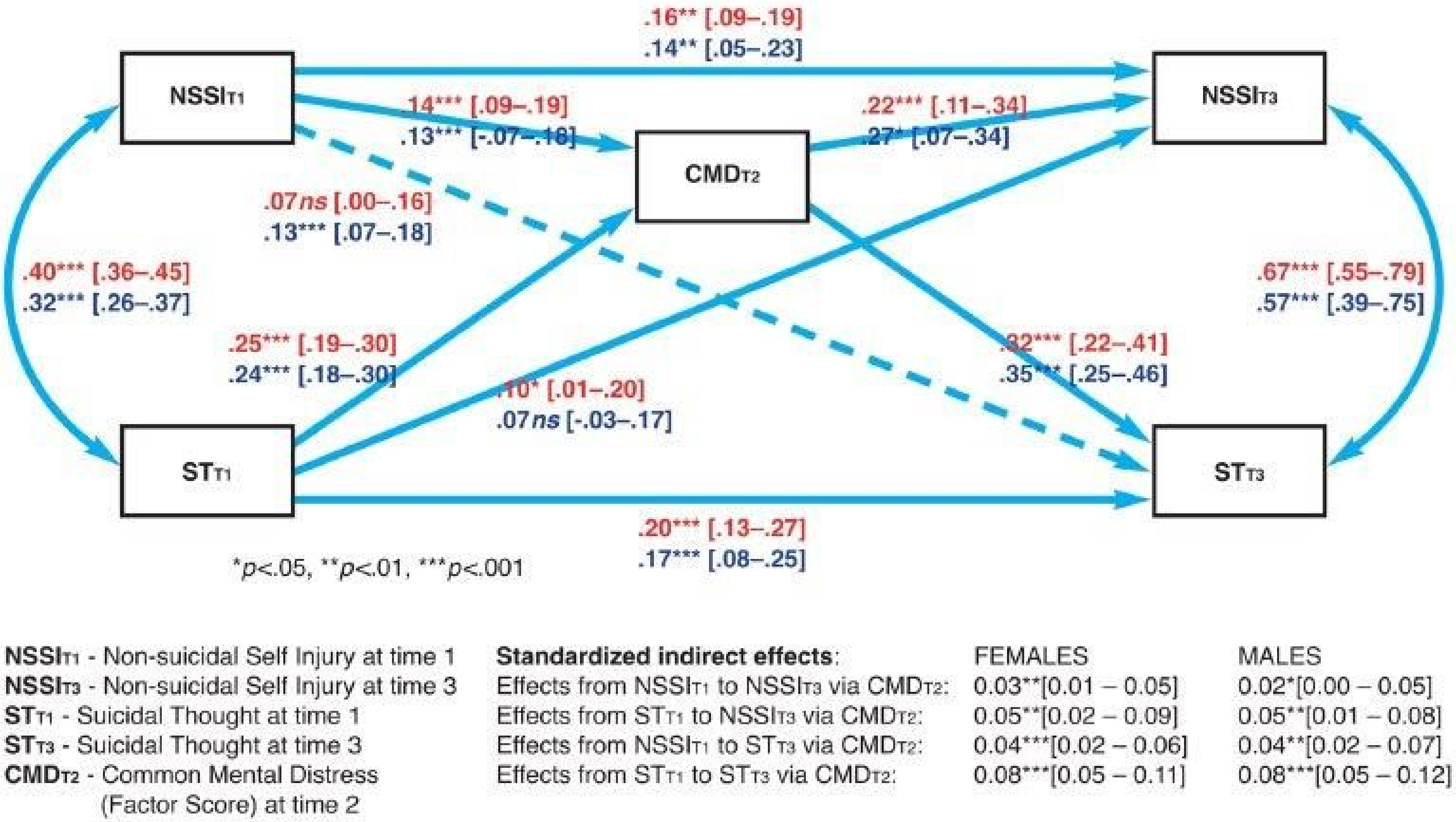

Supplementary Figure 2: Mediation effect of Common Mental Distress at time 2 (CMD_{T2}) moderated by sex (female n=1286 (red colour); male n=1115 (blue colour)) in the Cohort 1

Standardised pathway coefficients (with confidence intervals reported in squarer brackets) were obtained in multiple group pathway analysis in which sex was treated as a grouping variable. We tested the equivalence in pathway coefficients by means of comparing chi-square tests when the coefficient was "fixed" to be equal across sexes versus when it was free to vary across sexes[2]. We also tested the equivalence of fit indices of the model in both sexes. We found no evidence for differences in individual pathway coefficients or fit indices between sexes. This suggests that CMD at T2 mediated the longitudinal persistence of NSSI and ST in the same manner in females and males – no evidence of sex differences in the longitudinal mediation process was found. Additional details are reported in Supplementary Table 10.

Age and gender: Descriptive analysis

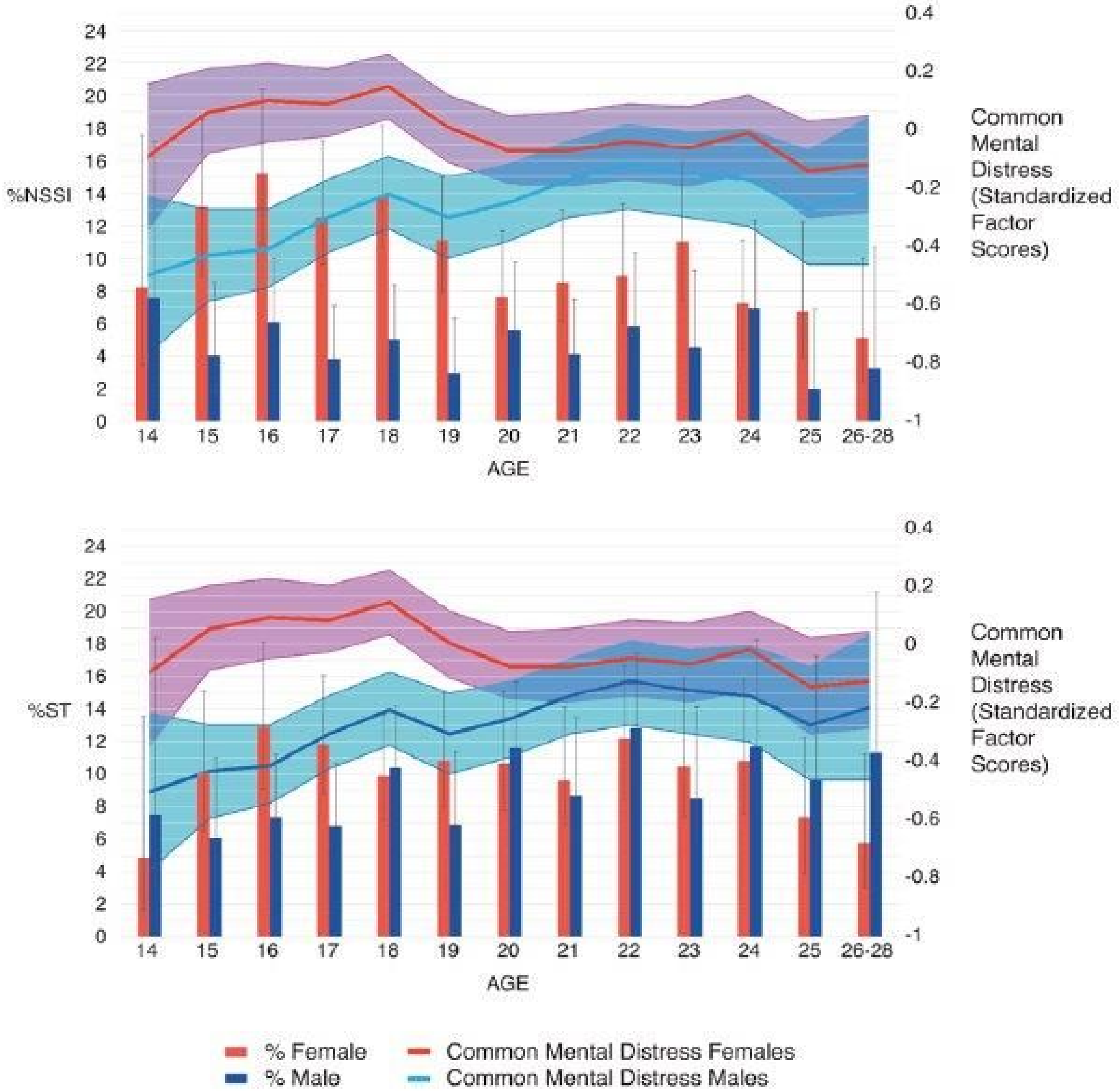

Supplementary Figure 3. Percentages of non-suicidal self-injury (NSSI), suicidal thoughts (ST) and levels of Common Mental Distress in age groups for both sexes in Cohort 1

To analyse the relationship between age, sex, NSSI, ST, and CMD descriptively, we grouped observations from all 3 time points in Cohort $1_{T1\text{-}T3}$ by age, rather than by data time point. This grouping allowed us to investigate levels of CMD, NSSI and ST in a broad age range of 14-28 years (note that this also entailed the inclusion of the same individuals from consecutive data sweeps (e.g., when an individual was 14, 15 and 16 years old) in the adjacent age groups). The histograms showing percentages of NSSI and ST with Wilson confidence intervals were plotted against the lines representing the means of CMD with confidence intervals for every age group for both sexes separately (Figure 3 above).

For peer review only - http://bmjopen.bmj.com/site/about/guidelines.xhtml

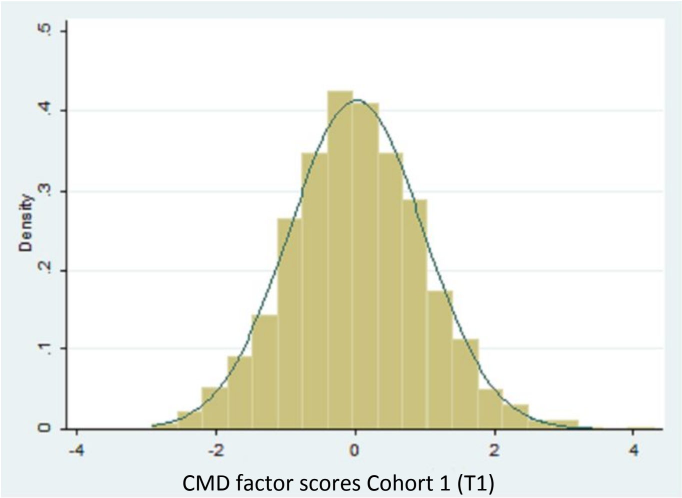

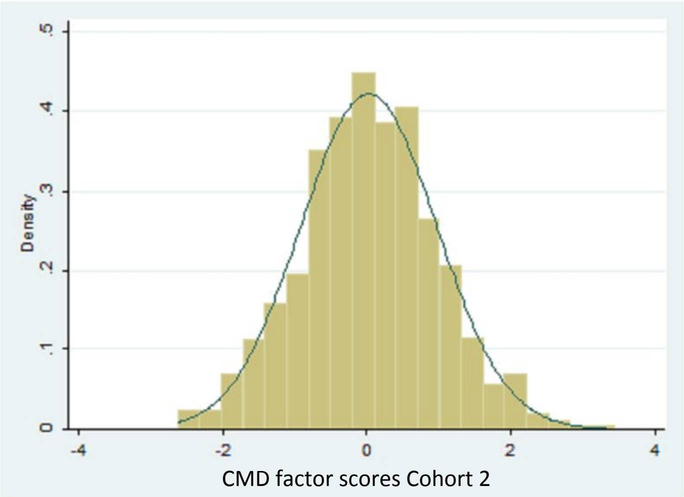

Supplementary Figure 4: Histograms of CMD factor scores in Cohort 1 (T1) and Cohort 2 with a schematic normal distribution line

For peer review only - http://bmjopen.bmj.com/site/about/guidelines.xhtml

Data collection tools:

Study data were collected and managed using REDCap electronic data capture tools[19] hosted at the University of Cambridge. REDCap (Research Electronic Data Capture) is a secure, web-based application designed to support data capture for research studies, providing 1) an intuitive interface for validated data entry; 2) audit trails for tracking data manipulation and export procedures; 3) automated export procedures for seamless data downloads to common statistical packages; and 4) procedures for importing data from external sources.

Group Information

NSPN (NeuroScience in Psychiatry Network: http://www.nspn.org.uk/) is a research consortium formed by the University of Cambridge and University College London, launched in November 2012 and supported by Wellcome Trust Award (095844/Z/11/Z). The group included the following members:

Principal investigators:

Edward Bullmore (CI from 01/01/2017) [1,2,3]

Raymond Dolan[4,5]

Ian Goodyer (CI until 01/01/2017) [1]

Peter Fonagy[6]

Peter Jones[1]

NSPN (funded) staff:

Michael Moutoussis[4,5]

Tobias Hauser[4,5]

Sharon Neufeld[1]

Petra Vértes[1,2]

Kirstie Whitaker[1,2]

Gita Prabhu[4,5]

Laura Villis[1]

Junaid Bhatti[1]

Becky Inkster[1]

Cinly Ooi[1]

Barry Widmer[1]

Ayesha Alrumaithi[1]

Sarah Birt[1]

For peer review only - http://bmjopen.bmj.com/site/about/guidelines.xhtml

Kalia Cleridou[5]

Hina Dadabhoy[5]

Sian Granville[5]

Elizabeth Harding[5]

Alexandra Hopkins[4,5]

Daniel Isaacs[5]

Janchai King[5]

Danae Kokorikou[5,6]

Harriet Mills[5]

Ciara O'Donnell[1]

Sara Pantaleone[5]

Aislinn Bowler[5]

Affiliated scientists:

Pasco Fearon[6]

Anne-Laura van Harmelen[1]

Rogier Kievit [4,7]

1 Department of Psychiatry, University of Cambridge, United Kingdom

2 Behavioural and Clinical Neuroscience Institute, University of Cambridge, United Kingdom

3 ImmunoPsychiatry, GlaxoSmithKline Research and Development, United Kingdom

4 Max Planck University College London Centre for Computational Psychiatry and Ageing Research,

University College London, UK

5 Wellcome Centre for Human Neuroimaging, University College London, United Kingdom

6 Research Department of Clinical, Educational and Health Psychology, University College London,

United Kingdom

7 Medical Research Council Cognition and Brain Sciences Unit, University of Cambridge, United Kingdom

For peer review only

References:

1 Reise, S. P. The rediscovery of bifactor measurement models. *Multivariate Behavioural Research*. 2012;47:667-696, doi:10.1080/00273171.2012.715555.

2 Muthen, L. & Muthen, B. *Mplus Users's Guide*. (Muthen & Muthen, 1998-2002).

3 Reise, S. P., Moore, T. M. & Haviland, M. G. Bifactor models and rotations: Exploring the extent to which multidimensional data yield univocal scale scores. *Journal of Personality Assessment*. 2010;92:544-559, doi:10.1080/00223891.2010.496477.

4 Noble M, McLennan, D, Wilkinson K, Whitworth A, & Barne H. The English Indices of Deprivation 2007. London: Department for Communities and Local Government. (2008).

5 van Buuren, S. & Groothuis-Oudshoorn, K. MICE: Multivariate Imputation by Chained Equations in R. *Journal of Statistical Software.*2011;45,1-67.

6 Sterne, J. A. C. *et al.* Multiple imputation for missing data in epidemiological and clinical research: Potential and pitfalls. *British Medical Journal.* 2009;339:157-160.

7 Rubin, D. B. *Multiple imputation for nonresponse in surveys.* (Wiley, 1987).

8 Hammerton, G., Zammit, S., Potter, R., Thapar, A. & Collishaw, S. Validation of a composite of suicide items from the Mood and Feelings Questionnaire (MFQ) in offspring of recurrently depressed parents. . *Psychiatry Research.* 2014;216:82-88.

9 Wilkinson P.O., Qiu T., Neufeld S., Jones P.B. & Goodyer I.M. Sporadic and recurrent non-suicidal self-injury before age 14 and incident onset of psychiatric disorders by 17 years: prospective cohort study. *British Journal of Psychiatry.*2018;212:222-226, doi:10.1192/bjp.2017.45.

10 Cassels M. *et al.* Poor family functioning mediates the link between childhood adversity and adolescent non-suicidal self-injury. *Journal of Child Psychology and Psychiatry*. 2018;59(8):881-887. doi: 10.1111/jcpp.12866

11 Angold, A. *et al.* The development of a short questionnaire for use in epidemiological studies of depression in children and adolescents. *International Journal of Methods in Psychiatric Research.*1995;5:237-249.

12 Reynolds, C. R. Concurrent validity of what I think and feel: The revised children's manifest anxiety scale. *Journal of Consulting and Clinical Psychology.* 1980;48:774-775. doi:10.1037/0022-006x.48.6.774 (1980).

13 Bamber, D., Tamplin, A., Park, R. J., Kyte, Z. A. & Goodyer, I. M. Development of a short Leyton Obsessional Inventory For Children and Adolescents. *Journal of the American Academy of Child and Adolescent Psychiatry.*2002;41:1246-1252.

14 St Clair C. M. *et al.* Characterising the latent structure and organisation of self-reported thoughts, feelings and behaviours in adolescents and young adults. *PLOS One.* 2017;12:1-27, doi:https://doi.org/10.1371/journal.pone.0175381.

15 Rosenberg, M. *Society and the adolescent self-image*. (Princeton University Press, 1965).

16 Tennant, R. *et al.* The Warwick-Edinburgh mental well-being scale (WEMWBS): development and UK validation. *Health and Quality of Life Outcomes* **5**, doi:10.1186/1477-7525-5-63 (2007).

17 Raine, A. The SPQ: A scale for the assessment of schizotypal personality based on DSM-III-R criteria. *Schizophrenia Bulletin.*1991;17:555-564.

18 Shaffer, D., Fisher, P., Lucas, C. P., Dulcan, M. K. & Schwab-Stone, M. E. NIMH Diagnostic Interview Schedule for Children Version IV (NIMH DISC-IV): description, differences from previous versions, and reliability of some common diagnoses. *Journal of the American Academy of Child and Adolescent Psychiatry*. 2000;39:28-38.

19 Harris PA, Taylor R, Thielke R, Payne J, Gonzalez N, Conde JG. Research electronic data capture (REDCap) – A metadata-driven methodology and workflow process for providing translational research informatics support. *Journal of Biomedical Informatics.* 2009;42(2):377-81.

For peer review only - http://bmjopen.bmj.com/site/about/guidelines.xhtml

STROBE (Strengthening The Reporting of OBservational Studies in Epidemiology) Checklist

A checklist of items that should be included in reports of observational studies. You must report the page number in your manuscript where you consider each of the items listed in this checklist. If you have not included this information, either revise your manuscript accordingly before submitting or note N/A.

Note: An Explanation and Elaboration article discusses each checklist item and gives methodological background and published examples of transparent reporting. The STROBE checklist is best used in conjunction with this article (freely available on the Web sites of PLoS Medicine at http://www.plosmedicine.org/, Annals of Internal Medicine at http://www.annals.org/, and Epidemiology at http://www.epidem.com/). Information on the STROBE Initiative is available at www.strobe-statement.org.

Section and Item	Item No.	Recommendation	Reported on Page No.
**Title and Abstract**	1	(*a*) Indicate the study's design with a commonly used term in the title or the abstract	1
		(*b*) Provide in the abstract an informative and balanced summary of what was done and what was found	2-3
**Introduction**			
Background/Rationale	2	Explain the scientific background and rationale for the investigation being reported	4-5
Objectives	3	State specific objectives, including any prespecified hypotheses	5
**Methods**			
Study Design	4	Present key elements of study design early in the paper	5-6
Setting	5	Describe the setting, locations, and relevant dates, including periods of recruitment, exposure, follow-up, and data collection	6-7
Participants	6	(*a*) *Cohort study*—Give the eligibility criteria, and the sources and methods of selection of participants. Describe methods of follow-up	6-7
		Case-control study—Give the eligibility criteria, and the sources and methods of case ascertainment and control selection. Give the rationale for the choice of cases and controls	not applicabl[illegible]
		Cross-sectional study—Give the eligibility criteria, and the sources and methods of selection of participants	not applicable
		(*b*) *Cohort study*—For matched studies, give matching criteria and number of exposed and unexposed	not applicable
		Case-control study—For matched studies, give matching criteria and the number of controls per case	not applicable
Variables	7	Clearly define all outcomes, exposures, predictors, potential confounders, and effect modifiers. Give diagnostic criteria, if applicable	7-8

For peer review only

Section and Item	Item No.	Recommendation	Reported on Page No.
Data Sources/ Measurement	8*	For each variable of interest, give sources of data and details of methods of assessment (measurement). Describe comparability of assessment methods if there is more than one group	Suppl. 3-8
Bias	9	Describe any efforts to address potential sources of bias	Suppl. 2, Ms.
Study Size	10	Explain how the study size was arrived at	Not applicabl
Quantitative Variables	11	Explain how quantitative variables were handled in the analyses. If applicable, describe which groupings were chosen and why	Supp.1, Ms. 7
Statistical Methods	12	(*a*) Describe all statistical methods, including those used to control for confounding	Supp.1, Ms. 7-
		(*b*) Describe any methods used to examine subgroups and interactions	Ms. 7-8
		(*c*) Explain how missing data were addressed	Suppl. 2
		(*d*) *Cohort study*—If applicable, explain how loss to follow-up was addressed	Suppl. 2
		Case-control study—If applicable, explain how matching of cases and controls was addressed	not applicable
		Cross-sectional study—If applicable, describe analytical methods taking account of sampling strategy	not applicable
		(*e*) Describe any sensitivity analyses	not applicable
**Results**			
Participants	13*	(a) Report numbers of individuals at each stage of study—eg numbers potentially eligible, examined for eligibility, confirmed eligible, included in the study, completing follow-up, and analysed	6
		(b) Give reasons for non-participation at each stage	not applicable
		(c) Consider use of a flow diagram	not applicable
Descriptive Data	14*	(a) Give characteristics of study participants (eg demographic, clinical, social) and information on exposures and potential confounders	Suppl.11, Ms.
		(b) Indicate number of participants with missing data for each variable of interest	Suppl. 2
		(c) *Cohort study*—Summarise follow-up time (eg, average and total amount)	6
Outcome Data	15*	*Cohort study*—Report numbers of outcome events or summary measures over time	9
		Case-control study—Report numbers in each exposure category, or summary measures of exposure	not applicable
		Cross-sectional study—Report numbers of outcome events or summary measures	not applicable

For peer review only

Section and Item	Item No.	Recommendation	Reported on Page No.
Main Results	16	(*a*) Give unadjusted estimates and, if applicable, confounder-adjusted estimates and their precision (eg, 95% confidence interval). Make clear which confounders were adjusted for and why they were included	Fig. 1, Suppl.[illegible]
		(*b*) Report category boundaries when continuous variables were categorized	Suppl. 9
		(*c*) If relevant, consider translating estimates of relative risk into absolute risk for a meaningful time period	Fig. 2
Other Analyses	17	Report other analyses done—eg analyses of subgroups and interactions, and sensitivity analyses	Suppl.9, 16
**Discussion**			
Key Results	18	Summarise key results with reference to study objectives	10-11
Limitations	19	Discuss limitations of the study, taking into account sources of potential bias or imprecision. Discuss both direction and magnitude of any potential bias	12
Interpretation	20	Give a cautious overall interpretation of results considering objectives, limitations, multiplicity of analyses, results from similar studies, and other relevant evidence	13-14
Generalisability	21	Discuss the generalisability (external validity) of the study results	13-14
**Other Information**			
Funding	22	Give the source of funding and the role of the funders for the present study and, if applicable, for the original study on which the present article is based	15

*Give information separately for cases and controls in case-control studies and, if applicable, for exposed and unexposed groups in cohort and cross-sectional studies.

Once you have completed this checklist, please save a copy and upload it as part of your submission. DO NOT include this checklist as part of the main manuscript document. It must be uploaded as a separate file.

For peer review only

BMJ Open

How do the prevalence and relative risk of non-suicidal self-injury and suicidal thoughts vary across the population distribution of common mental distress (the p-factor) in two independent UK cohorts of young people?

Journal:	*BMJ Open*
Manuscript ID	bmjopen-2019-032494.R2
Article Type:	Original research
Date Submitted by the Author:	04-Feb-2020
Complete List of Authors:	Polek, Ela; University of Cambridge, Psychiatry; University College Dublin, Psychology<br>Neufeld, Sharon A. S.; Univ Cambridge<br>Wilkinson, Paul; University of Cambridge, Cambridge Neuroscience<br>Goodyer, Ian; Cambridge University, Psychiatry<br>St Clair, Michelle<br>Prabhu, Gita<br>Dolan, Ray<br>Bullmore, Edward<br>Fonagy, Peter<br>Stochl, Jan; University of Cambridge, Department of Psychiatry; NIHR Collaboration for Leadership in Applied Health Research & Care (CLAHRC) East of England,<br>Jones, Peter; University of Cambridge, Department of Psychiatry
**Primary Subject Heading**:	Public health
Secondary Subject Heading:	Mental health
Keywords:	EPIDEMIOLOGY, Child & adolescent psychiatry < PSYCHIATRY, PUBLIC HEALTH, Suicide & self-harm < PSYCHIATRY

SCHOLARONE™
Manuscripts

I, the Submitting Author has the right to grant and does grant on behalf of all authors of the Work (as defined in the below author licence), an exclusive licence and/or a non-exclusive licence for contributions from authors who are: i) UK Crown employees; ii) where BMJ has agreed a CC-BY licence shall apply, and/or iii) in accordance with the terms applicable for US Federal Government officers or employees acting as part of their official duties; on a worldwide, perpetual, irrevocable, royalty-free basis to BMJ Publishing Group Ltd ("BMJ") its licensees and where the relevant Journal is co-owned by BMJ to the co-owners of the Journal, to publish the Work in this journal and any other BMJ products and to exploit all rights, as set out in our licence.

The Submitting Author accepts and understands that any supply made under these terms is made by BMJ to the Submitting Author unless you are acting as an employee on behalf of your employer or a postgraduate student of an affiliated institution which is paying any applicable article publishing charge ("APC") for Open Access articles. Where the Submitting Author wishes to make the Work available on an Open Access basis (and intends to pay the relevant APC), the terms of reuse of such Open Access shall be governed by a Creative Commons licence – details of these licences and which Creative Commons licence will apply to this Work are set out in our licence referred to above.

Other than as permitted in any relevant BMJ Author's Self Archiving Policies, I confirm this Work has not been accepted for publication elsewhere, is not being considered for publication elsewhere and does not duplicate material already published. I confirm all authors consent to publication of this Work and authorise the granting of this licence.

For peer review only

How do the prevalence and relative risk of non-suicidal self-injury and suicidal thoughts vary across the population distribution of common mental distress (the p-factor) in two independent UK cohorts of young people?

Ela Polek[1,9], Sharon Neufeld[1], Paul Wilkinson [1], Ian M. Goodyer[1], Michelle C. St Clair[2], Gita Prabhu[3], Ray Dolan[3,7], Ed Bullmore[1,5], Peter Fonagy[4], Jan Stochl[1,5,8] & Peter B. Jones[1,5,6]

[1] Department of Psychiatry, University of Cambridge, UK

[2] Department of Psychology, University of Bath, UK

[3] Wellcome Centre for Human Neuroimaging, University College London, UK

[4] Division of Psychology and Language Sciences, University College London, UK

[5] NIHR Applied Research Collaboration East of England, UK

[6] NIHR Cambridge Biomedical Research Centre, UK

[7] Max Planck UCL Centre for Computational Psychiatry and Ageing Research, UK

[8] Department of Kinanthropology, Charles University, Czech Republic

[9] School of Psychology, University College Dublin

Correspondence to:

Professor Peter B. Jones

Herchel Smith Building

Cambridge Biomedical Campus

CB2 0SZ

UK

Fax:01223 336581

e-mail: pbj21@cam.ac.uk

the total word count: 6026

For peer review only

Abstract:

Objectives: To inform suicide prevention policies and responses to youths at risk by investigating whether suicide risk is predicted by a summary measure of common mental distress (CMD, (the p-factor)) as well as by conventional psychopathological domains; to define the distribution of suicide risks over the population range of CMD; to test whether such distress mediates the medium-term persistence of suicide risks.

Design: Two independent samples of young people studied during three sweeps: the Neuroscience in Psychiatry (NSPN) 2400 cohort (n=2403) and the ROOTS cohort (n=1074); Cohorts 1 and 2, respectively.

Setting: Population-based in two UK centres.

Participants: Volunteers age 14-24 years recruited from primary health care registers, schools and colleges; advertisements to complete quotas in age-sex-strata.

Method: We analysed questionnaire data from Cohort 1 (sweeps 1-3) and Cohort 2 (sweep 3), collected between November 2012 – December 2016 and February 2008 – December 2009, respectively. We calculated a CMD score using confirmatory bifactor analysis; used logistic regressions to determine adjusted associations between risks and psychopathology (in continuous and above-the-norm categorical format); curve-fitting to examine the relative prevalence of suicidal thoughts (ST) and non-suicidal self-injury (NSSI) over the population distribution of CMD; and pathway mediation models to examine longitudinal associations.

Results: We found a dose-response relationship between levels of CMD and risk of suicide. The majority of all subjects experiencing ST and NSSI (78% and 76% in Cohort 1, and 66% and 71% in Cohort 2) had CMD scores no more than two standard deviations above the population mean; higher scores indicated the highest risk but were, by definition, infrequent. CMD mediated the longitudinal course of both ST and NSSI.

For peer review only

Conclusions. NSSI and ST in youths reflect common mental distress that also mediates their persistence. Universal prevention strategies reducing levels of CMD in the whole population without recourse to screening or measurement may prevent more suicides than approaches targeting youths with the most severe distress or with psychiatric disorders.

Article summary

Strengths and limitations of this study

- Replication of the findings in two independent cohorts strengthens confidence in the findings.
- Sample attrition was a limitation in both cohorts.
- Multiple imputations mitigated biases arising from attrition.

For peer review only - http://bmjopen.bmj.com/site/about/guidelines.xhtml

Introduction

Adolescence sees the onset of a range of psychopathology including suicidal thoughts (ST) and non-suicidal self-injury (NSSI)[1-3] that individually or together convey heightened risk of suicide attempts[4-6]. Non-suicidal and suicidal self-harm predict completed suicide[7], the second most common cause of deaths among 10 to 24-year-olds, worldwide[8]. Moreover, ST and NSSI are significant problems in their own right, representing a considerable burden to individuals, their families and health services. Prediction and prevention of self-harm and suicide in young people are priorities but NSSI (5-42% in community samples[9,10]) and ST (15-25% in community samples[11,12]) are common so it is difficult to predict who will ultimately make a serious attempt[13] or die by suicide. Indeed, the usefulness of clinical risk protocols relying on the identification of a psychiatric diagnosis is questionable[14,15]. The same problems affect public health suicide prevention programmes. A seminal study revealed a high prevalence of false-negatives in prospective identification of suicide[16]. Prevention policies that embrace the whole population might overcome these difficulties but lack theoretical or empirical foundations[1].

Suicidal thoughts and behaviours are routinely considered as markers of depression (e.g., in DSM-5) but by no means all young people dying by suicide have had a mood disorder[17]. NSSI is strongly associated with the risk of suicide when occurring in combination with any internalising or externalising symptoms[18,19], or with any psychiatric diagnosis[20], particularly multiple diagnoses[21]. Thus, this risk might be better predicted by multiple symptoms rather than by the presence of a single disorder, such as depression.

Recent studies suggest that a broad range of symptoms conventionally seen as components of distinct disorders are better construed as manifestations of a single, latent dimension distributed within the general population. This dimension has been variously referred to as the p-factor[22], general psychopathology[23] or, as we prefer here, common mental

For peer review only - http://bmjopen.bmj.com/site/about/guidelines.xhtml

distress (CMD)[24,25]. Parsimonious statistical models with dimensions that encompass low-prevalence phenomena such as psychotic experiences, fit empirical data better than models with distinct disorders[22,26]. High co-morbidity of psychiatric diagnoses, shared causal factors and treatments, and trans-diagnostic psychological and neural correlates support the validity of a CMD concept[22-24,26-29]. Suicide risk is related to multiple symptoms or disorders (and thus to higher CMD scores), not the presence of one specific symptom or disorder, so it is important to understand the nature of dose-response relationships between CMD and suicide risks. This could guide a clinical response in the face of suicide risk[30] and also shape population-based suicide prevention.

In this study, we describe the presence of a CMD dimension in young people aged 14-26 years and the occurrence of ST and NSSI referred to collectively, hereafter, as a suicide risk. We draw on a psychometric study[25] that demonstrated high theoretical validity and high measurement qualities of the CMD factor comprising measures of common mental illness (depression, anxiety, psychotic experiences, obsessions and compulsions) as well as traits and characteristics commonly considered to contribute to the general level of mental health (antisocial trait, well-being, self-esteem). Our approach had three steps whereby we:

1. Tested associations between CMD and suicide risk, and contrasted CMD with specific psychopathological domains, exploring the utility of this summary measure;
2. Defined the prevalence and relative risk of NSSI and ST across the distribution of CMD;
3. Established whether the CMD_{T2} dimension measured at time 2 mediate the relationship between ST_{T1} and $NSSI_{T1}$ at time 1 and $NSSI_{T3}$ and ST_{T3} at time 3.

We used data from two population-based cohorts with complementary designs and very similar measures. In step two we used cross-sectional data from Cohort 1, time 1 (used as a

For peer review only - http://bmjopen.bmj.com/site/about/guidelines.xhtml

discovery sample) and Cohort 2 (used as a stepwise replication sample); in the third step we used three longitudinal waves of Cohort 1 (see details in Method).

Method

Study Design and Participants

Cohort 1

Participants in the NSPN 2400 Cohort[31] were recruited largely via postal invitations sent through general practitioners and schools in Cambridgeshire and Greater London, UK. Data collection was carried out in two research centres: University College London and the University of Cambridge between November 2012 and December 2016. Purposive sampling obtained at least 200 males and 200 females from the community in 5 age groups: 14-15, 16-17, 18-19, 20-21, 22-24 years. Three data collections took place a year apart (T1-T3). At T1, 2403 individuals returned questionnaires (average age 18.9 years, SD=3.0; 54% females); at T2, 1815 returned questionnaires (76% response, average age 20.0 years, SD=3.1; 56% female), and 1245 at T3 (52% of baseline; average age 21.0 years, SD=3.1; 59% female).

Cohort 2

The ROOTS study[32] was used for replication of findings from Cohort 1. Two-stage sampling involved random selection of 27 schools in Cambridgeshire, UK. Eighteen schools agreed to participate; invitations were sent to 14-year-olds randomly selected from class registers and to their parents; 1238 students participated in the initial data collection (55% female) (and further 4 data collection waves took place). Note that in the current analysis we used only the data from the third data sweep collected between February 2008 and December 2009, when participants were of average age 17.5 years, *SD*=0.3 (N=1074, 56% female; 87% of baseline sample), the closest age to T1 of Cohort 1.

For peer review only - http://bmjopen.bmj.com/site/about/guidelines.xhtml

Both cohorts comprised predominantly white European (77% in Cohort 1 and 87% in Cohort 2) young people, consistent with the self-ascribed demographics of the two study populations. Written consent from participants age 14 or 15 years was supplemented by written consent from their parent or legal guardian; older participants gave their own written consent. Ethical approval was obtained for Cohort 1 from the National Health Service Research Ethics Service (# 97546) and for Cohort 2 from the Cambridgeshire 2 REC (# 03/302).

Measures

Sociodemographic information was collected using routine methods[31,33]. The Index of Multiple Deprivation (IMD), a summary measure of the socioeconomic status of participants' residential neighbourhood, is calculated from census information[34]. Questionnaires of mental illness and wellness are set out in Table 1 and items are listed in the Supplementary table 1. Scores in questionnaires were computed according to published manuals or validation studies (cited in Table 1), standardized to unify their measurement scales.

Table 1

Statistical analysis

Confirmatory bifactor analysis with a weighted least square mean and variance adjusted (WLMSV) estimator in Mplus 7.4 was used to compute factor scores for CMD in the three data sweeps of Cohort 1 and Cohort 2 based on the model validated elsewhere[25] (see CMD measures in Table 1 beneath; the list of used items and details of bifactor modelling can be found in the Supplementary table 1). CMD factor scores were then used in all subsequent computations. Next, we addressed attrition in Cohort 1 by means of multiple imputations (see details in the Supplement).

To prove that NSSI and ST were predicted by multiple psychopathological domains and also by CMD (which represents a summary of those domains), we used Stata 12 to

For peer review only - http://bmjopen.bmj.com/site/about/guidelines.xhtml

For peer review only

compute for Cohort 1_{T1} and Cohort 2 data sensitivity / specificity indicator – the area under the curve (AUC – reported in the Supplementary table 2) for NSSI and ST as criteria. We computed a series of logistic regressions, estimating odds ratios (OR) with confidence intervals for each predictor (treated as categorical with the cut-off point above 1SD and then continuous), while we controlled for effects of age and sex (Figure 1).

For step two, distributions of CMD scores in both cohorts were plotted against lines representing percentages of subjects reporting NSSI and ST within bands of CMD expressed as standard deviations (upper panel of Figure 2) and against bar histograms representing NSSI and ST frequencies in both cohorts (lower panel of Figure 2). In addition, NSSI and ST information curves were computed to determine in what range of the CMD dimension these items are located (see Supplementary figure 1).

Using Cohort $1_{T1\text{-}T3}$ data for step three, we examined the longitudinal relationship between CMD, NSSI and ST (in particular the predictive role of CMD in persistence of NSSI and ST): we computed direct and mediation (via CMD_{T2}) effects of ST_{T1} and $NSSI_{T1}$ on $NSSI_{T3}$ and ST_{T3} in a pathway mediation model with confidence intervals in Mplus 7.4 (computing bias-corrected bootstrapping was not possible due to the use of multiply imputed datasets). We computed this model for the total sample (Figure 3) and then for both sexes separately (Supplementary figure 2) using the Multiple Group Method, so as to test a moderated-mediation model (with CMD_{T2} as a mediator, and sex as a moderator). Age was a control variable. In both pathway analyses CMD_{T2} factor scores (computed on imputed data, as described above) were modelled as observed variables.

Results

Step one: Associations of NSSI and ST with demographic and psychopathological variables

For peer review only - http://bmjopen.bmj.com/site/about/guidelines.xhtml

In both cohorts NSSI and ST were unrelated to demographic variables, including sex and age (See Supplementary tables 3 and 4); CMD was negatively related to male gender (Supplementary table 5). When examined descriptively over the pooled age groups, the prevalence of NSSI and ST mirrored the CMD levels (see Supplementary figure 3). CMD and all "conventional" mental health disorders predicted NSSI and ST (i.e., had statistically significant ORs in logistic regression models - see Figure 1 and Supplementary table 2).

Figure 1

Prevalence of NSSI and ST in the two cohorts

In Cohort 1 (N=2403) there was no statistically significant change in the prevalence of NSSI (within the last month) over the three time points: in the imputed data 9.3% (n=223) reported $NSSI_{T1}$, 8.3% (n=199) $NSSI_{T2}$ and 8.2% (n=197) $NSSI_{T3}$. Similarly, there was no statistically significant change in prevalence of ST (within the last two weeks) over the three time points: 10.1% (n=243) ST_{T1}, 11.4% (n=274) ST_{T2} and 11.7% (n=281) ST_{T3} (see Supplementary tables 6 and 7).

In Cohort 2 (N=1074), 11.7% (n=126) reported lifetime NSSI and 5.4% (n=58) reported ST within the two last weeks. Accuracy and precision of these prevalence estimates were affected by attrition (see *Discussion: limitations*). Attrition in Cohort 1 at T2 and T3 was only marginally related to demographic and exposure variables at T1 (Spearman's rho 0.05-0.12), but unrelated to the outcome – NSSI and ST (see Supplementary table 8).

Step two: Associations of NSSI and ST with CMD

Next, we focused on absolute risk[1] and the numbers of NSSI and ST events generated by these risk functions. The dose-response curves in the upper panel of Figure 3 show that

[1] Absolute risk is the probability or chance of an event. It is usually used for the number of events (e.g., a suicide) that occurred in a group, divided by the number of people in that group.

For peer review only - http://bmjopen.bmj.com/site/about/guidelines.xhtml

relative risks[2] of NSSI and ST increased markedly with increasing severity of CMD, the highest risks being in those with very high scores beyond two standard deviations above the mean. On the other hand, most participants from both cohorts who reported NSSI or ST had mild (one SD above the mean) to moderate (two SD above the mean) CMD scores (lower panel of Figure 3). CMD was normally distributed (see Supplementary figure 4) so these scores were much more common; only a minority of the total reports came from the few participants with very high CMD (>2 standard deviations above mean CMD). Thus, the majority of subjects experiencing ST or NSSI (Cohort 1: 78% and 76%; Cohort 2: 66% and 71%, respectively) had CMD scores within two standard deviations above the population mean. Very high CMD scores indicated the highest suicide risk but were rare, so generated the minority of events.

Figure 2

Step three: Mediating effect of CMD on suicide risks in Cohort 1 over time

Cohort 1 CMD_{T2} contributed to the persistence of NSSI and ST over time (i.e. $NSSI_{T1}$ predicted $NSSI_{T3}$ directly, and via mediation through CMD_{T2}; it also completely mediated the longitudinal effect of $NSSI_{T1}$ on ST_{T3}). Moreover, CMD_{T2} contributed to the persistence of ST over time (i.e. ST_{T1} predicted ST_{T3} directly, as well as via mediating variable – CMD_{T2}). Overall, CMD_{T2} was a stronger predictor of $NSSI_{T3}$ and ST_{T3} than the antecedent variables measured at T1 (see Figure 3). The mediation effects of CMD_{T2} were similar for boys and girls (i.e., the effects were not moderated by sex – Supplementary figure 2 and Supplementary table 9). Age_{T1} was not a significant predictor of any variable in the model; the results when age was controlled for were very similar to those without controlling for age.

Figure 3

[2] A relative risk compares the risk of a health event (e.g., a suicide) among one group with the risk among another group.

For peer review only - http://bmjopen.bmj.com/site/about/guidelines.xhtml

Discussion

In the present study, all the domains of psychopathology and mental wellness available (depression, anxiety, self-esteem, well-being, psychotic-like experiences, antisocial trait, schizotypal trait, conduct problems, obsessions and compulsions) predicted risk of non-suicidal self-injury (NSSI) and suicidal thoughts (ST). Thus, the common mental distress factor with a normal population distribution appeared a parsimonious and efficient summary of these domains and was, itself, a key predictor of suicide risk in both cohorts. NSSI and ST were not confined to participants scoring in the very high, quasi-clinical range for CMD. Around half of all participants expressing NSSI or ST came from those scoring up to one standard deviation above mean CMD in a dose-response manner. The majority expressing these phenomena (two thirds to three quarters) scored within 2SD above the mean (Figure 2). Regarding medium-term determinants of persistent NSSI and ST we showed (Figure 3) that CMD_{T2} mediated the persistence of NSSI and ST over two years, independent of gender and age. This mediation operates in two stages: first, ST and NSSI persist because these behaviours are markers for worsening CMD in the general population. This extends findings in adolescents with depressive disorder, where suicidal thoughts are a predictor of poor outcome[35]. Second, this greater CMD, itself, predicts the risk for further suicidal thoughts and behaviours.

Strengths

Both cohorts were designed on epidemiological principles to capture behavioural and psychological variation in the population during the post-pubertal epoch during which risk for psychopathology accelerates. Replication of the findings in these independent cohorts strengthens confidence in the findings, as does internal consistency between cross-sectional associations found in both cohorts, and longitudinal associations found in Cohort 1.

For peer review only - http://bmjopen.bmj.com/site/about/guidelines.xhtml

Limitations

Sample attrition was the main bias in both cohorts. Each retained more young women than men; we found marginally higher attrition among lower socio-economic class, participants of non-white ethnicity and those with higher CMD (Supplementary table 8). Cohort 1 is robustly representative of the England and Wales population[31], whereas Cohort 2 under-represents participants with lowest socioeconomic status[32]. However, we have no reason to suppose that attrition biased our results, as it was unrelated to NSSI and ST (Supplementary table 8). If there was a bias, it probably limits power rather than skewing an effect and is mitigated by replication between the cohorts. We used multiple imputation to minimise this bias.

There was only modest reliability of our obsessionality measure and a skewed measure of conduct problems in Cohort 1. A completely comprehensive range of psychopathological (and behavioural) items was unavailable; we did not have measures of unstable or abnormally elevated mood, addictions, eating disorders or hyperactivity. Thus, our measurement of CMD focused primarily on internalising rather than externalising symptoms. Future studies could include a broader range of measures and extend the investigation into clinical populations to improve measurement precision at the highest levels of CMD. Although ethnicity and socioeconomic status (indicated by IMD) were unrelated to ST and NSSI (Supplementary tables 3 and 4), and thus were not included in our analyses, we did not control for the effect of other possible confounders such as adverse life experiences, early trauma, family structure or more detailed information about family socio-economic situation (unemployment, poverty etc.). Finally, we could not account for the effects of clustered design in the modelling, due to unavailability of the information about clustering of participants in both cohorts.

For peer review only - http://bmjopen.bmj.com/site/about/guidelines.xhtml

Implications & Conclusions

Our findings provide yet more evidence that a latent mental distress factor, conceptually akin to the p-factor, is a useful summary measure of psychopathology in the general population[24], diagnostic[22], and clinical[23] samples. We speculate that psychopathological items accumulate in a probabilistic manner rather than in diagnostic clusters, with common phenomena concerning depression and anxiety much more likely to occur before rarer phenomena such as NSSI, ST or psychotic experiences. Less frequent phenomena begin to co-occur as the severity of psychological disorder (or CMD) increases, in terms of more mental and behavioural phenomena or symptoms. This begins to yield clusters linked by common items that current diagnostic systems tend to ignore. This is consistent with the co-occurrence of suicidal risk and psychotic experiences seen in other[36-38] studies of young people, and with the present IRT analysis showing that NSSI and ST are measuring the higher end of CMD (Supplementary figure 1). The approach we have followed illustrates the value of moving away from categorical classification and embracing an empirically-rooted, dimensional, hierarchical taxonomy in psychopathology research[39]. Such hierarchical approaches to phenomenological classification had been put forward before[40] or shortly after[41] the publication of DSM-3 and its successor classifications. Hierarchical models merit renewed interest[42], as they may resolve problems of comorbidity[26] as well as overlapping causes and biological mechanisms for suicide risk and other phenomena[43,44]. In contrast to the CMD idea, there is also increasing interest in approaches focusing on individual symptoms and experiences, particularly to guide individual clinical interventions, rather than grouping the symptoms into diagnostic categories or higher-order constructs[45]. Future studies may investigate and compare the utility of such novel approaches (CMD and item-focused approach) for clinical practice and public health policies.

For peer review only - http://bmjopen.bmj.com/site/about/guidelines.xhtml

Our findings also have major implications for intervention and prevention of suicidal thoughts and behaviours. Clinically, the results suggest that NSSI and ST should never be dismissed or downplayed when they occur in young people without clear evidence of psychiatric disorder, a logical fallacy because NSSI and ST are *themselves* indicators of higher distress on a CMD factor. NSSI and ST will usually, but not always occur with other, more common psychopathology and their co-occurrence is a strong risk factor for suicide attempts[6]. Thus, NSSI and ST merit a swift professional response regardless of whether or not they occur with other symptoms that take individuals beyond conventional clinical thresholds and trigger traditional clinical risk protocols. Our findings help explain why research focused on high-risk subjects has yet to translate into useful clinical prediction tools[14,15,45].

From a public health and prevention perspective, the fact that rates of NSSI and ST begin to accelerate at levels of CMD well within a normal or non-clinical range argues strongly for universal interventions overtly aimed at lowering the population mean CMD and shifting the curve to the left. This should be alongside targeted approaches and effective clinical services[46]. Strategies concentrated on clinical populations, those with evidence of a psychiatric disorder or other individual markers will miss the majority of individuals experiencing ST or engaging in NSSI because there are so few compared with those at lower risk: the *prevention paradox*[30].

Defining putative universal interventions to shift the population distribution of CMD will require careful research that can draw from other areas of medicine such as cardiovascular disease and stroke[30]. Elements have been widely scoped in the USA[15] and elsewhere, but not for constructs of population health and wellbeing such as CMD. Interventions may involve decreasing common triggers[15] or improving young people's abilities to cope with stressors[47, 48, 49].

For peer review only - http://bmjopen.bmj.com/site/about/guidelines.xhtml

Conflict of Interest Disclosures

E.P., S.N., I.M.G., and J.S. have no competing interests. E.B., P.F., and P.B.J. are in receipt of National Institute for Health Research (NIHR) Senior Investigator Awards (NF-SI-0514-10157, and NF-SI-0514-10117. P.F. was in part supported by the NIHR Collaboration for Leadership in Applied Health Research and Care (CLAHRC) North Thames at Barts Health NHS Trust. P.W. has recent/current grant support from NIHR, Cambridgeshire County Council and CLAHRC East of England. P.W. discloses consulting for Lundbeck and Takeda; P.B.J. discloses consulting for Janssen and Ricordati. E.B. is employed half-time by the University of Cambridge and half-time by GlaxoSmithKline in which he holds stock.

Data sharing/ Data availability

E.P. had full access to all the data in the study and takes responsibility for the integrity of the data and the accuracy of the data analysis. The data are deposited in the University of Cambridge Data Repository, with the placeholder DOI https://doi.org/10.17863/CAM.25331 available to researchers via openNSPN@medschl.cam.ac.uk.

Funding/Support and Acknowledgments

The ROOTS study was supported by a Wellcome Trust Grant (Grant number 074296) to I.M.G. and P.B.J., the NIHR Collaborations for Leadership in Applied Research and Care (CLAHRC) East of England, and the NIHR Cambridge Biomedical Research Centre. The NSPN study was supported by the Wellcome Trust Strategic Award (095844/Z/11/Z) to I.M.G., E.B., P.B.J., R.D., P.F. The work has been carried out in the Department of Psychiatry, University of Cambridge. We wish to thank the NSPN and ROOTS participants and Dr Golam Khandaker for his comments and NSPN Consortium (see the list of members in the Supplement).

Role of the Funder/Sponsor

The funding sources had no role in the design and conduct of the study; collection, management, analysis, and interpretation of the data; preparation, review, or approval of the manuscript; and decision to submit the manuscript for publication.

For peer review only - http://bmjopen.bmj.com/site/about/guidelines.xhtml

NSPN Consortium Information –see the Supplement

Patient and public involvement: Patients and the public were not involved in the design or planning of the study.

For peer review only

For peer review only - http://bmjopen.bmj.com/site/about/guidelines.xhtml

Table 1. Measures used in both cohorts

Variables	*Measures*	Cohorts	
**Outcome variables:**		NSPN$_{T1-T3}$ (1)	ROOTS$_{age\ 17}$ (2)
Suicidal thoughts (ST)	One item from the MFQ[50]: I thought about killing myself. Responses were recoded into a binary format: no ST (original response option *Never)* and ST (original response options *Sometimes* or *Mostly* or *Always*).	×	×
Non-suicidal self-injury (NSSI)	One question from the Drug, Alcohol and Self-Injury (DASI)[25] questionnaire asking about engaging in self-injury without suicidal intent during the last month. Responses were recoded into a binary format indicating the occurrence of NSSI or lack of thereof.	×	
	One question asking about the occurrence of lifetime NSSI (DASI)[25]		×
**Predictors:**			
Conduct problems	11-item Antisocial Behaviour Questionnaire[25]	×	×
Anxiety	28-item Revised Children's Manifest Anxiety Scale[51]	×	×
Depression	29 items from the 33-item MFQ[50] (all items except for 4 items measuring suicidality)		
Obsessions and compulsions	11-item Revised Leyton Obsessional Inventory[52]	×	×
Psychotic-like experiences	11 items selected from the 74-item Schizotypal Personality Questionnaire (SPQ)[53]	×	
	11 items from the 20-item semi-structured interview from the Diagnostic Interview Schedule for Children-IV[54]		×
Self-esteem	10-item Rosenberg Self-Esteem Questionnaire (*)[55]	×	×
Well-being	14-item Warwick-Edinburgh Mental Well-Being Scale(*)[56]	×	×
Impulsivity	15 items from the 30-item Barratt Impulsiveness Scale[57] selected based on exploratory factor analysis - loadings above .25	×	
Antisocial traits	Total score from the 17-item Antisocial Process Screening Device (APSD)[58]	×	
Schizotypal traits	Total score from the 74-item Schizotypal Personality Questionnaire (SPQ) [53]	×	×

*scales were reversely scored, thus higher scores indicated lower self-esteem and well-being; for all other measures higher score indicates more psychopathology

For peer review only

For peer review only - http://bmjopen.bmj.com/site/about/guidelines.xhtml

Figures' legends:

Figure 1: Odds ratio in logistic regressions for suicidal thoughts (ST) and non-suicidal self-harm (NSSI) as outcomes predicted by psychopathological predictors (listed on the left) here treated as continuous variables; regressions were computed separately for each predictor and effects of age and sex were controlled in each regression for in both cohorts (see Supplementary Table 2).

Figure 2: Upper panel shows the dose-response effect of Common Mental Distress on non-suicidal self-harm (NSSI) and suicidal thought (ST) in Cohort 1 and Cohort 2. The lower panel shows the proportion of total reports in non-suicidal self-injury (NSSI) and suicidal thought (ST) broken down by standard deviations of Common Mental Distress; these add up to 100% from left to right. The normal population distribution of CMD, which was strikingly similar, but not identical, in Cohort 1 and 2, is shown by the purple line (see density plots in Supplement, Figure 1).

Figure 3: Mediation effect of Common Mental Distress at time 2 in Cohort 2: Standardised pathway coefficients with confidence intervals in square brackets.

For peer review only

For peer review only - http://bmjopen.bmj.com/site/about/guidelines.xhtml

References:

1. Hawton K, Saunders EA, O'Connor R. Self-harm and suicide in adolescents. *Lancet.* 2012;379:2373-2382.
2. Kidger J, Heron J, Lewis G, Evans J, Gunnell D. Adolescent self-harm and suicidal thoughts in the ALSPAC cohort: A self-report survey in England. *BMC Psychiatry.* 2012;12:1-12.
3. Nock MK. Future directions for the study of suicide and self-injury. J Clin Child Adolesc Psychol. 2012;41:255-259.
4. Scott LN, Pilkonis PA, Hipwell AE, Keenan K, Stepp SD. Non-suicidal self-injury and suicidal ideation as predictors of suicide attempts in adolescent girls: A multi-wave prospective study. *Compr Psychiatry.* 2015;58:1-10.
5. Ribeiro JD, Franklin JC, Fox KR, et al. Self-injurious thoughts and behaviors as risk factors for future suicide ideation, attempts, and death: A meta-analysis of longitudinal studies. *Psychol Med.* 2016;46:225-236.
6. Victor SE, Klonsky ED. Correlates of suicide attempts among self-injurers: A meta-analysis. *Clin Psychol Rev.* 2014;34(4):282-297.
7. Cooper J, Kapur N, Webb R, et al. Suicide after deliberate self-harm: a 4-year cohort study. *Am J Psychiatry.* 2005;162:297-303.
8. Patton GC, Coffey C, Sawyer SM, et al. Global patterns of mortality in young people: a systematic analysis of population health data. *Lancet*. 2009;374:881-892.
9. Muehlenkamp JJ, Claes L, Havertape L, Plener PL. International prevalence of adolescent non-suicidal self-injury and deliberate self-harm. *Child Adolesc Psychiatry Ment Health.* 2012;6(10).
10. Brunner R, Parzer P, Haffner J, et al. Prevalence and psychological correlates of occasional and repetitive deliberate self-harm in adolescents. *Arch Pediatr Adolesc Med.* 2007;161(7):641-649.
11. Bridge JA, Goldstein TR, Brent DA. Adolescent suicide and suicidal behavior. *J Child Psychol Psychiatry*. 2006;47:372-394.
12. Evans E, Hawton K, Rodham K, Deeks J. The prevalence of suicidal phenomena in adolescents: A systematic review of population-based study. *Suicide Life Threat Behav.* 2005;35(3):239-50.
13. Nielssen O, Wallace D, Large M. Pokorny's complaint: the insoluble problem of the overwhelming number of false positives generated by suicide risk assessment. *BJPsych Bulletin.* 2017;41:18-20.
14. Quinlivan L, Cooper J, Davies L, et al. Which are the most useful scales for predicting repeat self-harm? A systematic review evaluating risk scales using measures of diagnostic accuracy. *BMJ Open.* 2016;6(2): https://bmjopen.bmj.com/content/6/2/e009297.
15. Quinlivan L, Jayne Cooper J, Meehan D, et al. Predictive accuracy of risk scales following self-harm: multicentre, prospective cohort study. *Br J Psychiatry.* 2017;210:429-436.
16. Pokorny AD. Prediction of suicide in psychiatric patients. Report of a prospective study. *Arch Gen Psychiatry.* 1983;40:249-257.
17. Shaffer D, Gould MS, Fisher P, et al. Psychiatric diagnosis in child and adolescent suicide. *Arch Gen Psychiatry.* 1996;53:339-348.
18. Berona J, Horwitz AG, Czyz EK, King CA. Psychopathology profiles of acutely suicidal adolescents: Associations with post-discharge suicide attempts and rehospitalisation. *J Affect Disord*. 2017;209:97-104.
19. Nock MK, Joiner TE, Gordon KH, Lloyd-Richardson E, Prinstein MJ. Non-suicidal self-injury among adolescents: diagnostic correlates and relation to suicide attempts. *Psychiatry Res*. 2006;144(1):65-72.
20. Beckman K, Mittendorfer-Rutz E, Lichtenstein P, et al. Mental illness and suicide after self-harm among young adults: long-term follow-up of self-harm patients, admitted to hospital care, in a national cohort. *Psychol Med.* 2016;46:3397-3405.

For peer review only - http://bmjopen.bmj.com/site/about/guidelines.xhtml

21. Windfuhr K, While D, Kapur N, et al. Suicide risk linked with clinical consultation frequency, psychiatric diagnoses and psychotropic medication prescribing in a national study of primary-care patients. *Psychol Med.* 2016;46:3407-3417.
22. Caspi A, Houts RM, Belsky DW, et al. The p factor: One General Psychopathology Factor in the structure of psychiatric disorders? *Clin Psychol Sci.* 2014;2:119-137.
23. Patalay P, Fonagy P, Deighton J, et al. A general psychopathology factor in early adolescence. *The Br J Psychiatry.* 2015;207:15-22.
24. Stochl J, Khandaker GM, Lewis G, et al. Mood, anxiety and psychotic phenomena measure a common psychopathological factor. *Psychol Med.* 2015;45:1483-1493.
25. St Clair CM, Neufeld S, Jones BP, et al. Characterising the latent structure and organisation of self-reported thoughts, feelings and behaviours in adolescents and young adults. *PLOS* One. 2017; 12(4), 1-27. doi: https://doi.org/10.1371/journal.pone.0175381
26. Krueger RF. The structure of common mental disorders. *Arch Gen Psychiatry.* 1999;56:921-926.
27. Barch DM. The Neural Correlates of Transdiagnostic Dimensions of Psychopathology. *Am J Psychiatry*. 2017;174:613-615.
28. McTeague LM, Huemer J, Carreon DM, et al. Identification of Common Neural Circuit Disruptions in Cognitive Control Across Psychiatric Disorders. *Am J Psychiatry*. 2017;174:676-685.
29. Sharma A, Wolf DH, Ciric R, et al. Common Dimensional Reward Deficits Across Mood and Psychotic Disorders: A Connectome-Wide Association Study. *Am J Psychiatry.* 2017;174:657-666.
30. Rose G, Khaw M, Marmot G, Kay-Tee K, Marmot M. Chapter 3: The relation of risk to exposure. In: *Rose's strategy of preventive medicine (New ed.)* Oxford, Oxford University Press: 2008.
31. Kiddle B, Inkster B, Prabhu G, et al. The NSPN 2400 Cohort: a developmental sample supporting the Wellcome Trust NeuroScience in Psychiatry Network. *Int J Epidemiol.* 2018;47(1):18-19g.
32. Goodyer IM, Croudace T, Dunn V, Herbert J, Jones BP. Cohort Profile: Risk patterns and processes for psychopathology emerging during adolescence: the ROOTS project. *Int J Epidemiol.* 2010;39:361-369.
33. Office for National Statistics. Ethnicity and National Identity in England and Wales. 2012. Retrieved on 6th Feb 2018 from: https://www.ons.gov.uk/peoplepopulationandcommunity/culturalidentity/ethnicity/articles/ethnicityandnationalidentityinenglandandwales/2012-12-11#ethnicity-in-england-and-wales.
34. Noble M, McLennan, D, Wilkinson K, Whitworth A, & Barne H. The English Indices of Deprivation 2007. London: Department for Communities and Local Government. (2008).
35. King RA. Adolescent suicidal thoughts/behaviors are a marker of long-term vulnerability to poor adult outcomes. *J Am Acad Child Adolesc Psychiatry.* 2017;56:920-921.
36. Kelleher I, Keeley H, Corcoran P, et al. Clinicopathological significance of psychotic experiences in non-psychotic young people: evidence from four population-based studies. *Br J Psychiatry*. 2012;201:26-32.
37. Honings S, Drukker M, Groen R, van Os J. Psychotic experiences and risk of self-injurious behaviour in the general population: a systematic review and meta-analysis. *Psychol Med.* 2016;46:237-251.
38. Kelleher I, Corcoran P, Keeley H, et al. Symptoms and population risk for suicide attempt: A prospective cohort study. *JAMA Psychiatry.* 2013;70:940-948.
39. Lahey BB, Krueger RF, Rathouz PJ, Waldman ID, Zald DH. A hierarchical causal taxonomy of psychopathology across the life span. *Psychol Bull.* 2017;143:142-186.
40. Foulds GA, Bedford A. Hierarchy of classes of personal illness. *Psychol Med.* 1975;5:181-192.
41. Sturt E. Hierarchical patterns in the distribution of psychiatric symptoms. *Psychol Med.* 1981;11:783-792.

For peer review only - http://bmjopen.bmj.com/site/about/guidelines.xhtml

42. Kotov R, Krueger RF, Watson D. A paradigm shift in psychiatric classification: the Hierarchical Taxonomy Of Psychopathology (HiTOP). *World Psychiatry.* 2018;17:24-25.
43. Zald DH, Lahey BB. Implications of the Hierarchical Structure of Psychopathology for Psychiatric Neuroimaging. *Biol Psychiatry Cogn Neurosci Neuroimaging.* 2017;2:310-317.
44. Carver CS, Johnson SL, Timpano KR. Toward a functional view of the p factor in psychopathology. *Clinical Psychological Scienc.* 2017;5(5):880-889.
45. Fried EI, & Nesse RM. Depression sum-scores don't add up: why analyzing specific depression symptoms is essential. BMC Medicine. 2015; 13:72; https://bmcmedicine.biomedcentral.com/track/pdf/10.1186/s12916-015-0325-4
46. Franklin JC, Ribeiro JD, Fox KR, et al. Risk factors for suicidal thoughts and behaviors: A meta-analysis of 50 years of research. *Psychol Bull.* 2017;143(2):187-232.
47. Lewis G, Hawton K, Jones PB. Strategies for preventing suicide. *British Joournal of Psychiatry.* 1997;171:351-354.
48. Galante J, Dufour G, Vainre M, et al. A mindfulness-based intervention to increase resilience to stress in university students (the Mindful Student Study): a pragmatic randomised controlled trial. Lancet Public Health. 2018 Feb;3(2):e72-e81.
49. Wasserman D, Hoven CW, Wasserman C, et al. School-based suicide prevention programmes: the SEYLE cluster-randomised, controlled trial. *Lancet*. 2015;385:1536-1544.
50. Hammerton G, Zammit S, Potter R, Thapar A, Collishaw S. Validation of a composite of suicide items from the Mood and Feelings Questionnaire (MFQ) in offspring of recurrently depressed parents. *Psychiatry Res.* 2014;216:82-88.
51. Reynolds CR. Concurrent validity of what I think and feel: The revised children's manifest anxiety scale. J Consult Clin Psychol. 1980;48:774-775.
52. Bamber, D., Tamplin, A., Park, R.J., Kyte, Z.A. & Goodyer, I.M. Development of a short Leyton Obsessional Inventory For Children and Adolescents. *Journal of the American Academy of Child and Adolescent Psychiatry* **41**, 1246-1252 (2002).
53. Raine A. The SPQ: A scale for the assessment of schizotypal personality based on DSM-III-R criteria. *Schizophr Bull.* 1991;17:555-564.
54. Shaffer D, Fisher P, Lucas CP, Dulcan MK, Schwab-Stone ME. NIMH Diagnostic Interview Schedule for Children Version IV (NIMH DISC-IV): description, differences from previous versions, and reliability of some common diagnoses. *J Am Acad Child Adolesc Psychiatry.* 2000;39:28-38.
55. Rosenberg M. *Society and the adolescent self-image*. Princeton, NJ: Princeton University Press; 1965.
56. Tennant R, Hiller L, Fishwick R, et al. The Warwick-Edinburgh mental well-being scale (WEMWBS): development and UK validation. Health Qual Life Outcomes. 2007;5:63.
57. Patton JH, Stanford MS, Barratt ES. Factor structure of the Barratt Impulsiveness Scale. *J Soc Clin Psychol.* 1995;51:768-774.
58. Poythress NG, Poythress NG, Douglas KS, Falkenbach D, et al. Internal consistency reliability of the self-report antisocial process screening device. *Asmnt.* 2006;13:107-113.

For peer review only - http://bmjopen.bmj.com/site/about/guidelines.xhtml

Authors contributions:

E.P. – conceptualised the study, computed statistical analyses and drafted the manuscript

P.B.J. – provided senior supervision, conceptualised the study, advised on statistical analyses, read and critically apprised the manuscript, re-drafted and edited the manuscript

J.S. – provided statistical advice, replicated multiple imputations, provided data from multiple imputations, read and critically apprised the manuscript

S.N. – advised on handling missing data, replicated multiple imputations, read and critically apprised the manuscript

P.W. – read and critically apprised the manuscript, provided key referred articles

R.D. – read and critically apprised the manuscript, provided key referred articles

I.M.G. – read and critically apprised the manuscript, provided key referred articles

E.B. – read and critically apprised the manuscript, provided key referred articles

P.F. – read and critically apprised the manuscript, provided key referred articles

R.D. – read and critically apprised the manuscript

M.C.S.C. – contributed to data collection and project management, provided advise on bifactor modelling

G.P. – contributed to data collection and project management

For peer review only

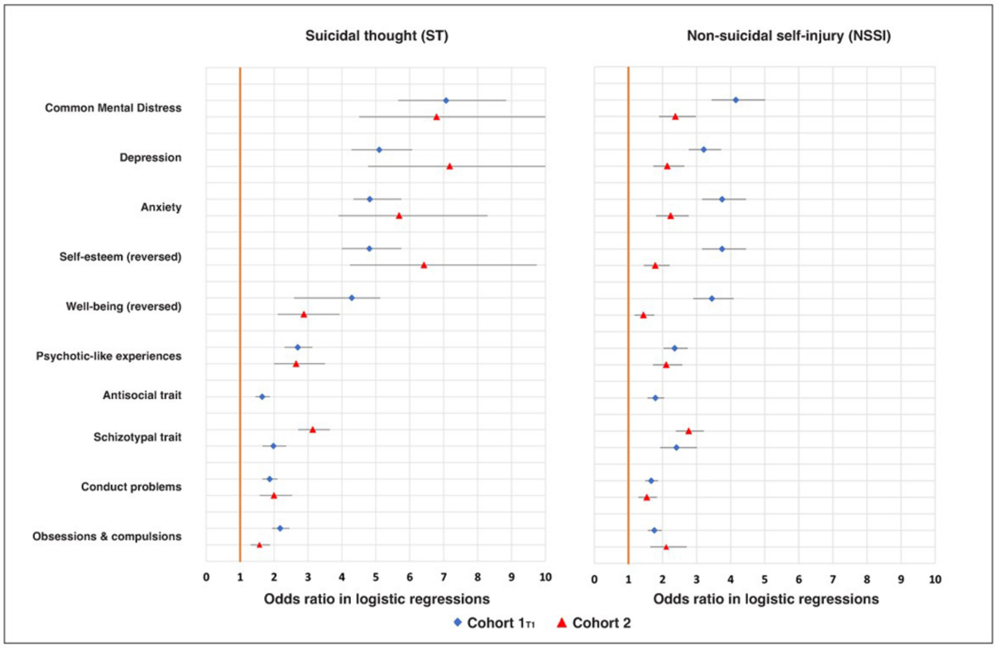

Figure 1: Odds ratio in logistic regressions for suicidal thoughts (ST) and non-suicidal self-harm (NSSI) as outcomes predicted by psychopathological predictors (listed on the left) here treated as continuous variables; regressions were computed separately for each predictor and effects of age and sex were controlled in each regression for in both cohorts (see Supplementary Table 2).

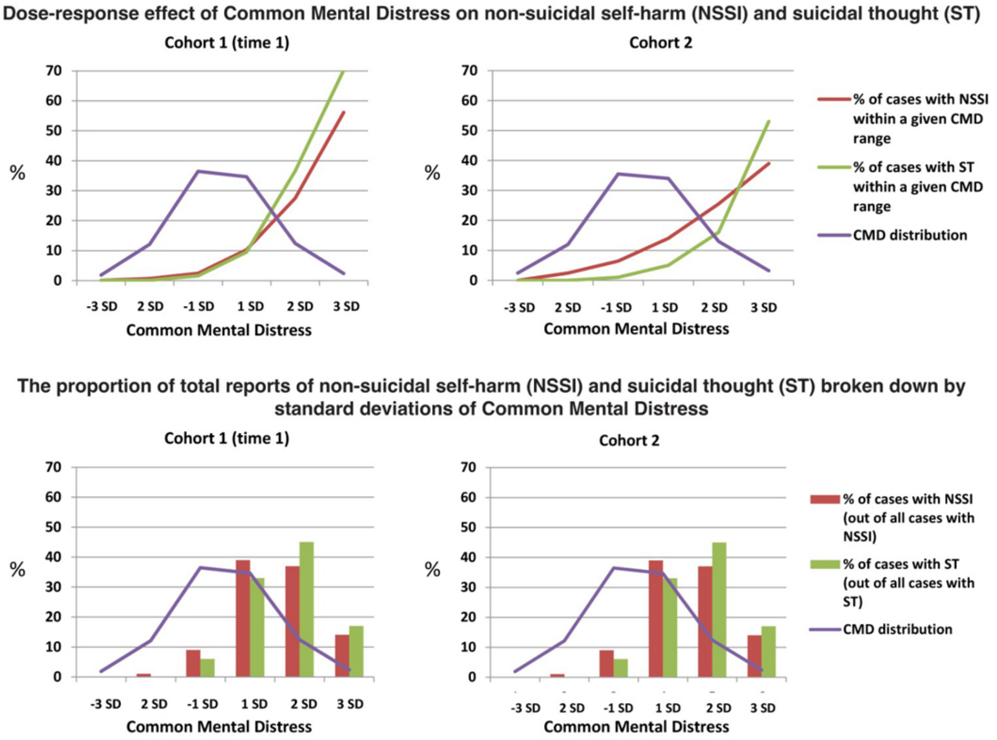

Figure 2: Upper panel shows the dose-response effect of Common Mental Distress on non-suicidal self-harm (NSSI) and suicidal thought (ST) in Cohort 1 and Cohort 2. The lower panel shows the proportion of total reports in non-suicidal self-injury (NSSI) and suicidal thought (ST) broken down by standard deviations of Common Mental Distress; these add up to 100% from left to right. The normal population distribution of CMD, which was strikingly similar, but not identical, in Cohort 1 and 2, is shown by the purple line (see density plots in Supplement, Figure 1).

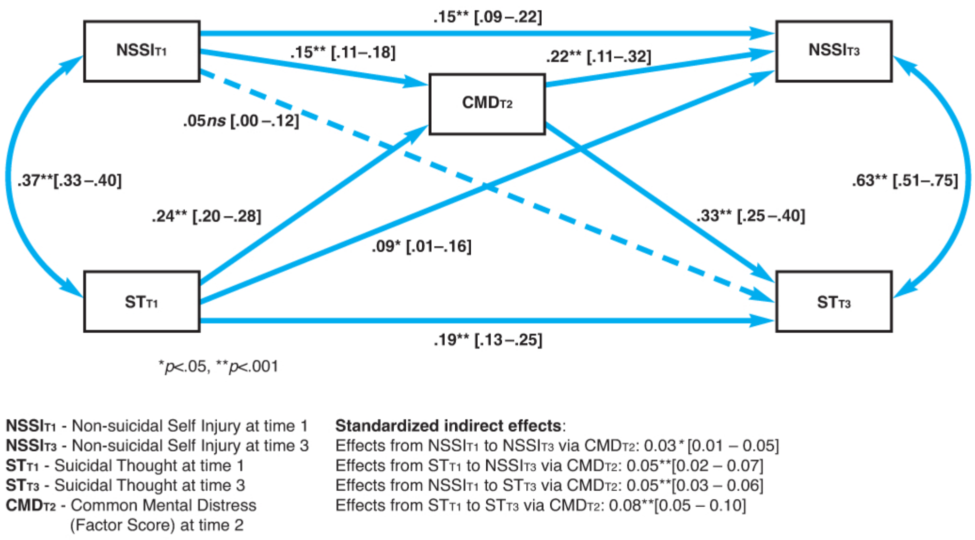

Figure 3: Mediation effect of Common Mental Distress at time 2 in Cohort 2: Standardised pathway coefficients with confidence intervals in square brackets.

Bifactor modelling:

Bifactor psychometric modelling is designed to extract variance common for all items in the model to generate one “general” factor. In addition to this general factor, specific factor/s may emerge, which are uncorrelated with each other or with the general factor. Specific factor/s contain the remaining variance after the extraction of the general factor[1]. St Clair et al. (2017) found in her psychometric study a bifactor model with one general factor and 5 specific factors, which fitted the data better than the correlated-factors model or second-order model. In our study, we first replicated St Clair et al. (2017) psychometric model in Cohort 1 (T1, T2, T3) and Cohort 2. In accordance with the original study, in our psychometric modelling the same measures of common mental illness frequently emerging during adolescence (depression, anxiety, psychotic experiences, obsessions and compulsions, conduct problems) as well as traits and characteristics commonly considered to contribute to mental wellness (well-being, self-esteem) were used as constructs contributing the general factor (see items below). Having replicated St Clair et al (2017) bifactor model, we then computed factor scores for the general factor – here termed *Common Mental Distress (CMD).*

The confirmatory bifactor analysis in Cohort 1 was computed with the multiple group method (MGM) in Mplus 8 with the three data point used as a grouping variable; the same model was fitted to the data in each group. MGM in Mplus by default holds thresholds and loadings invariant across groups[2], thus allowing the comparison if the model fits data well in all groups under study (here data from the three measurement points). The effective sample for the 3 data waves was, respectively, n=2403, n=1815, n=1245 (Total N=5463). The overall chi-square test for the model was $\chi2$=33648.24 (*df*=14983, *p*=0.000), for Time 1 it was $\chi2$=14791.20, for Time 2 it was $\chi2$=10400.56 and for Time 3 it was $\chi2$=8456.47. The overall Root Mean Square Error of Approximation (RMSEA) for the model was 0.026 (0.026-0.027), Comparative Fit Index (CFI) was 0.969, Tucker-Lewis Index (TLI) was 0.969, and weighted root mean square residual (WRMR) was 2.91. The confirmatory bifactor analysis was used in Cohort 2 as well. The following fit indexes were obtained in Cohort 2: $\chi2$=7602.17 (df=4462, *p*=0.000), RMSEA=0.026 (0.025-0.027), CFI=0.96, TLI=0.96, WRMR= 1.34. The above-cited fit indexes suggest that the bifactor model fitted the data well in both cohorts.

In both analyses – for Cohort 1 and 2 – we used WLSMV estimator and THETA parametrisation with PROBIT link, and all items were treated as ordered-categorical variables.

Much debate in the literature has focused on the issue of interpretability of specific factors, i.e., whether they should be considered as measures of meaningful concepts or should be treated as comprising the residual, uninterpretable variance[3]. The general factor in St Clair et al (2017) study demonstrated high reliability and validity, as well as low measurement error compared to validity and error of the specific factors. As follows, we focused in our study only on this general (CMD) factor; we did not attempt to interpret or use in our analyses the specific factors, even though they emerged in our bifactor modelling, due to their relatively high measurement error and ambiguity of their theoretical interpretation. The list of items contributing to CMD factor with factor loadings on this factor in Cohort 1 (T1, T2, T3) and Cohort 2 are listed below in Supplementary Table 1.

For peer review only - http://bmjopen.bmj.com/site/about/guidelines.xhtml

Multiple imputation procedure in Cohort 1:

Missingness in Cohort 1 predominantly arose from longitudinal attrition – 24% at T2 and 48% at T3; a small fraction of data was also missing due to omissions of items (between 0 to 6%). Before performing imputations, we examined if longitudinal attrition was related to demographic variables and other variables under study. Indeed, we found small, yet statistically significant correlations between attrition at T2 and T3 and demographic and exposure variables at T1 (see Supplementary Table 8), thus indicating that the assumption of "missing completely at random (MCAR) is not met. Moreover, we performed Little's MCAR test and found that it was significant ($p<.001$). Therefore, we assumed that MAR condition was met. As follows, we imputed missing data under MAR condition in Cohort 1 at T2 and T3 with the following variables in one imputation model: CMD factor scores, NSSI and ST variables. We used the following auxiliary variables: research points, sex, age, ethnicity, and Index of Multiple Deprivation (IMD) (as an indicator of a socioeconomic status[4]) as predictors of the missingness, in addition to main predictors – CMD factor scores, NSSI, and ST at T1.

Multiple imputations were computed in R program with MICE package[5]; convergence was examined by visual inspection of MCMC chains (with a maximum number of 20 iterations per chain and Gibbs sampling). Fifty-four (N=2403) datasets were generated to equal the percentage of missing data in CMD, NSSI, and ST at T3[6]. In terms of the imputation model, we used mean matching for continuous variables (CMD factor scores) and logistic regression for binary variables (NSSI and ST). The imputed 54 datasets were then used in pathway analysis (see the main manuscript and Supplementary Figure 3 for details) with MLM estimator in Mplus 7.4, which automates the process of analysing and combining parameter estimates from each imputed dataset using Rubin's rules[7].

For peer review only - http://bmjopen.bmj.com/site/about/guidelines.xhtml

Supplementary Table 1: List of all items used in the study

Outcome measures:

Suicidal Thought (ST)

I thought about killing myself (MFQ19, response options: *Always, Mostly, Sometimes, Never*)[Cohort 1 & 2]

This is one of the 4 items assessing suicidal thoughts in the 33-item Mood and Feelings Questionnaire (MFQ)[8] : MFQ16 - I thoughts that life was not worth living; MFQ17 - I thought about dying; MFQ18 – I thought my family would be better off without me; MFQ19 - I thought about killing myself. We used item 19, as it had the highest (.70) loading on this sub-subscale. Responses to this item were recoded into a binary format: no ST (original response option *Never*) and ST (original response options *Sometimes* or *Mostly* or *Always*). We did not include MFQ items 16-18 in CMD factor to avoid content overlap between the outcome measure (ST) and the predictor – the CMD factor.

Non-Suicidal Self-Injury (NSSI)

NSSI in Cohort 1 was assessed with one question from the Drug, Alcohol and Self-Injury (DASI) questionnaire asking about engaging in self-injury without suicidal intent during the last month:

In the last month, have you tried to hurt yourself on purpose without trying to kill yourself? *(*Response options: *Yes, No)*

NSSI in Cohort 2 was assessed with one question from the DASI questionnaire asking about life-time occurrence of NSSI:

Supplementary Table 9: Items comprising the Common Mental Distress (CMD) factor

**Items and associated measures**	**Standardised Factor Loadings**			
**The Moods and Feelings Questionnaire (MFQ)[11 Cohort 1 & 2]** **(response options: *Always, Mostly, Sometimes, Never*)** *Note:* 4 items measuring suicidality were excluded to avoid content overlap between the measures of variables treated here as predictors (CMD, Depression) and the outcome variable (ST). We excluded 4 other items which caused model convergence problems: *I was less hungry than usual (MFQ3), I ate more than usual (MFQ4), It was hard for me to make up my mind (MFQ10), I slept a lot more than usual (MFQ33)*	**Cohort 1**			**Cohort 2**
	**Time 1**	**Time 2**	**Time 3**	
1. I felt miserable or unhappy. (MFQ1)	.69	.73	.71	.73
2. I didn't enjoy anything. (MFQ2)	.62	.70	.72	.67
3. I felt so tired I just sat around and did nothing. (MFQ5)	.53	.56	.57	.54

For peer review only

For peer review only - http://bmjopen.bmj.com/site/about/guidelines.xhtml

4. I was moving and walking more slowly than usual. (MFQ6)	.54	.59	.54	.52
5. I was very restless. (MFQ7)	.48	.54	.56	.49
6. I felt I was no good any more. (MFQ8)	.78	.82	.84	.77
7. I sometimes blamed myself for things that weren't my fault. (MFQ9)	.70	.74	.75	.73
8. I got grumpy and cross easily. (MFQ11)	.60	.65	.68	.65
9. I felt like talking a lot less than usual. (MFQ12)	.64	.66	.69	.65
10. I was talking more slowly than usual. (MFQ13)	.56	.64	.55	.59
11. I cried a lot. (MFQ14)	.64	.64	.68	.69
12. I thought there was nothing good for me in the future. (MFQ15)	.72	.77	.78	.72
13. I didn't want to see my friends. (MFQ20)	.69	.73	.70	.66
14. I found it hard to think properly or concentrate. (MFQ21)	.73	.77	.77	.72
15. I thought bad things would happen to me. (MFQ22)	.76	.77	.80	.81
16. I hated myself. (MFQ23)	.81	.82	.85	.80
17. I was a bad person. (MFQ24)	.73	.76	.78	.72
18. I thought I looked ugly. (MFQ25)	.65	.70	.70	.69
19. I worried about aches and pains. (MFQ26)	.46	.50	.50	.56
20. I felt lonely. (MFQ27)	.70	.74	.73	.74
21. I thought nobody really loved me. (MFQ28)	.75	.79	.83	.76
22. I didn't have any fun at school / college / work. (MFQ29)	.62	.67	.66	.58
23. I thought I could never be as good as other people my age. (MFQ30)	.76	.79	.78	.76
24. I did everything wrong. (MFQ31)	.83	.85	.87	.82
25. I didn't sleep as well as usual. (MFQ32)	.53	.57	.61	.60
**The Revised Children's Manifest Anxiety Scale (RCMAS)[12] Cohort 1 & 2** **(response options: *Always, Mostly, Sometimes, Never*)**				
1. I had trouble making up my mind. (RCMAS1)	.60	.68	.71	.59
2. I worried when things did not go the right way for me. (RCMAS2)	.71	.77	.79	.78
3. Others seemed to do things more easily than I could. (RCMAS3)	.76	.80	.83	.76
4. Often I had trouble getting a breath. (RCMAS4)	.56	.60	.59	.55
5. I worried a lot of the time. (RCMAS5)	.78	.80	.82	.78
6. I was afraid of a lot of things. (RCMAS6)	.78	.80	.82	.77
7. I got angry easily. (RCMAS7)	.63	.68	.74	.68

For peer review only - http://bmjopen.bmj.com/site/about/guidelines.xhtml

8. I worried about what my parents would say to me. (RCMAS8)	.62	.67	.71	.65
9. I felt that others did not like the way I did things. (RCMAS9)	.73	.79	.78	.74
10. It was hard for me to get to sleep at night. (RCMAS10)	.55	.63	.58	.57
11. I worried about what other people thought about me. (RCMAS11)	.74	.79	.80	.71
12. I felt alone even when there were people with me. (RCMAS12)	.80	.84	.86	.85
13. Often I felt sick to my stomach. (RCMAS13)	.69	.74	.74	.76
16. I was tired a lot. (RCMAS16)	.62	.67	.69	.65
17. I worried about what was going to happen. (RCMAS17)	.77	.80	.81	.79
18. Other people my age were happier than me. (RCMAS18)	.79	.83	.83	.79
19. I had bad dreams. (RCMAS19)	.54	.59	.57	.62
20. My feelings got hurt easily when I was fussed at. (RCMAS20)	.75	.76	.78	.77
21. I felt someone would tell me I did things the wrong way. (RCMAS21)	.70	.77	.77	.71
22. I wake up scared some of the time. (RCMAS22)	.64	.74	.72	.67
23. I worried when I went to bed at night. (RCMAS23)	.67	.74	.73	.75
24. It was hard for me to keep my mind on my work. (RCMAS24)	.48	.58	.56	.55
25. I wiggled in my seat a lot. (RCMAS25)	.77	.79	.80	.76
27. A lot of people were against me. (RCMAS27)	.75	.80	.83	.80
28. I often worried about something bad happening to me. (RCMAS28)	.74	.79	.79	.80
**The Revised Leyton Obsessional Inventory (R-LOI)**[13] **Cohort 1 & 2** **(response options: *Always, Mostly, Sometimes, Never*)**				
1. I felt I had to do things in a certain way, like counting or saying special words, to stop something bad from happening. (R-LOI1)	.53	.58	.50	.47
2. I had trouble finishing my homework or other jobs because I had to do things over and over again. (R-LOI2)	.58	.63	.64	.53
3. I hated dirt and dirty things. (R-LOI3)	.35	.44	.43	.39
4. I had a special number that I counted up to, or I felt I had to do things just that number of times. (R-LOI4)	.40	.46	.42	.41
5. I often felt guilty or bad about things I had done even though no one else thought I had done anything wrong. (R-LOI5)	.71	.77	.79	.73
6. I worried about being clean enough. (R-LOI6)	.48	.51	.55	.45

For peer review only - http://bmjopen.bmj.com/site/about/guidelines.xhtml

7. I moved or talked in a special way to avoid bad luck. (R-LOI7)	.38	.46	.38	.33
8. I worried a lot if I did something, not exactly the way I liked. (R-LOI8)	.60	.67	.66	.53
9. I was fussy about keeping my hands clean. (R-LOI9)	.35	.40	.41	.35
10. I had special numbers or words that I said because I hoped they kept bad luck or bad things away. (R-LOI10)	.43	.47	.47	.42
11. I kept thinking about the things that I had done because I wasn't sure that they were the right things to do. (R-LOI11)	.71	.73	.71	.67

For peer review only

For peer review only - http://bmjopen.bmj.com/site/about/guidelines.xhtml

**Antisocial Behaviour Questionnaire (ABQ)[14] Cohort 1 & 2** **(response options: *Always, Mostly, Sometimes, Never*)**				
1. I deliberately broke the rules or disobeyed people (e.g. parents, teachers or supervisors). (ABQ1)	.45	.48	.47	.38
2. I stole things (e.g. from home or a shop or school). (ABQ2)	.37	.40	.36	.26
3. I deliberately damaged property (e.g. broke windows or chairs or wrote graffiti or started fires). (ABQ3)	.35	.39	.39	.38
4. I skipped lessons/work, skived, or played truant from school. (ABQ5)	.36	.39	.40	.35
5. I deliberately lied or cheated to get what I wanted. (ABQ6)	.43	.39	.41	.40
6. I ran away from home (e.g. for half a day or overnight). (ABQ7)	.51	.56	.58	.56
**Rosenberg Self-Esteem Questionnaire (RSEQ)[15] Cohort 1 & 2** **(response options: *Always, Mostly, Sometimes, Never*)**				
1. At times, I thought I was no good at all. (RSEQ1)	.82	.84	.85	.83
2. I was satisfied with myself. (RSEQ2)	-.58	-.61	-.60	-.53
3. I felt I had a number of good qualities. (RSEQ3)	-.53	-.55	-.56	-.52
4. I was able to do things as well as most people. (RSEQ4)	-.56	-.60	-.62	-.56
5. I felt I did not have much to be proud of. (RSEQ5)	.70	.73	.72	.70
6. I certainly felt useless at times. (RSEQ6)	.79	.81	.79	.77
7. I felt that I was as good as anyone else. (RSEQ7)	-.53	-.56	-.54	-.44
8. I wished I could have more respect for myself. (RSEQ8)	.62	.66	.68	.69
9. I felt that I was a failure. (RSEQ9)	.80	.82	.83	.75
10. I took a positive attitude toward myself. (RSEQ10)	-.60	-.63	-.63	-.56
**Warwick-Edinburgh Mental Well-Being Scale (WEMWBS)[16] Cohort 1 & 2** **(response options: *None of the time, Rarely, Some of the time, Often, All of the time*)**				
1. I've been feeling optimistic about the future. (WEMWBS1)	-.46	-.51	-.54	-.25
2. I've been feeling useful. (WEMWBS2)	-.52	-.58	-.60	-.33
3. I've been feeling relaxed. (WEMWBS3)	-.57	-.62	-.63	-.49
4. I've had the energy to spare. (WEMWBS5)	-.40	-.46	-.49	-.36
5. I've been dealing with problems well. (WEMWBS6)	-.57	-.63	-.64	-.46
6. I've been thinking clearly. (WEMWBS7)	-.62	-.67	-.68	-.48
7. I've been feeling good about myself. (WEMWBS8)	-.65	-.71	-.70	-.55
8. I've been feeling close to other people. (WEMWBS9)	-.44	-.50	-.52	-.28

For peer review only

9. I've been feeling confident. (WEMWBS10)	-.58	-.63	-.66	-.46
10. I've been able to make up my own mind about things. (WEMWBS11)	-.52	-.59	-.60	-.39
11. I've been feeling loved. (WEMWBS12)	-.49	-.54	-.60	-.29
12. I've been interested in new things. (WEMWBS13)	-.36	-.45	-.46	-.20
13. I've been feeling cheerful. (WEMWBS14)	-.61	-.67	-.67	-.49
**Psychotic-Like Experiences:**<br>**Cohort 1 – selected 10 items from the Schizotypal Personality Questionnaire (SPQ)**[17]<br>**Cohort 2 – selected 7 items from the Diagnostic Interview Schedule for Children (DISC)**[18]<br>**(response options: *Yes, No*)**				
1. Have you often mistaken objects or shadows for people or noises for voices? (SPQ4) **Cohort 1**	.38	.43	.41	*Not used*
2. I am sure I am being talked about behind my back. (SPQ9, DISC3) **Cohort 1 & 2**	.59	.67	.66	.60
3. Have you ever had the sense that some person or force is around you, even though you cannot see anyone? (SPQ13, DISC5) **Cohort 1 & 2**	.33	.38	.34	.41
4. Have you ever noticed a common event or object that seemed to be a special sign for you? (SPQ28, DISC8) **Cohort 1 & 2**	.33	.33	.35	.38
5. I often hear a voice speaking my thoughts aloud. (SPQ31, DISC10) **Cohort 1 & 2**	.33	.39	.34	.40
6. Have you ever seen things invisible to other people? (SPQ40, DISC13) **Cohort 1 & 2**	.36	.50	.37	.48
7. Do you sometimes feel that other people are watching you? (SPQ60, DISC19) **Cohort 1 & 2**	.53	.55	.59	.54
8. Do you ever suddenly feel distracted by distant sounds that you are not normally aware of? (SPQ61) **Cohort 1**	.40	.49	.45	*Not used*
9. Do you sometimes feel that people are talking about you? (SPQ63, DISC15) **Cohort 1 & 2**	.52	.56	.59	.60
10. Are your thoughts sometimes so strong that you can almost hear them? (SPQ64) **Cohort 1**	.44	.52	.50	*Not used*

For peer review only - http://bmjopen.bmj.com/site/about/guidelines.xhtml

Supplementary Table 2: Predictive power of Common Mental Distress versus the conventional psychopathology dimensions in Cohort 1_{T1} and Cohort 2: AUC (for ST and NSSI as criteria) and ORs for continuous and binary predictors (with cut-off point of 1SD)

		AUC		Suicidal thought (ST)				Non-suicidal self-injury (NSSI)			
				Continuous predictor		Binary (1SD cut-off)		Continuous predictor		Binary (1SD cut-off)	
		ST	NSSI	OR	95% C.I.	OR	95% C.I.	OR	95% C.I.	OR	95% C.I.
Common Mental Distress	Cohort 1_{T1}	.87	.83	7.07	[5.66 - 8.84]	15.60	[11.56 - 21.06]	4.15	[3.44 - 5.01]	8.93	[6.63 - 12.03]
	Cohort 2	.88	.72	6.79	[4.51 - 10.21]	20.97	[6.47 - 67.92]	2.38	[1.90 - 2.98]	4.00	[2.55 - 6.28]
Depression	Cohort 1_{T1}	.88	.83	5.10	[4.28 - 6.07]	15.60	[11.56 - 21.06]	3.21	[2.77 - 3.72]	8.28	[6.15 - 11.14]
	Cohort 2	.88	.70	7.18	[4.77 - 10.80]	15.32	[8.52 - 27.57]	2.14	[1.73 - 2.64]	3.56	[2.32 - 5.46]
Anxiety	Cohort 1_{T1}	.85	.81	4.82	[4.04 - 5.75]	13.62	[10.11 - 18.34]	3.75	[3.16 - 4.45]	7.61	[5.67 - 10.22]
	Cohort 2	.86	.71	5.69	[3.90 - 8.29]	10.51	[5.89 - 18.73]	2.24	[1.81 - 2.77]	3.68	[2.39 - 5.67]
Self-esteem (reversed)	Cohort 1_{T1}	.85	.83	4.81	[4.00 - 5.79]	15.62	[11.49 - 21.23]	3.75	[3.16 - 4.45]	9.86	[7.28 - 13.35]
	Cohort 2	.87	.65	6.42	[4.24 - 9.74]	15.16	[8.32 - 27.62]	1.79	[1.45 - 2.21]	3.34	[2.20 - 5.07]
Well-being (reversed)	Cohort 1_{T1}	.82	.80	4.29	[3.59 - 5.13]	10.31	[8.06 - 13.19]	3.45	[2.90 - 4.09]	6.66	[4.93 - 8.99]
	Cohort 2	.78	.61	2.88	[2.11 - 3.93]	5.27	[3.01 - 9.24]	1.44	[1.18 - 1.76]	2.19	[1.40 - 3.42]
Psychotic-like experiences	Cohort 1_{T1}	.74	.73	2.70	[2.32 - 3.13]	4.94	[3.70 - 6.60]	2.36	[2.03 - 2.74]	4.03	[2.98 - 5.45]
	Cohort 2	.74	.71	2.65	[2.00 - 3.50]	6.78	[3.89 - 11.83]	2.11	[1.72 - 2.58]	4.11	[2.69 - 6.27]
Antisocial trait*	Cohort 1_{T1}	.64	.63	1.65	[1.45 - 1.88]	2.67	[1.96 - 3.63]	1.79	[1.56 - 2.05]	2.48	[1.78 - 3.47]
Schizotypal trait	Cohort 1_{T1}	.79	.78	3.14	[2.71 - 3.64]	6.26	[4.70 - 8.32]	2.77	[2.39 - 3.21]	6.08	[4.52 - 8.19]
	Cohort 2	.76	.72	1.98	[1.66 - 2.36]	5.66	[3.23 - 9.91]	2.41	[1.93 - 3.01]	4.45	[2.90 - 6.83]
Conduct problems	Cohort 1_{T1}	.69	.67	1.87	[1.66 - 2.10]	3.38	[2.52 - 4.52]	1.67	[1.49 - 1.87]	3.46	[2.54 - 4.71]
	Cohort 2	.68	.61	2.00	[1.58 - 2.53]	3.78	[2.16 - 6.63]	1.54	[1.29 - 1.84]	2.13	[1.36 - 3.34]
Obsessions & compulsions	Cohort 1_{T1}	.76	.72	2.18	[1.94 - 2.45]	5.74	[4.25 - 7.75]	1.76	[1.57 - 1.98]	3.55	[2.58 - 4.89]
	Cohort 2	.71	.63	1.57	[1.31 - 1.88]	4.16	[2.37 - 7.28]	2.11	[1.64 - 2.71]	2.75	[1.79 - 4.22]

** measures were available only for Cohort 1_{T1}*

For peer review only - http://bmjopen.bmj.com/site/about/guidelines.xhtml

Supplementary Table 3: Association between ST and demographic variables in Cohort 1 (T1, T2, T3) and Cohort 2 (polychoric correlations)

	ST Cohort 1			ST Cohort 2
	**T1**	**T2**	**T3**	
Socioeconomic status (IMD)	-.05	-.01	-.01	.02
Research centre (0-Cambridge, 1-London)	-.12	-.04	-.03	not applicable
Ethnicity (1-white; 0-other)	-.08	-.02	-.04	-0.01
Age	-.05	-.02	-.05	0.03
Gender (0-Female, 1-Male)	-.10	-.08	-.01	0.03

All p-values non-significant

Supplementary Table 4: Association between NSSI and demographic variables in Cohort 1 (T1, T2, T3) and Cohort 2 (polychoric correlations)

	Cohort 1			Cohort 2
	**T1**	**T2**	**T3**	
Socioeconomic status (IMD)	.00	.00	.02	-.01
Research centre (0-Cambridge, 1-London)	-.01	-.01	.00	not applicable
Ethnicity (1-white; 0-other)	.00	.00	.00	.00
Age	-.02	-.04	-.01	-.02
Gender (0-Female, 1-Male)	.05	-.23	.02	.08

All p-values non-significant

Supplementary Table 5: Association between CMD and demographic variables in Cohort 1 (T1, T2, T3) and Cohort 2 (polychoric correlations)

	Cohort 1			Cohort 2
	**T1**	**T2**	**T3**	
Socioeconomic status (IMD)	-.02	-.02	-.01	.02
Research centre (0-Cambridge, 1-London)	.07*	.01	.01	not applicable
Ethnicity (1-white; 0-other)	-.08**	-.04	-.04	.04
Age	.01	.01	.01	.01
Gender (0-Female, 1-Male)	-.15**	-.15*	-.11**	.20**

*$p<.01$, **$p<.001$

For peer review only - http://bmjopen.bmj.com/site/about/guidelines.xhtml

Supplementary Table 6: Test of change in the prevalence of NSSI in Cohort 1: frequency over three time points (chi-square test)

	T1	T2	T3
NSSI	223	199	197
No-NSSI	2180	2204	2206

Chi-square=2.22, *df*=2, *p*=0.32, Yates' chi-square =2.04, *p*=0.35

Supplementary Table 7: Test of change in the prevalence of ST in Cohort 1: frequency over three time points (chi-square test)

	T1	T2	T3
NSSI	243	274	281
No-NSSI	2160	2129	2122

Chi-square=3.45, *df*=2, *p*=0.17, Yates' chi-square =3.26, *p*=0.19

For peer review only

For peer review only - http://bmjopen.bmj.com/site/about/guidelines.xhtml

Supplementary Table 8: Association between attrition in Cohort 1 at T2 and T3 and other variables in the study (Spearman rho)

	Attrition Cohort 1	
T1 variables:	**T2**	**T3**
Socioeconomic status (IMD index)[#]	-.07**	-.05*
Research centre (0-Cambridge, 1-London)	.05*	.05*
Ethnicity (1-white; 0-other)	-.05*	-.05*
Age	.07**	.05*
Gender (0-Female, 1-Male)	.09**	.12**
NSSI	-.01	.00
ST	-.01	-.03
Common Mental Distress	.06*	.05*
Depression	.06**	.05*
Impulsivity	.10**	.14**
Anxiety	.04*	.04*
Self - esteem (reversed)	.07**	.06*
Well - being (reversed)	.06*	.05*
Psychotic - like experiences even coerced	.00	.01
Antisocial trait	.08**	.12**
Schizotypal trait	.04*	.03
Conduct problems	.10**	.13**
Obsessions & compulsions	.03	.03

***p*<.001, **p*<.01
[#]higher number indicated *lower* socioeconomic deprivation

For peer review only

Supplementary Table 9: Direct and indirect effects in mediation (pathway) models in a female (F), male (M) and total (T) sample

		Standardised				Non- standardised			
		Coeff.	S.E.	Lower 95% C.I.	Upper 95% C.I.	Coeff.	S.E.	Lower 95% C.I.	Upper 95% C.I.
**NSSI$_{T1}$->CMD$_{T2}$**	F	**.14*** **	.03	.09	.19	***.46****	.09	.30	.61
	M	**13*** **	.03	.07	.18	***.56****	.14	.32	.80
	T	**.15*** **	.02	.11	.18	***.53****	.07	.40	.66
**NSSI$_{T1}$->NSSI$_{T3}$**	F	**.16** **	.05	.07	.25	***.54*** **	.17	.25	.83
	M	**.14** **	.05	.05	.23	***.65*** **	.24	.25	1.05
	T	**.15** **	.03	.09	.22	***.58*** **	.14	.34	.82
**NSSI$_{T1}$->ST$_{T3}$**	F	**.07**	.05	.00	.16	***.27***	.17	-.01	.56
	M	**.04**	.05	-.03	.13	***.22***	.24	-.18	.62
	T	**.05**	.03	.00	.12	***.22***	.15	-.02	.47
**ST$_{T1}$->CMD$_{T2}$**	F	**.25*** **	.03	.19	.30	***.83****	.10	.66	1.00
	M	**.24*** **	.03	.18	.30	***.85****	.11	.65	1.05
	T	**.24*** **	.02	.20	.28	***.83****	.07	.70	.96
**ST$_{T1}$->NSSI$_{T3}$**	F	**.10* **	.05	.01	.20	***.38***	.19	.05	.70
	M	**.07**	.06	-.03	.17	***.25***	.23	-.13	.64
	T	**.19* **	.04	.13	.25	***.33***	.16	.06	.60
**ST$_{T1}$->ST$_{T3}$**	F	**.20*** **	.04	.13	.27	***.76****	.16	.49	1.03
	M	**.17*** **	.05	.08	.25	***.66****	.19	.33	.98
	T	**.19** **	.03	.13	.25	***.72****	.13	.50	.95
**CMD$_{T2}$-> NSSI$_{T3}$**	F	**.22*** **	.07	.11	.34	***.24*** **	.07	.11	.37
	M	**.21* **	.08	.07	.34	***.21****	.09	.06	.36
	T	**.22*** **	.06	.11	.32	***.22****	.06	.11	.34
**CMD$_{T2}$-> ST$_{T3}$**	F	**.32*** **	.05	.22	.41	***.35****	.07	.23	.47
	M	**.35*** **	.06	.25	.46	***.39****	.07	.26	.51
	T	**.33*** **	.04	.25	.40	***.35****	.05	.26	.45
**NSS$_{T1}$<->ST$_{T1}$**	F	**.40*** **	.02	.36	.45	***.04****	.00	.03	.04
	M	**.32*** **	.03	.26	.37	***.02****	.00	.01	.03
	T	**.37*** **	.02	.33	.40	***.03****	.00	.02	.03
**NSSI$_{T3}$<->ST$_{T3}$**	F	**.67*** **	.07	.55	.79	***.67****	.07	.55	.79
	M	**.57*** **	.10	.39	.75	***.57****	.10	.39	.75
	T	**.63*** **	.07	.51	.75	***.63****	.07	.51	.75
**NSSI$_{T1}$->CMD$_{T2}$ ->NSSI$_{T3}$**	F	**.03** **	.01	.01	.05	***.11*** **	.04	.04	.18
	M	**.02* **	.01	.00	.05	***.12****	.06	.02	.22

For peer review only - http://bmjopen.bmj.com/site/about/guidelines.xhtml

	T	**.03***	.01	.01	.05	***.12****	.04	.05	.19
**ST_{T1}->CMD_{T2}->$NSSI_{T3}$**	F	**.05****	.02	.02	.09	***.20****	.07	.08	.32
	M	**.05***	.02	.01	.08	***.18****	.08	.05	.31
	T	**.05****	.01	.02	.07	***.19*****	.05	.09	.28
**$NSSI_{T1}$->CMD_{T2}->ST_{T3}**	F	**.04*****	.01	.02	.06	***16*****	.04	.08	.24
	M	**.04*****	.01	.02	.07	***.22****	.07	.10	.33
	T	**.05****	.01	.03	.06	***.19*****	.04	.12	.26
**ST_{T1}->CMD_{T2}->ST_{T3}**	F	**.08*****	.01	.05	.11	***.29*****	.07	.17	.41
	M	**.08*****	.02	.05	.12	***.33*****	.08	.20	.47
	T	**.08****	.01	.05	.10	***.30*****	.05	.20	.39

Significance levels: *p<.05, **p<.01, ***p<.001

For peer review only - http://bmjopen.bmj.com/site/about/guidelines.xhtml

Item Response Theory (IRT) analysis

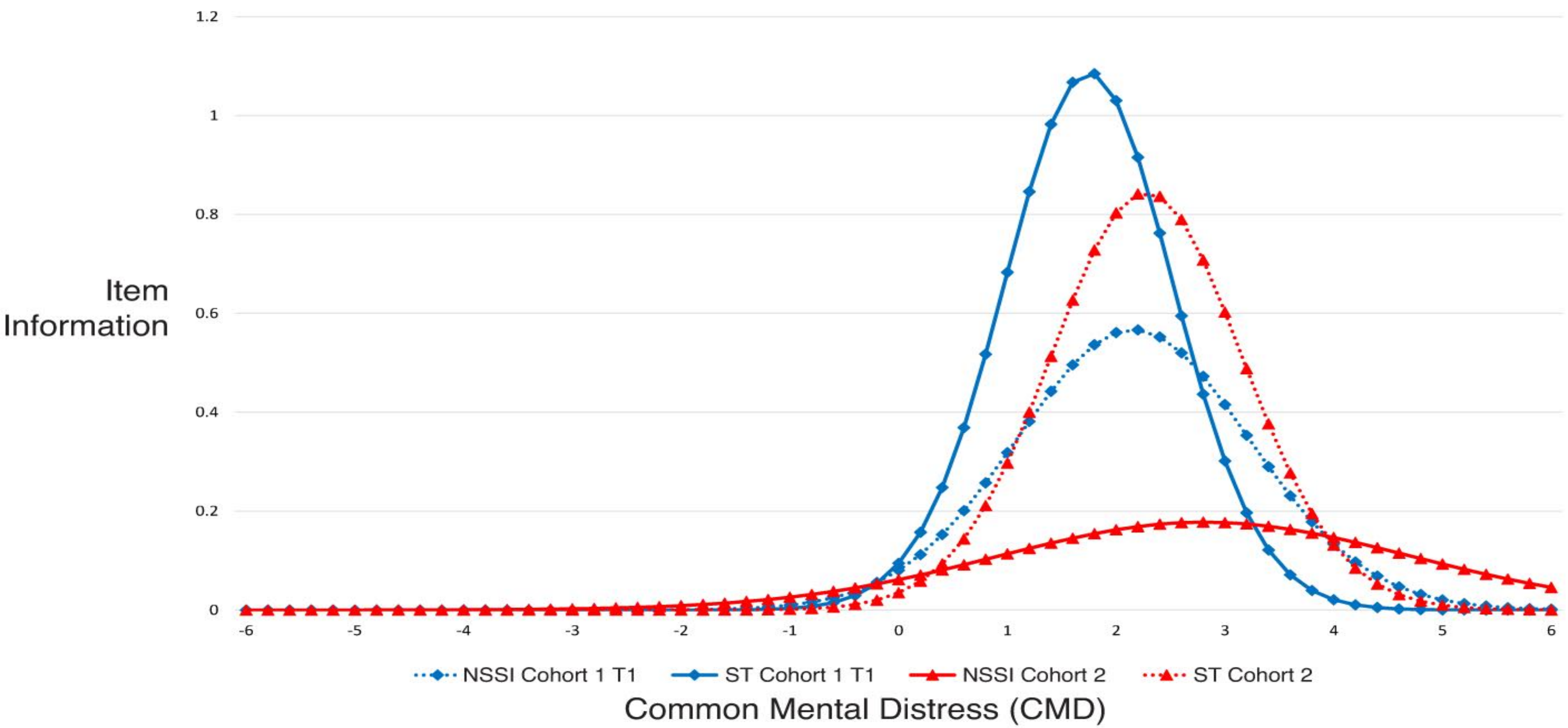

Supplementary Figure 1: Hierarchy of symptoms: the place of non-suicidal self-harm (NSSI) and suicidal though (ST) on the latent continuum of Common Mental Distress (in standard deviations) in Cohort 1_{T1} and Cohort 2.

Item Response Theory (IRT) analysis is concerned, broadly speaking, with investigating the relationship between items and the latent construct. Here we computed item response function showing how much information NSSI and ST (here treated as indicators of CMD) contribute to the latent variable – CMD. The above graph shows that NSSI and ST provided information in above-average to high ranges of CMD, with the peak of the information curves for NSSI occurring around +2 SD in both cohorts. The information curve for ST in Cohort 2 was flatter, suggesting less contribution to the latent CMD dimension than ST had in Cohort 1_{T1} dataset. This may be due to the differences in age structure and psychopathology status in both cohorts. Nonetheless, in both cohorts the peak in the ST curves occurred between +2 and +3 SD (high end of the CMD dimension), showing that ST lies on the more severe spectrum of CMD dimension than NSSI does.

For peer review only - http://bmjopen.bmj.com/site/about/guidelines.xhtml

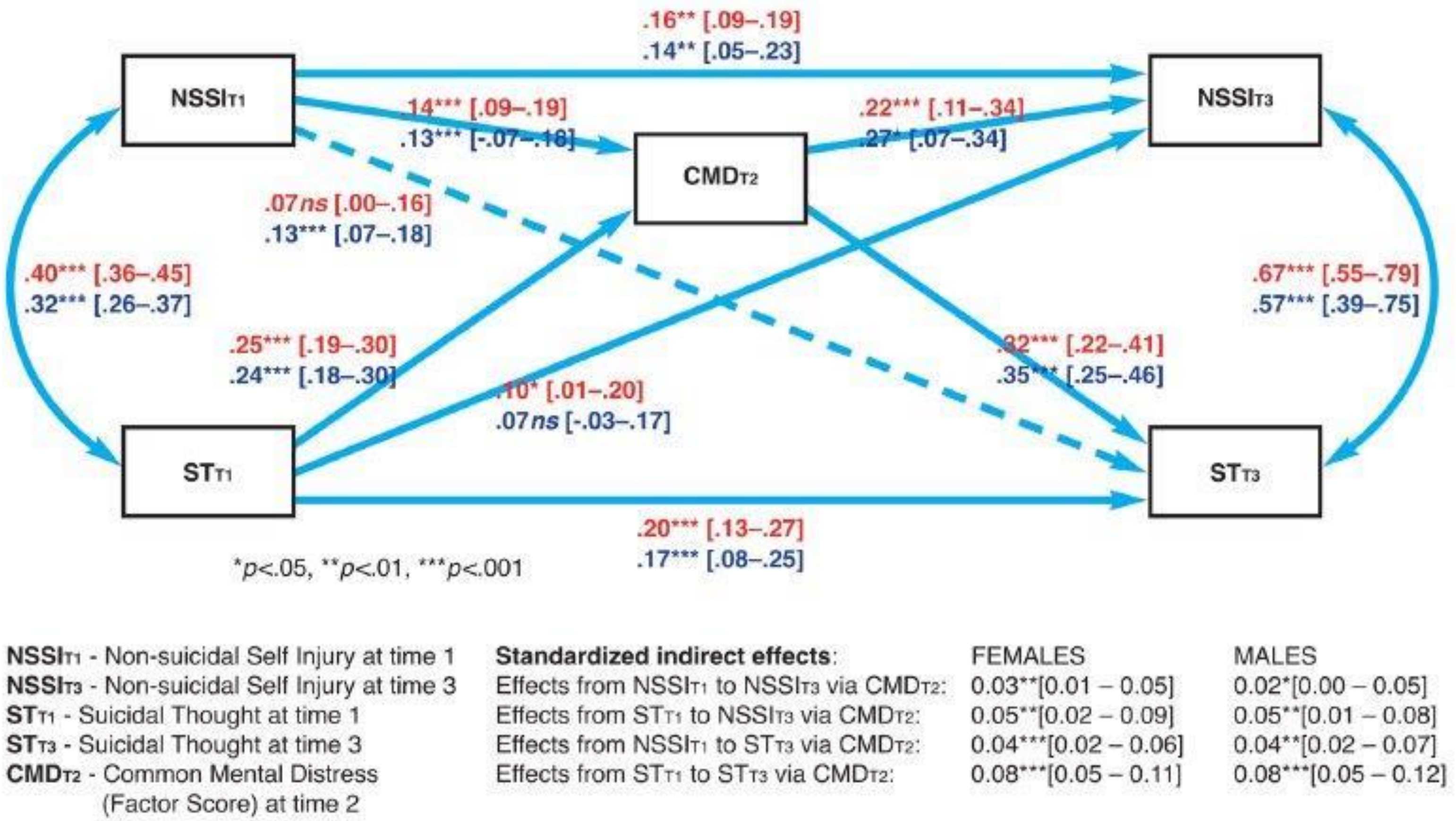

Supplementary Figure 2: Mediation effect of Common Mental Distress at time 2 (CMD_{T2}) moderated by sex (female n=1286 (red colour); male n=1115 (blue colour)) in the Cohort 1

Standardised pathway coefficients (with confidence intervals reported in squarer brackets) were obtained in multiple group pathway analysis in which sex was treated as a grouping variable. We tested the equivalence in pathway coefficients by means of comparing chi-square tests when the coefficient was "fixed" to be equal across sexes versus when it was free to vary across sexes[2]. We also tested the equivalence of fit indices of the model in both sexes. We found no evidence for differences in individual pathway coefficients or fit indices between sexes. This suggests that CMD at T2 mediated the longitudinal persistence of NSSI and ST in the same manner in females and males – no evidence of sex differences in the longitudinal mediation process was found. Additional details are reported in Supplementary Table 10.

Age and gender: Descriptive analysis

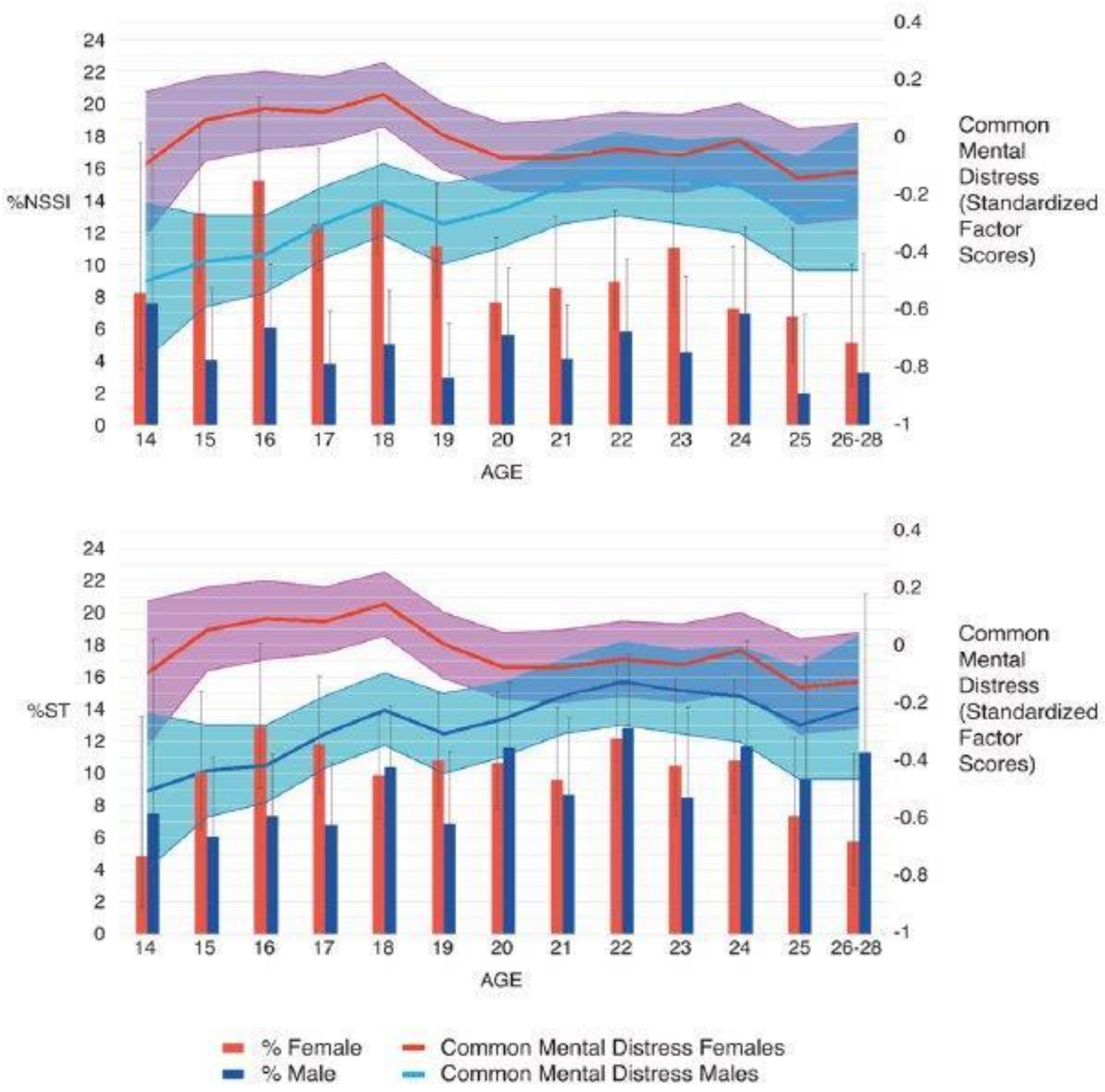

Supplementary Figure 3. Percentages of non-suicidal self-injury (NSSI), suicidal thoughts (ST) and levels of Common Mental Distress in age groups for both sexes in Cohort 1

To analyse the relationship between age, sex, NSSI, ST, and CMD descriptively, we grouped observations from all 3 time points in Cohort $1_{T1\text{-}T3}$ by age, rather than by data time point. This grouping allowed us to investigate levels of CMD, NSSI and ST in a broad age range of 14-28 years (note that this also entailed the inclusion of the same individuals from consecutive data sweeps (e.g., when an individual was 14, 15 and 16 years old) in the adjacent age groups). The histograms showing percentages of NSSI and ST with Wilson confidence intervals were plotted against the lines representing the means of CMD with confidence intervals for every age group for both sexes separately (Figure 3 above).

For peer review only - http://bmjopen.bmj.com/site/about/guidelines.xhtml

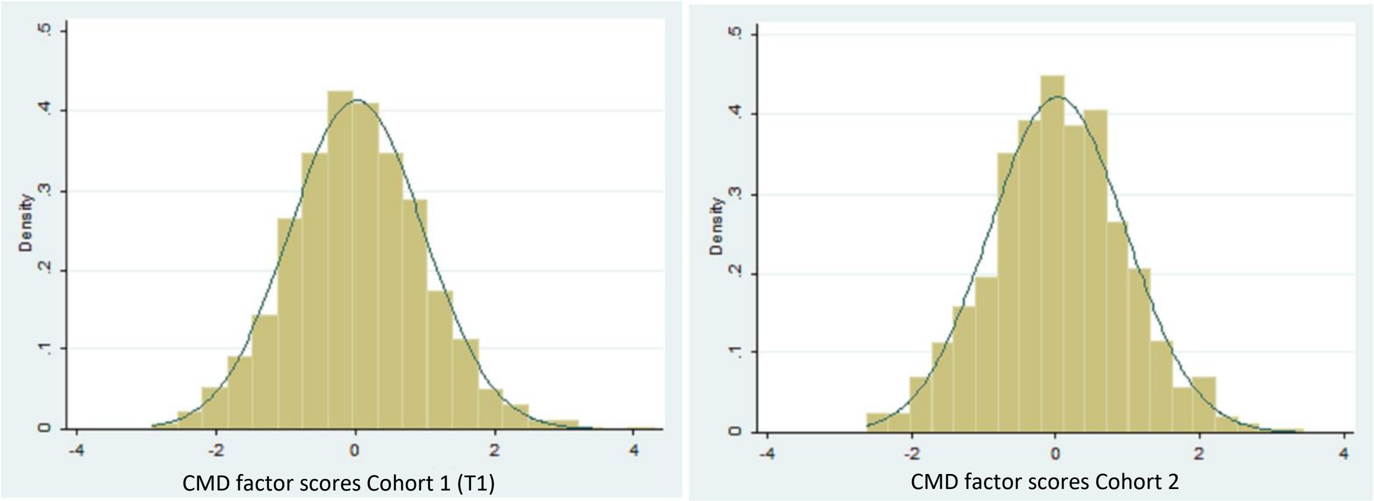

Supplementary Figure 4: Histograms of CMD factor scores in Cohort 1 (T1) and Cohort 2 with a schematic normal distribution line

For peer review only - http://bmjopen.bmj.com/site/about/guidelines.xhtml

Data collection tools:

Study data were collected and managed using REDCap electronic data capture tools[19] hosted at the University of Cambridge. REDCap (Research Electronic Data Capture) is a secure, web-based application designed to support data capture for research studies, providing 1) an intuitive interface for validated data entry; 2) audit trails for tracking data manipulation and export procedures; 3) automated export procedures for seamless data downloads to common statistical packages; and 4) procedures for importing data from external sources.

Group Information

NSPN (NeuroScience in Psychiatry Network: http://www.nspn.org.uk/) is a research consortium formed by the University of Cambridge and University College London, launched in November 2012 and supported by Wellcome Trust Award (095844/Z/11/Z). The group included the following members:

Principal investigators:

Edward Bullmore (CI from 01/01/2017)[1,2]

Ian Goodyer (CI until 01/01/2017)[1]

Peter Jones[1,2,3]

Raymond Dolan[4,5]

Peter Fonagy[6]

NSPN (funded) staff:

Michael Moutoussis[4,5]

Tobias Hauser[4,5]

Sharon Neufeld[1]

Petra Vértes[1,2]

Kirstie Whitaker[1,2]

Gita Prabhu[4,5]

Laura Villis[1]

Junaid Bhatti[1]

Becky Inkster[1]

Cinly Ooi[1]

Barry Widmer[1]

Ayesha Alrumaithi[1]

Sarah Birt[1]

For peer review only - http://bmjopen.bmj.com/site/about/guidelines.xhtml

Kalia Cleridou[5]

Hina Dadabhoy[5]

Sian Granville[5]

Elizabeth Harding[5]

Alexandra Hopkins[4,5]

Daniel Isaacs[5]

Janchai King[5]

Danae Kokorikou[5,6]

Harriet Mills[5]

Ciara O'Donnell[1]

Sara Pantaleone[5]

Aislinn Bowler[5]

Affiliated scientists:

Pasco Fearon[6]

Anne-Laura van Harmelen[1]

Rogier Kievit[4,7]

1 Department of Psychiatry, University of Cambridge, United Kingdom

2 NIHR Applied Research Collaboration East of England, UK

3 NIHR Cambridge Biomedical Research Centre, UK

4 Max Planck University College London Centre for Computational Psychiatry and Ageing Research, University College London, UK

5 Wellcome Centre for Human Neuroimaging, University College London, United Kingdom

6 Research Department of Clinical, Educational and Health Psychology, University College London,

United Kingdom

7 Medical Research Council Cognition and Brain Sciences Unit, University of Cambridge, United Kingdom

For peer review only

For peer review only - http://bmjopen.bmj.com/site/about/guidelines.xhtml

References:

1 Reise, S. P. The rediscovery of bifactor measurement models. *Multivariate Behavioural Research*. 2012;47:667-696, doi:10.1080/00273171.2012.715555.

2 Muthen, L. & Muthen, B. *Mplus Users's Guide*. (Muthen & Muthen, 1998-2002).

3 Reise, S. P., Moore, T. M. & Haviland, M. G. Bifactor models and rotations: Exploring the extent to which multidimensional data yield univocal scale scores. *Journal of Personality Assessment*. 2010;92:544-559, doi:10.1080/00223891.2010.496477.

4 Noble M, McLennan, D, Wilkinson K, Whitworth A, & Barne H. The English Indices of Deprivation 2007. London: Department for Communities and Local Government. (2008).

5 van Buuren, S. & Groothuis-Oudshoorn, K. MICE: Multivariate Imputation by Chained Equations in R. *Journal of Statistical Software*.2011;45,1-67.

6 Sterne, J. A. C. *et al.* Multiple imputation for missing data in epidemiological and clinical research: Potential and pitfalls. *British Medical Journal*. 2009;339:157-160.

7 Rubin, D. B. *Multiple imputation for nonresponse in surveys*. (Wiley, 1987).

8 Hammerton, G., Zammit, S., Potter, R., Thapar, A. & Collishaw, S. Validation of a composite of suicide items from the Mood and Feelings Questionnaire (MFQ) in offspring of recurrently depressed parents. . *Psychiatry Research*. 2014;216:82-88.

9 Wilkinson P.O., Qiu T., Neufeld S., Jones P.B. & Goodyer I.M. Sporadic and recurrent non-suicidal self-injury before age 14 and incident onset of psychiatric disorders by 17 years: prospective cohort study. *British Journal of Psychiatry*.2018;212:222-226, doi:10.1192/bjp.2017.45.

10 Cassels M. *et al.* Poor family functioning mediates the link between childhood adversity and adolescent non-suicidal self-injury. *Journal of Child Psychology and Psychiatry*. 2018;59(8):881-887. doi: 10.1111/jcpp.12866

11 Angold, A. *et al.* The development of a short questionnaire for use in epidemiological studies of depression in children and adolescents. *International Journal of Methods in Psychiatric Research*.1995;5:237-249.

12 Reynolds, C. R. Concurrent validity of what I think and feel: The revised children's manifest anxiety scale. *Journal of Consulting and Clinical Psychology*. 1980;48:774-775. doi:10.1037/0022-006x.48.6.774 (1980).

13 Bamber, D., Tamplin, A., Park, R. J., Kyte, Z. A. & Goodyer, I. M. Development of a short Leyton Obsessional Inventory For Children and Adolescents. *Journal of the American Academy of Child and Adolescent Psychiatry*.2002;41:1246-1252.

14 St Clair C. M. *et al.* Characterising the latent structure and organisation of self-reported thoughts, feelings and behaviours in adolescents and young adults. *PLOS One*. 2017;12:1-27, doi:https://doi.org/10.1371/journal.pone.0175381.

15 Rosenberg, M. *Society and the adolescent self-image*. (Princeton University Press, 1965).

16 Tennant, R. *et al.* The Warwick-Edinburgh mental well-being scale (WEMWBS): development and UK validation. *Health and Quality of Life Outcomes* **5**, doi:10.1186/1477-7525-5-63 (2007).

17 Raine, A. The SPQ: A scale for the assessment of schizotypal personality based on DSM-III-R criteria. *Schizophrenia Bulletin*.1991;17:555-564.

18 Shaffer, D., Fisher, P., Lucas, C. P., Dulcan, M. K. & Schwab-Stone, M. E. NIMH Diagnostic Interview Schedule for Children Version IV (NIMH DISC-IV): description, differences from previous versions, and reliability of some common diagnoses. *Journal of the American Academy of Child and Adolescent Psychiatry*. 2000;39:28-38.

19 Harris PA, Taylor R, Thielke R, Payne J, Gonzalez N, Conde JG. Research electronic data capture (REDCap) – A metadata-driven methodology and workflow process for providing translational research informatics support. *Journal of Biomedical Informatics*. 2009;42(2):377-81.

For peer review only - http://bmjopen.bmj.com/site/about/guidelines.xhtml

STROBE (Strengthening The Reporting of OBservational Studies in Epidemiology) Checklist

A checklist of items that should be included in reports of observational studies. You must report the page number in your manuscript where you consider each of the items listed in this checklist. If you have not included this information, either revise your manuscript accordingly before submitting or note N/A.

Note: An Explanation and Elaboration article discusses each checklist item and gives methodological background and published examples of transparent reporting. The STROBE checklist is best used in conjunction with this article (freely available on the Web sites of PLoS Medicine at http://www.plosmedicine.org/, Annals of Internal Medicine at http://www.annals.org/, and Epidemiology at http://www.epidem.com/). Information on the STROBE Initiative is available at www.strobe-statement.org.

Section and Item	Item No.	Recommendation	Reported on Page No.
**Title and Abstract**	1	(*a*) Indicate the study's design with a commonly used term in the title or the abstract	1
		(*b*) Provide in the abstract an informative and balanced summary of what was done and what was found	2-3
**Introduction**			
Background/Rationale	2	Explain the scientific background and rationale for the investigation being reported	4-5
Objectives	3	State specific objectives, including any prespecified hypotheses	5
**Methods**			
Study Design	4	Present key elements of study design early in the paper	5-6
Setting	5	Describe the setting, locations, and relevant dates, including periods of recruitment, exposure, follow-up, and data collection	6-7
Participants	6	(*a*) *Cohort study*—Give the eligibility criteria, and the sources and methods of selection of participants. Describe methods of follow-up	6-7
		Case-control study—Give the eligibility criteria, and the sources and methods of case ascertainment and control selection. Give the rationale for the choice of cases and controls	not applicable
		Cross-sectional study—Give the eligibility criteria, and the sources and methods of selection of participants	not applicable
		(*b*) *Cohort study*—For matched studies, give matching criteria and number of exposed and unexposed	not applicable
		Case-control study—For matched studies, give matching criteria and the number of controls per case	not applicable
Variables	7	Clearly define all outcomes, exposures, predictors, potential confounders, and effect modifiers. Give diagnostic criteria, if applicable	7-8

For peer review only

Section and Item	Item No.	Recommendation	Reported on Page No.
Data Sources/ Measurement	8*	For each variable of interest, give sources of data and details of methods of assessment (measurement). Describe comparability of assessment methods if there is more than one group	Suppl. 3-8
Bias	9	Describe any efforts to address potential sources of bias	Suppl. 2, Ms.1
Study Size	10	Explain how the study size was arrived at	Not applicabl
Quantitative Variables	11	Explain how quantitative variables were handled in the analyses. If applicable, describe which groupings were chosen and why	Supp.1, Ms. 7-8
Statistical Methods	12	(*a*) Describe all statistical methods, including those used to control for confounding	Supp.1, Ms. 7-8
		(*b*) Describe any methods used to examine subgroups and interactions	Ms. 7-8
		(*c*) Explain how missing data were addressed	Suppl. 2
		(*d*) *Cohort study*—If applicable, explain how loss to follow-up was addressed	Suppl. 2
		Case-control study—If applicable, explain how matching of cases and controls was addressed	not applicable
		Cross-sectional study—If applicable, describe analytical methods taking account of sampling strategy	not applicable
		(*e*) Describe any sensitivity analyses	not applicable
**Results**			
Participants	13*	(a) Report numbers of individuals at each stage of study—eg numbers potentially eligible, examined for eligibility, confirmed eligible, included in the study, completing follow-up, and analysed	6
		(b) Give reasons for non-participation at each stage	not applicable
		(c) Consider use of a flow diagram	not applicable
Descriptive Data	14*	(a) Give characteristics of study participants (eg demographic, clinical, social) and information on exposures and potential confounders	Suppl.11, Ms.6
		(b) Indicate number of participants with missing data for each variable of interest	Suppl. 2
		(c) *Cohort study*—Summarise follow-up time (eg, average and total amount)	6
Outcome Data	15*	*Cohort study*—Report numbers of outcome events or summary measures over time	9
		Case-control study—Report numbers in each exposure category, or summary measures of exposure	not applicable
		Cross-sectional study—Report numbers of outcome events or summary measures	not applicable

For peer review only

Section and Item	Item No.	Recommendation	Reported on Page No.
Main Results	16	(*a*) Give unadjusted estimates and, if applicable, confounder-adjusted estimates and their precision (eg, 95% confidence interval). Make clear which confounders were adjusted for and why they were included	Fig. 1, Suppl.9
		(*b*) Report category boundaries when continuous variables were categorized	Suppl. 9
		(*c*) If relevant, consider translating estimates of relative risk into absolute risk for a meaningful time period	Fig. 2
Other Analyses	17	Report other analyses done—eg analyses of subgroups and interactions, and sensitivity analyses	Suppl.9, 16
**Discussion**			
Key Results	18	Summarise key results with reference to study objectives	10-11
Limitations	19	Discuss limitations of the study, taking into account sources of potential bias or imprecision. Discuss both direction and magnitude of any potential bias	12
Interpretation	20	Give a cautious overall interpretation of results considering objectives, limitations, multiplicity of analyses, results from similar studies, and other relevant evidence	13-14
Generalisability	21	Discuss the generalisability (external validity) of the study results	13-14
**Other Information**			
Funding	22	Give the source of funding and the role of the funders for the present study and, if applicable, for the original study on which the present article is based	15

*Give information separately for cases and controls in case-control studies and, if applicable, for exposed and unexposed groups in cohort and cross-sectional studies.

Once you have completed this checklist, please save a copy and upload it as part of your submission. DO NOT include this checklist as part of the main manuscript document. It must be uploaded as a separate file.

For peer review only

BMJ Open

How do the prevalence and relative risk of non-suicidal self-injury and suicidal thoughts vary across the population distribution of common mental distress (the p-factor)? Observational analyses replicated in two independent UK cohorts of young people

Journal:	*BMJ Open*
Manuscript ID	bmjopen-2019-032494.R3
Article Type:	Original research
Date Submitted by the Author:	01-Mar-2020
Complete List of Authors:	Polek, Ela; University of Cambridge, Psychiatry; University College Dublin, Psychology<br>Neufeld, Sharon A. S.; Univ Cambridge<br>Wilkinson, Paul; University of Cambridge, Cambridge Neuroscience<br>Goodyer, Ian; Cambridge University, Psychiatry<br>St Clair, Michelle<br>Prabhu, Gita<br>Dolan, Ray<br>Bullmore, Edward<br>Fonagy, Peter<br>Stochl, Jan; University of Cambridge, Department of Psychiatry; NIHR Collaboration for Leadership in Applied Health Research & Care (CLAHRC) East of England,<br>Jones, Peter; University of Cambridge, Department of Psychiatry
**Primary Subject Heading**:	Public health
Secondary Subject Heading:	Mental health
Keywords:	EPIDEMIOLOGY, Child & adolescent psychiatry < PSYCHIATRY, PUBLIC HEALTH, Suicide & self-harm < PSYCHIATRY

SCHOLARONE™
Manuscripts

I, the Submitting Author has the right to grant and does grant on behalf of all authors of the Work (as defined in the below author licence), an exclusive licence and/or a non-exclusive licence for contributions from authors who are: i) UK Crown employees; ii) where BMJ has agreed a CC-BY licence shall apply, and/or iii) in accordance with the terms applicable for US Federal Government officers or employees acting as part of their official duties; on a worldwide, perpetual, irrevocable, royalty-free basis to BMJ Publishing Group Ltd ("BMJ") its licensees and where the relevant Journal is co-owned by BMJ to the co-owners of the Journal, to publish the Work in this journal and any other BMJ products and to exploit all rights, as set out in our licence.

The Submitting Author accepts and understands that any supply made under these terms is made by BMJ to the Submitting Author unless you are acting as an employee on behalf of your employer or a postgraduate student of an affiliated institution which is paying any applicable article publishing charge ("APC") for Open Access articles. Where the Submitting Author wishes to make the Work available on an Open Access basis (and intends to pay the relevant APC), the terms of reuse of such Open Access shall be governed by a Creative Commons licence – details of these licences and which Creative Commons licence will apply to this Work are set out in our licence referred to above.

Other than as permitted in any relevant BMJ Author's Self Archiving Policies, I confirm this Work has not been accepted for publication elsewhere, is not being considered for publication elsewhere and does not duplicate material already published. I confirm all authors consent to publication of this Work and authorise the granting of this licence.

For peer review only

How do the prevalence and relative risk of non-suicidal self-injury and suicidal thoughts vary across the population distribution of common mental distress (the p-factor)? Observational analyses replicated in two independent UK cohorts of young people

Ela Polek[1,9], Sharon Neufeld[1], Paul Wilkinson [1], Ian M. Goodyer[1], Michelle C. St Clair[2], Gita Prabhu[3], Ray Dolan[3,7], Ed Bullmore[1,5], Peter Fonagy[4], Jan Stochl[1,5,8] & Peter B. Jones[1,5,6]

[1] Department of Psychiatry, University of Cambridge, UK

[2] Department of Psychology, University of Bath, UK

[3] Wellcome Centre for Human Neuroimaging, University College London, UK

[4] Division of Psychology and Language Sciences, University College London, UK

[5] NIHR Applied Research Collaboration East of England, UK

[6] NIHR Cambridge Biomedical Research Centre, UK

[7] Max Planck UCL Centre for Computational Psychiatry and Ageing Research, UK

[8] Department of Kinanthropology, Charles University, Czech Republic

[9] School of Psychology, University College Dublin

Correspondence to:

Professor Peter B. Jones

Herchel Smith Building

Cambridge Biomedical Campus

CB2 0SZ

UK

Fax:01223 336581

e-mail: pbj21@cam.ac.uk

For peer review only - http://bmjopen.bmj.com/site/about/guidelines.xhtml

For peer review only

the total word count: 6026

Abstract:

Objectives: To inform suicide prevention policies and responses to youths at risk by investigating whether suicide risk is predicted by a summary measure of common mental distress (CMD, (the p-factor)) as well as by conventional psychopathological domains; to define the distribution of suicide risks over the population range of CMD; to test whether such distress mediates the medium-term persistence of suicide risks.

Design: Two independent population-based cohorts.

Setting: Population-based in two UK centres.

Participants: Volunteers age 14-24 years recruited from primary health care registers, schools and colleges, with advertisements to complete quotas in age-sex-strata. Cohort 1 is the Neuroscience in Psychiatry Network (NSPN; N=2403); Cohort 2 is the ROOTS sample (N=1074).

Primary outcome measures: Suicidal thoughts (ST) and non-suicidal self-injury (NSSI).

Results: We calculated a CMD score using confirmatory bifactor analysis and then used logistic regressions to determine adjusted associations between risks and CMD; curve-fitting was used to examine the relative prevalence of suicidal thoughts (ST) and non-suicidal self-injury (NSSI) over the population distribution of CMD. We found a dose-response relationship between levels of CMD and risk of suicide. The majority of all subjects experiencing ST and NSSI (78% and 76% in Cohort 1, and 66% and 71% in Cohort 2) had CMD scores no more than two standard deviations above the population mean; higher scores indicated the highest risk but were, by definition, infrequent. Pathway mediation models showed that CMD mediated the longitudinal course of both ST and NSSI.

Conclusions. NSSI and ST in youths reflect common mental distress that also mediates their persistence. Universal prevention strategies reducing levels of CMD in the whole population

For peer review only - http://bmjopen.bmj.com/site/about/guidelines.xhtml

without recourse to screening or measurement may prevent more suicides than approaches targeting youths with the most severe distress or with psychiatric disorders.

Strengths and limitations:

- The samples were population-based with several self-reported outcomes regarding suicidal risk.
- Replication of the findings in two independent cohorts strengthens confidence in the findings.
- Results were robust across different statistical models and approaches to data classification.
- Sample attrition was a limitation in both cohorts.
- Multiple imputations mitigated biases arising from attrition.

For peer review only

Introduction

Adolescence sees the onset of a range of psychopathology including suicidal thoughts (ST) and non-suicidal self-injury (NSSI)[1-3] that individually or together convey heightened risk of suicide attempts[4-6]. Non-suicidal and suicidal self-harm predict completed suicide[7], the second most common cause of deaths among 10 to 24-year-olds, worldwide[8]. Moreover, ST and NSSI are significant problems in their own right, representing a considerable burden to individuals, their families and health services. Prediction and prevention of self-harm and suicide in young people are priorities but NSSI (5-42% in community samples[9,10]) and ST (15-25% in community samples[11,12]) are common so it is difficult to predict who will ultimately make a serious attempt[13] or die by suicide. Indeed, the usefulness of clinical risk protocols relying on the identification of a psychiatric diagnosis is questionable[14,15]. The same problems affect public health suicide prevention programmes. A seminal study revealed a high prevalence of false-negatives in prospective identification of suicide[16]. Prevention policies that embrace the whole population might overcome these difficulties but lack theoretical or empirical foundations[1].

Suicidal thoughts and behaviours are routinely considered as markers of depression (e.g., in DSM-5) but by no means all young people dying by suicide have had a mood disorder[17]. NSSI is strongly associated with the risk of suicide when occurring in combination with any internalising or externalising symptoms[18,19], or with any psychiatric diagnosis[20], particularly multiple diagnoses[21]. Thus, this risk might be better predicted by multiple symptoms rather than by the presence of a single disorder, such as depression.

Recent studies suggest that a broad range of symptoms conventionally seen as components of distinct disorders are better construed as manifestations of a single, latent dimension distributed within the general population. This dimension has been variously referred to as the p-factor[22], general psychopathology[23] or, as we prefer here, common mental

For peer review only - http://bmjopen.bmj.com/site/about/guidelines.xhtml

distress (CMD)[24,25]. Parsimonious statistical models with dimensions that encompass low-prevalence phenomena such as psychotic experiences, fit empirical data better than models with distinct disorders[22,26]. High co-morbidity of psychiatric diagnoses, shared causal factors and treatments, and trans-diagnostic psychological and neural correlates support the validity of a CMD concept[22-24,26-29]. Suicide risk is related to multiple symptoms or disorders (and thus to higher CMD scores), not the presence of one specific symptom or disorder, so it is important to understand the nature of dose-response relationships between CMD and suicide risks. This could guide a clinical response in the face of suicide risk[30] and also shape population-based suicide prevention.

In this study, we describe the presence of a CMD dimension in young people aged 14-26 years and the occurrence of ST and NSSI referred to collectively, hereafter, as a suicide risk. We draw on a psychometric study[25] that demonstrated high theoretical validity and high measurement qualities of the CMD factor comprising measures of common mental illness (depression, anxiety, psychotic experiences, obsessions and compulsions) as well as traits and characteristics commonly considered to contribute to the general level of mental health (antisocial trait, well-being, self-esteem). Our approach had three steps whereby we:

1. Tested associations between CMD and suicide risk, and contrasted CMD with specific psychopathological domains, exploring the utility of this summary measure;
2. Defined the prevalence and relative risk of NSSI and ST across the distribution of CMD;
3. Established whether the CMD_{T2} dimension measured at time 2 mediate the relationship between ST_{T1} and $NSSI_{T1}$ at time 1 and $NSSI_{T3}$ and ST_{T3} at time 3.

We used data from two population-based cohorts with complementary designs and very similar measures. In step two we used cross-sectional data from Cohort 1, time 1 (used as a

For peer review only - http://bmjopen.bmj.com/site/about/guidelines.xhtml

discovery sample) and Cohort 2 (used as a stepwise replication sample); in the third step we used three longitudinal waves of Cohort 1 (see details in Method).

Method

Study Design and Participants

Cohort 1

Participants in the NSPN 2400 Cohort[31] were recruited largely via postal invitations sent through general practitioners and schools in Cambridgeshire and Greater London, UK. Data collection was carried out in two research centres: University College London and the University of Cambridge between November 2012 and December 2016. Purposive sampling obtained at least 200 males and 200 females from the community in 5 age groups: 14-15, 16-17, 18-19, 20-21, 22-24 years. Three data collections took place a year apart (T1-T3). At T1, 2403 individuals returned questionnaires (average age 18.9 years, SD=3.0; 54% females); at T2, 1815 returned questionnaires (76% response, average age 20.0 years, SD=3.1; 56% female), and 1245 at T3 (52% of baseline; average age 21.0 years, SD=3.1; 59% female).

Cohort 2

The ROOTS study[32] was used for replication of findings from Cohort 1. Two-stage sampling involved random selection of 27 schools in Cambridgeshire, UK. Eighteen schools agreed to participate; invitations were sent to 14-year-olds randomly selected from class registers and to their parents; 1238 students participated in the initial data collection (55% female) (and further 4 data collection waves took place). Note that in the current analysis we used only the data from the third data sweep collected between February 2008 and December 2009, when participants were of average age 17.5 years, SD=0.3 (N=1074, 56% female; 87% of baseline sample), the closest age to T1 of Cohort 1.

For peer review only - http://bmjopen.bmj.com/site/about/guidelines.xhtml

Both cohorts comprised predominantly white European (77% in Cohort 1 and 87% in Cohort 2) young people, consistent with the self-ascribed demographics of the two study populations. Written consent from participants age 14 or 15 years was supplemented by written consent from their parent or legal guardian; older participants gave their own written consent. Ethical approval was obtained for Cohort 1 from the National Health Service Research Ethics Service (# 97546) and for Cohort 2 from the Cambridgeshire 2 REC (# 03/302).

For peer review only

Measures

Sociodemographic information was collected using routine methods[31,33]. The Index of Multiple Deprivation (IMD), a summary measure of the socioeconomic status of participants' residential neighbourhood, is calculated from census information[34]. Questionnaires of mental illness and wellness are set out in Table 1 and items are listed in the Supplementary table 1. Scores in questionnaires were computed according to published manuals or validation studies (cited in Table 1), standardized to unify their measurement scales.

Table 1

Statistical analysis

Confirmatory bifactor analysis with a weighted least square mean and variance adjusted (WLMSV) estimator in Mplus 7.4 was used to compute factor scores for CMD in the three data sweeps of Cohort 1 and Cohort 2 based on the model validated elsewhere[25] (see CMD measures in Table 1 beneath; the list of used items and details of bifactor modelling can be found in the Supplementary table 1). CMD factor scores were then used in all subsequent computations. Next, we addressed attrition in Cohort 1 by means of multiple imputations (see details in the Supplement).

To prove that NSSI and ST were predicted by multiple psychopathological domains and also by CMD (which represents a summary of those domains), we used Stata 12 to

compute for Cohort 1_{T1} and Cohort 2 data sensitivity / specificity indicator – the area under the curve (AUC – reported in the Supplementary table 2) for NSSI and ST as criteria. We computed a series of logistic regressions, estimating odds ratios (OR) with confidence intervals for each predictor (treated as categorical with the cut-off point above 1SD and then continuous), while we controlled for effects of age and sex (Figure 1).

For step two, distributions of CMD scores in both cohorts were plotted against lines representing percentages of subjects reporting NSSI and ST within bands of CMD expressed as standard deviations (upper panel of Figure 2) and against bar histograms representing NSSI and ST frequencies in both cohorts (lower panel of Figure 2). In addition, NSSI and ST information curves were computed to determine in what range of the CMD dimension these items are located (see Supplementary figure 1).

Using Cohort $1_{T1\text{-}T3}$ data for step three, we examined the longitudinal relationship between CMD, NSSI and ST (in particular the predictive role of CMD in persistence of NSSI and ST): we computed direct and mediation (via CMD_{T2}) effects of ST_{T1} and $NSSI_{T1}$ on $NSSI_{T3}$ and ST_{T3} in a pathway mediation model with confidence intervals in Mplus 7.4 (computing bias-corrected bootstrapping was not possible due to the use of multiply imputed datasets). We computed this model for the total sample (Figure 3) and then for both sexes separately (Supplementary figure 2) using the Multiple Group Method, so as to test a moderated-mediation model (with CMD_{T2} as a mediator, and sex as a moderator). Age was a control variable. In both pathway analyses CMD_{T2} factor scores (computed on imputed data, as described above) were modelled as observed variables.

Results

Step one: Associations of NSSI and ST with demographic and psychopathological variables

For peer review only - http://bmjopen.bmj.com/site/about/guidelines.xhtml

In both cohorts NSSI and ST were unrelated to demographic variables, including sex and age (See Supplementary tables 3 and 4); CMD was negatively related to male gender (Supplementary table 5). When examined descriptively over the pooled age groups, the prevalence of NSSI and ST mirrored the CMD levels (see Supplementary figure 3). CMD and all "conventional" mental health disorders predicted NSSI and ST (i.e., had statistically significant ORs in logistic regression models - see Figure 1 and Supplementary table 2).

Figure 1

Prevalence of NSSI and ST in the two cohorts

In Cohort 1 (N=2403) there was no statistically significant change in the prevalence of NSSI (within the last month) over the three time points: in the imputed data 9.3% (n=223) reported $NSSI_{T1}$, 8.3% (n=199) $NSSI_{T2}$ and 8.2% (n=197) $NSSI_{T3}$. Similarly, there was no statistically significant change in prevalence of ST (within the last two weeks) over the three time points: 10.1% (n=243) ST_{T1}, 11.4% (n=274) ST_{T2} and 11.7% (n=281) ST_{T3} (see Supplementary tables 6 and 7).

In Cohort 2 (N=1074), 11.7% (n=126) reported lifetime NSSI and 5.4% (n=58) reported ST within the two last weeks. Accuracy and precision of these prevalence estimates were affected by attrition (see *Discussion: limitations*). Attrition in Cohort 1 at T2 and T3 was only marginally related to demographic and exposure variables at T1 (Spearman's rho 0.05-0.12), but unrelated to the outcome – NSSI and ST (see Supplementary table 8).

Step two: Associations of NSSI and ST with CMD

Next, we focused on absolute risk[i] and the numbers of NSSI and ST events generated by these risk functions. The dose-response curves in the upper panel of Figure 3 show that relative risks[ii] of NSSI and ST increased markedly with increasing severity of CMD, the highest risks being in those with very high scores beyond two standard deviations above the

For peer review only - http://bmjopen.bmj.com/site/about/guidelines.xhtml

mean. On the other hand, most participants from both cohorts who reported NSSI or ST had mild (one SD above the mean) to moderate (two SD above the mean) CMD scores (lower panel of Figure 3). CMD was normally distributed (see Supplementary figure 4) so these scores were much more common; only a minority of the total reports came from the few participants with very high CMD (>2 standard deviations above mean CMD). Thus, the majority of subjects experiencing ST or NSSI (Cohort 1: 78% and 76%; Cohort 2: 66% and 71%, respectively) had CMD scores within two standard deviations above the population mean. Very high CMD scores indicated the highest suicide risk but were rare, so generated the minority of events.

Figure 2

Step three: Mediating effect of CMD on suicide risks in Cohort 1 over time

Cohort 1 CMD_{T2} contributed to the persistence of NSSI and ST over time (i.e. $NSSI_{T1}$ predicted $NSSI_{T3}$ directly, and via mediation through CMD_{T2}; it also completely mediated the longitudinal effect of $NSSI_{T1}$ on ST_{T3}). Moreover, CMD_{T2} contributed to the persistence of ST over time (i.e. ST_{T1} predicted ST_{T3} directly, as well as via mediating variable – CMD_{T2}). Overall, CMD_{T2} was a stronger predictor of $NSSI_{T3}$ and ST_{T3} than the antecedent variables measured at T1 (see Figure 3). The mediation effects of CMD_{T2} were similar for boys and girls (i.e., the effects were not moderated by sex – Supplementary figure 2 and Supplementary table 9). Age_{T1} was not a significant predictor of any variable in the model; the results when age was controlled for were very similar to those without controlling for age.

Figure 3

For peer review only - http://bmjopen.bmj.com/site/about/guidelines.xhtml

Discussion

In the present study, all the domains of psychopathology and mental wellness available (depression, anxiety, self-esteem, well-being, psychotic-like experiences, antisocial trait, schizotypal trait, conduct problems, obsessions and compulsions) predicted risk of non-suicidal self-injury (NSSI) and suicidal thoughts (ST). Thus, the common mental distress factor with a normal population distribution appeared a parsimonious and efficient summary of these domains and was, itself, a key predictor of suicide risk in both cohorts. NSSI and ST were not confined to participants scoring in the very high, quasi-clinical range for CMD. Around half of all participants expressing NSSI or ST came from those scoring up to one standard deviation above mean CMD in a dose-response manner. The majority expressing these phenomena (two thirds to three quarters) scored within 2SD above the mean (Figure 2). Regarding medium-term determinants of persistent NSSI and ST we showed (Figure 3) that CMD_{T2} mediated the persistence of NSSI and ST over two years, independent of gender and age. This mediation operates in two stages: first, ST and NSSI persist because these behaviours are markers for worsening CMD in the general population. This extends findings in adolescents with depressive disorder, where suicidal thoughts are a predictor of poor outcome[35]. Second, this greater CMD, itself, predicts the risk for further suicidal thoughts and behaviours.

Strengths

Both cohorts were designed on epidemiological principles to capture behavioural and psychological variation in the population during the post-pubertal epoch during which risk for psychopathology accelerates. Replication of the findings in these independent cohorts strengthens confidence in the findings, as does internal consistency between cross-sectional associations found in both cohorts, and longitudinal associations found in Cohort 1.

For peer review only - http://bmjopen.bmj.com/site/about/guidelines.xhtml

Limitations

Sample attrition was the main bias in both cohorts. Each retained more young women than men; we found marginally higher attrition among lower socio-economic class, participants of non-white ethnicity and those with higher CMD (Supplementary table 8). Cohort 1 is robustly representative of the England and Wales population[31], whereas Cohort 2 under-represents participants with lowest socioeconomic status[32]. However, we have no reason to suppose that attrition biased our results, as it was unrelated to NSSI and ST (Supplementary table 8). If there was a bias, it probably limits power rather than skewing an effect and is mitigated by replication between the cohorts. We used multiple imputation to minimise this bias.

There was only modest reliability of our obsessionality measure and a skewed measure of conduct problems in Cohort 1. A completely comprehensive range of psychopathological (and behavioural) items was unavailable; we did not have measures of unstable or abnormally elevated mood, addictions, eating disorders or hyperactivity. Thus, our measurement of CMD focused primarily on internalising rather than externalising symptoms. Future studies could include a broader range of measures and extend the investigation into clinical populations to improve measurement precision at the highest levels of CMD. Although ethnicity and socioeconomic status (indicated by IMD) were unrelated to ST and NSSI (Supplementary tables 3 and 4), and thus were not included in our analyses, we did not control for the effect of other possible confounders such as adverse life experiences, early trauma, family structure or more detailed information about family socio-economic situation (unemployment, poverty etc.). Finally, we could not account for the effects of clustered design in the modelling, due to unavailability of the information about clustering of participants in both cohorts.

For peer review only - http://bmjopen.bmj.com/site/about/guidelines.xhtml

Implications & Conclusions

Our findings provide yet more evidence that a latent mental distress factor, conceptually akin to the p-factor, is a useful summary measure of psychopathology in the general population[24], diagnostic[22], and clinical[23] samples. We speculate that psychopathological items accumulate in a probabilistic manner rather than in diagnostic clusters, with common phenomena concerning depression and anxiety much more likely to occur before rarer phenomena such as NSSI, ST or psychotic experiences. Less frequent phenomena begin to co-occur as the severity of psychological disorder (or CMD) increases, in terms of more mental and behavioural phenomena or symptoms. This begins to yield clusters linked by common items that current diagnostic systems tend to ignore. This is consistent with the co-occurrence of suicidal risk and psychotic experiences seen in other[36-38] studies of young people, and with the present IRT analysis showing that NSSI and ST are measuring the higher end of CMD (Supplementary figure 1). The approach we have followed illustrates the value of moving away from categorical classification and embracing an empirically-rooted, dimensional, hierarchical taxonomy in psychopathology research[39]. Such hierarchical approaches to phenomenological classification had been put forward before[40] or shortly after[41] the publication of DSM-3 and its successor classifications. Hierarchical models merit renewed interest[42], as they may resolve problems of comorbidity[26] as well as overlapping causes and biological mechanisms for suicide risk and other phenomena[43,44]. In contrast to the CMD idea, there is also increasing interest in approaches focusing on individual symptoms and experiences, particularly to guide individual clinical interventions, rather than grouping the symptoms into diagnostic categories or higher-order constructs[45]. Future studies may investigate and compare the utility of such novel approaches (CMD and item-focused approach) for clinical practice and public health policies.

For peer review only - http://bmjopen.bmj.com/site/about/guidelines.xhtml

Our findings also have major implications for intervention and prevention of suicidal thoughts and behaviours. Clinically, the results suggest that NSSI and ST should never be dismissed or downplayed when they occur in young people without clear evidence of psychiatric disorder, a logical fallacy because NSSI and ST are *themselves* indicators of higher distress on a CMD factor. NSSI and ST will usually, but not always occur with other, more common psychopathology and their co-occurrence is a strong risk factor for suicide attempts[6]. Thus, NSSI and ST merit a swift professional response regardless of whether or not they occur with other symptoms that take individuals beyond conventional clinical thresholds and trigger traditional clinical risk protocols. Our findings help explain why research focused on high-risk subjects has yet to translate into useful clinical prediction tools[14,15,45].

From a public health and prevention perspective, the fact that rates of NSSI and ST begin to accelerate at levels of CMD well within a normal or non-clinical range argues strongly for universal interventions overtly aimed at lowering the population mean CMD and shifting the curve to the left. This should be alongside targeted approaches and effective clinical services[46]. Strategies concentrated on clinical populations, those with evidence of a psychiatric disorder or other individual markers will miss the majority of individuals experiencing ST or engaging in NSSI because there are so few compared with those at lower risk: the *prevention paradox*[30].

Defining putative universal interventions to shift the population distribution of CMD will require careful research that can draw from other areas of medicine such as cardiovascular disease and stroke[30]. Elements have been widely scoped in the USA[15] and elsewhere, but not for constructs of population health and wellbeing such as CMD. Interventions may involve decreasing common triggers[15] or improving young people's abilities to cope with stressors[47, 48, 49].

For peer review only - http://bmjopen.bmj.com/site/about/guidelines.xhtml

Conflict of Interest Disclosures

E.P., S.N., I.M.G., and J.S. have no competing interests. E.B., P.F., and P.B.J. are in receipt of National Institute for Health Research (NIHR) Senior Investigator Awards (NF-SI-0514-10157, and NF-SI-0514-10117. P.F. was in part supported by the NIHR Collaboration for Leadership in Applied Health Research and Care (CLAHRC) North Thames at Barts Health NHS Trust. P.W. has recent/current grant support from NIHR, Cambridgeshire County Council and CLAHRC East of England. P.W. discloses consulting for Lundbeck and Takeda; P.B.J. discloses consulting for Janssen and Ricordati. E.B. is employed half-time by the University of Cambridge and half-time by GlaxoSmithKline in which he holds stock.

Data sharing/ Data availability

E.P. had full access to all the data in the study and takes responsibility for the integrity of the data and the accuracy of the data analysis. The data are deposited in the University of Cambridge Data Repository, with the placeholder DOI https://doi.org/10.17863/CAM.25331 available to researchers via openNSPN@medschl.cam.ac.uk.

Funding/Support and Acknowledgments

The ROOTS study was supported by a Wellcome Trust Grant (Grant number 074296) to I.M.G. and P.B.J., the NIHR Collaborations for Leadership in Applied Research and Care (CLAHRC) East of England, and the NIHR Cambridge Biomedical Research Centre. The NSPN study was supported by the Wellcome Trust Strategic Award (095844/Z/11/Z) to I.M.G., E.B., P.B.J., R.D., P.F. The work has been carried out in the Department of Psychiatry, University of Cambridge. We wish to thank the NSPN and ROOTS participants and Dr Golam Khandaker for his comments and NSPN Consortium (see the list of members in the Supplement).

Role of the Funder/Sponsor

The funding sources had no role in the design and conduct of the study; collection, management, analysis, and interpretation of the data; preparation, review, or approval of the manuscript; and decision to submit the manuscript for publication.

For peer review only - http://bmjopen.bmj.com/site/about/guidelines.xhtml

NSPN Consortium Information –see the Supplement

Patient and public involvement: Patients and the public were not involved in the design or planning of the study.

For peer review only

For peer review only - http://bmjopen.bmj.com/site/about/guidelines.xhtml

Table 1. Measures used in both cohorts

Variables	*Measures*	Cohorts	
**Outcome variables:**		$NSPN_{T1-T3}$ (1)	$ROOTS_{age\ 17}$ (2)
Suicidal thoughts (ST)	One item from the MFQ[50]: I thought about killing myself. Responses were recoded into a binary format: no ST (original response option *Never)* and ST (original response options *Sometimes* or *Mostly* or *Always*).	×	×
Non-suicidal self-injury (NSSI)	One question from the Drug, Alcohol and Self-Injury (DASI)[25] questionnaire asking about engaging in self-injury without suicidal intent during the last month. Responses were recoded into a binary format indicating the occurrence of NSSI or lack of thereof.	×	
	One question asking about the occurrence of lifetime NSSI (DASI)[25]		×
**Predictors:**			
Conduct problems	11-item Antisocial Behaviour Questionnaire[25]	×	×
Anxiety	28-item Revised Children's Manifest Anxiety Scale[51]	×	×
Depression	29 items from the 33-item MFQ[50] (all items except for 4 items measuring suicidality)		
Obsessions and compulsions	11-item Revised Leyton Obsessional Inventory[52]	×	×
Psychotic-like experiences	11 items selected from the 74-item Schizotypal Personality Questionnaire (SPQ)[53]	×	
	11 items from the 20-item semi-structured interview from the Diagnostic Interview Schedule for Children-IV[54]		×
Self-esteem	10-item Rosenberg Self-Esteem Questionnaire (*)[55]	×	×
Well-being	14-item Warwick-Edinburgh Mental Well-Being Scale(*)[56]	×	×
Impulsivity	15 items from the 30-item Barratt Impulsiveness Scale[57] selected based on exploratory factor analysis - loadings above .25	×	
Antisocial traits	Total score from the 17-item Antisocial Process Screening Device (APSD)[58]	×	
Schizotypal traits	Total score from the 74-item Schizotypal Personality Questionnaire (SPQ) [53]	×	×

*scales were reversely scored, thus higher scores indicated lower self-esteem and well-being; for all other measures higher score indicates more psychopathology

For peer review only

Figures' legends:

Figure 1: Odds ratio in logistic regressions for suicidal thoughts (ST) and non-suicidal self-harm (NSSI) as outcomes predicted by psychopathological predictors (listed on the left) here treated as continuous variables; regressions were computed separately for each predictor and effects of age and sex were controlled in each regression for in both cohorts (see Supplementary Table 2).

Figure 2: Upper panel shows the dose-response effect of Common Mental Distress on non-suicidal self-harm (NSSI) and suicidal thought (ST) in Cohort 1 and Cohort 2. The lower panel shows the proportion of total reports in non-suicidal self-injury (NSSI) and suicidal thought (ST) broken down by standard deviations of Common Mental Distress; these add up to 100% from left to right. The normal population distribution of CMD, which was strikingly similar, but not identical, in Cohort 1 and 2, is shown by the purple line (see density plots in Supplement, Figure 1).

Figure 3: Mediation effect of Common Mental Distress at time 2 in Cohort 2: Standardised pathway coefficients with confidence intervals in square brackets.

For peer review only

For peer review only - http://bmjopen.bmj.com/site/about/guidelines.xhtml

References:

1. Hawton K, Saunders EA, O'Connor R. Self-harm and suicide in adolescents. *Lancet.* 2012;379:2373-2382.
2. Kidger J, Heron J, Lewis G, Evans J, Gunnell D. Adolescent self-harm and suicidal thoughts in the ALSPAC cohort: A self-report survey in England. *BMC Psychiatry.* 2012;12:1-12.
3. Nock MK. Future directions for the study of suicide and self-injury. J Clin Child Adolesc Psychol. 2012;41:255-259.
4. Scott LN, Pilkonis PA, Hipwell AE, Keenan K, Stepp SD. Non-suicidal self-injury and suicidal ideation as predictors of suicide attempts in adolescent girls: A multi-wave prospective study. *Compr Psychiatry.* 2015;58:1-10.
5. Ribeiro JD, Franklin JC, Fox KR, et al. Self-injurious thoughts and behaviors as risk factors for future suicide ideation, attempts, and death: A meta-analysis of longitudinal studies. *Psychol Med.* 2016;46:225-236.
6. Victor SE, Klonsky ED. Correlates of suicide attempts among self-injurers: A meta-analysis. *Clin Psychol Rev.* 2014;34(4):282-297.
7. Cooper J, Kapur N, Webb R, et al. Suicide after deliberate self-harm: a 4-year cohort study. *Am J Psychiatry.* 2005;162:297-303.
8. Patton GC, Coffey C, Sawyer SM, et al. Global patterns of mortality in young people: a systematic analysis of population health data. *Lancet*. 2009;374:881-892.
9. Muehlenkamp JJ, Claes L, Havertape L, Plener PL. International prevalence of adolescent non-suicidal self-injury and deliberate self-harm. *Child Adolesc Psychiatry Ment Health.* 2012;6(10).
10. Brunner R, Parzer P, Haffner J, et al. Prevalence and psychological correlates of occasional and repetitive deliberate self-harm in adolescents. *Arch Pediatr Adolesc Med.* 2007;161(7):641-649.
11. Bridge JA, Goldstein TR, Brent DA. Adolescent suicide and suicidal behavior. *J Child Psychol Psychiatry*. 2006;47:372-394.
12. Evans E, Hawton K, Rodham K, Deeks J. The prevalence of suicidal phenomena in adolescents: A systematic review of population-based study. *Suicide Life Threat Behav.* 2005;35(3):239-50.
13. Nielssen O, Wallace D, Large M. Pokorny's complaint: the insoluble problem of the overwhelming number of false positives generated by suicide risk assessment. *BJPsych Bulletin.* 2017;41:18-20.
14. Quinlivan L, Cooper J, Davies L, et al. Which are the most useful scales for predicting repeat self-harm? A systematic review evaluating risk scales using measures of diagnostic accuracy. *BMJ Open.* 2016;6(2): https://bmjopen.bmj.com/content/6/2/e009297.
15. Quinlivan L, Jayne Cooper J, Meehan D, et al. Predictive accuracy of risk scales following self-harm: multicentre, prospective cohort study. *Br J Psychiatry.* 2017;210:429-436.
16. Pokorny AD. Prediction of suicide in psychiatric patients. Report of a prospective study. *Arch Gen Psychiatry.* 1983;40:249-257.
17. Shaffer D, Gould MS, Fisher P, et al. Psychiatric diagnosis in child and adolescent suicide. *Arch Gen Psychiatry.* 1996;53:339-348.
18. Berona J, Horwitz AG, Czyz EK, King CA. Psychopathology profiles of acutely suicidal adolescents: Associations with post-discharge suicide attempts and rehospitalisation. *J Affect Disord*. 2017;209:97-104.
19. Nock MK, Joiner TE, Gordon KH, Lloyd-Richardson E, Prinstein MJ. Non-suicidal self-injury among adolescents: diagnostic correlates and relation to suicide attempts. *Psychiatry Res*. 2006;144(1):65-72.
20. Beckman K, Mittendorfer-Rutz E, Lichtenstein P, et al. Mental illness and suicide after self-harm among young adults: long-term follow-up of self-harm patients, admitted to hospital care, in a national cohort. *Psychol Med.* 2016;46:3397-3405.

For peer review only - http://bmjopen.bmj.com/site/about/guidelines.xhtml

21. Windfuhr K, While D, Kapur N, et al. Suicide risk linked with clinical consultation frequency, psychiatric diagnoses and psychotropic medication prescribing in a national study of primary-care patients. *Psychol Med.* 2016;46:3407-3417.
22. Caspi A, Houts RM, Belsky DW, et al. The p factor: One General Psychopathology Factor in the structure of psychiatric disorders? *Clin Psychol Sci.* 2014;2:119-137.
23. Patalay P, Fonagy P, Deighton J, et al. A general psychopathology factor in early adolescence. *The Br J Psychiatry.* 2015;207:15-22.
24. Stochl J, Khandaker GM, Lewis G, et al. Mood, anxiety and psychotic phenomena measure a common psychopathological factor. *Psychol Med.* 2015;45:1483-1493.
25. St Clair CM, Neufeld S, Jones BP, et al. Characterising the latent structure and organisation of self-reported thoughts, feelings and behaviours in adolescents and young adults. *PLOS* One. 2017; 12(4), 1-27. doi: https://doi.org/10.1371/journal.pone.0175381
26. Krueger RF. The structure of common mental disorders. *Arch Gen Psychiatry.* 1999;56:921-926.
27. Barch DM. The Neural Correlates of Transdiagnostic Dimensions of Psychopathology. *Am J Psychiatry*. 2017;174:613-615.
28. McTeague LM, Huemer J, Carreon DM, et al. Identification of Common Neural Circuit Disruptions in Cognitive Control Across Psychiatric Disorders. *Am J Psychiatry*. 2017;174:676-685.
29. Sharma A, Wolf DH, Ciric R, et al. Common Dimensional Reward Deficits Across Mood and Psychotic Disorders: A Connectome-Wide Association Study. *Am J Psychiatry.* 2017;174:657-666.
30. Rose G, Khaw M, Marmot G, Kay-Tee K, Marmot M. Chapter 3: The relation of risk to exposure. In: *Rose's strategy of preventive medicine (New ed.)* Oxford, Oxford University Press: 2008.
31. Kiddle B, Inkster B, Prabhu G, et al. The NSPN 2400 Cohort: a developmental sample supporting the Wellcome Trust NeuroScience in Psychiatry Network. *Int J Epidemiol.* 2018;47(1):18-19g.
32. Goodyer IM, Croudace T, Dunn V, Herbert J, Jones BP. Cohort Profile: Risk patterns and processes for psychopathology emerging during adolescence: the ROOTS project. *Int J Epidemiol.* 2010;39:361-369.
33. Office for National Statistics. Ethnicity and National Identity in England and Wales. 2012. Retrieved on 6th Feb 2018 from: https://www.ons.gov.uk/peoplepopulationandcommunity/culturalidentity/ethnicity/articles/ethnicityandnationalidentityinenglandandwales/2012-12-11#ethnicity-in-england-and-wales.
34. Noble M, McLennan, D, Wilkinson K, Whitworth A, & Barne H. The English Indices of Deprivation 2007. London: Department for Communities and Local Government. (2008).
35. King RA. Adolescent suicidal thoughts/behaviors are a marker of long-term vulnerability to poor adult outcomes. *J Am Acad Child Adolesc Psychiatry.* 2017;56:920-921.
36. Kelleher I, Keeley H, Corcoran P, et al. Clinicopathological significance of psychotic experiences in non-psychotic young people: evidence from four population-based studies. *Br J Psychiatry*. 2012;201:26-32.
37. Honings S, Drukker M, Groen R, van Os J. Psychotic experiences and risk of self-injurious behaviour in the general population: a systematic review and meta-analysis. *Psychol Med.* 2016;46:237-251.
38. Kelleher I, Corcoran P, Keeley H, et al. Symptoms and population risk for suicide attempt: A prospective cohort study. *JAMA Psychiatry.* 2013;70:940-948.
39. Lahey BB, Krueger RF, Rathouz PJ, Waldman ID, Zald DH. A hierarchical causal taxonomy of psychopathology across the life span. *Psychol Bull.* 2017;143:142-186.
40. Foulds GA, Bedford A. Hierarchy of classes of personal illness. *Psychol Med.* 1975;5:181-192.
41. Sturt E. Hierarchical patterns in the distribution of psychiatric symptoms. *Psychol Med.* 1981;11:783-792.

For peer review only - http://bmjopen.bmj.com/site/about/guidelines.xhtml

42. Kotov R, Krueger RF, Watson D. A paradigm shift in psychiatric classification: the Hierarchical Taxonomy Of Psychopathology (HiTOP). *World Psychiatry.* 2018;17:24-25.
43. Zald DH, Lahey BB. Implications of the Hierarchical Structure of Psychopathology for Psychiatric Neuroimaging. *Biol Psychiatry Cogn Neurosci Neuroimaging.* 2017;2:310-317.
44. Carver CS, Johnson SL, Timpano KR. Toward a functional view of the p factor in psychopathology. *Clinical Psychological Scienc.* 2017;5(5):880-889.
45. Fried EI, & Nesse RM. Depression sum-scores don't add up: why analyzing specific depression symptoms is essential. BMC Medicine. 2015; 13:72; https://bmcmedicine.biomedcentral.com/track/pdf/10.1186/s12916-015-0325-4
46. Franklin JC, Ribeiro JD, Fox KR, et al. Risk factors for suicidal thoughts and behaviors: A meta-analysis of 50 years of research. *Psychol Bull.* 2017;143(2):187-232.
47. Lewis G, Hawton K, Jones PB. Strategies for preventing suicide. *British Joournal of Psychiatry.* 1997;171:351-354.
48. Galante J, Dufour G, Vainre M, et al. A mindfulness-based intervention to increase resilience to stress in university students (the Mindful Student Study): a pragmatic randomised controlled trial. Lancet Public Health. 2018 Feb;3(2):e72-e81.
49. Wasserman D, Hoven CW, Wasserman C, et al. School-based suicide prevention programmes: the SEYLE cluster-randomised, controlled trial. *Lancet*. 2015;385:1536-1544.
50. Hammerton G, Zammit S, Potter R, Thapar A, Collishaw S. Validation of a composite of suicide items from the Mood and Feelings Questionnaire (MFQ) in offspring of recurrently depressed parents. *Psychiatry Res.* 2014;216:82-88.
51. Reynolds CR. Concurrent validity of what I think and feel: The revised children's manifest anxiety scale. J Consult Clin Psychol. 1980;48:774-775.
52. Bamber, D., Tamplin, A., Park, R.J., Kyte, Z.A. & Goodyer, I.M. Development of a short Leyton Obsessional Inventory For Children and Adolescents. *Journal of the American Academy of Child and Adolescent Psychiatry* **41**, 1246-1252 (2002).
53. Raine A. The SPQ: A scale for the assessment of schizotypal personality based on DSM-III-R criteria. *Schizophr Bull.* 1991;17:555-564.
54. Shaffer D, Fisher P, Lucas CP, Dulcan MK, Schwab-Stone ME. NIMH Diagnostic Interview Schedule for Children Version IV (NIMH DISC-IV): description, differences from previous versions, and reliability of some common diagnoses. *J Am Acad Child Adolesc Psychiatry.* 2000;39:28-38.
55. Rosenberg M. *Society and the adolescent self-image*. Princeton, NJ: Princeton University Press; 1965.
56. Tennant R, Hiller L, Fishwick R, et al. The Warwick-Edinburgh mental well-being scale (WEMWBS): development and UK validation. Health Qual Life Outcomes. 2007;5:63.
57. Patton JH, Stanford MS, Barratt ES. Factor structure of the Barratt Impulsiveness Scale. *J Soc Clin Psychol.* 1995;51:768-774.
58. Poythress NG, Poythress NG, Douglas KS, Falkenbach D, et al. Internal consistency reliability of the self-report antisocial process screening device. *Asmnt.* 2006;13:107-113.

For peer review only

Authors contributions:

E.P. – conceptualised the study, computed statistical analyses and drafted the manuscript

P.B.J. – provided senior supervision, conceptualised the study, advised on statistical analyses, read and critically apprised the manuscript, re-drafted and edited the manuscript

J.S. – provided statistical advice, replicated multiple imputations, provided data from multiple imputations, read and critically apprised the manuscript

S.N. – advised on handling missing data, replicated multiple imputations, read and critically apprised the manuscript

P.W. – read and critically apprised the manuscript, provided key referred articles

R.D. – read and critically apprised the manuscript, provided key referred articles

I.M.G. – read and critically apprised the manuscript, provided key referred articles

E.B. – read and critically apprised the manuscript, provided key referred articles

P.F. – read and critically apprised the manuscript, provided key referred articles

R.D. – read and critically apprised the manuscript

M.C.S.C. – contributed to data collection and project management, provided advise on bifactor modelling

G.P. – contributed to data collection and project management

---

[i] Absolute risk is the probability or chance of an event. It is usually used for the number of events (e.g., a suicide) that occurred in a group, divided by the number of people in that group.

[ii] A relative risk compares the risk of a health event (e.g., a suicide) among one group with the risk among another group.

For peer review only - http://bmjopen.bmj.com/site/about/guidelines.xhtml

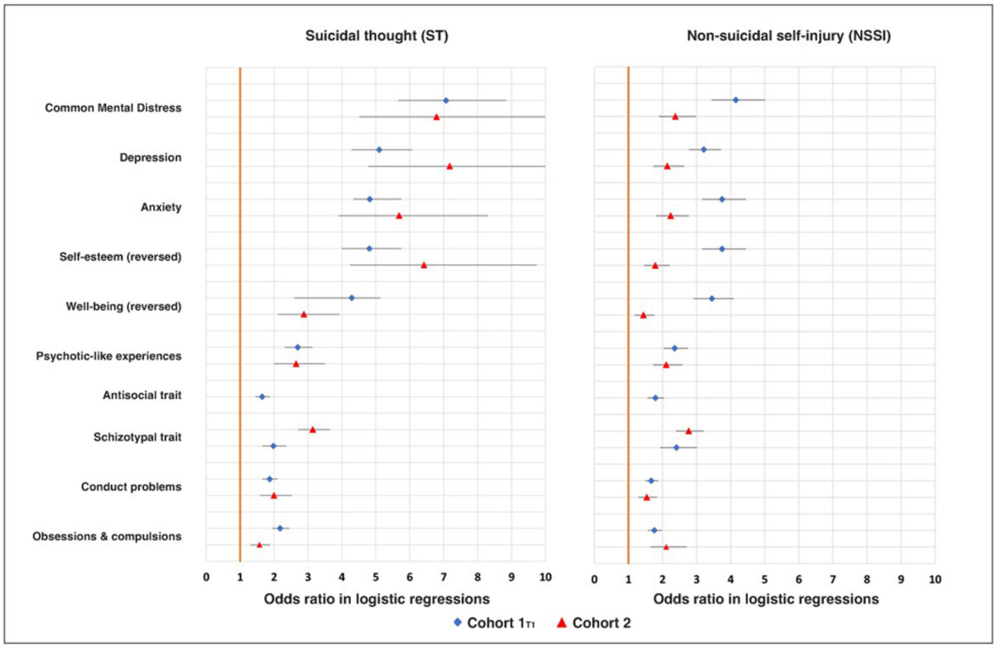

Figure 1: Odds ratio in logistic regressions for suicidal thoughts (ST) and non-suicidal self-harm (NSSI) as outcomes predicted by psychopathological predictors (listed on the left) here treated as continuous variables; regressions were computed separately for each predictor and effects of age and sex were controlled in each regression for in both cohorts (see Supplementary Table 2).

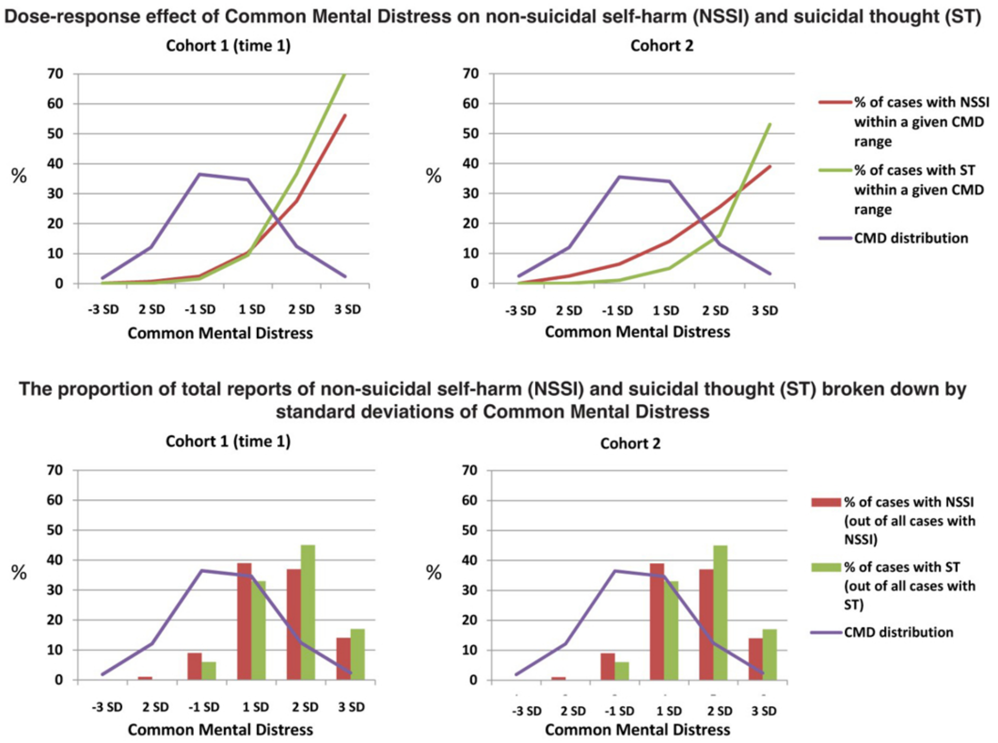

Figure 2: Upper panel shows the dose-response effect of Common Mental Distress on non-suicidal self-harm (NSSI) and suicidal thought (ST) in Cohort 1 and Cohort 2. The lower panel shows the proportion of total reports in non-suicidal self-injury (NSSI) and suicidal thought (ST) broken down by standard deviations of Common Mental Distress; these add up to 100% from left to right. The normal population distribution of CMD, which was strikingly similar, but not identical, in Cohort 1 and 2, is shown by the purple line (see density plots in Supplement, Figure 1).

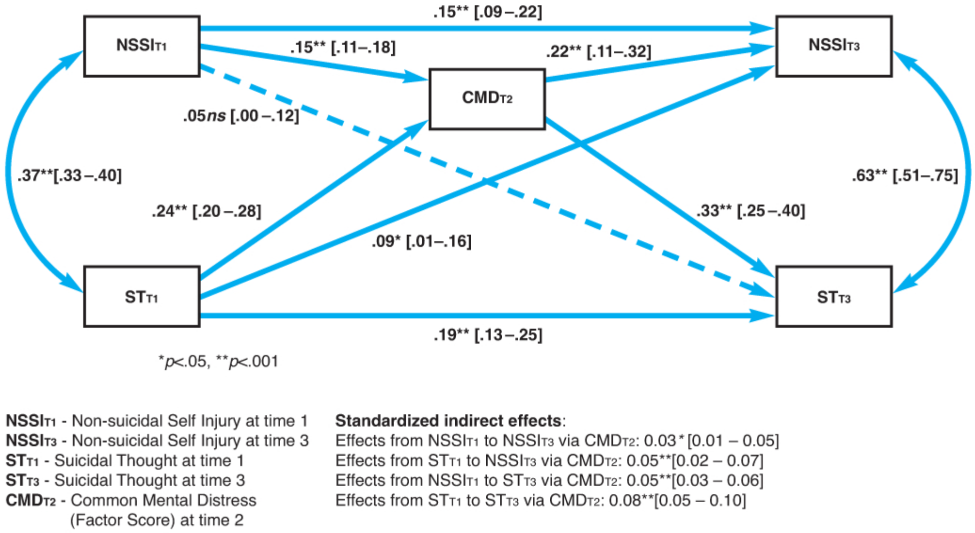

Figure 3: Mediation effect of Common Mental Distress at time 2 in Cohort 2: Standardised pathway coefficients with confidence intervals in square brackets.

Bifactor modelling:

Bifactor psychometric modelling is designed to extract variance common for all items in the model to generate one "general" factor. In addition to this general factor, specific factor/s may emerge, which are uncorrelated with each other or with the general factor. Specific factor/s contain the remaining variance after the extraction of the general factor[1]. St Clair et al. (2017) found in her psychometric study a bifactor model with one general factor and 5 specific factors, which fitted the data better than the correlated-factors model or second-order model. In our study, we first replicated St Clair et al. (2017) psychometric model in Cohort 1 (T1, T2, T3) and Cohort 2. In accordance with the original study, in our psychometric modelling the same measures of common mental illness frequently emerging during adolescence (depression, anxiety, psychotic experiences, obsessions and compulsions, conduct problems) as well as traits and characteristics commonly considered to contribute to mental wellness (well-being, self-esteem) were used as constructs contributing the general factor (see items below). Having replicated St Clair et al (2017) bifactor model, we then computed factor scores for the general factor – here termed *Common Mental Distress (CMD).*

The confirmatory bifactor analysis in Cohort 1 was computed with the multiple group method (MGM) in Mplus 8 with the three data point used as a grouping variable; the same model was fitted to the data in each group. MGM in Mplus by default holds thresholds and loadings invariant across groups[2], thus allowing the comparison if the model fits data well in all groups under study (here data from the three measurement points). The effective sample for the 3 data waves was, respectively, n=2403, n=1815, n=1245 (Total N=5463). The overall chi-square test for the model was $\chi2$=33648.24 (*df*=14983, *p*=0.000), for Time 1 it was $\chi2$=14791.20, for Time 2 it was $\chi2$=10400.56 and for Time 3 it was $\chi2$=8456.47. The overall Root Mean Square Error of Approximation (RMSEA) for the model was 0.026 (0.026-0.027), Comparative Fit Index (CFI) was 0.969, Tucker-Lewis Index (TLI) was 0.969, and weighted root mean square residual (WRMR) was 2.91. The confirmatory bifactor analysis was used in Cohort 2 as well. The following fit indexes were obtained in Cohort 2: $\chi2$=7602.17 (df=4462, *p*=0.000), RMSEA=0.026 (0.025-0.027), CFI=0.96, TLI=0.96, WRMR= 1.34. The above-cited fit indexes suggest that the bifactor model fitted the data well in both cohorts.

In both analyses – for Cohort 1 and 2 – we used WLSMV estimator and THETA parametrisation with PROBIT link, and all items were treated as ordered-categorical variables.

Much debate in the literature has focused on the issue of interpretability of specific factors, i.e., whether they should be considered as measures of meaningful concepts or should be treated as comprising the residual, uninterpretable variance[3]. The general factor in St Clair et al (2017) study demonstrated high reliability and validity, as well as low measurement error compared to validity and error of the specific factors. As follows, we focused in our study only on this general (CMD) factor; we did not attempt to interpret or use in our analyses the specific factors, even though they emerged in our bifactor modelling, due to their relatively high measurement error and ambiguity of their theoretical interpretation. The list of items contributing to CMD factor with factor loadings on this factor in Cohort 1 (T1, T2, T3) and Cohort 2 are listed below in Supplementary Table 1.

For peer review only - http://bmjopen.bmj.com/site/about/guidelines.xhtml

Multiple imputation procedure in Cohort 1:

Missingness in Cohort 1 predominantly arose from longitudinal attrition – 24% at T2 and 48% at T3; a small fraction of data was also missing due to omissions of items (between 0 to 6%). Before performing imputations, we examined if longitudinal attrition was related to demographic variables and other variables under study. Indeed, we found small, yet statistically significant correlations between attrition at T2 and T3 and demographic and exposure variables at T1 (see Supplementary Table 8), thus indicating that the assumption of "missing completely at random (MCAR) is not met. Moreover, we performed Little's MCAR test and found that it was significant ($p<.001$). Therefore, we assumed that MAR condition was met. As follows, we imputed missing data under MAR condition in Cohort 1 at T2 and T3 with the following variables in one imputation model: CMD factor scores, NSSI and ST variables. We used the following auxiliary variables: research points, sex, age, ethnicity, and Index of Multiple Deprivation (IMD) (as an indicator of a socioeconomic status[4]) as predictors of the missingness, in addition to main predictors – CMD factor scores, NSSI, and ST at T1.

Multiple imputations were computed in R program with MICE package[5]; convergence was examined by visual inspection of MCMC chains (with a maximum number of 20 iterations per chain and Gibbs sampling). Fifty-four (N=2403) datasets were generated to equal the percentage of missing data in CMD, NSSI, and ST at T3[6]. In terms of the imputation model, we used mean matching for continuous variables (CMD factor scores) and logistic regression for binary variables (NSSI and ST). The imputed 54 datasets were then used in pathway analysis (see the main manuscript and Supplementary Figure 3 for details) with MLM estimator in Mplus 7.4, which automates the process of analysing and combining parameter estimates from each imputed dataset using Rubin's rules[7].

For peer review only - http://bmjopen.bmj.com/site/about/guidelines.xhtml

Supplementary Table 1: List of all items used in the study

Outcome measures:

Suicidal Thought (ST)

I thought about killing myself (MFQ19, response options: *Always, Mostly, Sometimes, Never*)[Cohort 1 & 2]

This is one of the 4 items assessing suicidal thoughts in the 33-item Mood and Feelings Questionnaire (MFQ)[8] : MFQ16 - I thoughts that life was not worth living; MFQ17 - I thought about dying; MFQ18 – I thought my family would be better off without me; MFQ19 - I thought about killing myself. We used item 19, as it had the highest (.70) loading on this sub-subscale. Responses to this item were recoded into a binary format: no ST (original response option *Never*) and ST (original response options *Sometimes* or *Mostly* or *Always*). We did not include MFQ items 16-18 in CMD factor to avoid content overlap between the outcome measure (ST) and the predictor – the CMD factor.

Non-Suicidal Self-Injury (NSSI)

NSSI in Cohort 1 was assessed with one question from the Drug, Alcohol and Self-Injury (DASI) questionnaire asking about engaging in self-injury without suicidal intent during the last month:

In the last month, have you tried to hurt yourself on purpose without trying to kill yourself? (Response options: *Yes, No)*

NSSI in Cohort 2 was assessed with one question from the DASI questionnaire asking about life-time occurrence of NSSI:

Supplementary Table 9: Items comprising the Common Mental Distress (CMD) factor

**Items and associated measures**	**Standardised Factor Loadings**			
**The Moods and Feelings Questionnaire (MFQ)[11 Cohort 1 & 2]** **(response options: *Always, Mostly, Sometimes, Never*)** *Note:* 4 items measuring suicidality were excluded to avoid content overlap between the measures of variables treated here as predictors (CMD, Depression) and the outcome variable (ST). We excluded 4 other items which caused model convergence problems: *I was less hungry than usual (MFQ3), I ate more than usual (MFQ4), It was hard for me to make up my mind (MFQ10), I slept a lot more than usual (MFQ33)*	**Cohort 1**			**Cohort 2**
	**Time 1**	**Time 2**	**Time 3**	
1. I felt miserable or unhappy. (MFQ1)	.69	.73	.71	.73
2. I didn't enjoy anything. (MFQ2)	.62	.70	.72	.67
3. I felt so tired I just sat around and did nothing. (MFQ5)	.53	.56	.57	.54

For peer review only - http://bmjopen.bmj.com/site/about/guidelines.xhtml

4. I was moving and walking more slowly than usual. (MFQ6)	.54	.59	.54	.52
5. I was very restless. (MFQ7)	.48	.54	.56	.49
6. I felt I was no good any more. (MFQ8)	.78	.82	.84	.77
7. I sometimes blamed myself for things that weren't my fault. (MFQ9)	.70	.74	.75	.73
8. I got grumpy and cross easily. (MFQ11)	.60	.65	.68	.65
9. I felt like talking a lot less than usual. (MFQ12)	.64	.66	.69	.65
10. I was talking more slowly than usual. (MFQ13)	.56	.64	.55	.59
11. I cried a lot. (MFQ14)	.64	.64	.68	.69
12. I thought there was nothing good for me in the future. (MFQ15)	.72	.77	.78	.72
13. I didn't want to see my friends. (MFQ20)	.69	.73	.70	.66
14. I found it hard to think properly or concentrate. (MFQ21)	.73	.77	.77	.72
15. I thought bad things would happen to me. (MFQ22)	.76	.77	.80	.81
16. I hated myself. (MFQ23)	.81	.82	.85	.80
17. I was a bad person. (MFQ24)	.73	.76	.78	.72
18. I thought I looked ugly. (MFQ25)	.65	.70	.70	.69
19. I worried about aches and pains. (MFQ26)	.46	.50	.50	.56
20. I felt lonely. (MFQ27)	.70	.74	.73	.74
21. I thought nobody really loved me. (MFQ28)	.75	.79	.83	.76
22. I didn't have any fun at school / college / work. (MFQ29)	.62	.67	.66	.58
23. I thought I could never be as good as other people my age. (MFQ30)	.76	.79	.78	.76
24. I did everything wrong. (MFQ31)	.83	.85	.87	.82
25. I didn't sleep as well as usual. (MFQ32)	.53	.57	.61	.60
**The Revised Children's Manifest Anxiety Scale (RCMAS)[12] Cohort 1 & 2** **(response options: *Always, Mostly, Sometimes, Never*)**				
1. I had trouble making up my mind. (RCMAS1)	.60	.68	.71	.59
2. I worried when things did not go the right way for me. (RCMAS2)	.71	.77	.79	.78
3. Others seemed to do things more easily than I could. (RCMAS3)	.76	.80	.83	.76
4. Often I had trouble getting a breath. (RCMAS4)	.56	.60	.59	.55
5. I worried a lot of the time. (RCMAS5)	.78	.80	.82	.78
6. I was afraid of a lot of things. (RCMAS6)	.78	.80	.82	.77
7. I got angry easily. (RCMAS7)	.63	.68	.74	.68

For peer review only

8. I worried about what my parents would say to me. (RCMAS8)	.62	.67	.71	.65
9. I felt that others did not like the way I did things. (RCMAS9)	.73	.79	.78	.74
10. It was hard for me to get to sleep at night. (RCMAS10)	.55	.63	.58	.57
11. I worried about what other people thought about me. (RCMAS11)	.74	.79	.80	.71
12. I felt alone even when there were people with me. (RCMAS12)	.80	.84	.86	.85
13. Often I felt sick to my stomach. (RCMAS13)	.69	.74	.74	.76
16. I was tired a lot. (RCMAS16)	.62	.67	.69	.65
17. I worried about what was going to happen. (RCMAS17)	.77	.80	.81	.79
18. Other people my age were happier than me. (RCMAS18)	.79	.83	.83	.79
19. I had bad dreams. (RCMAS19)	.54	.59	.57	.62
20. My feelings got hurt easily when I was fussed at. (RCMAS20)	.75	.76	.78	.77
21. I felt someone would tell me I did things the wrong way. (RCMAS21)	.70	.77	.77	.71
22. I wake up scared some of the time. (RCMAS22)	.64	.74	.72	.67
23. I worried when I went to bed at night. (RCMAS23)	.67	.74	.73	.75
24. It was hard for me to keep my mind on my work. (RCMAS24)	.48	.58	.56	.55
25. I wiggled in my seat a lot. (RCMAS25)	.77	.79	.80	.76
27. A lot of people were against me. (RCMAS27)	.75	.80	.83	.80
28. I often worried about something bad happening to me. (RCMAS28)	.74	.79	.79	.80
**The Revised Leyton Obsessional Inventory (R-LOI)[13] Cohort 1 & 2**<br>**(response options: *Always, Mostly, Sometimes, Never*)**				
1. I felt I had to do things in a certain way, like counting or saying special words, to stop something bad from happening. (R-LOI1)	.53	.58	.50	.47
2. I had trouble finishing my homework or other jobs because I had to do things over and over again. (R-LOI2)	.58	.63	.64	.53
3. I hated dirt and dirty things. (R-LOI3)	.35	.44	.43	.39
4. I had a special number that I counted up to, or I felt I had to do things just that number of times. (R-LOI4)	.40	.46	.42	.41
5. I often felt guilty or bad about things I had done even though no one else thought I had done anything wrong. (R-LOI5)	.71	.77	.79	.73
6. I worried about being clean enough. (R-LOI6)	.48	.51	.55	.45

For peer review only - http://bmjopen.bmj.com/site/about/guidelines.xhtml

7. I moved or talked in a special way to avoid bad luck. (R-LOI7)	.38	.46	.38	.33
8. I worried a lot if I did something, not exactly the way I liked. (R-LOI8)	.60	.67	.66	.53
9. I was fussy about keeping my hands clean. (R-LOI9)	.35	.40	.41	.35
10. I had special numbers or words that I said because I hoped they kept bad luck or bad things away. (R-LOI10)	.43	.47	.47	.42
11. I kept thinking about the things that I had done because I wasn't sure that they were the right things to do. (R-LOI11)	.71	.73	.71	.67

For peer review only

For peer review only - http://bmjopen.bmj.com/site/about/guidelines.xhtml

**Antisocial Behaviour Questionnaire (ABQ)[14] Cohort 1 & 2** **(response options: *Always, Mostly, Sometimes, Never*)**				
1. I deliberately broke the rules or disobeyed people (e.g. parents, teachers or supervisors). (ABQ1)	.45	.48	.47	.38
2. I stole things (e.g. from home or a shop or school). (ABQ2)	.37	.40	.36	.26
3. I deliberately damaged property (e.g. broke windows or chairs or wrote graffiti or started fires). (ABQ3)	.35	.39	.39	.38
4. I skipped lessons/work, skived, or played truant from school. (ABQ5)	.36	.39	.40	.35
5. I deliberately lied or cheated to get what I wanted. (ABQ6)	.43	.39	.41	.40
6. I ran away from home (e.g. for half a day or overnight). (ABQ7)	.51	.56	.58	.56
**Rosenberg Self-Esteem Questionnaire (RSEQ)[15] Cohort 1 & 2** **(response options: *Always, Mostly, Sometimes, Never*)**				
1. At times, I thought I was no good at all. (RSEQ1)	.82	.84	.85	.83
2. I was satisfied with myself. (RSEQ2)	-.58	-.61	-.60	-.53
3. I felt I had a number of good qualities. (RSEQ3)	-.53	-.55	-.56	-.52
4. I was able to do things as well as most people. (RSEQ4)	-.56	-.60	-.62	-.56
5. I felt I did not have much to be proud of. (RSEQ5)	.70	.73	.72	.70
6. I certainly felt useless at times. (RSEQ6)	.79	.81	.79	.77
7. I felt that I was as good as anyone else. (RSEQ7)	-.53	-.56	-.54	-.44
8. I wished I could have more respect for myself. (RSEQ8)	.62	.66	.68	.69
9. I felt that I was a failure. (RSEQ9)	.80	.82	.83	.75
10. I took a positive attitude toward myself. (RSEQ10)	-.60	-.63	-.63	-.56
**Warwick-Edinburgh Mental Well-Being Scale (WEMWBS)[16] Cohort 1 & 2** **(response options: *None of the time, Rarely, Some of the time, Often, All of the time*)**				
1. I've been feeling optimistic about the future. (WEMWBS1)	-.46	-.51	-.54	-.25
2. I've been feeling useful. (WEMWBS2)	-.52	-.58	-.60	-.33
3. I've been feeling relaxed. (WEMWBS3)	-.57	-.62	-.63	-.49
4. I've had the energy to spare. (WEMWBS5)	-.40	-.46	-.49	-.36
5. I've been dealing with problems well. (WEMWBS6)	-.57	-.63	-.64	-.46
6. I've been thinking clearly. (WEMWBS7)	-.62	-.67	-.68	-.48
7. I've been feeling good about myself. (WEMWBS8)	-.65	-.71	-.70	-.55
8. I've been feeling close to other people. (WEMWBS9)	-.44	-.50	-.52	-.28

For peer review only

9. I've been feeling confident. (WEMWBS10)	-.58	-.63	-.66	-.46
10. I've been able to make up my own mind about things. (WEMWBS11)	-.52	-.59	-.60	-.39
11. I've been feeling loved. (WEMWBS12)	-.49	-.54	-.60	-.29
12. I've been interested in new things. (WEMWBS13)	-.36	-.45	-.46	-.20
13. I've been feeling cheerful. (WEMWBS14)	-.61	-.67	-.67	-.49
**Psychotic-Like Experiences:**<br>**Cohort 1 – selected 10 items from the Schizotypal Personality Questionnaire (SPQ)**[17]<br>**Cohort 2 – selected 7 items from the Diagnostic Interview Schedule for Children (DISC)**[18]<br>**(response options: *Yes, No*)**				
1. Have you often mistaken objects or shadows for people or noises for voices? (SPQ4) [Cohort 1]	.38	.43	.41	*Not used*
2. I am sure I am being talked about behind my back. (SPQ9, DISC3) [Cohort 1 & 2]	.59	.67	.66	.60
3. Have you ever had the sense that some person or force is around you, even though you cannot see anyone? (SPQ13, DISC5) [Cohort 1 & 2]	.33	.38	.34	.41
4. Have you ever noticed a common event or object that seemed to be a special sign for you? (SPQ28, DISC8) [Cohort 1 & 2]	.33	.33	.35	.38
5. I often hear a voice speaking my thoughts aloud. (SPQ31, DISC10) [Cohort 1 & 2]	.33	.39	.34	.40
6. Have you ever seen things invisible to other people? (SPQ40, DISC13) [Cohort 1 & 2]	.36	.50	.37	.48
7. Do you sometimes feel that other people are watching you? (SPQ60, DISC19) [Cohort 1 & 2]	.53	.55	.59	.54
8. Do you ever suddenly feel distracted by distant sounds that you are not normally aware of? (SPQ61) [Cohort 1]	.40	.49	.45	*Not used*
9. Do you sometimes feel that people are talking about you? (SPQ63, DISC15) [Cohort 1 & 2]	.52	.56	.59	.60
10. Are your thoughts sometimes so strong that you can almost hear them? (SPQ64) [Cohort 1]	.44	.52	.50	*Not used*

For peer review only

Supplementary Table 2: Predictive power of Common Mental Distress versus the conventional psychopathology dimensions in Cohort 1_{T1} and Cohort 2: AUC (for ST and NSSI as criteria) and ORs for continuous and binary predictors (with cut-off point of 1SD)

		AUC		Suicidal thought (ST)				Non-suicidal self-injury (NSSI)			
				Continuous predictor		Binary (1SD cut-off)		Continuous predictor		Binary (1SD cut-off)	
		ST	NSSI	OR	95% C.I.	OR	95% C.I.	OR	95% C.I.	OR	95% C.I.
Common Mental Distress	Cohort 1_{T1}	.87	.83	7.07	[5.66 - 8.84]	15.60	[11.56 - 21.06]	4.15	[3.44 - 5.01]	8.93	[6.63 - 12.03]
	Cohort 2	.88	.72	6.79	[4.51 - 10.21]	20.97	[6.47 - 67.92]	2.38	[1.90 - 2.98]	4.00	[2.55 - 6.28]
Depression	Cohort 1_{T1}	.88	.83	5.10	[4.28 - 6.07]	15.60	[11.56 - 21.06]	3.21	[2.77 - 3.72]	8.28	[6.15 - 11.14]
	Cohort 2	.88	.70	7.18	[4.77 - 10.80]	15.32	[8.52 - 27.57]	2.14	[1.73 - 2.64]	3.56	[2.32 - 5.46]
Anxiety	Cohort 1_{T1}	.85	.81	4.82	[4.04 - 5.75]	13.62	[10.11 - 18.34]	3.75	[3.16 - 4.45]	7.61	[5.67 - 10.22]
	Cohort 2	.86	.71	5.69	[3.90 - 8.29]	10.51	[5.89 - 18.73]	2.24	[1.81 - 2.77]	3.68	[2.39 - 5.67]
Self-esteem (reversed)	Cohort 1_{T1}	.85	.83	4.81	[4.00 - 5.79]	15.62	[11.49 - 21.23]	3.75	[3.16 - 4.45]	9.86	[7.28 - 13.35]
	Cohort 2	.87	.65	6.42	[4.24 - 9.74]	15.16	[8.32 - 27.62]	1.79	[1.45 - 2.21]	3.34	[2.20 - 5.07]
Well-being (reversed)	Cohort 1_{T1}	.82	.80	4.29	[3.59 - 5.13]	10.31	[8.06 - 13.19]	3.45	[2.90 - 4.09]	6.66	[4.93 - 8.99]
	Cohort 2	.78	.61	2.88	[2.11 - 3.93]	5.27	[3.01 - 9.24]	1.44	[1.18 - 1.76]	2.19	[1.40 - 3.42]
Psychotic-like experiences	Cohort 1_{T1}	.74	.73	2.70	[2.32 - 3.13]	4.94	[3.70 - 6.60]	2.36	[2.03 - 2.74]	4.03	[2.98 - 5.45]
	Cohort 2	.74	.71	2.65	[2.00 - 3.50]	6.78	[3.89 - 11.83]	2.11	[1.72 - 2.58]	4.11	[2.69 - 6.27]
Antisocial trait*	Cohort 1_{T1}	.64	.63	1.65	[1.45 - 1.88]	2.67	[1.96 - 3.63]	1.79	[1.56 - 2.05]	2.48	[1.78 - 3.47]
Schizotypal trait	Cohort 1_{T1}	.79	.78	3.14	[2.71 - 3.64]	6.26	[4.70 - 8.32]	2.77	[2.39 - 3.21]	6.08	[4.52 - 8.19]
	Cohort 2	.76	.72	1.98	[1.66 - 2.36]	5.66	[3.23 - 9.91]	2.41	[1.93 - 3.01]	4.45	[2.90 - 6.83]
Conduct problems	Cohort 1_{T1}	.69	.67	1.87	[1.66 - 2.10]	3.38	[2.52 - 4.52]	1.67	[1.49 - 1.87]	3.46	[2.54 - 4.71]
	Cohort 2	.68	.61	2.00	[1.58 - 2.53]	3.78	[2.16 - 6.63]	1.54	[1.29 - 1.84]	2.13	[1.36 - 3.34]
Obsessions & compulsions	Cohort 1_{T1}	.76	.72	2.18	[1.94 - 2.45]	5.74	[4.25 - 7.75]	1.76	[1.57 - 1.98]	3.55	[2.58 - 4.89]
	Cohort 2	.71	.63	1.57	[1.31 - 1.88]	4.16	[2.37 - 7.28]	2.11	[1.64 - 2.71]	2.75	[1.79 - 4.22]

** measures were available only for Cohort 1_{T1}*

For peer review only

Supplementary Table 3: Association between ST and demographic variables in Cohort 1 (T1, T2, T3) and Cohort 2 (polychoric correlations)

	ST Cohort 1			ST Cohort 2
	T1	T2	T3	
Socioeconomic status (IMD)	-.05	-.01	-.01	.02
Research centre (0-Cambridge, 1-London)	-.12	-.04	-.03	not applicable
Ethnicity (1-white; 0-other)	-.08	-.02	-.04	-0.01
Age	-.05	-.02	-.05	0.03
Gender (0-Female, 1-Male)	-.10	-.08	-.01	0.03

All p-values non-significant

Supplementary Table 4: Association between NSSI and demographic variables in Cohort 1 (T1, T2, T3) and Cohort 2 (polychoric correlations)

	Cohort 1			Cohort 2
	T1	T2	T3	
Socioeconomic status (IMD)	.00	.00	.02	-.01
Research centre (0-Cambridge, 1-London)	-.01	-.01	.00	not applicable
Ethnicity (1-white; 0-other)	.00	.00	.00	.00
Age	-.02	-.04	-.01	-.02
Gender (0-Female, 1-Male)	.05	-.23	.02	.08

All p-values non-significant

Supplementary Table 5: Association between CMD and demographic variables in Cohort 1 (T1, T2, T3) and Cohort 2 (polychoric correlations)

	Cohort 1			Cohort 2
	T1	T2	T3	
Socioeconomic status (IMD)	-.02	-.02	-.01	.02
Research centre (0-Cambridge, 1-London)	.07*	.01	.01	not applicable
Ethnicity (1-white; 0-other)	-.08**	-.04	-.04	.04
Age	.01	.01	.01	.01
Gender (0-Female, 1-Male)	-.15**	-.15*	-.11**	.20**

*p<.01, **p<.001

For peer review only - http://bmjopen.bmj.com/site/about/guidelines.xhtml

Supplementary Table 6: Test of change in the prevalence of NSSI in Cohort 1: frequency over three time points (chi-square test)

	T1	T2	T3
NSSI	223	199	197
No-NSSI	2180	2204	2206

Chi-square=2.22, *df*=2, *p*=0.32, Yates' chi-square =2.04, *p*=0.35

Supplementary Table 7: Test of change in the prevalence of ST in Cohort 1: frequency over three time points (chi-square test)

	T1	T2	T3
NSSI	243	274	281
No-NSSI	2160	2129	2122

Chi-square=3.45, *df*=2, *p*=0.17, Yates' chi-square =3.26, *p*=0.19

For peer review only

For peer review only - http://bmjopen.bmj.com/site/about/guidelines.xhtml

Supplementary Table 8: Association between attrition in Cohort 1 at T2 and T3 and other variables in the study (Spearman rho)

	Attrition Cohort 1	
T1 variables:	**T2**	**T3**
Socioeconomic status (IMD index)[#]	-.07**	-.05*
Research centre (0-Cambridge, 1-London)	.05*	.05*
Ethnicity (1-white; 0-other)	-.05*	-.05*
Age	.07**	.05*
Gender (0-Female, 1-Male)	.09**	.12**
NSSI	-.01	.00
ST	-.01	-.03
Common Mental Distress	.06*	.05*
Depression	.06**	.05*
Impulsivity	.10**	.14**
Anxiety	.04*	.04*
Self - esteem (reversed)	.07**	.06*
Well - being (reversed)	.06*	.05*
Psychotic - like experiences even coerced	.00	.01
Antisocial trait	.08**	.12**
Schizotypal trait	.04*	.03
Conduct problems	.10**	.13**
Obsessions & compulsions	.03	.03

**$p<.001$, *$p<.01$
[#]higher number indicated *lower* socioeconomic deprivation

For peer review only

Supplementary Table 9: Direct and indirect effects in mediation (pathway) models in a female (F), male (M) and total (T) sample

		Standardised				Non- standardised			
		Coeff.	S.E.	Lower 95% C.I.	Upper 95% C.I.	Coeff.	S.E.	Lower 95% C.I.	Upper 95% C.I.
NSSI$_{T1}$->CMD$_{T2}$	F	.14***	.03	.09	.19	.46***	.09	.30	.61
	M	13***	.03	.07	.18	.56***	.14	.32	.80
	T	.15***	.02	.11	.18	.53***	.07	.40	.66
NSSI$_{T1}$->NSSI$_{T3}$	F	.16**	.05	.07	.25	.54**	.17	.25	.83
	M	.14**	.05	.05	.23	.65**	.24	.25	1.05
	T	.15**	.03	.09	.22	.58**	.14	.34	.82
NSSI$_{T1}$->ST$_{T3}$	F	.07	.05	.00	.16	.27	.17	-.01	.56
	M	.04	.05	-.03	.13	.22	.24	-.18	.62
	T	.05	.03	.00	.12	.22	.15	-.02	.47
ST$_{T1}$->CMD$_{T2}$	F	.25***	.03	.19	.30	.83***	.10	.66	1.00
	M	.24***	.03	.18	.30	.85***	.11	.65	1.05
	T	.24***	.02	.20	.28	.83***	.07	.70	.96
ST$_{T1}$->NSSI$_{T3}$	F	.10*	.05	.01	.20	.38	.19	.05	.70
	M	.07	.06	-.03	.17	.25	.23	-.13	.64
	T	.19*	.04	.13	.25	.33	.16	.06	.60
ST$_{T1}$->ST$_{T3}$	F	.20***	.04	.13	.27	.76***	.16	.49	1.03
	M	.17***	.05	.08	.25	.66***	.19	.33	.98
	T	.19**	.03	.13	.25	.72***	.13	.50	.95
CMD$_{T2}$-> NSSI$_{T3}$	F	.22***	.07	.11	.34	.24**	.07	.11	.37
	M	.21*	.08	.07	.34	.21*	.09	.06	.36
	T	.22***	.06	.11	.32	.22***	.06	.11	.34
CMD$_{T2}$-> ST$_{T3}$	F	.32***	.05	.22	.41	.35***	.07	.23	.47
	M	.35***	.06	.25	.46	.39***	.07	.26	.51
	T	.33***	.04	.25	.40	.35***	.05	.26	.45
NSS$_{T1}$<->ST$_{T1}$	F	.40***	.02	.36	.45	.04***	.00	.03	.04
	M	.32***	.03	.26	.37	.02***	.00	.01	.03
	T	.37***	.02	.33	.40	.03***	.00	.02	.03
NSSI$_{T3}$<->ST$_{T3}$	F	.67***	.07	.55	.79	.67***	.07	.55	.79
	M	.57***	.10	.39	.75	.57***	.10	.39	.75
	T	.63***	.07	.51	.75	.63***	.07	.51	.75
NSSI$_{T1}$->CMD$_{T2}$ ->NSSI$_{T3}$	F	.03**	.01	.01	.05	.11**	.04	.04	.18
	M	.02*	.01	.00	.05	.12*	.06	.02	.22

For peer review only

For peer review only - http://bmjopen.bmj.com/site/about/guidelines.xhtml

	T	**.03***	.01	.01	.05	***.12****	.04	.05	.19
**ST_{T1}->CMD_{T2}->$NSSI_{T3}$**	F	**.05****	.02	.02	.09	***.20****	.07	.08	.32
	M	**.05***	.02	.01	.08	***.18****	.08	.05	.31
	T	**.05****	.01	.02	.07	***.19******	.05	.09	.28
**$NSSI_{T1}$->CMD_{T2}->ST_{T3}**	F	**.04*****	.01	.02	.06	***16******	.04	.08	.24
	M	**.04*****	.01	.02	.07	***.22****	.07	.10	.33
	T	**.05****	.01	.03	.06	***.19******	.04	.12	.26
**ST_{T1}->CMD_{T2}->ST_{T3}**	F	**.08*****	.01	.05	.11	***.29******	.07	.17	.41
	M	**.08*****	.02	.05	.12	***.33******	.08	.20	.47
	T	**.08****	.01	.05	.10	***.30******	.05	.20	.39

*Significance levels:**p<.05, **p<.01, ***p<.001

For peer review only

Item Response Theory (IRT) analysis

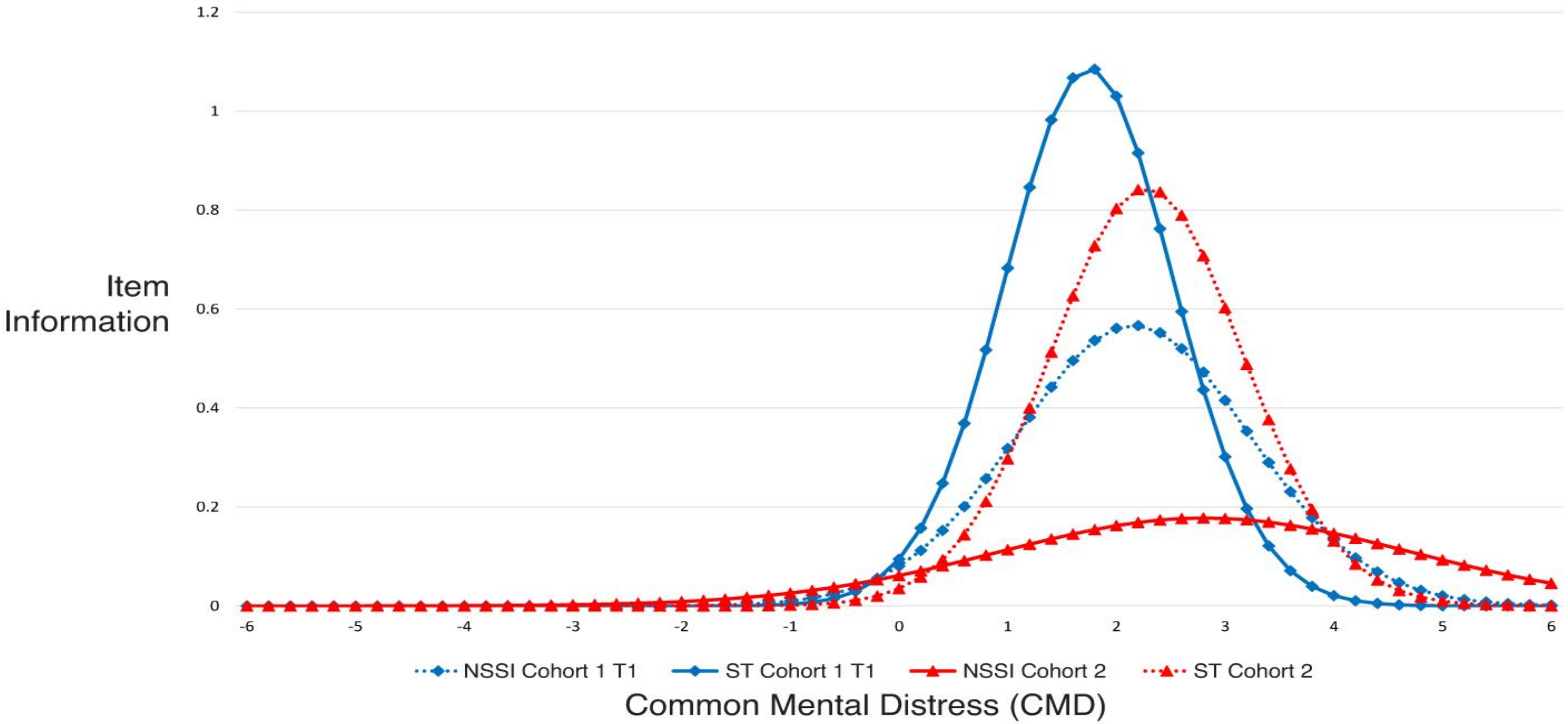

Supplementary Figure 1: Hierarchy of symptoms: the place of non-suicidal self-harm (NSSI) and suicidal though (ST) on the latent continuum of Common Mental Distress (in standard deviations) in Cohort 1$_{T1}$ and Cohort 2.

Item Response Theory (IRT) analysis is concerned, broadly speaking, with investigating the relationship between items and the latent construct. Here we computed item response function showing how much information NSSI and ST (here treated as indicators of CMD) contribute to the latent variable – CMD. The above graph shows that NSSI and ST provided information in above-average to high ranges of CMD, with the peak of the information curves for NSSI occurring around +2 SD in both cohorts. The information curve for ST in Cohort 2 was flatter, suggesting less contribution to the latent CMD dimension than ST had in Cohort 1$_{T1}$ dataset. This may be due to the differences in age structure and psychopathology status in both cohorts. Nonetheless, in both cohorts the peak in the ST curves occurred between +2 and +3 SD (high end of the CMD dimension), showing that ST lies on the more severe spectrum of CMD dimension than NSSI does.

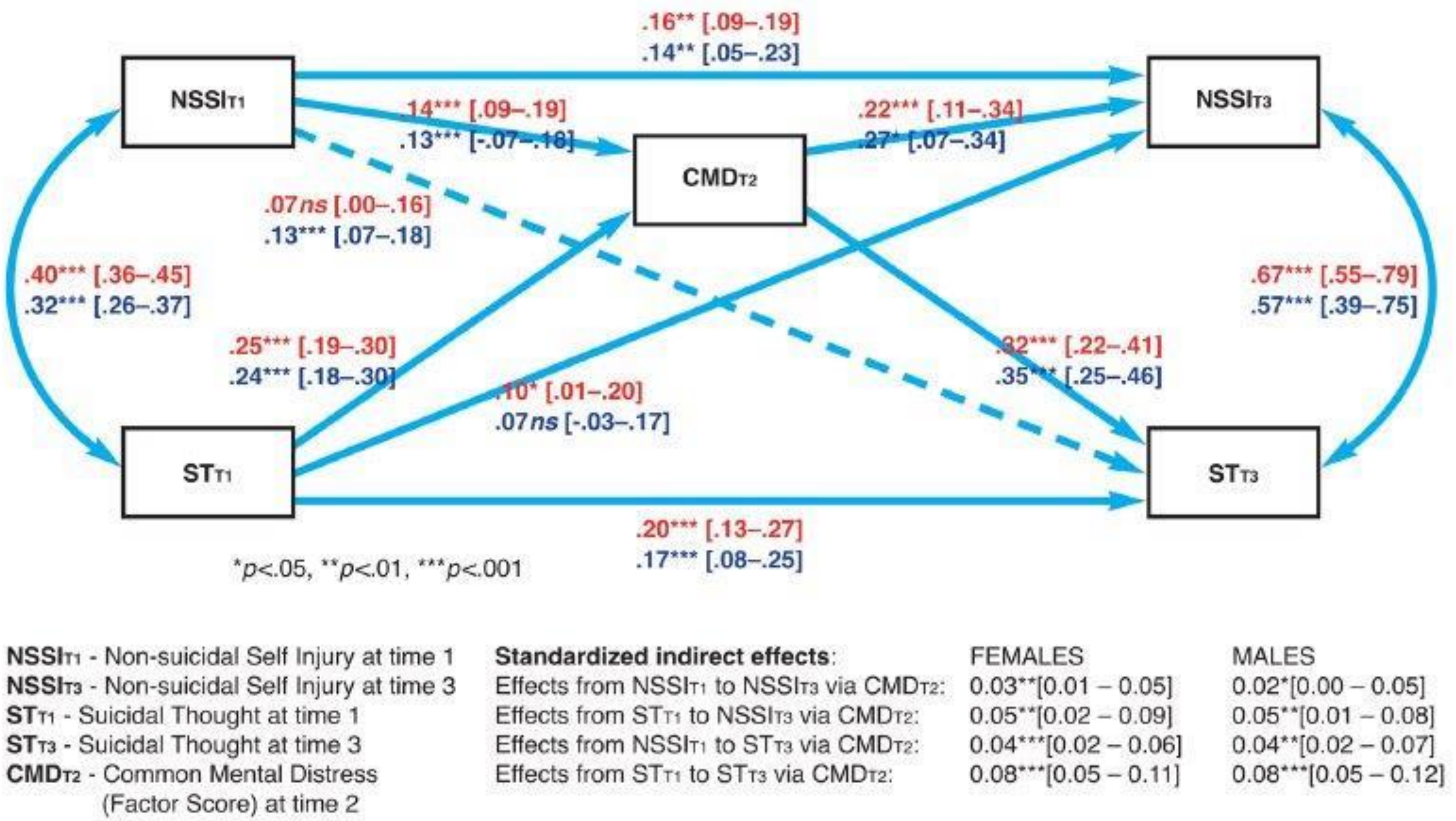

Supplementary Figure 2: Mediation effect of Common Mental Distress at time 2 (CMD_{T2}) moderated by sex (female n=1286 (red colour); male n=1115 (blue colour)) in the Cohort 1

Standardised pathway coefficients (with confidence intervals reported in squarer brackets) were obtained in multiple group pathway analysis in which sex was treated as a grouping variable. We tested the equivalence in pathway coefficients by means of comparing chi-square tests when the coefficient was "fixed" to be equal across sexes versus when it was free to vary across sexes[2]. We also tested the equivalence of fit indices of the model in both sexes. We found no evidence for differences in individual pathway coefficients or fit indices between sexes. This suggests that CMD at T2 mediated the longitudinal persistence of NSSI and ST in the same manner in females and males – no evidence of sex differences in the longitudinal mediation process was found. Additional details are reported in Supplementary Table 10.

Age and gender: Descriptive analysis

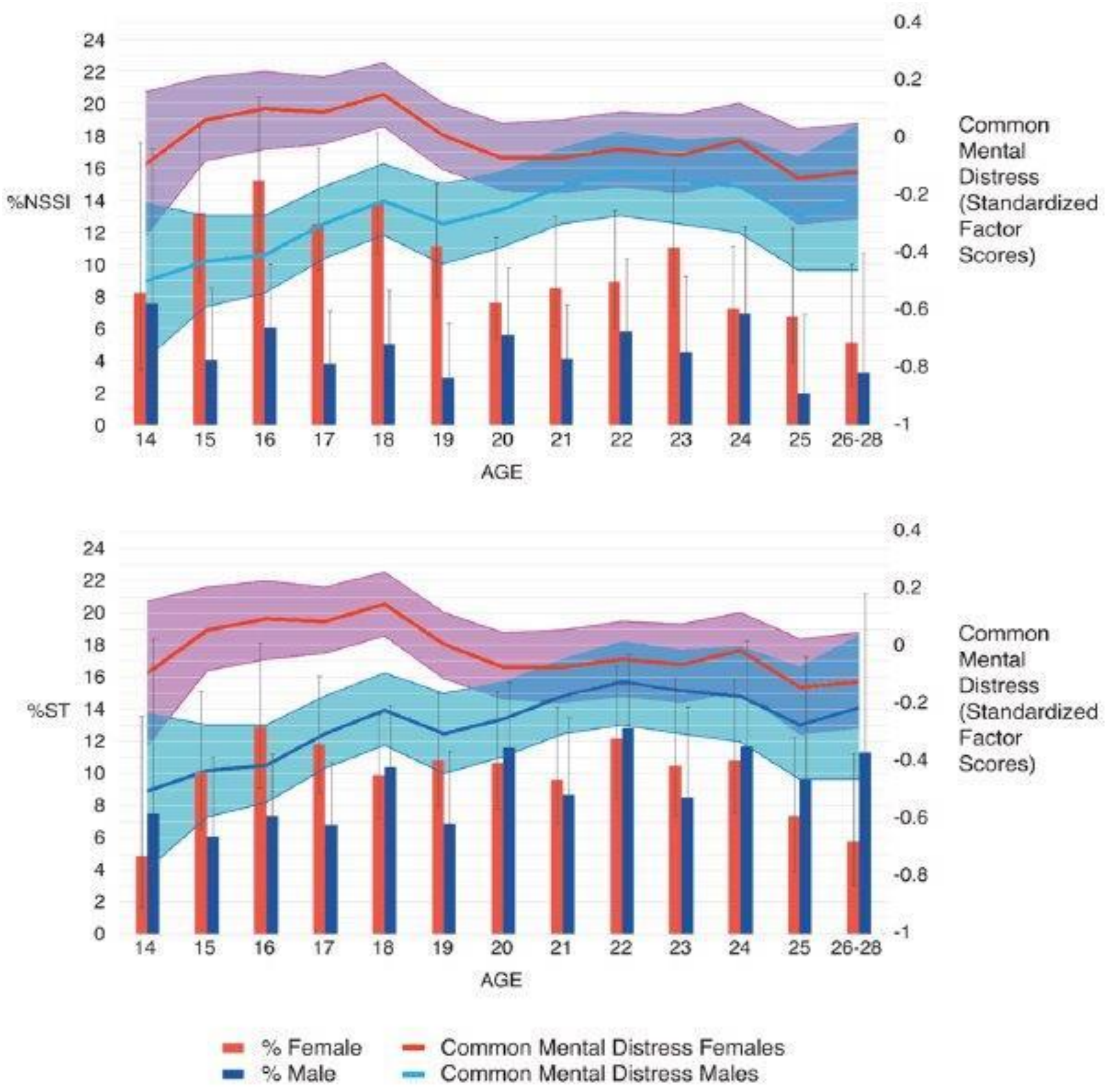

Supplementary Figure 3. Percentages of non-suicidal self-injury (NSSI), suicidal thoughts (ST) and levels of Common Mental Distress in age groups for both sexes in Cohort 1

To analyse the relationship between age, sex, NSSI, ST, and CMD descriptively, we grouped observations from all 3 time points in Cohort $1_{T1\text{-}T3}$ by age, rather than by data time point. This grouping allowed us to investigate levels of CMD, NSSI and ST in a broad age range of 14-28 years (note that this also entailed the inclusion of the same individuals from consecutive data sweeps (e.g., when an individual was 14, 15 and 16 years old) in the adjacent age groups). The histograms showing percentages of NSSI and ST with Wilson confidence intervals were plotted against the lines representing the means of CMD with confidence intervals for every age group for both sexes separately (Figure 3 above).

For peer review only - http://bmjopen.bmj.com/site/about/guidelines.xhtml

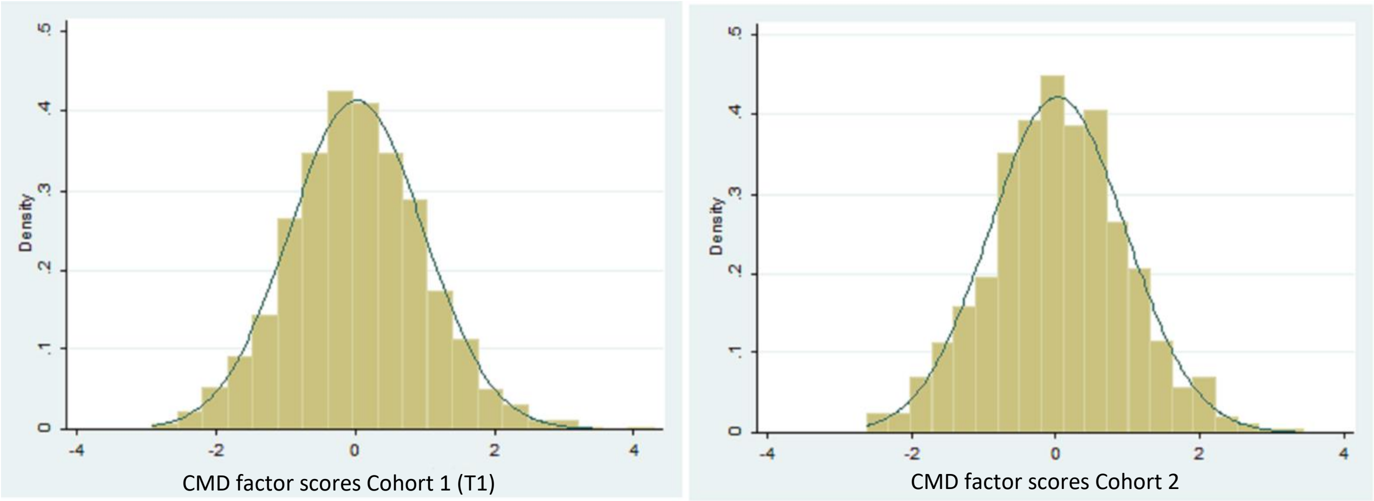

Supplementary Figure 4: Histograms of CMD factor scores in Cohort 1 (T1) and Cohort 2 with a schematic normal distribution line

For peer review only - http://bmjopen.bmj.com/site/about/guidelines.xhtml

Data collection tools:

Study data were collected and managed using REDCap electronic data capture tools[19] hosted at the University of Cambridge. REDCap (Research Electronic Data Capture) is a secure, web-based application designed to support data capture for research studies, providing 1) an intuitive interface for validated data entry; 2) audit trails for tracking data manipulation and export procedures; 3) automated export procedures for seamless data downloads to common statistical packages; and 4) procedures for importing data from external sources.

Group Information

NSPN (NeuroScience in Psychiatry Network: http://www.nspn.org.uk/) is a research consortium formed by the University of Cambridge and University College London, launched in November 2012 and supported by Wellcome Trust Award (095844/Z/11/Z). The group included the following members:

Principal investigators:

Edward Bullmore (CI from 01/01/2017)[1,2]

Ian Goodyer (CI until 01/01/2017)[1]

Peter Jones[1,2,3]

Raymond Dolan[4,5]

Peter Fonagy[6]

NSPN (funded) staff:

Michael Moutoussis[4,5]

Tobias Hauser[4,5]

Sharon Neufeld[1]

Petra Vértes[1,2]

Kirstie Whitaker[1,2]

Gita Prabhu[4,5]

Laura Villis[1]

Junaid Bhatti[1]

Becky Inkster[1]

Cinly Ooi[1]

Barry Widmer[1]

Ayesha Alrumaithi[1]

Sarah Birt[1]

For peer review only - http://bmjopen.bmj.com/site/about/guidelines.xhtml

Kalia Cleridou[5]

Hina Dadabhoy[5]

Sian Granville[5]

Elizabeth Harding[5]

Alexandra Hopkins[4,5]

Daniel Isaacs[5]

Janchai King[5]

Danae Kokorikou[5,6]

Harriet Mills[5]

Ciara O'Donnell[1]

Sara Pantaleone[5]

Aislinn Bowler[5]

Affiliated scientists:

Pasco Fearon[6]

Anne-Laura van Harmelen[1]

Rogier Kievit[4,7]

1 Department of Psychiatry, University of Cambridge, United Kingdom

2 NIHR Applied Research Collaboration East of England, UK

3 NIHR Cambridge Biomedical Research Centre, UK

4 Max Planck University College London Centre for Computational Psychiatry and Ageing Research, University College London, UK

5 Wellcome Centre for Human Neuroimaging, University College London, United Kingdom

6 Research Department of Clinical, Educational and Health Psychology, University College London,

United Kingdom

7 Medical Research Council Cognition and Brain Sciences Unit, University of Cambridge, United Kingdom

For peer review only

References:

1 Reise, S. P. The rediscovery of bifactor measurement models. *Multivariate Behavioural Research*. 2012;47:667-696, doi:10.1080/00273171.2012.715555.

2 Muthen, L. & Muthen, B. *Mplus Users's Guide*. (Muthen & Muthen, 1998-2002).

3 Reise, S. P., Moore, T. M. & Haviland, M. G. Bifactor models and rotations: Exploring the extent to which multidimensional data yield univocal scale scores. *Journal of Personality Assessment*. 2010;92:544-559, doi:10.1080/00223891.2010.496477.

4 Noble M, McLennan, D, Wilkinson K, Whitworth A, & Barne H. The English Indices of Deprivation 2007. London: Department for Communities and Local Government. (2008).

5 van Buuren, S. & Groothuis-Oudshoorn, K. MICE: Multivariate Imputation by Chained Equations in R. *Journal of Statistical Software*.2011;45,1-67.

6 Sterne, J. A. C. *et al.* Multiple imputation for missing data in epidemiological and clinical research: Potential and pitfalls. *British Medical Journal.* 2009;339:157-160.

7 Rubin, D. B. *Multiple imputation for nonresponse in surveys.* (Wiley, 1987).

8 Hammerton, G., Zammit, S., Potter, R., Thapar, A. & Collishaw, S. Validation of a composite of suicide items from the Mood and Feelings Questionnaire (MFQ) in offspring of recurrently depressed parents. . *Psychiatry Research.* 2014;216:82-88.

9 Wilkinson P.O., Qiu T., Neufeld S., Jones P.B. & Goodyer I.M. Sporadic and recurrent non-suicidal self-injury before age 14 and incident onset of psychiatric disorders by 17 years: prospective cohort study. *British Journal of Psychiatry*.2018;212:222-226, doi:10.1192/bjp.2017.45.

10 Cassels M. *et al.* Poor family functioning mediates the link between childhood adversity and adolescent non-suicidal self-injury. *Journal of Child Psychology and Psychiatry*. 2018;59(8):881-887. doi: 10.1111/jcpp.12866

11 Angold, A. *et al.* The development of a short questionnaire for use in epidemiological studies of depression in children and adolescents. *International Journal of Methods in Psychiatric Research*.1995;5:237-249.

12 Reynolds, C. R. Concurrent validity of what I think and feel: The revised children's manifest anxiety scale. *Journal of Consulting and Clinical Psychology*. 1980;48:774-775. doi:10.1037/0022-006x.48.6.774 (1980).

13 Bamber, D., Tamplin, A., Park, R. J., Kyte, Z. A. & Goodyer, I. M. Development of a short Leyton Obsessional Inventory For Children and Adolescents. *Journal of the American Academy of Child and Adolescent Psychiatry*.2002;41:1246-1252.

14 St Clair C. M. *et al.* Characterising the latent structure and organisation of self-reported thoughts, feelings and behaviours in adolescents and young adults. *PLOS One.* 2017;12:1-27, doi:https://doi.org/10.1371/journal.pone.0175381.

15 Rosenberg, M. *Society and the adolescent self-image*. (Princeton University Press, 1965).

16 Tennant, R. *et al.* The Warwick-Edinburgh mental well-being scale (WEMWBS): development and UK validation. *Health and Quality of Life Outcomes* **5**, doi:10.1186/1477-7525-5-63 (2007).

17 Raine, A. The SPQ: A scale for the assessment of schizotypal personality based on DSM-III-R criteria. *Schizophrenia Bulletin*.1991;17:555-564.

18 Shaffer, D., Fisher, P., Lucas, C. P., Dulcan, M. K. & Schwab-Stone, M. E. NIMH Diagnostic Interview Schedule for Children Version IV (NIMH DISC-IV): description, differences from previous versions, and reliability of some common diagnoses. *Journal of the American Academy of Child and Adolescent Psychiatry*. 2000;39:28-38.

19 Harris PA, Taylor R, Thielke R, Payne J, Gonzalez N, Conde JG. Research electronic data capture (REDCap) – A metadata-driven methodology and workflow process for providing translational research informatics support. *Journal of Biomedical Informatics.* 2009;42(2):377-81.

For peer review only - http://bmjopen.bmj.com/site/about/guidelines.xhtml

STROBE (Strengthening The Reporting of OBservational Studies in Epidemiology) Checklist

A checklist of items that should be included in reports of observational studies. You must report the page number in your manuscript where you consider each of the items listed in this checklist. If you have not included this information, either revise your manuscript accordingly before submitting or note N/A.

Note: An Explanation and Elaboration article discusses each checklist item and gives methodological background and published examples of transparent reporting. The STROBE checklist is best used in conjunction with this article (freely available on the Web sites of PLoS Medicine at http://www.plosmedicine.org/, Annals of Internal Medicine at http://www.annals.org/, and Epidemiology at http://www.epidem.com/). Information on the STROBE Initiative is available at www.strobe-statement.org.

Section and Item	Item No.	Recommendation	Reported on Page No.
**Title and Abstract**	1	(*a*) Indicate the study's design with a commonly used term in the title or the abstract	1
		(*b*) Provide in the abstract an informative and balanced summary of what was done and what was found	2-3
**Introduction**			
Background/Rationale	2	Explain the scientific background and rationale for the investigation being reported	4-5
Objectives	3	State specific objectives, including any prespecified hypotheses	5
**Methods**			
Study Design	4	Present key elements of study design early in the paper	5-6
Setting	5	Describe the setting, locations, and relevant dates, including periods of recruitment, exposure, follow-up, and data collection	6-7
Participants	6	(*a*) *Cohort study*—Give the eligibility criteria, and the sources and methods of selection of participants. Describe methods of follow-up	6-7
		Case-control study—Give the eligibility criteria, and the sources and methods of case ascertainment and control selection. Give the rationale for the choice of cases and controls	not applicable
		Cross-sectional study—Give the eligibility criteria, and the sources and methods of selection of participants	not applicable
		(*b*) *Cohort study*—For matched studies, give matching criteria and number of exposed and unexposed	not applicable
		Case-control study—For matched studies, give matching criteria and the number of controls per case	not applicable
Variables	7	Clearly define all outcomes, exposures, predictors, potential confounders, and effect modifiers. Give diagnostic criteria, if applicable	7-8

For peer review only

Section and Item	Item No.	Recommendation	Reported on Page No.
Data Sources/ Measurement	8*	For each variable of interest, give sources of data and details of methods of assessment (measurement). Describe comparability of assessment methods if there is more than one group	Suppl. 3-8
Bias	9	Describe any efforts to address potential sources of bias	Suppl. 2, Ms.1
Study Size	10	Explain how the study size was arrived at	Not applicabl
Quantitative Variables	11	Explain how quantitative variables were handled in the analyses. If applicable, describe which groupings were chosen and why	Supp.1, Ms. 7-8
Statistical Methods	12	(*a*) Describe all statistical methods, including those used to control for confounding	Supp.1, Ms. 7-8
		(*b*) Describe any methods used to examine subgroups and interactions	Ms. 7-8
		(*c*) Explain how missing data were addressed	Suppl. 2
		(*d*) *Cohort study*—If applicable, explain how loss to follow-up was addressed	Suppl. 2
		Case-control study—If applicable, explain how matching of cases and controls was addressed	not applicable
		Cross-sectional study—If applicable, describe analytical methods taking account of sampling strategy	not applicable
		(*e*) Describe any sensitivity analyses	not applicable
**Results**			
Participants	13*	(a) Report numbers of individuals at each stage of study—eg numbers potentially eligible, examined for eligibility, confirmed eligible, included in the study, completing follow-up, and analysed	6
		(b) Give reasons for non-participation at each stage	not applicable
		(c) Consider use of a flow diagram	not applicable
Descriptive Data	14*	(a) Give characteristics of study participants (eg demographic, clinical, social) and information on exposures and potential confounders	Suppl.11, Ms.6
		(b) Indicate number of participants with missing data for each variable of interest	Suppl. 2
		(c) *Cohort study*—Summarise follow-up time (eg, average and total amount)	6
Outcome Data	15*	*Cohort study*—Report numbers of outcome events or summary measures over time	9
		Case-control study—Report numbers in each exposure category, or summary measures of exposure	not applicable
		Cross-sectional study—Report numbers of outcome events or summary measures	not applicable

For peer review only

Section and Item	Item No.	Recommendation	Reported on Page No.
Main Results	16	(*a*) Give unadjusted estimates and, if applicable, confounder-adjusted estimates and their precision (eg, 95% confidence interval). Make clear which confounders were adjusted for and why they were included	Fig. 1, Suppl.9
		(*b*) Report category boundaries when continuous variables were categorized	Suppl. 9
		(*c*) If relevant, consider translating estimates of relative risk into absolute risk for a meaningful time period	Fig. 2
Other Analyses	17	Report other analyses done—eg analyses of subgroups and interactions, and sensitivity analyses	Suppl.9, 16
**Discussion**			
Key Results	18	Summarise key results with reference to study objectives	10-11
Limitations	19	Discuss limitations of the study, taking into account sources of potential bias or imprecision. Discuss both direction and magnitude of any potential bias	12
Interpretation	20	Give a cautious overall interpretation of results considering objectives, limitations, multiplicity of analyses, results from similar studies, and other relevant evidence	13-14
Generalisability	21	Discuss the generalisability (external validity) of the study results	13-14
**Other Information**			
Funding	22	Give the source of funding and the role of the funders for the present study and, if applicable, for the original study on which the present article is based	15

*Give information separately for cases and controls in case-control studies and, if applicable, for exposed and unexposed groups in cohort and cross-sectional studies.

Once you have completed this checklist, please save a copy and upload it as part of your submission. DO NOT include this checklist as part of the main manuscript document. It must be uploaded as a separate file.

For peer review only
